# Identifying environmental risk factors for inflammatory bowel diseases: a Mendelian randomization study

**DOI:** 10.1038/s41598-020-76361-2

**Published:** 2020-11-06

**Authors:** Robert Carreras-Torres, Gemma Ibáñez-Sanz, Mireia Obón-Santacana, Eric J. Duell, Victor Moreno

**Affiliations:** 1 Colorectal Cancer Group, ONCOBELL Program, Bellvitge Biomedical Research Institute (IDIBELL), Hospitalet de Llobregat, Avinguda de La Granvia de L’Hospitalet, 199, 08908 Barcelona, Spain; 2 Unit of Biomarkers and Susceptibility, Oncology Data Analytics Program, Catalan Institute of Oncology (ICO). Hospitalet de Llobregat, Barcelona, Spain; 3 Consortium for Biomedical Research in Epidemiology and Public Health (CIBERESP), Barcelona, Spain; 4 Department of Clinical Sciences, Faculty of Medicine, University of Barcelona, Barcelona, Spain

**Keywords:** Inflammatory bowel disease, Crohn's disease, Ulcerative colitis, Risk factors

## Abstract

Several studies have examined environmental factors and inflammatory bowel diseases (IBD) using traditional approaches; however, provided results are still conflicting. Our aim was to determine whether lifestyle and nutrient exposures, related to IBD in observational meta-analyses, influence IBD risk using a Mendelian randomization (MR) approach. A two-sample MR approach was applied on summary-level genome-wide association results. Genetic variants strongly associated with measures of tobacco smoking, obesity and fat distribution, physical activity, and blood levels of vitamins and fatty acids were evaluated on genetic data from international IBD consortia including a total of 25,042 IBD cases (12,194 cases of Crohn’s disease (CD) and 12,366 cases of ulcerative colitis (UC)) and 34,915 controls. Our results indicated that, among lifestyle exposures, being a smoker was positively associated with CD (OR 1.13, P = 0.02), but it was not associated with UC risk (OR 0.99, P = 0.88). Body-mass index (BMI) and body fat percentage were positively associated with CD (OR 1.11, P = 0.02, per standard deviation (SD) of 4.6 kg/m^2^; and OR 1.50, P = 3 × 10^–10^, per SD of 6.6%; respectively); while for UC, BMI was inversely associated (OR 0.85, P = 5 × 10^–5^; per SD) and body fat percentage showed a OR of 1.11 (P = 0.11; per SD). Additionally, among nutrient exposures, omega-3 fatty acids levels were inversely associated with CD (OR 0.67, P = 2 × 10^–6^). Our MR results did not support a protective effect for being a smoker on UC risk; however, they are compatible with a risk effect for higher body fat proportion and a protective role for higher levels of omega-3 fatty acids on CD etiology.

## Introduction

Inflammatory bowel diseases (IBD) become chronic disorders as consequence of dysregulated immune response to intestinal dysbiosis in genetically susceptible individuals, with fluctuating periods between remission and relapses^1^. There exist two main types of IBD, namely Crohn’s disease (CD) and ulcerative colitis (UC). They differ, among other features, on the location of the affected mucosa; CD can affect discontinuously different regions of the gastrointestinal tract, typically ileum and colon, while UC starts in the rectum and may involve proximally the colon in a continuous fashion^2,3^.

IBD incidence and prevalence have stabilized in most economically developed areas, but continue to rise in areas in transition towards developed economies^4^. This links the IBD main risk factors to westernization of lifestyle. External environmental factors can contribute to gut dysbiosis, reducing microbiome diversity or introducing non-commensal microorganism, alter the integrity of the epithelial barrier, and trigger an altered immune response^5^. Environmental risk factors for gut dysbiosis and IBD are lifestyle parameters such as cigarette smoking, obesity, physical inactivity, and a western-type of diet (characterized by high intake of saturated fat, refined carbohydrates, red and processed meat, and low intake of fruits, vegetables, fiber, and fish)^6,7^. A recent umbrella review of meta-analyses identified risk and protective factors with different levels of strength of epidemiologic evidence; however, subgroup analyses showed differential associations for factors such as smoking and folate levels^7^.

**Figure 1:**
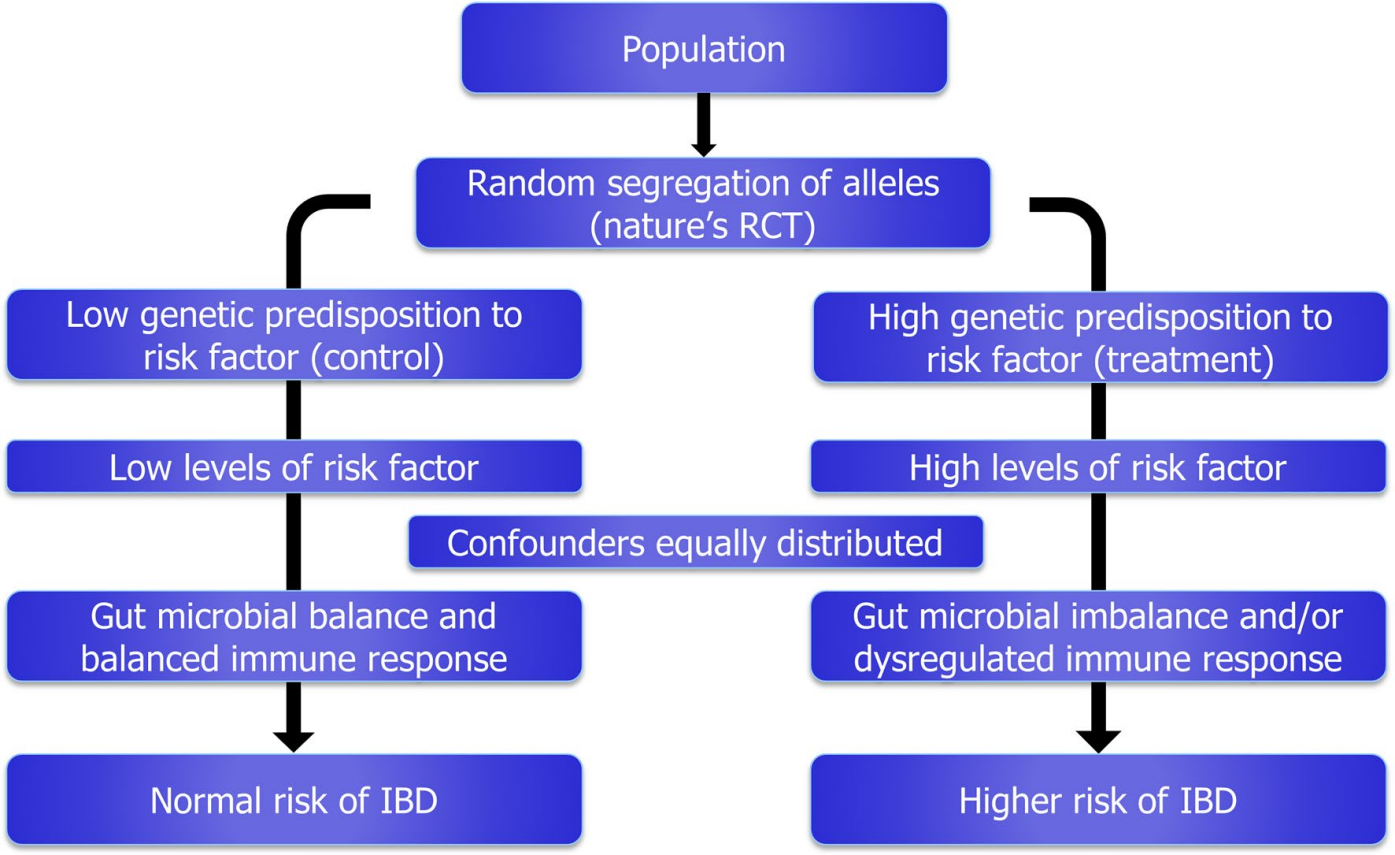
Mendelian randomization as an analogous approach to a classical randomized control trial (RCT).

Novel analytical methodologies aim to provide evidence of causality using genetic proxies for putative risk factors in instrumental variable analyses. Recently, one of these methods, called Mendelian randomization (MR), has become popular as large-scale genome-wide association studies (GWAS) allow the identification of genetic variants reliably associated with putative risk factors^8^. This methodology was proposed as analogous approach to a classical randomized controlled trial (RCT), the gold standard to establish causality. The random segregation of alleles at conception provides naturally two groups of population differentiated exclusively by the genetics of the putative risk factors (Fig. 1), mimicking the effect of the treatment and control arms in a RCT. Under the methodological assumptions, this approach avoids biasing effects from reverse causation (as disease onset does not affect germ-line genetics), and it is largely free from confounding (as the genetic basis of different risk factors are independent)^9^. The results from a MR approach allow a better estimation of the risk contribution of a putative risk factor, and support the development of preventive disease strategies.

In this study, for those environmental risk factors previously identified in large meta-analyses and with available GWAS data, we aimed to provide further evidence of potential causation using a two-sample MR approach. We reviewed and identified genome-wide variants associated with putative risk factors, and tested them on GWAS data from large-scale IBD international consortia comprising more than 50,000 individuals.

## Materials and methods

This study was based on summary-level genome-wide association results under a two-sample MR approach^10^. Initially, genetic variants strongly associated with each exposure were identified as instruments in large-scale GWAS results (first sample). Subsequently, for the identified instruments, association data on IBD and subtypes were retrieved from IBD international consortia results (second sample). Genetics-to-exposure results were combined with genetics-to-IBD results to obtain exposure-to-IBD effect estimates through a likelihood-based MR approach. The consistency of initial results was evaluated by applying several complementary MR analyses.

### Identification of genetic determinants for potential risk factors

Analyzed factors included measures of tobacco smoking, obesity and fat distribution, overall physical activity, and blood metabolite levels affected by a western-type of diet, such as vitamins and fatty acids.

Initially, genome-wide associated single nucleotide polymorphisms (SNPs) (P < 5 × 10^–8^) were identified as genetic instruments in European-based studies. Recently published GWAS using or including the European individuals from the UK Biobank cohort are a rich source of genetic instruments. UK Biobank is a prospective cohort that recruited more than 500,000 men and women aged 40–96 years between 2006 and 2010, and collected anthropometric, health and lifestyle data^11^. The genetic etiology of tobacco smoking was recently described in a GWAS^12^ comprising up to 1.2 million individuals from the Tobacco and Genetics (TAG) consortium, 23andMe database, and the UK Biobank cohort. From this study we identified genetic instruments for risk of ever being smoker (ever vs never smokers), and number of cigarettes smoked per day. Regarding obesity and fat distribution measures, genetic instruments for body-mass index (BMI)^13^ and waist-to-hip ratio^14^ were retrieved from meta-analyses combining GWAS results from the Genetic Investigation of ANthropometric Traits (GIANT) consortium and the UK Biobank cohort comprising nearly 700,000 individuals. Genome-wide associated SNPs for body fat percentage (total fat mass/ total mass)^15^ were identified from GWAS using nearly 360,000 UK Biobank individuals. Likewise, physical activity was recently genetically assessed in the UK Biobank cohort. Overall physical activity was measured using a wrist-worn accelerometer device in a subsample of 91,000 individuals^16^. Finally, we analyzed vitamins and fatty acids levels as metabolites affected by the type of dietary intake^17,18^. Genetic instruments were identified for circulating levels of vitamin D^19^, vitamin B9 and B12^20^, polyunsaturated omega-3 and omega-6, monounsaturated (mainly omega-7 and omega-9) fatty acids, and total fatty acids^21^.

From the initial sets of SNPs, we selected uncorrelated SNPs based on linkage disequilibrium (LD) R^2^ < 0.01. SNPs with ambiguous strand codification (A/T or C/G) were replaced by SNPs in genetic linkage (R^2^ > 0.8) using the *proxysnps* R package (European populations) (R Project). SNP-to-exposure association summary statistics were retrieved from the original GWAS studies, and can be observed in Supplementary Table S1 online. In the studies where summary statistics were not reported in standard deviations (SD), they were transformed based on SD reported in the original GWAS studies. Table 1 describes genetic instruments features on the number of SNPs included, the proportion of phenotype variance explained by the SNPs (or cumulative SNP liability in the case of binary outcomes), as well as the mean and SD of the respective exposure in the original GWAS studies.

**Table 1:** Description of genetic instruments for observed environmental risk factors.

Potential risk factor	n SNP	Mean	SD	Units	Var (%)	Pub ref	Minimum detectable OR		
							IBD	CD	UC
**Lifestyle**									
Smoking status	331	-	-	Ever/never	2.3	^12^	1.17	1.22	1.21
Cigarettes per day	44	2.35	0.94	n cigarettes	1.1	^12^	1.25	1.34	1.32
Body-mass index	816	26.97	4.65	Kg/m^2^	6.0	^13^	1.10	1.13	1.13
Waist-to-hip ratio	403	1	0.1	–	3.0	^14^	1.14	1.19	1.19
Body fat percentage (fat/total mass)	378	31.8	6.6	%	3.5	^15^	1.13	1.18	1.17
Overall physical activity	2	27.89	8.14	Mili-gravities (acceleration)	0.08	^16^	2.27	2.93	2.83
**Nutrients**									
Vitamin D	59	70.0	34.7	nmol/l	3.4	^19^	1.13	1.18	1.17
Vitamin B9 (folate)	2	13.4	8.44	nmol/l	1.0	^20^	1.26	1.36	1.34
Vitamin B12	10	342.68	150.11	pmol/L	6.3	^20^	1.10	1.13	1.12
Omega-3 fatty acids	4	0.41	0.08–1.80*	mmol/l	2.4	^21^	1.16	1.22	1.21
Omega-6 fatty acids	8	4	1.8–12.0*	mmol/l	4.6	^21^	1.11	1.15	1.15
Monounsaturated fatty acids (mainly omega-9 and omega-7)	4	3.3	1.1–15.0*	mmol/l	2.4	^21^	1.16	1.22	1.21
Total fatty acids	8	11.7	4.6–42.0*	mmol/l	3.7	^21^	1.13	1.17	1.17

*nSNP* number of single nucleotide polymorphisms, *SD* standard deviation, Var: explained phenotype variance, *Pub ref* discovery GWAS, *OR* odds ratio; *IBD* Inflammatory bowel disease; *CD* Crohn’s disease; *UC* ulcerative colitis.

*Range (minimum–maximum).

### GWAS data on inflammatory bowel diseases and subtypes

The largest GWAS on IBD and subtypes was based on meta-analyses results of European samples from the International IBD Genetics Consortium and the UK IBD Genetics Consortium^22^. This study comprised a total of 25,042 cases of IBD, including 12,194 cases of CD and 12,366 cases of UC, and 34,915 controls. SNP-to-IBD and subtypes association summary statistics for the selected SNPs were retrieved from this GWAS study, and can be observed in Supplementary Table S1 online.

### Statistical analyses

Following the method proposed by Burgess^23^, a priori power calculations were performed for MR associations of nominal statistical significance (α < 0.05). Power estimates depend on the phenotype variance explained by the genetic instrument, outcome sample size and case/control ratio. Minimum detectable odds ratios (OR) by the given genetic instruments and IBD samples can be observed in Table 1. In brief, most of the tested measures, 10 out of 13, showed enough power to detect moderate risk increases of 20% (minimum detectable OR < 1.20) (Table 1). The analyses with less power were for overall physical activity, which genetic instrument explained 0.08% of phenotype variance, with a minimum detectable OR of 2.27 for IBD risk.

Estimated effects of each exposure on IBD, CD and UC outcomes reflected by each genetic variant (Wald estimates: β_outcome_/β_exposure_) were combined in a single estimate per outcome through a likelihood-based MR approach^10^. This method assumes a linear relationship between the risk factor and outcome and a bivariate normal distribution for the genetic association estimates. Bonferroni multiple test correction was applied to consider evidence of exposure-to-IBD association (significance = 0.05/13: 0.0038). Heterogeneity between CD and UC risk effects was evaluated estimating the P value (P_disease het_) for the Q statistic for heterogeneity, assuming a fixed-effect model of 1 degree of freedom. This MR approach is considered the most accurate method when the assumption of linear relationship between the exposure and the outcome (common assumption for the other methods as well) is held^24^. However, it is sensitive to invalid instruments due to pleiotropic SNP effects (i.e. SNP effects on the outcome independent from the proxied exposure: horizontal pleiotropy).

Several complementary MR approaches were applied to detect the presence of horizontal pleiotropy, outliers (SNPs with strong pleiotropy), and unbalanced horizontal pleiotropy of weak instruments. The initial sensitivity test is called MR pleiotropy residual sum and outlier (MR-PRESSO) test^25^. This method identifies heterogeneity between SNP effects (P_Global_) as evidence of horizontal pleiotropy. Subsequently, it identifies outlier SNPs and provides evidence of biased effect on the initial risk estimates (P_Distortion_). Another complementary MR method is called MR-Egger test^26^. This method identifies the presence of non-balanced horizontal pleiotropy in weak instruments (i.e. SNP effects biased towards one direction) in the intercept test of the regression analyses between SNP-to-exposure effects and SNP-to-outcome effects. The violation of this assumption (non-balanced pleiotropy) is critical for all other MR methods. Finally, to test the consistency of the initial risk estimates, two methods that rely on the density distribution of individual SNP exposure-to-outcome effects were applied, namely weighted median MR method^27^ and the modal-based MR estimate approach^28^. These methods estimate the weighed median and the mode, respectively, of this density distribution, and are less sensitive to the presence of invalid SNPs (SNPs with mild pleiotropic effect).

To visualize the SNP exposure-to-outcome effects and the presence of pleiotropic SNPs, scatter plots were used to depict the SNP-to-exposure and SNP-to-IBD effects, including the likelihood-based MR risk estimate. Statistical analyses were performed using R (The R project; R version 3.4.1), the *TwoSampleMR* (version 0.4.18) and *MRPRESSO* (version 1.0) R packages, and plots were generated using *ggplot2* R package (version 3.1.0).

## Results

### Lifestyle exposures

**Figure 2:**
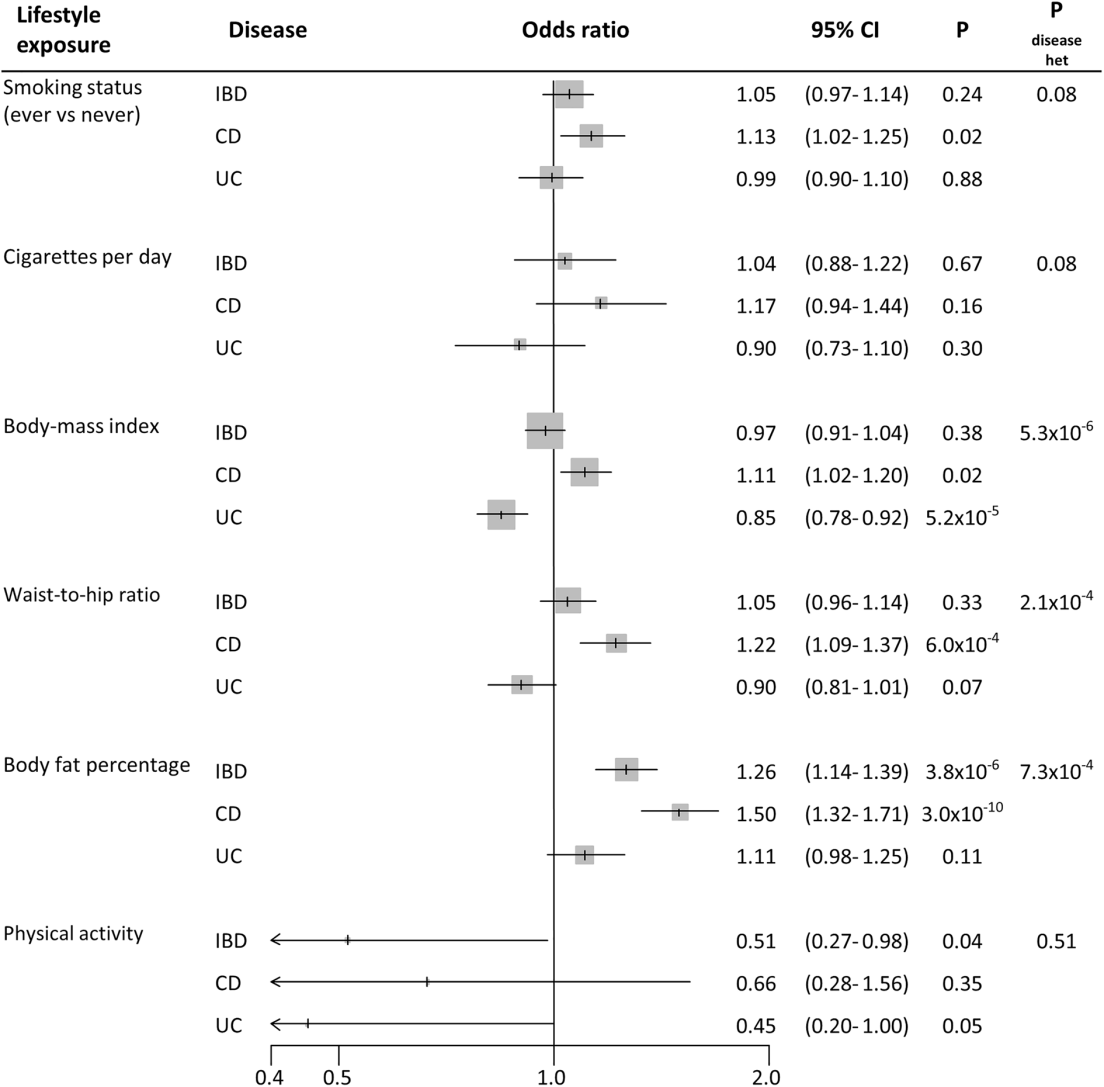
Forest plot of IBD risk for increment in lifestyle exposures. OR per standard deviation increase were estimated using likelihood-based MR approach. *95% CI* 95% confidence interval; *P* P value. *P*_*disease het*_ P value of heterogeneity between Crohn’s disease (CD) and ulcerative colitis (UC).

**Figure 3:**
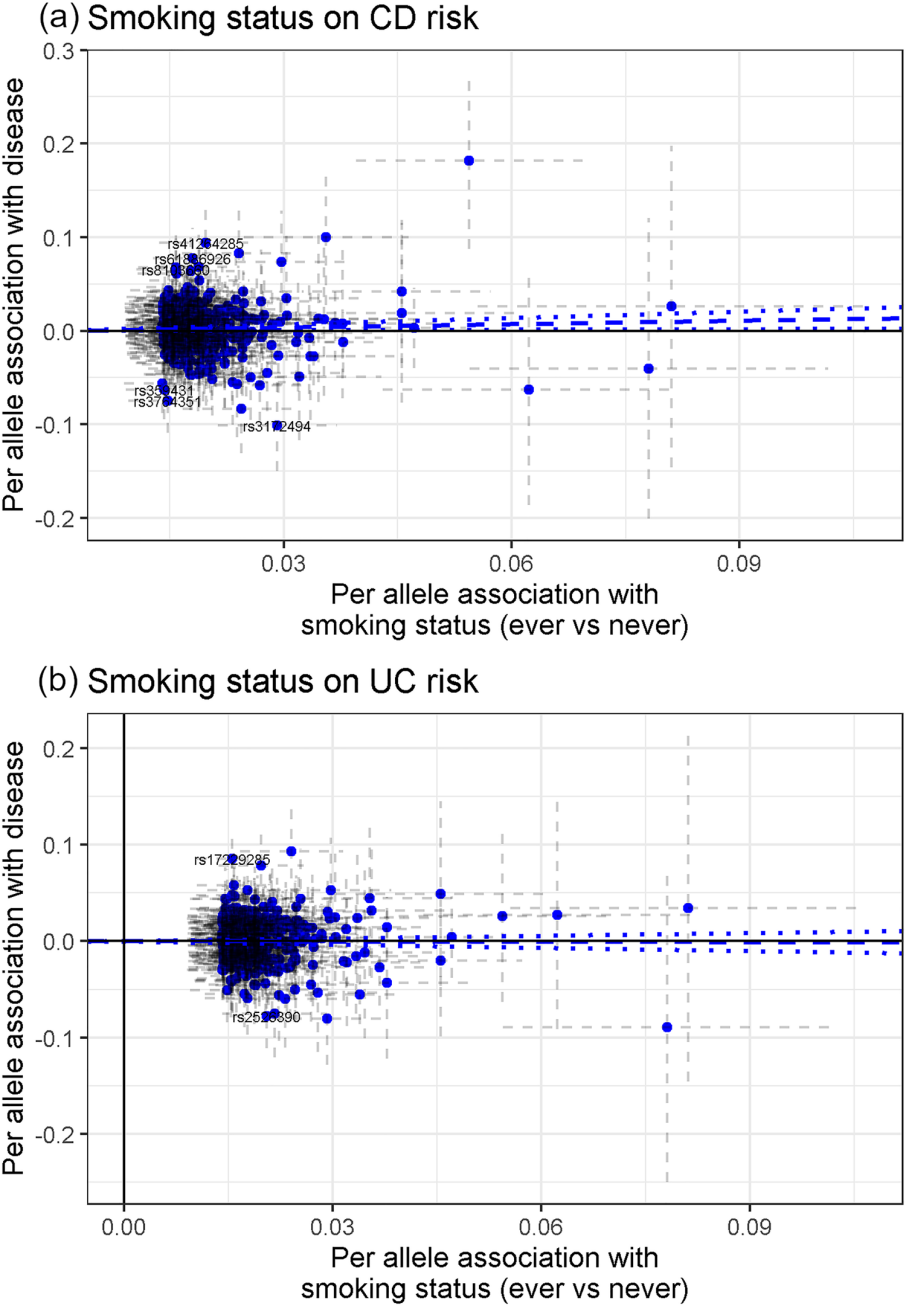
Scatter plots depicting summary statistics for genetic association and likelihood-based MR results of smoking status on CD (**a**), and smoking status on UC (**b**).

Tobacco smoking was not robustly related to IBD risk. Initially, the genetic instrument for risk of ever being smoker was associated with a mild 13% risk increase for CD (95% confidence interval (95% CI) 1.02–1.25); while it was not for UC risk (OR 0.99, 95% CI 0.90–1.10) (P_disease het_ = 0.08) (Fig. 2). Smoking intensity (number of cigarettes smoked per day) showed OR of 1.17 (95% CI 0.94–1.44, per SD of 1.1 extra cigarettes) and 0.90 (95% CI 0.73–1.10, per SD) for CD and UC, respectively (Fig. 2). In the sensitivity analyses, MR-PRESSO test detected horizontal pleiotropy (P_Global_ < 8.7 × 10^–3^) and potential outlier SNPs; however, the presence of outlier SNPs did not bias the initial risk estimates (P_Distortion_ > 0.19). MR Egger test detected evidence of directional pleiotropy for smoking intensity and CD risk (P_Egger intercept_ = 4 × 10^–3^), and provided an OR of 0.73 (95% CI 0.41–1.28, per SD). The other MR sensitivity approaches provided association estimates around the null for smoking status and smoking intensity on CD and UC risk (Supplementary Table S2 online). The scatter plots for the MR smoking status-to-CD association and smoking status-to-UC association are included in Fig. 3, while the other scatter plots can be observed in Supplementary Fig. S1 online.

**Figure 4:**
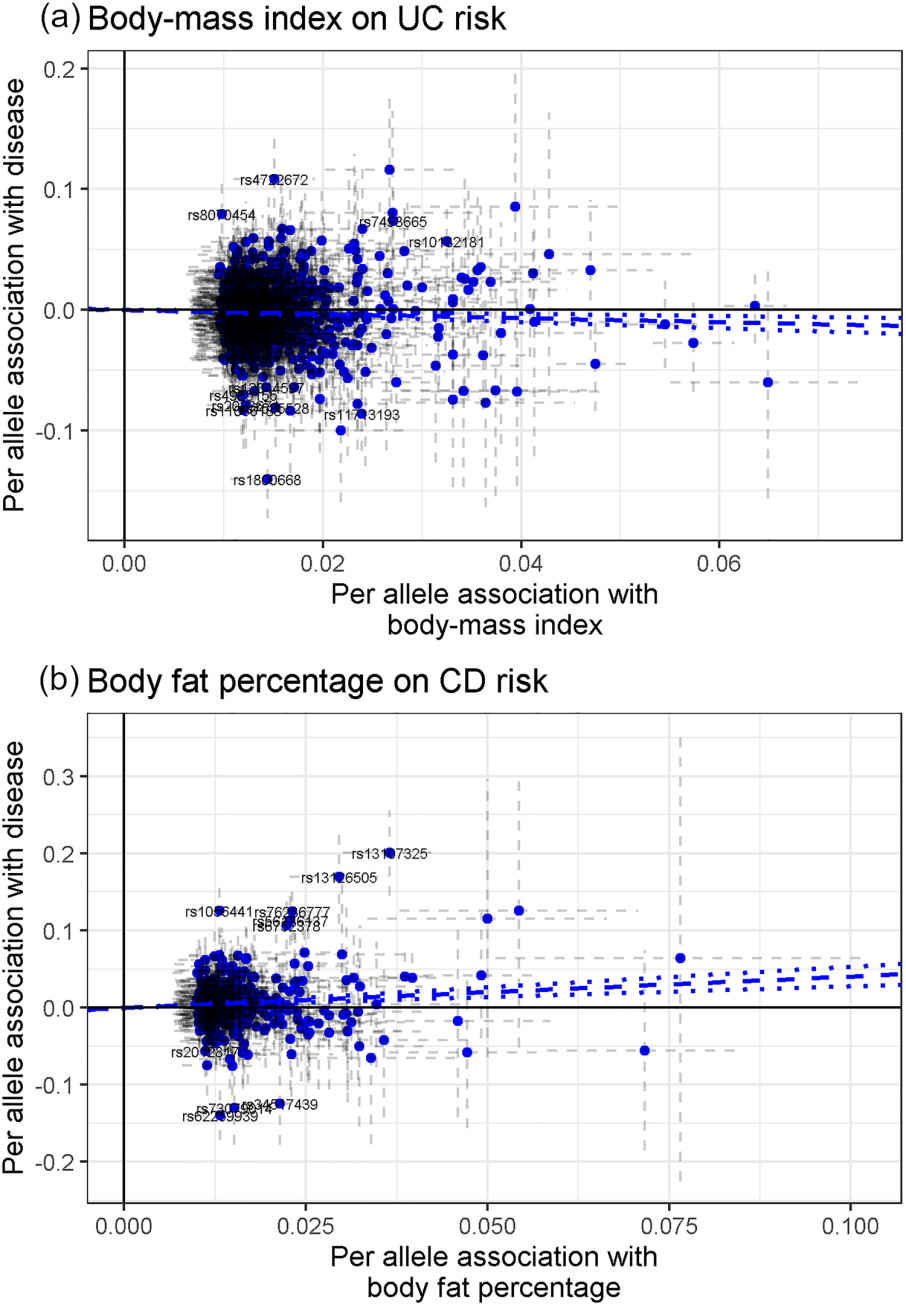
Scatter plots depicting summary statistics for genetic association and likelihood-based MR results of body-mass index on UC (**a**), and body fat percentage on CD (**b**).

We initially estimated that each SD increase in body-mass index (BMI; 4.6 kg/m^2^) and waist-to-hip ratio (0.1) were positively associated with CD risk (OR 1.11, 95% CI 1.02–1.20; and OR 1.22, 95% CI 1.09–1.37; respectively), but were inversely associated with UC risk (OR 0.85, 95% CI 0.78–0.92; and OR 0.90, 95% CI 0.81–1.01; respectively) (P_disease het_ < 2.1 × 10^–4^) (Fig. 2). On the contrary, each SD increase in body fat percentage (6.6%) was positively associated with CD risk (OR 1.50, 95% CI 1.32–1.71) and UC risk (OR 1.11, 95% CI 0.98–1.25) (P_disease het_ = 7.3 × 10^–4^) (Fig. 2). Horizontal pleiotropy and potential outliers were observed in all the cases, however without biasing the observed risk estimates (P_Global_ < 1 × 10^–4^, P_Distortion_ > 0.08) (Supplementary Table S2 online). Directional pleiotropy was not observed (P_Egger intercept_ > 0.02), and the other MR sensitivity approaches provided similar relative risk estimates (Supplementary Table S2 online). The scatter plots depicting the MR BMI-to-UC association and body fat percentage-to-CD association are included in Fig. 4. The other scatter plots can be observed in Supplementary Fig. S1 online.

Overall physical activity showed an inverse association with IBD risk (OR 0.51, 95% CI 0.27–0.98, per SD of 8.1 miligravities of acceleration), without heterogeneity between CD and UC (P_disease het_ = 0.51) (Fig. 2). However, SNP heterogeneity was observed for CD (P = 1.5 × 10^–3^), which was significant for a 2-SNP genetic instrument (Supplementary Table S2 online).

### Nutrient exposures

**Figure 5:**
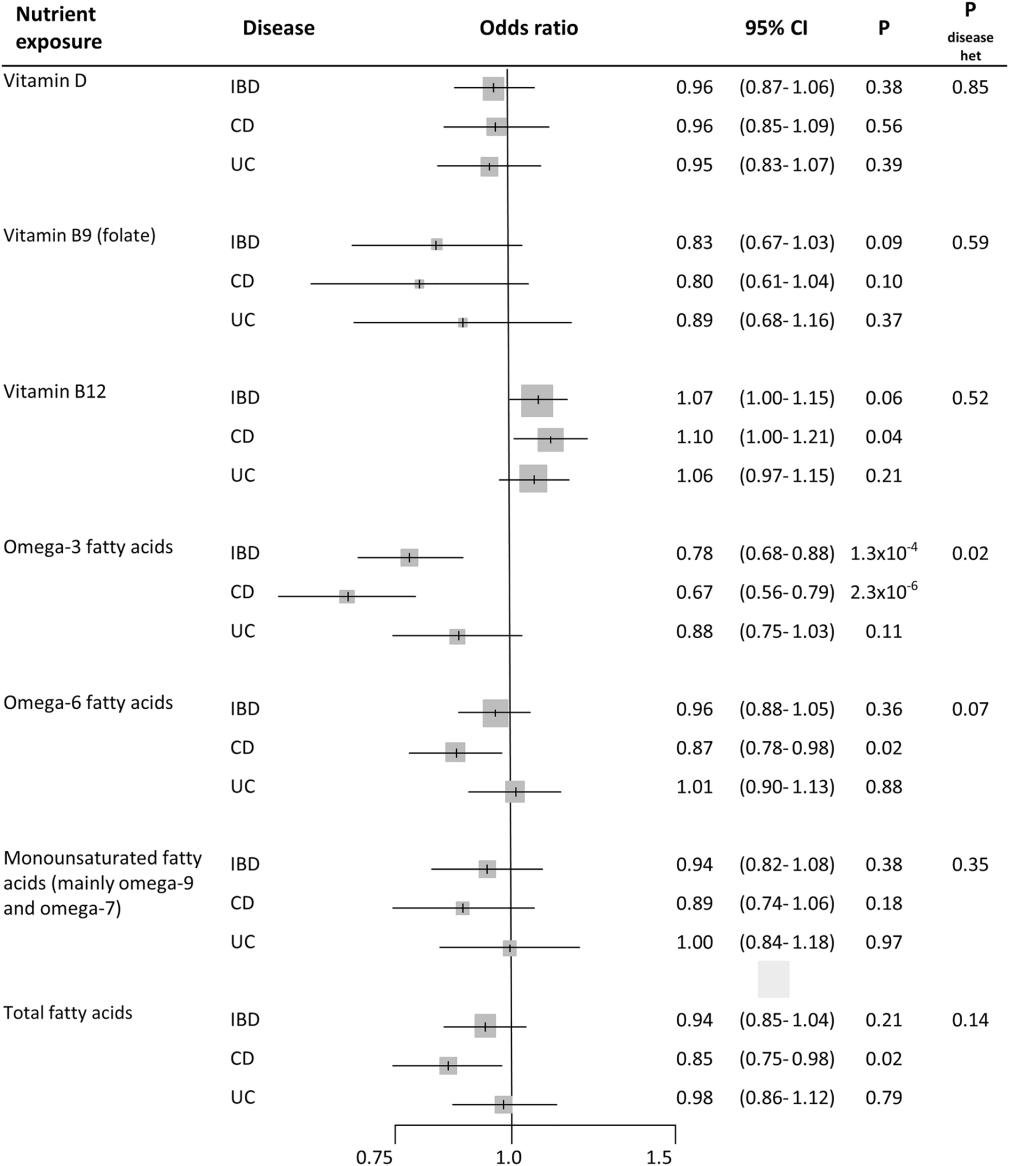
Forest plot of IBD risk for increment in nutrient exposures. OR per standard deviation increase were estimated using likelihood-based MR approach. 95% CI: 95% Confidence Interval; P: P value. P_disease het_: P value of heterogeneity between Crohn’s disease (CD) and ulcerative colitis (UC).

**Figure 6:**
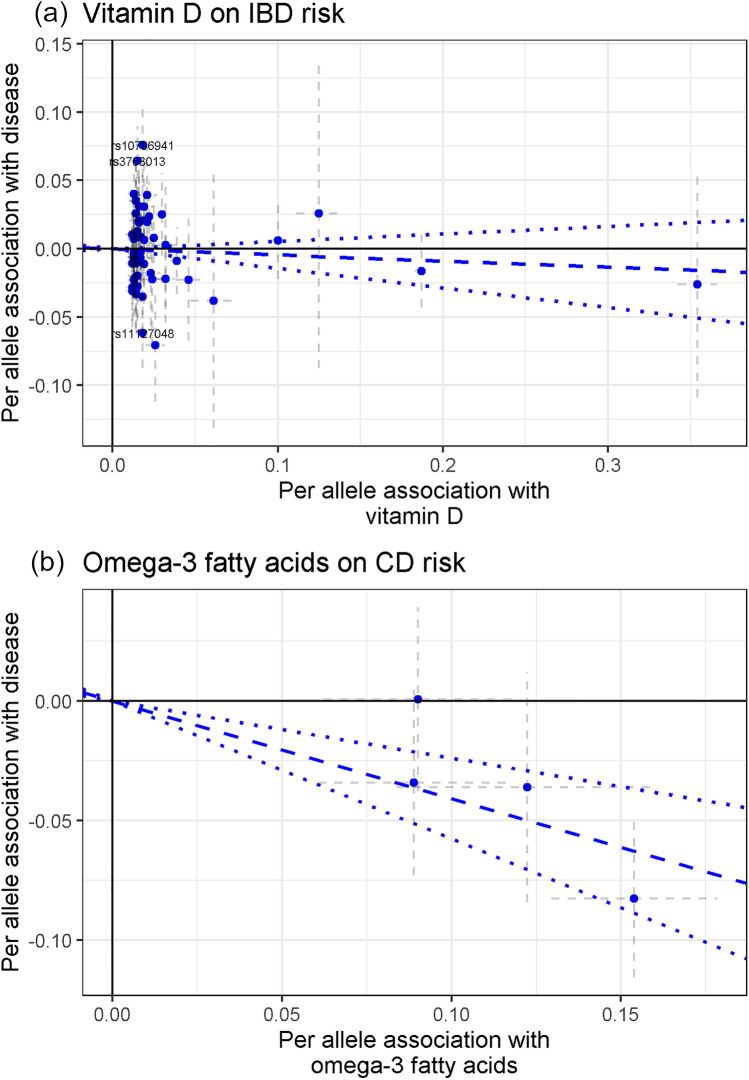
Scatter plots depicting summary statistics for genetic association and likelihood-based MR results of vitamin D on IBD (**a**), and omega-3 fatty acids on CD (**b**).

One SD increase in circulating vitamin D levels (34.7 nmol/l) showed null association with IBD risk (OR 0.96, 95% CI 0.87–1.06), without heterogeneity between CD and UC (P_disease het_ = 0.85) (Fig. 5). Horizontal pleiotropy and potential outliers were observed, however without biasing the observed risk estimates (P_Global_ < 1 × 10^–4^, P_Distortion_ > 0.77) (Supplementary Table S2 online). MR vitamin D-to-IBD scatter plot can be observed in Fig. 6a. Regarding B vitamins, one SD increase in circulating vitamin B9 levels (8.44 nmol/l) showed an OR of 0.83 (95% CI 0.67–1.03) for IBD (P_disease het_ = 0.59) (Fig. 5); however, SNP heterogeneity was observed for the 2-SNP instrument (P = 2.5 × 10^–3^; Supplementary Table S2 online). One SD increase in vitamin B12 levels (150.11 pmol/L) was initially associated with CD (OR 1.10, 95% CI 1.00–1.21) and UC (OR 1.06, 95% CI 0.97–1.15) (P_disease het_ = 0.52) (Fig. 5). However, MR-PRESSO test detected horizontal pleiotropy (P_Global_ < 1 × 10^–4^) and an outlier SNPs slightly biasing the initial risk estimate for CD (P_Distortion_ = 0.02), while the other sensitivity MR analyses provided null estimates for the vitamin B12-to-IBD and subtypes associations (OR from 0.92 to 1.09) (Supplementary Table S2 online). Scatter plots can be observed in Supplementary Fig. S1 online.

Finally, we observed that omega-3 fatty acid levels were inversely associated with IBD (OR 0.78, 95% CI 0.68–0.88, per SD of ~ 0.20 mmol/l). Some heterogeneity was observed between CD and UC (P_disease het_ = 0.02), with ORs of 0.67 (95% CI 0.56–0.79) and 0.88 (95% CI 0.75–1.03), respectively (Fig. 5). The other analyzed fatty acid measures did not show association with IBD (OR 0.96, 95% CI 0.88–1.05, for omega-6 fatty acids; OR 0.94, 95% CI 0.82–1.08, for monounsaturated fatty acids (mainly omega-7 and omega-9); and OR 0.94, 95% CI 0.85–1.04, for total fatty acids) (Fig. 5). Neither outlier SNPs, nor horizontal or directional pleiotropic effects were observed in these analyses. Additionally, the other MR methods provide similar relative risk estimates (Supplementary Table S2 online). The scatter plot for the MR omega-3 fatty acids-to-CD relationship is included in Fig. 6b, while the other scatter plots can be observed in Supplementary Fig. S1 online.

## Discussion

In this Mendelian randomization study, we found no evidence for a robust role of smoking on IBD and subtypes etiology. On the contrary, our results support an increased risk for higher body fat proportion and a protective role for higher levels of omega-3 fatty acids on CD etiology, as observed in large meta-analyses.

The most consistently reported potential risk factor for CD is tobacco smoking, showing risk increases higher than 50%. On the contrary, the role of smoking on UC has been observed as protective with similar strength^7^. Our MR study is powered enough to validate the described associations, but we only observed a potential relative risk increase of 13% for ever being smoker on CD risk; with inconclusive results in the MR sensitivity analyses. Therefore, the observed associations in traditional meta-analyses could be confounded by other factors associated with both smoking and IBD subtypes. Supporting this evidence, there are some studies describing a null association with other types of nicotine consumption (moist snuff)^29^ or in non-European populations^30^.

There is considerable evidence that links obesity and IBD risk; a considerable proportion of IBD patients are obese, the incidence of both obesity and IBD is increasing, and obesity strongly modifies gut microbiota^31,32^. Despite this, findings from cross-sectional and cohort studies are conflicting; cross-sectional studies showed an inverse association between BMI and IBD and subtypes^7^, while cohort and population-based studies have described a positive association between BMI and CD, but a negative trend between BMI and UC^31,33^. The present MR study is supporting the observations from cohort-based studies, and reveals a potential effect of reverse causation in the results from cross-sectional studies. Additionally, this opposing effect of BMI on CD and UC is similar to the reported pattern between being smoker and IBD subtypes in observational studies. Recently, a MR study analyzing obesity measures and smoking habits in more than 300,000 individuals identified higher levels of obesity as a factor increasing the risk for ever being a smoker and smoking at higher intensity^34^. Therefore, we hypothesize that the traditionally observed relation between smoking and IBD subtypes could be confounded by obesity, specially BMI. Finally, our MR results also confirmed that fat proportion would be more predictive for developing IBD, specially CD, than general obesity measures^31^.

Physical activity showed a robust inverse association with IBD and subtypes in large meta-analyses^7^. One limitation of the present MR study is the low statistical power for the overall physical activity genetic instrument (2 SNPs explaining the 0.08% of phenotype variance). Despite this, our MR estimate would be compatible with a protective effect of physical activity on IDB and subtypes risk.

Westernization of diet, characterized by high levels of saturated fat, red and processed meat instead of fish, and vitamin deficiency, is thought to be partially behind the rise in IBD incidence^35^. Both higher levels of D and B9 vitamins have been observed to reduce the IBD risk by 30% or more in large meta-analyses^7,36^. In a previous MR study on vitamin D, an association with IBD risk was not found; however this study included approximately 2,000 IBD cases^37^. Our well-powered MR study, including 25,042 IBD cases, would not be consistent with a protective effect for higher levels of vitamin D and folate on IBD and subtypes risk. Regarding fatty acids, omega-3 fatty acids have been associated with a reduced risk, while omega-6 fatty acids are considered a risk factor; however, there is no clear evidence of omega-3 therapeutic efficacy^38–41^. Our MR study provided evidence for a reduction of CD risk with higher levels of omega-3 fatty acids. The contribution of omega-3 reducing the CD risk would be mediated by their contribution to provide pro-resolving molecules in inflammatory processes^41^.

Despite limitations already mention, the main limitation of this study is the lack of analyses for putative risk factors related with living spaces, pre and post-natal, and birth conditions, surgeries, drugs, and microbiota risk factors, also observed in large meta-analyses^7^. For most of those factors, genetic instruments were not available because of the lack of genetic predisposition for those exposures. Recently, it has been assessed under a MR approach the contribution of microbial related traits (fecal propionate levels and butyrate gut production) on several metabolic parameters^42^. These genetic instruments did not reach our selection parameters, and we considered them unreliable. Studies with larger sample sizes are needed to identify microbial genetic instruments. Finally, another limitation of this MR study is the potential violation of MR assumptions regarding pleiotropic effects on the analyzed genetic instruments; however, MR sensitivity analyses and graphical scatter plots were used to avoid biases of this nature.

In conclusion, our MR results did not support a robust role of smoking on IBD and subtypes etiology; however, they were compatible with a risk effect for higher body fat proportion and a protective effect for higher levels of omega-3 fatty acids on CD etiology, observed in meta-analyses. These results provided a better estimation of the contribution of risk factors to IBD etiology, and supported the development of preventive disease strategies related to physical activity and diet.

## Supplementary information

Supplementary Information.

## Data Availability

The dataset needed to reproduce the results is found in the Supplementary Table S1 online. URL for GWAS on IBD and subtypes: ftp://ftp.sanger.ac.uk/pub/project/humgen/summary_statistics/human/2016-11-07/.
