# Supplementary material for: Identifying environmental risk factors for inflammatory bowel diseases: a Mendelian randomization study
Source: Sci Rep. 2020 Nov 6;10:19273. doi: 10.1038/s41598-020-76361-2 (PMC7648100; doi:10.1038/s41598-020-76361-2)
Supplement: Supplementary file 1 — Supplementary Information. [file 41598_2020_76361_MOESM1_ESM.pdf]

Original article:

# Identifying environmental risk factors for inflammatory bowel diseases: A Mendelian randomization study.

Robert Carreras-Torres[1,2,3,*], Gemma Ibáñez-Sanz[2,3], Mireia Obón-Santacana[1,2,3], Eric J. Duell[1,2], Victor Moreno[1,2,3,4].

*[1]Colorectal Cancer Group, ONCOBELL Program, Bellvitge Biomedical Research Institute (IDIBELL). Hospitalet de Llobregat, Barcelona, Spain.*

*[2]Unit of Biomarkers and Susceptibility,,Oncology Data Analytics Program, Catalan Institute of Oncology (ICO). Hospitalet de Llobregat, Barcelona, Spain.*

*[3]Consortium for Biomedical Research in Epidemiology and Public Health (CIBERESP), Spain.*

*[4]Department of Clinical Sciences, Faculty of Medicine, University of Barcelona, Barcelona, Spain.*

**Running Head:** Causality appraisal of risk factors for IBD risk

**Keywords:** IBD, Crohn's disease; ulcerative colitis, risk factors, two-sample Mendelian randomization

***Corresponding author:**
Robert Carreras-Torres,
Bellvitge Biomedical Research Institute,
Avinguda de la Granvia de l'Hospitalet, 199, 08908, L'Hospitalet de Llobregat, Barcelona, Spain.
Tel.: +34 932 603 786;
email: rcarrerastorres@gmail.com

**Figure S1** – Scatter plots of summary statistics for genetic association on potential risk exposures and inflammatory bowel diseases.

## Lifestyle exposure

**Smoking status (ever vs never smoker)** **Number of cigarettes smoked per day**

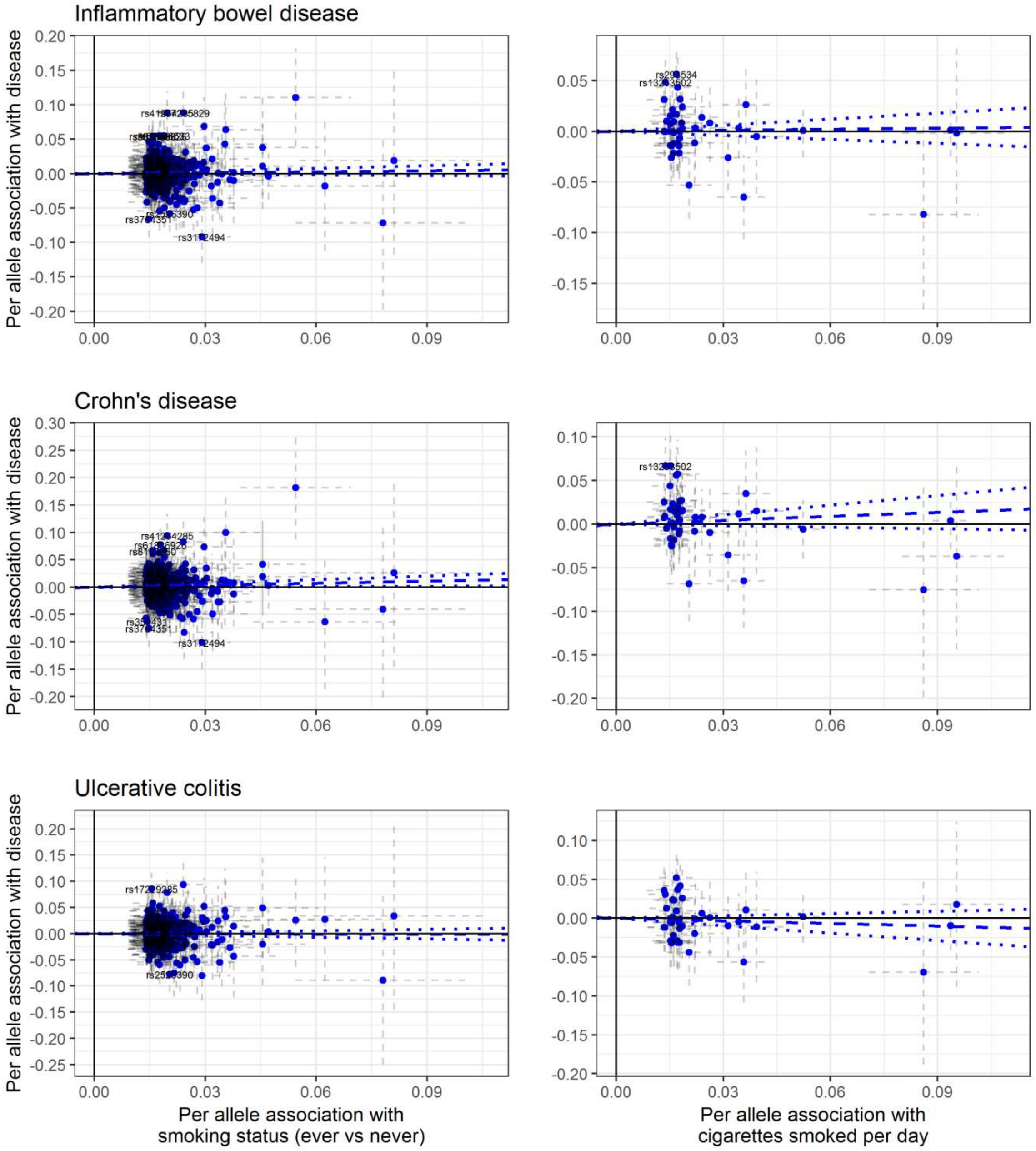

**Body-mass index** **Waist-to-hip ratio**

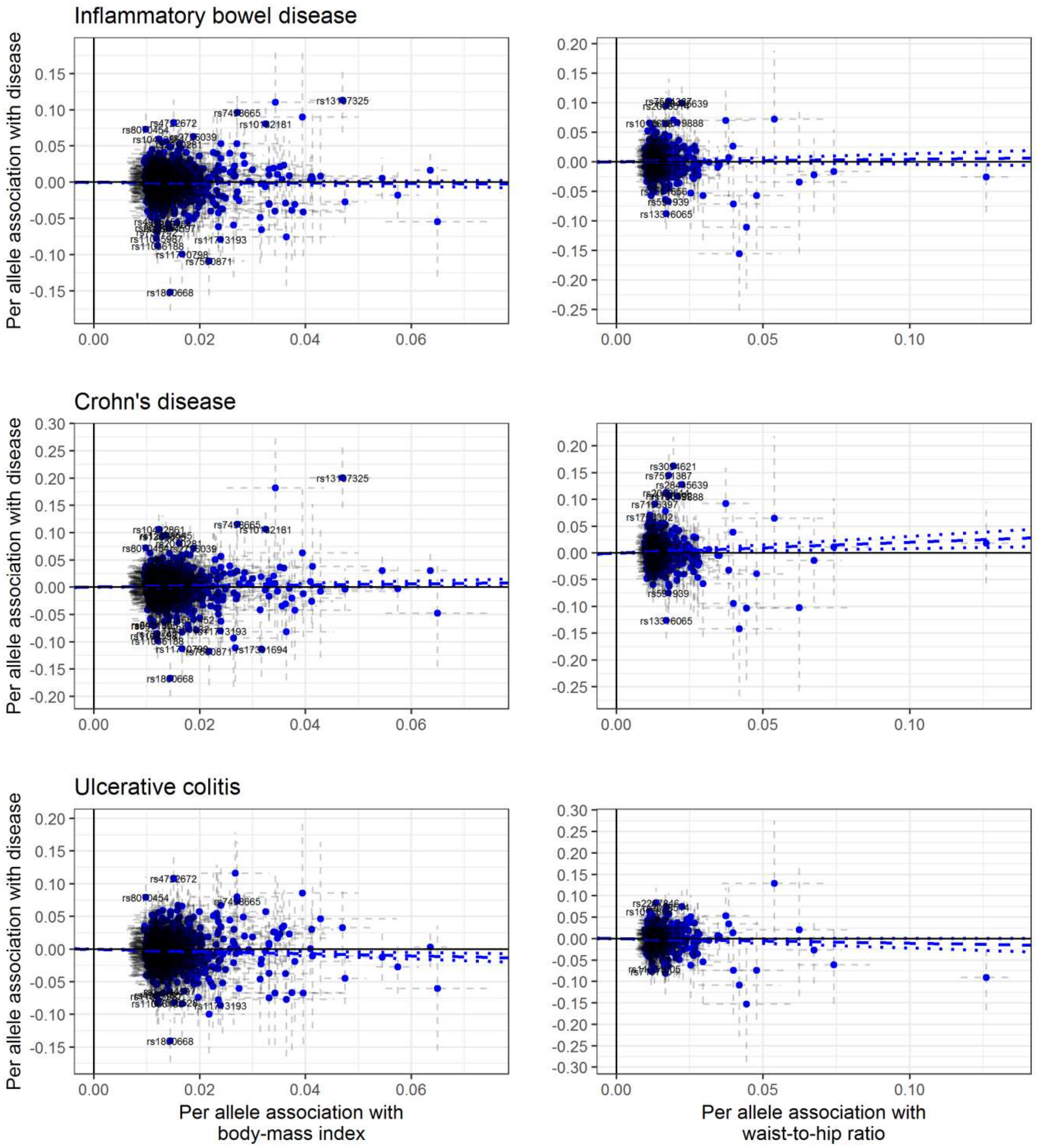

**Body fat percentage** | **Physical activity**

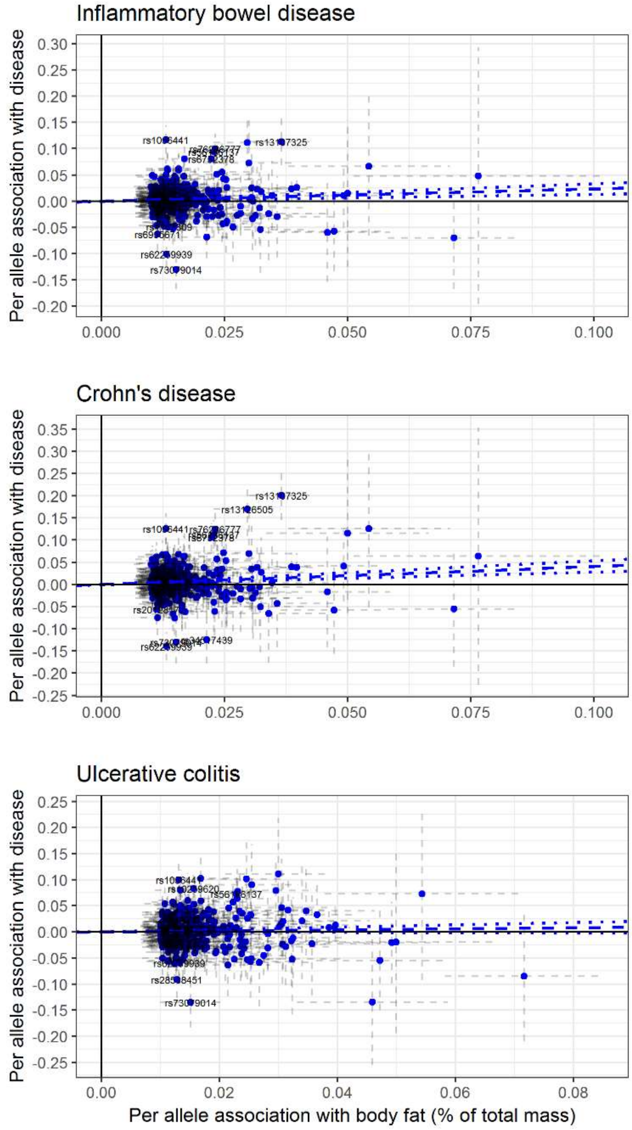


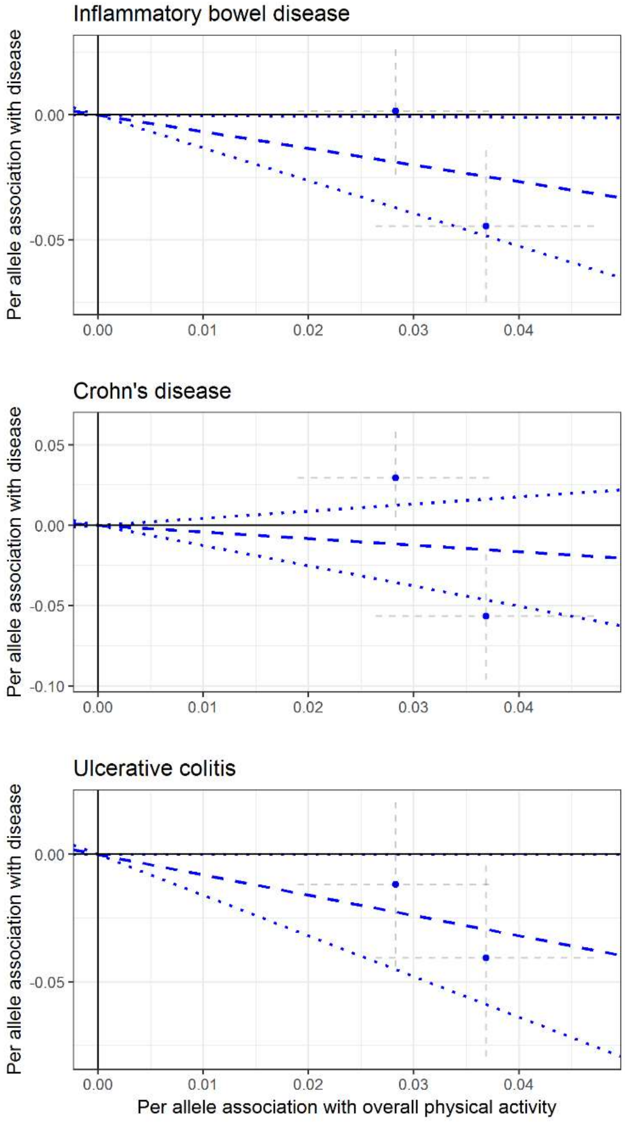

# Nutrient exposures

**Vitamin D** **Vitamin B9**

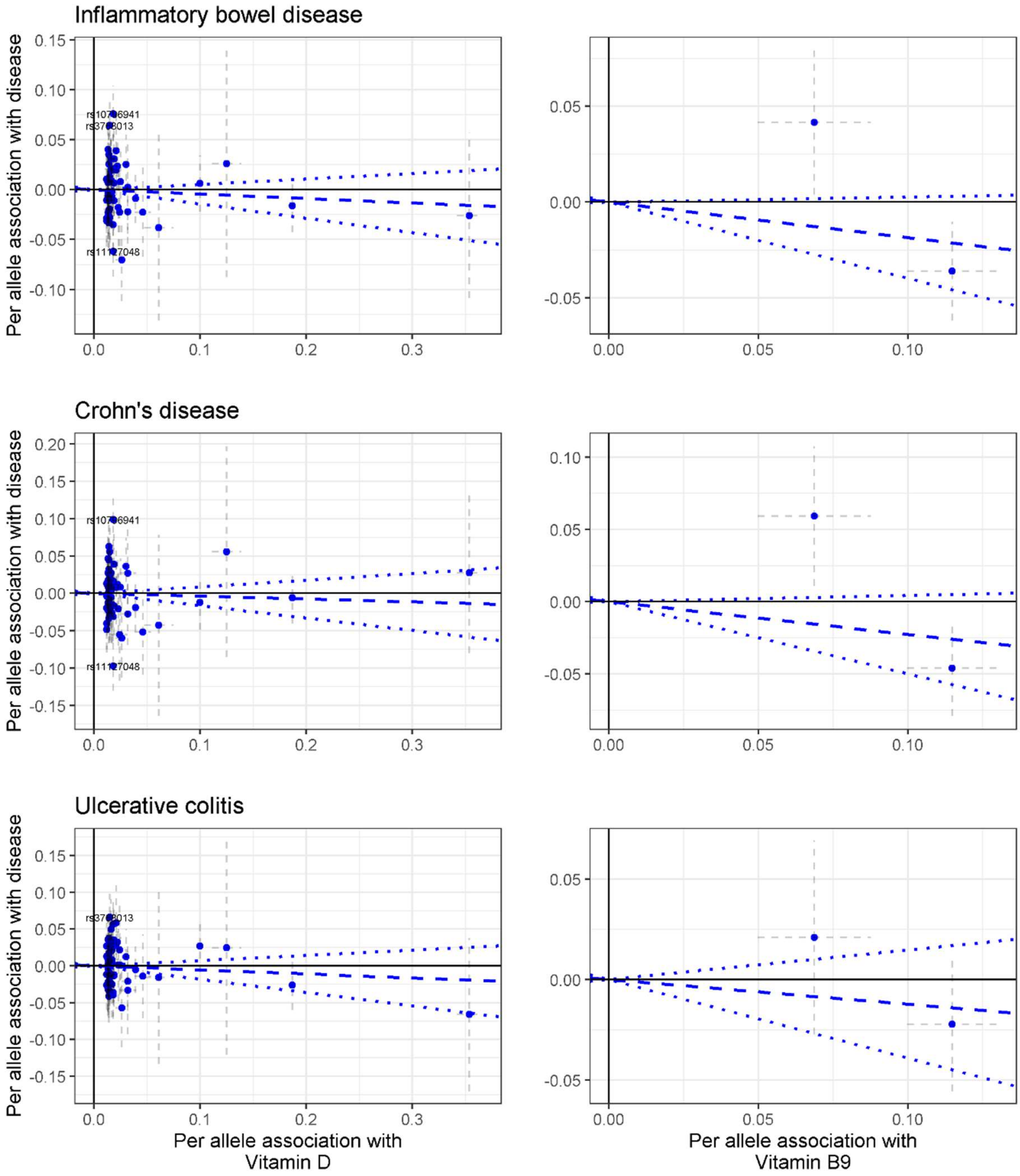

**Vitamin B12**

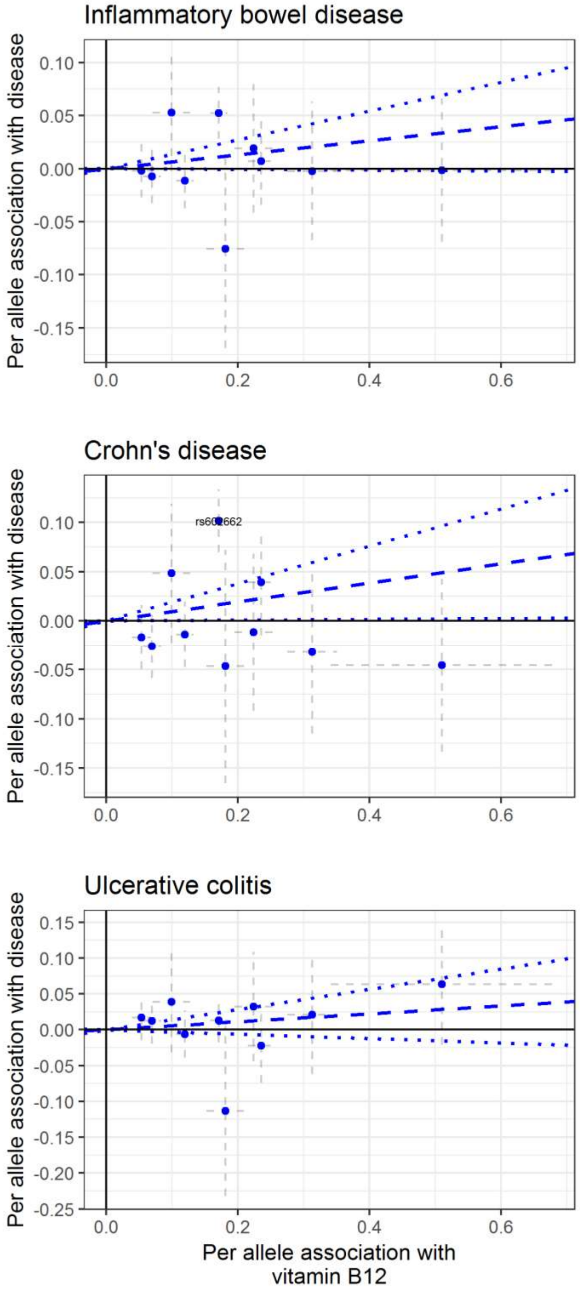

Inflammatory bowel disease
Crohn's disease
Ulcerative colitis
Per allele association with disease
Per allele association with vitamin B12
0.0
0.2
0.4
0.6

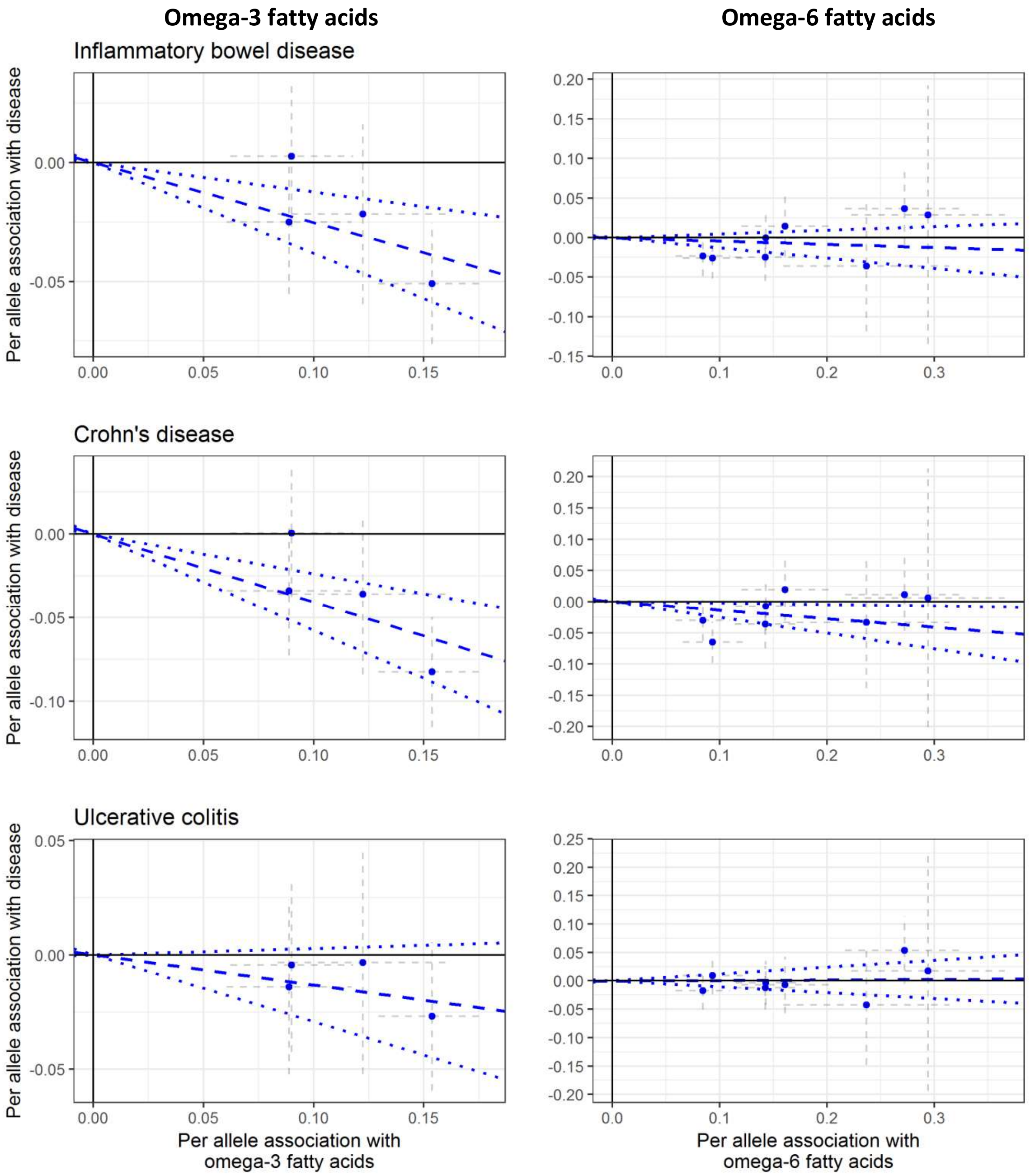

Omega-3 fatty acids
Omega-6 fatty acids
Inflammatory bowel disease
Crohn's disease
Ulcerative colitis
Per allele association with disease
Per allele association with omega-3 fatty acids
Per allele association with omega-6 fatty acids
0.00
0.05
0.10
0.15
-0.05
-0.10
0.0
0.1
0.2
0.3
0.20
0.25
-0.15
-0.20

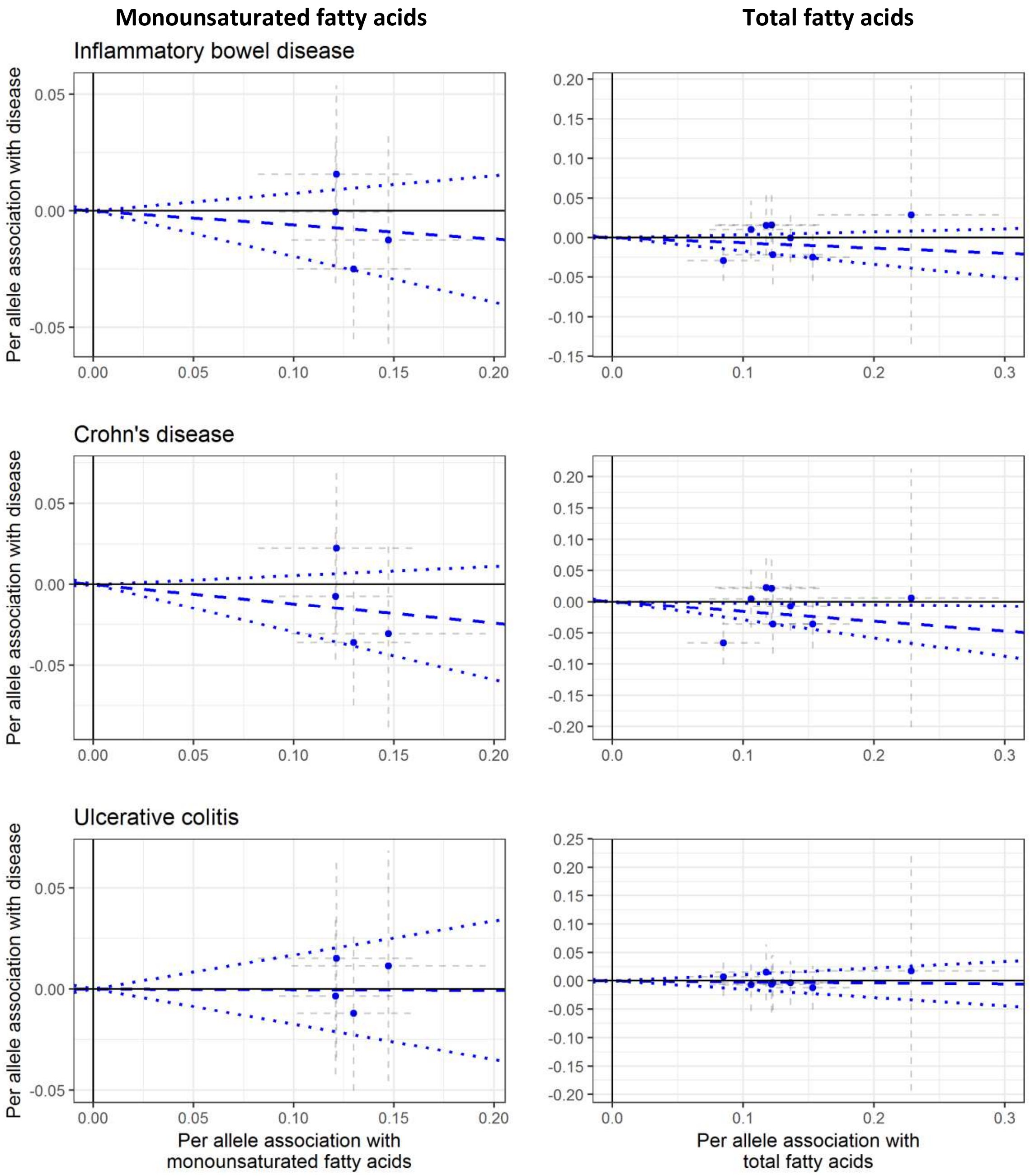

Monounsaturated fatty acids
Total fatty acids
Inflammatory bowel disease
Crohn's disease
Ulcerative colitis
Per allele association with disease
Per allele association with monounsaturated fatty acids
Per allele association with total fatty acids

**Table S1- Association summary statistics of genetic instruments for observed environmental risk factors and inflammatory bowel diseases (IBD), including Crohn's disease (CD), and ulcerative colitis (UC). CHR: Chromosome. BP. Base pair position on hg19. SE: standard error.**

| | | | | | | Phenotype | | IBD | | CD | | UC | |
|---|---|---|---|---|---|---|---|---|---|---|---|---|---|
| Phenotype | SNP | CHR | BP | Incrasing trait/ Effect allele | Other allele | BETA | SE | BETA | SE | BETA | SE | BETA | SE |
| Smoking status (ever vs never) | rs12130857 | 1 | 7791461 | G | A | 0.018 | 0.003 | 0.005 | 0.013 | -0.007 | 0.017 | 0.023 | 0.017 |
| | rs301807 | 1 | 8484823 | G | A | 0.018 | 0.003 | 0.002 | 0.013 | 0.033 | 0.016 | -0.021 | 0.016 |
| | rs3820277 | 1 | 18436657 | G | T | 0.019 | 0.003 | -0.002 | 0.013 | 0.002 | 0.017 | -0.004 | 0.016 |
| | rs1889571 | 1 | 32195819 | G | T | 0.022 | 0.004 | -0.033 | 0.019 | -0.036 | 0.024 | -0.031 | 0.024 |
| | rs10914684 | 1 | 33795572 | G | A | 0.016 | 0.003 | 0.045 | 0.013 | 0.033 | 0.017 | 0.058 | 0.017 |
| | rs2637869 | 1 | 38757237 | A | G | 0.018 | 0.003 | 0.025 | 0.014 | 0.014 | 0.018 | 0.029 | 0.017 |
| | rs12755632 | 1 | 41776623 | A | G | 0.015 | 0.003 | -0.027 | 0.013 | -0.027 | 0.017 | -0.024 | 0.017 |
| | rs951740 | 1 | 44011737 | A | G | 0.030 | 0.003 | 0.016 | 0.013 | 0.010 | 0.017 | 0.024 | 0.016 |
| | rs925524 | 1 | 46496709 | G | A | 0.016 | 0.003 | -0.028 | 0.014 | -0.015 | 0.018 | -0.040 | 0.018 |
| | rs12022778 | 1 | 50603995 | C | A | 0.027 | 0.003 | -0.052 | 0.016 | -0.058 | 0.020 | -0.045 | 0.020 |
| | rs7553439 | 1 | 50861622 | C | T | 0.018 | 0.003 | -0.020 | 0.015 | -0.047 | 0.019 | 0.003 | 0.019 |
| | rs4912332 | 1 | 58815243 | T | C | 0.014 | 0.003 | 0.003 | 0.012 | -0.005 | 0.016 | 0.008 | 0.016 |
| | rs6690398 | 1 | 66447394 | A | G | 0.020 | 0.003 | -0.005 | 0.012 | -0.018 | 0.016 | 0.007 | 0.016 |
| | rs1022528 | 1 | 71490122 | A | G | 0.017 | 0.003 | -0.024 | 0.013 | -0.041 | 0.017 | -0.018 | 0.017 |
| | rs12740789 | 1 | 72752073 | G | A | 0.028 | 0.003 | 0.013 | 0.016 | 0.007 | 0.021 | 0.003 | 0.020 |
| | rs10789369 | 1 | 73824909 | A | G | 0.023 | 0.003 | 0.014 | 0.013 | 0.021 | 0.016 | 0.014 | 0.016 |
| | rs1514176 | 1 | 74991596 | G | A | 0.019 | 0.003 | -0.008 | 0.013 | -0.002 | 0.016 | -0.010 | 0.016 |
| | rs10873871 | 1 | 76689019 | G | A | 0.017 | 0.003 | -0.019 | 0.015 | -0.017 | 0.020 | -0.018 | 0.020 |
| | rs11162019 | 1 | 87913176 | C | T | 0.015 | 0.003 | -0.018 | 0.013 | 0.007 | 0.017 | -0.040 | 0.016 |
| | rs1008078 | 1 | 91189731 | T | C | 0.023 | 0.003 | 0.019 | 0.013 | 0.008 | 0.017 | 0.020 | 0.016 |
| | rs1935571 | 1 | 96414335 | T | G | 0.016 | 0.003 | 0.005 | 0.012 | -0.001 | 0.016 | 0.008 | 0.016 |
| | rs12027999 | 1 | 154206358 | T | C | 0.024 | 0.004 | -0.041 | 0.019 | -0.083 | 0.025 | -0.012 | 0.024 |
| | rs41264285 | 1 | 155033918 | T | C | 0.020 | 0.003 | 0.089 | 0.016 | 0.094 | 0.020 | 0.078 | 0.020 |
| | rs2901785 | 1 | 174104743 | G | A | 0.017 | 0.003 | -0.004 | 0.012 | -0.017 | 0.016 | 0.002 | 0.016 |
| | rs147052174 | 1 | 179783167 | T | G | 0.062 | 0.010 | -0.018 | 0.048 | -0.063 | 0.063 | 0.027 | 0.060 |
| | rs35656245 | 1 | 190957480 | A | G | 0.016 | 0.003 | 0.005 | 0.014 | -0.009 | 0.018 | 0.016 | 0.018 |
| | rs12739243 | 1 | 210302043 | T | C | 0.021 | 0.003 | 0.022 | 0.015 | -0.011 | 0.019 | 0.040 | 0.019 |
| | rs12563365 | 1 | 236872829 | A | G | 0.017 | 0.003 | 0.031 | 0.013 | 0.026 | 0.016 | 0.032 | 0.016 |
| | rs876793 | 1 | 237852083 | T | C | 0.018 | 0.003 | 0.000 | 0.013 | 0.002 | 0.017 | 0.007 | 0.017 |
| | rs62106258 | 2 | 417167 | T | C | 0.045 | 0.006 | 0.011 | 0.032 | 0.042 | 0.042 | -0.021 | 0.040 |
| | rs6731872 | 2 | 624205 | G | T | 0.032 | 0.003 | -0.018 | 0.016 | -0.012 | 0.021 | -0.021 | 0.021 |
| | rs1022376 | 2 | 22067213 | T | C | 0.015 | 0.003 | -0.004 | 0.013 | -0.003 | 0.016 | -0.003 | 0.016 |
| | rs61533748 | 2 | 22582968 | C | T | 0.017 | 0.003 | 0.004 | 0.013 | 0.015 | 0.017 | -0.006 | 0.016 |
| | rs72790288 | 2 | 29513404 | G | A | 0.046 | 0.008 | 0.038 | 0.038 | 0.019 | 0.049 | 0.049 | 0.049 |
| | rs2710634 | 2 | 32808804 | T | C | 0.018 | 0.003 | -0.018 | 0.013 | -0.010 | 0.016 | -0.029 | 0.016 |
| | rs62137126 | 2 | 44250149 | A | G | 0.024 | 0.004 | 0.018 | 0.019 | 0.034 | 0.025 | -0.005 | 0.024 |
| | rs1004787 | 2 | 45159091 | A | G | 0.028 | 0.003 | -0.003 | 0.013 | -0.016 | 0.016 | 0.007 | 0.016 |
| | rs10490159 | 2 | 51341259 | T | C | 0.017 | 0.003 | 0.026 | 0.013 | 0.031 | 0.016 | 0.018 | 0.016 |
| | rs17616642 | 2 | 59022210 | A | G | 0.017 | 0.003 | 0.004 | 0.014 | -0.020 | 0.019 | 0.028 | 0.018 |
| | rs6730325 | 2 | 59315828 | G | A | 0.015 | 0.003 | 0.005 | 0.013 | 0.011 | 0.016 | 0.005 | 0.016 |
| | rs2539706 | 2 | 59819545 | A | G | 0.016 | 0.003 | 0.009 | 0.012 | 0.016 | 0.016 | 0.001 | 0.016 |
| | rs1863161 | 2 | 60139524 | A | G | 0.015 | 0.003 | -0.002 | 0.013 | -0.001 | 0.016 | -0.013 | 0.016 |
| | rs359249 | 2 | 60480237 | G | A | 0.022 | 0.003 | -0.007 | 0.013 | -0.006 | 0.017 | -0.004 | 0.016 |

| | | | | | | | | | | | | |
|---|---|---|---|---|---|---|---|---|---|---|---|---|
| rs62180324 | 2 | 63416606 | G | A | 0.020 | 0.003 | -0.014 | 0.015 | -0.029 | 0.020 | -0.001 | 0.019 |
| rs6750107 | 2 | 80748807 | A | G | 0.015 | 0.003 | -0.007 | 0.013 | -0.003 | 0.017 | -0.016 | 0.016 |
| rs12714017 | 2 | 80999398 | C | T | 0.015 | 0.003 | -0.017 | 0.012 | -0.011 | 0.016 | -0.019 | 0.016 |
| rs56208390 | 2 | 83247997 | G | A | 0.022 | 0.004 | 0.010 | 0.019 | 0.013 | 0.025 | 0.011 | 0.024 |
| rs11692435 | 2 | 98275354 | A | G | 0.025 | 0.005 | 0.000 | 0.024 | -0.011 | 0.030 | 0.013 | 0.030 |
| rs13392222 | 2 | 100672408 | A | C | 0.023 | 0.004 | -0.019 | 0.018 | -0.008 | 0.024 | -0.028 | 0.023 |
| rs1901477 | 2 | 104126983 | G | A | 0.030 | 0.003 | 0.005 | 0.012 | 0.016 | 0.016 | 0.004 | 0.016 |
| rs11889814 | 2 | 104432494 | A | C | 0.021 | 0.004 | 0.017 | 0.019 | 0.018 | 0.024 | 0.024 | 0.024 |
| rs3811038 | 2 | 113240183 | C | T | 0.019 | 0.003 | 0.013 | 0.014 | 0.026 | 0.018 | -0.002 | 0.018 |
| rs75210106 | 2 | 113246436 | C | T | 0.019 | 0.003 | 0.023 | 0.017 | 0.025 | 0.021 | 0.023 | 0.021 |
| rs34399632 | 2 | 137571174 | G | A | 0.019 | 0.003 | -0.004 | 0.015 | -0.013 | 0.019 | 0.008 | 0.019 |
| rs74697736 | 2 | 145412271 | A | G | 0.022 | 0.003 | -0.005 | 0.014 | 0.018 | 0.018 | -0.019 | 0.018 |
| rs6756212 | 2 | 146140132 | C | T | 0.034 | 0.003 | -0.043 | 0.013 | -0.027 | 0.016 | -0.055 | 0.016 |
| rs16826827 | 2 | 147825689 | T | C | 0.022 | 0.004 | -0.045 | 0.019 | -0.034 | 0.025 | -0.056 | 0.024 |
| rs1445649 | 2 | 155682556 | C | T | 0.021 | 0.003 | 0.009 | 0.012 | -0.004 | 0.016 | 0.022 | 0.016 |
| rs1722666 | 2 | 161816880 | T | C | 0.016 | 0.003 | -0.030 | 0.014 | -0.014 | 0.018 | -0.039 | 0.018 |
| rs12474587 | 2 | 162802993 | T | G | 0.024 | 0.003 | -0.004 | 0.013 | 0.020 | 0.016 | -0.021 | 0.016 |
| rs357304 | 2 | 164862639 | C | T | 0.017 | 0.003 | -0.018 | 0.014 | 0.003 | 0.018 | -0.030 | 0.018 |
| rs13007361 | 2 | 166250244 | A | G | 0.018 | 0.003 | 0.012 | 0.015 | 0.012 | 0.020 | 0.016 | 0.020 |
| rs7600835 | 2 | 172521827 | G | A | 0.015 | 0.003 | -0.003 | 0.014 | 0.010 | 0.018 | -0.022 | 0.017 |
| rs6750529 | 2 | 182027603 | T | C | 0.020 | 0.003 | 0.013 | 0.014 | 0.034 | 0.018 | -0.011 | 0.018 |
| rs17229285 | 2 | 199523122 | C | T | 0.015 | 0.003 | 0.040 | 0.012 | 0.000 | 0.016 | 0.085 | 0.016 |
| rs3115418 | 2 | 200936399 | T | C | 0.014 | 0.003 | -0.022 | 0.012 | -0.013 | 0.016 | -0.030 | 0.016 |
| rs62193862 | 2 | 202843875 | A | G | 0.024 | 0.004 | -0.036 | 0.022 | -0.057 | 0.029 | -0.027 | 0.028 |
| rs4674916 | 2 | 225365635 | C | A | 0.018 | 0.003 | 0.022 | 0.013 | 0.043 | 0.017 | 0.002 | 0.017 |
| rs4674993 | 2 | 226332033 | A | G | 0.024 | 0.003 | 0.015 | 0.016 | 0.016 | 0.020 | 0.017 | 0.020 |
| rs11713899 | 3 | 2365026 | C | A | 0.019 | 0.003 | -0.023 | 0.016 | 0.003 | 0.021 | -0.045 | 0.021 |
| rs748832 | 3 | 16851202 | G | A | 0.017 | 0.003 | 0.017 | 0.013 | 0.041 | 0.017 | -0.017 | 0.016 |
| rs10446419 | 3 | 25725501 | A | G | 0.020 | 0.003 | -0.006 | 0.016 | 0.005 | 0.021 | -0.010 | 0.020 |
| rs3172494 | 3 | 48731487 | G | T | 0.029 | 0.004 | -0.092 | 0.019 | -0.101 | 0.025 | -0.080 | 0.025 |
| rs2526390 | 3 | 50192760 | T | C | 0.020 | 0.003 | -0.058 | 0.013 | -0.040 | 0.017 | -0.078 | 0.017 |
| rs2276825 | 3 | 52886605 | C | T | 0.019 | 0.003 | -0.050 | 0.015 | -0.046 | 0.019 | -0.046 | 0.019 |
| rs73831818 | 3 | 55988394 | G | A | 0.032 | 0.005 | -0.036 | 0.027 | -0.049 | 0.035 | -0.022 | 0.034 |
| rs1910236 | 3 | 59434420 | A | G | 0.015 | 0.003 | 0.001 | 0.013 | -0.019 | 0.017 | 0.012 | 0.016 |
| rs7640107 | 3 | 59966156 | C | T | 0.014 | 0.003 | 0.020 | 0.013 | 0.012 | 0.016 | 0.026 | 0.016 |
| rs2734390 | 3 | 60459291 | G | A | 0.015 | 0.003 | 0.016 | 0.013 | 0.031 | 0.017 | 0.008 | 0.016 |
| rs221988 | 3 | 64234307 | A | C | 0.015 | 0.003 | -0.015 | 0.013 | -0.014 | 0.017 | -0.015 | 0.017 |
| rs6776440 | 3 | 70888609 | A | G | 0.019 | 0.003 | -0.007 | 0.014 | -0.021 | 0.018 | 0.005 | 0.017 |
| rs62246017 | 3 | 71483084 | G | A | 0.016 | 0.003 | 0.021 | 0.013 | 0.002 | 0.017 | 0.034 | 0.017 |
| rs57876564 | 3 | 75000248 | T | G | 0.022 | 0.003 | -0.024 | 0.016 | -0.024 | 0.020 | -0.024 | 0.020 |
| rs6782116 | 3 | 77176032 | C | T | 0.015 | 0.003 | 0.008 | 0.013 | -0.001 | 0.016 | 0.007 | 0.016 |
| rs13066050 | 3 | 81325861 | T | C | 0.019 | 0.003 | 0.006 | 0.015 | -0.014 | 0.020 | 0.019 | 0.020 |
| rs1549979 | 3 | 85460131 | C | T | 0.025 | 0.003 | -0.039 | 0.013 | -0.029 | 0.016 | -0.050 | 0.016 |
| rs57153235 | 3 | 85902536 | T | G | 0.019 | 0.003 | -0.032 | 0.013 | -0.041 | 0.017 | -0.030 | 0.017 |
| rs6437769 | 3 | 107997514 | T | C | 0.014 | 0.003 | 0.023 | 0.013 | 0.023 | 0.016 | 0.026 | 0.016 |
| rs9288999 | 3 | 114147927 | A | G | 0.017 | 0.003 | 0.016 | 0.014 | 0.033 | 0.019 | 0.014 | 0.018 |
| rs6438436 | 3 | 117822149 | T | C | 0.025 | 0.003 | 0.009 | 0.016 | 0.016 | 0.021 | 0.004 | 0.021 |
| rs12053870 | 3 | 118302515 | G | T | 0.016 | 0.003 | 0.002 | 0.013 | 0.009 | 0.016 | 0.001 | 0.016 |
| rs2279829 | 3 | 147106319 | C | T | 0.017 | 0.003 | 0.001 | 0.015 | 0.012 | 0.020 | -0.009 | 0.019 |
| rs2319545 | 3 | 147719648 | A | C | 0.023 | 0.004 | -0.004 | 0.018 | -0.055 | 0.023 | 0.017 | 0.022 |
| rs10935779 | 3 | 149543102 | C | T | 0.014 | 0.003 | -0.005 | 0.013 | -0.012 | 0.016 | 0.002 | 0.016 |

| | | | | | | | | | | | | |
|---|---|---|---|---|---|---|---|---|---|---|---|---|
| rs963354 | 3 | 157393770 | A | C | 0.015 | 0.003 | -0.006 | 0.013 | -0.029 | 0.017 | 0.014 | 0.017 |
| rs1714521 | 3 | 158284861 | A | C | 0.016 | 0.003 | -0.022 | 0.013 | -0.019 | 0.016 | -0.015 | 0.016 |
| rs1449012 | 3 | 159048333 | C | T | 0.015 | 0.003 | 0.007 | 0.013 | 0.014 | 0.016 | -0.003 | 0.016 |
| rs9850597 | 3 | 161761866 | G | A | 0.019 | 0.003 | -0.002 | 0.016 | 0.007 | 0.021 | -0.011 | 0.020 |
| rs1187820 | 3 | 173072584 | C | T | 0.014 | 0.003 | 0.001 | 0.013 | 0.009 | 0.017 | -0.011 | 0.016 |
| rs16828799 | 3 | 173353739 | T | G | 0.020 | 0.004 | -0.015 | 0.017 | -0.018 | 0.022 | 0.001 | 0.022 |
| rs9841807 | 3 | 175718927 | T | C | 0.016 | 0.003 | 0.027 | 0.014 | 0.035 | 0.018 | 0.024 | 0.018 |
| rs7631379 | 3 | 181409057 | C | T | 0.021 | 0.003 | 0.008 | 0.017 | 0.009 | 0.022 | 0.018 | 0.021 |
| rs973629 | 4 | 15463221 | A | G | 0.014 | 0.003 | -0.005 | 0.013 | 0.020 | 0.016 | -0.030 | 0.016 |
| rs12642744 | 4 | 28027176 | G | T | 0.017 | 0.003 | -0.005 | 0.014 | -0.006 | 0.018 | 0.000 | 0.018 |
| rs59537158 | 4 | 28246049 | T | C | 0.022 | 0.003 | 0.000 | 0.016 | 0.006 | 0.020 | 0.002 | 0.020 |
| rs73123371 | 4 | 28813881 | T | C | 0.017 | 0.003 | 0.013 | 0.014 | 0.027 | 0.019 | 0.003 | 0.018 |
| rs55944129 | 4 | 29082156 | T | C | 0.018 | 0.003 | -0.013 | 0.014 | -0.002 | 0.018 | -0.024 | 0.018 |
| rs58400863 | 4 | 31184484 | G | A | 0.020 | 0.003 | -0.013 | 0.013 | -0.020 | 0.017 | -0.004 | 0.017 |
| rs13142792 | 4 | 35512296 | C | T | 0.019 | 0.003 | -0.023 | 0.012 | -0.018 | 0.016 | -0.020 | 0.016 |
| rs112725451 | 4 | 68017710 | T | C | 0.026 | 0.003 | 0.008 | 0.016 | 0.012 | 0.021 | 0.005 | 0.021 |
| rs10021088 | 4 | 94059475 | C | T | 0.015 | 0.003 | -0.026 | 0.012 | -0.019 | 0.016 | -0.028 | 0.016 |
| rs1435479 | 4 | 94550450 | T | G | 0.016 | 0.003 | -0.003 | 0.014 | -0.005 | 0.018 | -0.004 | 0.017 |
| rs3934797 | 4 | 112467612 | G | A | 0.021 | 0.003 | -0.005 | 0.017 | 0.023 | 0.021 | -0.031 | 0.021 |
| rs71602617 | 4 | 136406155 | C | T | 0.018 | 0.003 | -0.005 | 0.015 | 0.000 | 0.020 | -0.003 | 0.019 |
| rs7696257 | 4 | 137474783 | A | G | 0.015 | 0.003 | 0.000 | 0.013 | 0.003 | 0.017 | 0.005 | 0.016 |
| rs13109980 | 4 | 140886963 | G | A | 0.022 | 0.003 | -0.013 | 0.013 | 0.016 | 0.017 | -0.029 | 0.017 |
| rs1116690 | 4 | 143510148 | G | A | 0.016 | 0.003 | 0.019 | 0.014 | 0.035 | 0.018 | 0.000 | 0.018 |
| rs13110073 | 4 | 147797913 | T | C | 0.025 | 0.003 | 0.002 | 0.013 | 0.001 | 0.016 | 0.001 | 0.016 |
| rs62334821 | 4 | 176865038 | A | G | 0.017 | 0.003 | -0.009 | 0.016 | -0.010 | 0.020 | -0.020 | 0.020 |
| rs12517438 | 5 | 30842054 | G | T | 0.015 | 0.003 | -0.005 | 0.012 | -0.008 | 0.016 | -0.005 | 0.016 |
| rs71627581 | 5 | 43161351 | G | A | 0.027 | 0.004 | 0.019 | 0.022 | 0.031 | 0.028 | 0.014 | 0.028 |
| rs10052591 | 5 | 50812738 | T | C | 0.016 | 0.003 | 0.009 | 0.013 | 0.021 | 0.016 | 0.009 | 0.016 |
| rs71592686 | 5 | 60121271 | C | T | 0.021 | 0.003 | -0.006 | 0.014 | 0.013 | 0.018 | -0.032 | 0.018 |
| rs2028269 | 5 | 79308315 | A | G | 0.016 | 0.003 | 0.012 | 0.013 | 0.007 | 0.016 | 0.016 | 0.016 |
| rs6874731 | 5 | 80263865 | G | T | 0.015 | 0.003 | -0.020 | 0.013 | -0.001 | 0.016 | -0.041 | 0.016 |
| rs10075489 | 5 | 88872383 | T | C | 0.018 | 0.003 | -0.004 | 0.014 | -0.007 | 0.018 | -0.013 | 0.017 |
| rs181508347 | 5 | 91366274 | G | T | 0.081 | 0.013 | 0.019 | 0.070 | 0.026 | 0.088 | 0.034 | 0.091 |
| rs42417 | 5 | 94198290 | T | C | 0.017 | 0.003 | 0.002 | 0.014 | -0.027 | 0.018 | 0.021 | 0.018 |
| rs72780746 | 5 | 103929588 | T | C | 0.026 | 0.003 | 0.017 | 0.016 | 0.013 | 0.021 | 0.017 | 0.021 |
| rs10060196 | 5 | 106455988 | A | C | 0.018 | 0.003 | -0.011 | 0.013 | -0.009 | 0.016 | -0.014 | 0.016 |
| rs17534089 | 5 | 106829219 | A | G | 0.026 | 0.004 | -0.026 | 0.018 | -0.050 | 0.023 | -0.001 | 0.023 |
| rs17165769 | 5 | 107365642 | G | A | 0.016 | 0.003 | 0.013 | 0.013 | 0.016 | 0.016 | 0.007 | 0.016 |
| rs329124 | 5 | 133865452 | A | G | 0.016 | 0.003 | -0.034 | 0.013 | -0.036 | 0.016 | -0.024 | 0.016 |
| rs1385108 | 5 | 154839646 | T | C | 0.019 | 0.003 | -0.001 | 0.014 | 0.000 | 0.019 | -0.010 | 0.018 |
| rs1173461 | 5 | 157707571 | T | C | 0.017 | 0.003 | 0.011 | 0.013 | 0.017 | 0.017 | -0.003 | 0.017 |
| rs3909281 | 5 | 165096435 | G | T | 0.021 | 0.003 | -0.012 | 0.013 | -0.028 | 0.017 | -0.003 | 0.016 |
| rs3843905 | 5 | 165427280 | C | T | 0.015 | 0.003 | 0.003 | 0.013 | 0.000 | 0.016 | 0.003 | 0.016 |
| rs79476395 | 5 | 166063680 | G | A | 0.033 | 0.005 | -0.012 | 0.024 | -0.008 | 0.031 | -0.016 | 0.030 |
| rs6890961 | 5 | 166778503 | C | T | 0.019 | 0.003 | 0.001 | 0.014 | -0.002 | 0.018 | 0.003 | 0.017 |
| rs4044321 | 5 | 166989513 | A | G | 0.023 | 0.003 | 0.021 | 0.013 | 0.027 | 0.017 | 0.016 | 0.016 |
| rs10042827 | 5 | 170299916 | C | T | 0.017 | 0.003 | -0.010 | 0.013 | -0.007 | 0.017 | -0.008 | 0.017 |
| rs359431 | 5 | 173288534 | C | T | 0.014 | 0.003 | -0.042 | 0.013 | -0.063 | 0.016 | -0.027 | 0.016 |
| rs1059490 | 6 | 26171250 | T | C | 0.019 | 0.003 | -0.004 | 0.013 | 0.005 | 0.017 | -0.003 | 0.016 |
| rs1150668 | 6 | 28129789 | T | G | 0.019 | 0.003 | -0.007 | 0.013 | -0.005 | 0.016 | -0.007 | 0.016 |
| rs1632941 | 6 | 29796685 | T | C | 0.016 | 0.003 | 0.049 | 0.014 | 0.067 | 0.018 | 0.034 | 0.018 |

| | | | | | | | | | | | | |
|---|---|---|---|---|---|---|---|---|---|---|---|---|
| rs3218116 | 6 | 41901763 | C | T | 0.020 | 0.003 | -0.011 | 0.014 | 0.001 | 0.018 | -0.022 | 0.018 |
| rs160631 | 6 | 52895230 | T | G | 0.017 | 0.003 | -0.027 | 0.014 | 0.009 | 0.018 | -0.055 | 0.018 |
| rs7743165 | 6 | 67521222 | G | T | 0.019 | 0.003 | 0.009 | 0.012 | 0.027 | 0.016 | -0.004 | 0.016 |
| rs10945141 | 6 | 69470709 | A | G | 0.018 | 0.003 | -0.004 | 0.014 | -0.003 | 0.018 | -0.003 | 0.018 |
| rs4707675 | 6 | 92221558 | G | A | 0.014 | 0.003 | 0.009 | 0.012 | 0.003 | 0.016 | 0.018 | 0.016 |
| rs619087 | 6 | 94175279 | G | A | 0.014 | 0.003 | -0.015 | 0.013 | -0.005 | 0.016 | -0.021 | 0.016 |
| rs6568832 | 6 | 97702876 | A | G | 0.019 | 0.003 | -0.005 | 0.014 | -0.035 | 0.019 | 0.019 | 0.018 |
| rs12195240 | 6 | 98636905 | A | G | 0.025 | 0.003 | 0.011 | 0.014 | 0.008 | 0.018 | 0.021 | 0.017 |
| rs6936160 | 6 | 100347745 | T | C | 0.020 | 0.003 | -0.017 | 0.014 | -0.027 | 0.018 | -0.006 | 0.017 |
| rs12530388 | 6 | 101329173 | A | C | 0.018 | 0.003 | -0.011 | 0.012 | -0.016 | 0.016 | -0.013 | 0.016 |
| rs3800227 | 6 | 108994161 | G | A | 0.017 | 0.003 | 0.010 | 0.014 | -0.002 | 0.018 | 0.026 | 0.018 |
| rs118202 | 6 | 111658371 | G | T | 0.037 | 0.003 | -0.009 | 0.016 | 0.008 | 0.021 | -0.027 | 0.020 |
| rs9331343 | 6 | 157738258 | T | C | 0.014 | 0.003 | -0.006 | 0.014 | -0.006 | 0.019 | -0.004 | 0.018 |
| rs10698713 | 6 | 158882320 | G | A | 0.034 | 0.006 | 0.000 | 0.027 | -0.027 | 0.035 | 0.024 | 0.035 |
| rs1764021 | 6 | 163805682 | C | T | 0.017 | 0.003 | 0.009 | 0.014 | 0.010 | 0.018 | 0.015 | 0.018 |
| rs10272990 | 7 | 1703675 | T | C | 0.021 | 0.003 | 0.022 | 0.013 | 0.016 | 0.017 | 0.035 | 0.017 |
| rs57257948 | 7 | 3330595 | C | T | 0.019 | 0.003 | 0.010 | 0.014 | -0.011 | 0.018 | 0.031 | 0.018 |
| rs7809303 | 7 | 69484366 | G | A | 0.021 | 0.003 | 0.004 | 0.013 | -0.006 | 0.017 | 0.017 | 0.017 |
| rs7802996 | 7 | 77771983 | C | T | 0.021 | 0.003 | -0.004 | 0.017 | -0.017 | 0.022 | 0.011 | 0.021 |
| rs1030015 | 7 | 78139581 | T | G | 0.014 | 0.003 | 0.011 | 0.012 | 0.036 | 0.016 | -0.016 | 0.016 |
| rs4727189 | 7 | 88442568 | C | T | 0.015 | 0.003 | 0.022 | 0.013 | 0.027 | 0.017 | 0.025 | 0.017 |
| rs12673563 | 7 | 91280470 | C | T | 0.023 | 0.004 | -0.040 | 0.021 | -0.022 | 0.027 | -0.060 | 0.026 |
| rs1799068 | 7 | 97707069 | T | G | 0.017 | 0.003 | -0.002 | 0.013 | 0.027 | 0.016 | -0.033 | 0.016 |
| rs13437771 | 7 | 99071478 | A | G | 0.027 | 0.004 | -0.015 | 0.017 | -0.007 | 0.022 | -0.025 | 0.022 |
| rs11766326 | 7 | 111100585 | T | C | 0.018 | 0.003 | -0.026 | 0.013 | -0.011 | 0.016 | -0.036 | 0.016 |
| rs6968380 | 7 | 114940159 | G | A | 0.023 | 0.003 | 0.005 | 0.014 | 0.018 | 0.018 | -0.001 | 0.017 |
| rs112913817 | 7 | 115077394 | G | A | 0.078 | 0.012 | -0.071 | 0.065 | -0.040 | 0.082 | -0.090 | 0.082 |
| rs10233018 | 7 | 117523709 | G | A | 0.025 | 0.003 | -0.009 | 0.012 | 0.016 | 0.016 | -0.020 | 0.016 |
| rs10953957 | 7 | 121954709 | A | G | 0.014 | 0.003 | -0.002 | 0.013 | 0.012 | 0.017 | -0.013 | 0.016 |
| rs77283305 | 7 | 132593831 | G | A | 0.015 | 0.003 | 0.013 | 0.013 | 0.003 | 0.017 | 0.020 | 0.017 |
| rs10279261 | 7 | 133589846 | G | A | 0.019 | 0.003 | 0.004 | 0.013 | 0.008 | 0.017 | 0.003 | 0.016 |
| rs1561112 | 7 | 133840652 | T | C | 0.015 | 0.003 | 0.008 | 0.013 | 0.013 | 0.017 | 0.012 | 0.016 |
| rs4840525 | 8 | 10765635 | C | A | 0.018 | 0.003 | 0.015 | 0.012 | 0.035 | 0.016 | -0.009 | 0.016 |
| rs11783093 | 8 | 27425349 | C | T | 0.047 | 0.003 | -0.004 | 0.017 | 0.003 | 0.022 | 0.004 | 0.022 |
| rs7836565 | 8 | 52569449 | C | T | 0.016 | 0.003 | 0.026 | 0.014 | 0.025 | 0.018 | 0.019 | 0.018 |
| rs13261666 | 8 | 59814666 | G | T | 0.020 | 0.003 | 0.034 | 0.012 | 0.037 | 0.016 | 0.035 | 0.016 |
| rs2063976 | 8 | 91096366 | C | T | 0.020 | 0.003 | -0.018 | 0.013 | -0.017 | 0.017 | -0.018 | 0.017 |
| rs6993429 | 8 | 92733282 | C | A | 0.019 | 0.003 | 0.013 | 0.012 | 0.012 | 0.016 | 0.003 | 0.016 |
| rs6986430 | 8 | 93048104 | T | C | 0.024 | 0.003 | 0.007 | 0.015 | -0.003 | 0.019 | 0.007 | 0.019 |
| rs9987376 | 8 | 93190014 | T | G | 0.020 | 0.003 | 0.014 | 0.013 | 0.008 | 0.016 | 0.020 | 0.016 |
| rs290601 | 8 | 115374642 | T | C | 0.016 | 0.003 | -0.022 | 0.014 | -0.019 | 0.018 | -0.023 | 0.018 |
| rs3847244 | 9 | 3025368 | T | C | 0.019 | 0.003 | 0.015 | 0.013 | 0.031 | 0.016 | 0.002 | 0.016 |
| rs11791671 | 9 | 3398679 | T | C | 0.028 | 0.005 | -0.049 | 0.025 | -0.045 | 0.032 | -0.054 | 0.031 |
| rs7024924 | 9 | 8282399 | C | T | 0.019 | 0.003 | 0.036 | 0.016 | 0.054 | 0.021 | 0.016 | 0.021 |
| rs2382252 | 9 | 10988832 | C | A | 0.016 | 0.003 | -0.007 | 0.013 | -0.004 | 0.016 | -0.008 | 0.016 |
| rs2078580 | 9 | 11159452 | A | G | 0.018 | 0.003 | -0.003 | 0.012 | 0.008 | 0.016 | -0.010 | 0.016 |
| rs7867822 | 9 | 20676454 | A | G | 0.015 | 0.003 | -0.020 | 0.013 | -0.024 | 0.017 | -0.018 | 0.017 |
| rs10966092 | 9 | 23831658 | T | C | 0.020 | 0.003 | -0.001 | 0.014 | -0.001 | 0.018 | -0.002 | 0.018 |
| rs988961 | 9 | 29746545 | G | A | 0.014 | 0.003 | 0.001 | 0.012 | -0.025 | 0.016 | 0.020 | 0.016 |
| rs4877285 | 9 | 81354129 | G | A | 0.018 | 0.003 | 0.004 | 0.014 | 0.031 | 0.017 | -0.022 | 0.017 |
| rs1930371 | 9 | 81444104 | C | T | 0.017 | 0.003 | 0.024 | 0.014 | 0.028 | 0.019 | 0.016 | 0.018 |

| | | | | | | | | | | | | |
|---|---|---|---|---|---|---|---|---|---|---|---|---|
| rs2378662 | 9 | 86707289 | A | G | 0.015 | 0.003 | -0.001 | 0.013 | 0.009 | 0.016 | -0.007 | 0.016 |
| rs1927901 | 9 | 120519111 | T | C | 0.014 | 0.003 | 0.009 | 0.012 | 0.021 | 0.016 | 0.001 | 0.016 |
| rs4837631 | 9 | 122061948 | C | T | 0.015 | 0.003 | 0.003 | 0.012 | -0.003 | 0.016 | 0.012 | 0.016 |
| rs1759433 | 9 | 128073097 | A | G | 0.015 | 0.003 | -0.003 | 0.013 | 0.008 | 0.016 | -0.015 | 0.016 |
| rs34553878 | 9 | 134334588 | G | A | 0.025 | 0.004 | 0.014 | 0.021 | 0.042 | 0.027 | -0.017 | 0.027 |
| rs7026534 | 9 | 134907263 | T | G | 0.017 | 0.003 | 0.020 | 0.014 | 0.029 | 0.018 | 0.014 | 0.017 |
| rs10905461 | 10 | 8803551 | T | C | 0.016 | 0.003 | -0.010 | 0.014 | -0.011 | 0.018 | -0.005 | 0.018 |
| rs10905649 | 10 | 10042641 | C | T | 0.016 | 0.003 | -0.031 | 0.012 | -0.035 | 0.016 | -0.023 | 0.016 |
| rs1291821 | 10 | 11133823 | G | A | 0.014 | 0.003 | 0.017 | 0.012 | -0.002 | 0.016 | 0.034 | 0.016 |
| rs11258417 | 10 | 13533053 | C | T | 0.015 | 0.003 | -0.006 | 0.013 | -0.023 | 0.016 | 0.014 | 0.016 |
| rs7072776 | 10 | 22032942 | A | G | 0.022 | 0.003 | 0.021 | 0.014 | 0.003 | 0.018 | 0.032 | 0.018 |
| rs2796793 | 10 | 36634124 | A | G | 0.014 | 0.003 | 0.013 | 0.012 | 0.019 | 0.016 | 0.022 | 0.016 |
| rs1733760 | 10 | 56698174 | C | T | 0.015 | 0.003 | 0.046 | 0.012 | 0.043 | 0.016 | 0.044 | 0.016 |
| rs7083526 | 10 | 63675843 | T | C | 0.023 | 0.003 | -0.010 | 0.013 | -0.008 | 0.016 | -0.009 | 0.016 |
| rs7901883 | 10 | 103186838 | G | A | 0.019 | 0.003 | 0.024 | 0.015 | 0.011 | 0.019 | 0.023 | 0.019 |
| rs11594623 | 10 | 103960351 | C | T | 0.027 | 0.003 | 0.011 | 0.015 | 0.017 | 0.019 | 0.002 | 0.018 |
| rs12244388 | 10 | 104640052 | A | G | 0.026 | 0.003 | -0.005 | 0.013 | -0.001 | 0.017 | 0.003 | 0.017 |
| rs34970111 | 10 | 106078937 | C | T | 0.015 | 0.003 | -0.001 | 0.013 | -0.004 | 0.017 | 0.007 | 0.016 |
| rs9787523 | 10 | 106460460 | T | C | 0.016 | 0.003 | 0.007 | 0.013 | 0.006 | 0.016 | 0.006 | 0.016 |
| rs11192347 | 10 | 106929313 | G | A | 0.026 | 0.004 | 0.016 | 0.020 | 0.012 | 0.026 | 0.014 | 0.026 |
| rs10885480 | 10 | 115378364 | T | C | 0.019 | 0.003 | 0.011 | 0.014 | 0.026 | 0.018 | -0.010 | 0.018 |
| rs4752018 | 10 | 118678712 | A | C | 0.019 | 0.003 | -0.036 | 0.015 | -0.043 | 0.019 | -0.034 | 0.019 |
| rs6265 | 11 | 27679916 | C | T | 0.029 | 0.003 | 0.008 | 0.016 | -0.026 | 0.020 | 0.030 | 0.020 |
| rs4275621 | 11 | 28652996 | A | G | 0.021 | 0.003 | -0.016 | 0.013 | -0.009 | 0.017 | -0.018 | 0.016 |
| rs62618693 | 11 | 32956492 | C | T | 0.035 | 0.006 | 0.043 | 0.032 | 0.012 | 0.042 | 0.044 | 0.041 |
| rs2939756 | 11 | 41436297 | G | A | 0.016 | 0.003 | 0.022 | 0.012 | 0.023 | 0.016 | 0.021 | 0.016 |
| rs1381775 | 11 | 42442826 | T | C | 0.016 | 0.003 | -0.011 | 0.014 | 0.003 | 0.018 | -0.020 | 0.018 |
| rs2959084 | 11 | 46078656 | A | G | 0.017 | 0.003 | 0.014 | 0.014 | 0.027 | 0.018 | 0.000 | 0.017 |
| rs3740977 | 11 | 46393574 | C | T | 0.019 | 0.003 | 0.004 | 0.017 | 0.006 | 0.022 | 0.010 | 0.021 |
| rs61886926 | 11 | 64133552 | C | T | 0.018 | 0.003 | 0.055 | 0.013 | 0.077 | 0.017 | 0.031 | 0.016 |
| rs61884449 | 11 | 64485193 | T | C | 0.020 | 0.004 | 0.001 | 0.018 | 0.010 | 0.023 | -0.017 | 0.023 |
| rs644740 | 11 | 65561468 | C | T | 0.014 | 0.003 | 0.023 | 0.012 | 0.029 | 0.016 | 0.023 | 0.016 |
| rs7943721 | 11 | 73309393 | G | A | 0.021 | 0.003 | 0.004 | 0.018 | -0.006 | 0.023 | 0.017 | 0.023 |
| rs7929518 | 11 | 85980958 | G | A | 0.019 | 0.003 | -0.022 | 0.015 | -0.015 | 0.019 | -0.015 | 0.019 |
| rs586699 | 11 | 92289734 | G | A | 0.015 | 0.003 | -0.015 | 0.012 | -0.030 | 0.016 | 0.006 | 0.016 |
| rs2155646 | 11 | 112912811 | C | T | 0.038 | 0.003 | 0.002 | 0.013 | -0.012 | 0.016 | 0.015 | 0.016 |
| rs1713676 | 11 | 113660576 | A | G | 0.017 | 0.003 | 0.013 | 0.012 | 0.021 | 0.016 | 0.004 | 0.016 |
| rs238896 | 11 | 113994505 | G | A | 0.017 | 0.003 | 0.033 | 0.013 | 0.038 | 0.016 | 0.017 | 0.016 |
| rs540860 | 11 | 121530888 | G | A | 0.018 | 0.003 | -0.002 | 0.013 | 0.010 | 0.016 | -0.012 | 0.016 |
| rs1944689 | 11 | 121634334 | T | G | 0.018 | 0.003 | -0.054 | 0.015 | -0.047 | 0.019 | -0.059 | 0.019 |
| rs1834306 | 11 | 122023187 | A | G | 0.014 | 0.003 | 0.019 | 0.013 | -0.008 | 0.017 | 0.044 | 0.017 |
| rs1106363 | 11 | 131966264 | T | C | 0.017 | 0.003 | -0.007 | 0.013 | -0.032 | 0.017 | 0.011 | 0.017 |
| rs2010921 | 11 | 132098205 | A | G | 0.017 | 0.003 | -0.005 | 0.014 | 0.012 | 0.018 | -0.022 | 0.017 |
| rs11057005 | 12 | 16748721 | A | G | 0.016 | 0.003 | -0.019 | 0.013 | -0.034 | 0.016 | -0.018 | 0.016 |
| rs13906 | 12 | 49952394 | C | T | 0.025 | 0.004 | 0.005 | 0.020 | -0.019 | 0.026 | 0.018 | 0.025 |
| rs4759229 | 12 | 56474480 | G | A | 0.016 | 0.003 | -0.011 | 0.013 | -0.016 | 0.017 | -0.012 | 0.017 |
| rs7969559 | 12 | 69655167 | A | G | 0.017 | 0.003 | 0.025 | 0.014 | 0.042 | 0.018 | 0.016 | 0.017 |
| rs7134009 | 12 | 75263193 | T | C | 0.016 | 0.003 | 0.012 | 0.014 | 0.020 | 0.018 | 0.001 | 0.018 |
| rs77215829 | 12 | 112618346 | A | C | 0.024 | 0.004 | 0.089 | 0.020 | 0.083 | 0.025 | 0.093 | 0.025 |
| rs1109480 | 12 | 121083279 | G | A | 0.017 | 0.003 | -0.011 | 0.013 | 0.000 | 0.017 | -0.015 | 0.017 |
| rs11611651 | 12 | 133380790 | A | G | 0.027 | 0.005 | -0.001 | 0.023 | 0.012 | 0.029 | 0.000 | 0.029 |

| | | | | | | | | | | | | |
|---|---|---|---|---|---|---|---|---|---|---|---|---|
| rs17197663 | 13 | 38172867 | G | A | 0.022 | 0.004 | -0.034 | 0.018 | 0.000 | 0.024 | -0.075 | 0.023 |
| rs4264267 | 13 | 38359676 | T | C | 0.015 | 0.003 | 0.003 | 0.013 | 0.012 | 0.016 | -0.003 | 0.016 |
| rs61959481 | 13 | 55834929 | G | A | 0.020 | 0.003 | -0.005 | 0.016 | -0.004 | 0.020 | -0.016 | 0.020 |
| rs3098272 | 13 | 55931424 | A | C | 0.018 | 0.003 | 0.026 | 0.016 | 0.028 | 0.020 | 0.017 | 0.020 |
| rs9538162 | 13 | 59265043 | C | T | 0.017 | 0.003 | 0.014 | 0.013 | 0.032 | 0.016 | 0.001 | 0.016 |
| rs1413119 | 13 | 59339281 | C | T | 0.015 | 0.003 | 0.021 | 0.013 | 0.032 | 0.016 | 0.007 | 0.016 |
| rs56367474 | 13 | 59454139 | C | T | 0.017 | 0.003 | 0.042 | 0.014 | 0.046 | 0.018 | 0.032 | 0.018 |
| rs55786907 | 13 | 59871584 | G | A | 0.019 | 0.003 | -0.017 | 0.017 | -0.008 | 0.022 | -0.026 | 0.021 |
| rs4886207 | 13 | 60705792 | T | C | 0.016 | 0.003 | 0.010 | 0.013 | 0.016 | 0.017 | 0.008 | 0.016 |
| rs9540731 | 13 | 66949370 | C | T | 0.018 | 0.003 | 0.001 | 0.012 | 0.014 | 0.016 | -0.012 | 0.016 |
| rs9545155 | 13 | 80191873 | T | C | 0.016 | 0.003 | -0.011 | 0.012 | -0.024 | 0.016 | 0.002 | 0.016 |
| rs1772572 | 13 | 81191176 | C | A | 0.017 | 0.003 | 0.002 | 0.013 | -0.012 | 0.017 | 0.013 | 0.017 |
| rs75674569 | 13 | 96823724 | G | A | 0.025 | 0.004 | 0.015 | 0.021 | -0.006 | 0.026 | 0.044 | 0.026 |
| rs7333559 | 13 | 100546450 | G | A | 0.023 | 0.003 | 0.010 | 0.016 | -0.007 | 0.021 | 0.022 | 0.020 |
| rs12855717 | 13 | 101252635 | T | C | 0.016 | 0.003 | 0.019 | 0.013 | 0.001 | 0.016 | 0.032 | 0.016 |
| rs12878369 | 14 | 28346502 | A | C | 0.017 | 0.003 | 0.006 | 0.013 | -0.011 | 0.016 | 0.015 | 0.016 |
| rs9323328 | 14 | 58653514 | A | G | 0.014 | 0.003 | 0.011 | 0.013 | 0.012 | 0.016 | 0.013 | 0.016 |
| rs1811739 | 14 | 77529375 | A | G | 0.018 | 0.003 | -0.006 | 0.014 | -0.001 | 0.019 | -0.016 | 0.018 |
| rs8005334 | 14 | 79563654 | G | T | 0.017 | 0.003 | -0.003 | 0.013 | -0.018 | 0.016 | 0.001 | 0.016 |
| rs34940743 | 14 | 80102233 | G | A | 0.016 | 0.003 | 0.006 | 0.014 | -0.016 | 0.018 | 0.018 | 0.018 |
| rs2925128 | 14 | 98362355 | T | C | 0.017 | 0.003 | 0.011 | 0.013 | 0.004 | 0.016 | 0.011 | 0.016 |
| rs1381287 | 14 | 98597552 | T | C | 0.018 | 0.003 | -0.008 | 0.013 | -0.022 | 0.016 | 0.007 | 0.016 |
| rs55913542 | 14 | 99693843 | T | G | 0.019 | 0.003 | -0.002 | 0.016 | 0.000 | 0.021 | 0.018 | 0.021 |
| rs1435672 | 15 | 36399479 | C | T | 0.014 | 0.003 | 0.018 | 0.012 | 0.025 | 0.016 | 0.020 | 0.016 |
| rs281296 | 15 | 47685010 | A | G | 0.025 | 0.003 | 0.031 | 0.013 | 0.030 | 0.017 | 0.036 | 0.017 |
| rs56902655 | 15 | 63898709 | T | G | 0.022 | 0.004 | 0.017 | 0.018 | 0.023 | 0.023 | 0.007 | 0.023 |
| rs2289791 | 15 | 67476952 | G | T | 0.018 | 0.003 | 0.055 | 0.015 | 0.064 | 0.019 | 0.053 | 0.019 |
| rs60833441 | 15 | 74048768 | A | G | 0.014 | 0.003 | -0.006 | 0.012 | 0.020 | 0.016 | -0.029 | 0.016 |
| rs62007780 | 15 | 78025464 | G | T | 0.016 | 0.003 | 0.004 | 0.013 | 0.022 | 0.016 | -0.013 | 0.016 |
| rs55958435 | 15 | 96852638 | A | G | 0.018 | 0.003 | 0.030 | 0.015 | 0.041 | 0.019 | 0.022 | 0.019 |
| rs8027457 | 15 | 99204101 | C | T | 0.015 | 0.003 | 0.034 | 0.013 | 0.035 | 0.016 | 0.033 | 0.016 |
| rs1139897 | 16 | 720986 | G | A | 0.024 | 0.003 | -0.008 | 0.015 | -0.003 | 0.019 | -0.007 | 0.019 |
| rs11076962 | 16 | 5811367 | C | T | 0.018 | 0.003 | -0.007 | 0.014 | -0.015 | 0.018 | -0.004 | 0.018 |
| rs7192140 | 16 | 10173748 | T | C | 0.017 | 0.003 | -0.023 | 0.012 | -0.027 | 0.016 | -0.020 | 0.016 |
| rs9922607 | 16 | 17570220 | C | T | 0.022 | 0.003 | 0.011 | 0.016 | -0.015 | 0.020 | 0.023 | 0.020 |
| rs7188873 | 16 | 24727064 | G | A | 0.020 | 0.003 | 0.031 | 0.013 | 0.041 | 0.017 | 0.025 | 0.016 |
| rs6497840 | 16 | 25351633 | A | G | 0.023 | 0.003 | -0.012 | 0.014 | -0.022 | 0.018 | 0.012 | 0.018 |
| rs4785187 | 16 | 49766772 | A | G | 0.020 | 0.003 | 0.018 | 0.015 | 0.019 | 0.020 | 0.020 | 0.019 |
| rs8050598 | 16 | 49891964 | T | C | 0.019 | 0.003 | -0.014 | 0.016 | -0.047 | 0.020 | 0.006 | 0.020 |
| rs12918191 | 16 | 50945156 | A | G | 0.020 | 0.003 | -0.027 | 0.015 | -0.044 | 0.019 | -0.007 | 0.019 |
| rs9302604 | 16 | 69576894 | G | A | 0.019 | 0.003 | 0.030 | 0.012 | 0.030 | 0.016 | 0.031 | 0.016 |
| rs9936784 | 16 | 72230694 | G | T | 0.014 | 0.003 | -0.027 | 0.012 | -0.056 | 0.016 | 0.002 | 0.016 |
| rs62052918 | 16 | 72578594 | T | G | 0.032 | 0.005 | 0.021 | 0.024 | -0.003 | 0.032 | 0.012 | 0.031 |
| rs4788676 | 16 | 72950468 | T | C | 0.018 | 0.003 | -0.027 | 0.015 | -0.032 | 0.019 | -0.028 | 0.019 |
| rs117657830 | 16 | 75766873 | A | G | 0.038 | 0.006 | -0.010 | 0.032 | 0.008 | 0.041 | -0.043 | 0.040 |
| rs1050847 | 16 | 87443734 | C | T | 0.015 | 0.003 | 0.001 | 0.013 | 0.008 | 0.016 | 0.010 | 0.016 |
| rs11642231 | 16 | 89608702 | G | A | 0.016 | 0.003 | 0.013 | 0.013 | 0.026 | 0.017 | 0.008 | 0.017 |
| rs4790874 | 17 | 1995177 | T | C | 0.017 | 0.003 | 0.007 | 0.013 | -0.005 | 0.016 | 0.013 | 0.016 |
| rs11078713 | 17 | 7795972 | A | G | 0.015 | 0.003 | 0.003 | 0.013 | 0.011 | 0.017 | -0.001 | 0.016 |
| rs28441558 | 17 | 7803118 | T | C | 0.036 | 0.006 | 0.064 | 0.028 | 0.100 | 0.037 | 0.032 | 0.036 |
| rs11651955 | 17 | 16235462 | G | A | 0.014 | 0.003 | -0.001 | 0.013 | 0.006 | 0.016 | 0.001 | 0.016 |

| | | | | | | | | | | | | | |
|---|---|---|---|---|---|---|---|---|---|---|---|---|---|
| | rs67777803 | 17 | 27323322 | G | T | 0.025 | 0.003 | 0.014 | 0.017 | -0.006 | 0.021 | 0.021 | 0.021 |
| | rs2344976 | 17 | 30685935 | T | C | 0.015 | 0.003 | 0.025 | 0.013 | 0.028 | 0.016 | 0.021 | 0.016 |
| | rs3764351 | 17 | 37824339 | G | A | 0.015 | 0.003 | -0.066 | 0.013 | -0.075 | 0.017 | -0.051 | 0.017 |
| | rs75919030 | 17 | 50193197 | T | C | 0.021 | 0.003 | -0.016 | 0.014 | -0.011 | 0.018 | -0.020 | 0.018 |
| | rs2938134 | 17 | 50243397 | C | A | 0.018 | 0.003 | 0.000 | 0.013 | 0.022 | 0.017 | -0.014 | 0.017 |
| | rs2587507 | 17 | 77790135 | T | C | 0.015 | 0.003 | -0.016 | 0.012 | -0.010 | 0.016 | -0.016 | 0.016 |
| | rs34342129 | 18 | 5872472 | T | C | 0.014 | 0.003 | -0.003 | 0.012 | 0.006 | 0.016 | -0.015 | 0.016 |
| | rs4476253 | 18 | 25253297 | G | A | 0.018 | 0.003 | 0.002 | 0.015 | 0.020 | 0.019 | -0.014 | 0.019 |
| | rs7505855 | 18 | 31696075 | C | T | 0.017 | 0.003 | -0.006 | 0.013 | 0.003 | 0.016 | 0.003 | 0.016 |
| | rs8096225 | 18 | 36921851 | C | A | 0.016 | 0.003 | -0.002 | 0.014 | 0.002 | 0.018 | -0.005 | 0.017 |
| | rs67050670 | 18 | 39297254 | A | G | 0.020 | 0.003 | 0.007 | 0.015 | -0.008 | 0.019 | 0.011 | 0.019 |
| | rs72898831 | 18 | 42658643 | A | G | 0.024 | 0.004 | 0.000 | 0.017 | -0.011 | 0.022 | 0.012 | 0.022 |
| | rs8083764 | 18 | 49874515 | G | T | 0.016 | 0.003 | -0.016 | 0.013 | -0.023 | 0.017 | -0.014 | 0.017 |
| | rs1373178 | 18 | 49967811 | T | G | 0.020 | 0.003 | -0.040 | 0.013 | -0.034 | 0.016 | -0.044 | 0.016 |
| | rs72938304 | 18 | 53661743 | G | A | 0.027 | 0.004 | -0.005 | 0.020 | -0.003 | 0.026 | -0.006 | 0.025 |
| | rs11872397 | 18 | 72535282 | G | A | 0.017 | 0.003 | 0.015 | 0.015 | 0.009 | 0.019 | 0.017 | 0.019 |
| | rs71367544 | 18 | 77574374 | T | C | 0.021 | 0.003 | -0.041 | 0.016 | -0.052 | 0.020 | -0.025 | 0.020 |
| | rs76608582 | 19 | 4474725 | C | A | 0.035 | 0.006 | -0.001 | 0.036 | 0.013 | 0.046 | -0.012 | 0.045 |
| | rs10853981 | 19 | 4965064 | A | G | 0.015 | 0.003 | -0.010 | 0.013 | -0.009 | 0.017 | 0.000 | 0.017 |
| | rs113230003 | 19 | 18460956 | G | A | 0.019 | 0.003 | 0.014 | 0.015 | 0.020 | 0.019 | 0.022 | 0.019 |
| | rs8103660 | 19 | 18566395 | C | T | 0.016 | 0.003 | 0.032 | 0.013 | 0.066 | 0.017 | -0.001 | 0.017 |
| | rs78081759 | 19 | 51130909 | A | G | 0.030 | 0.005 | 0.037 | 0.026 | 0.035 | 0.034 | 0.025 | 0.034 |
| | rs1126757 | 19 | 55879872 | T | C | 0.014 | 0.003 | -0.007 | 0.013 | -0.014 | 0.017 | -0.005 | 0.017 |
| | rs6050446 | 20 | 25195509 | G | A | 0.054 | 0.008 | 0.110 | 0.036 | 0.182 | 0.048 | 0.026 | 0.045 |
| | rs6058782 | 20 | 29946968 | T | C | 0.030 | 0.004 | 0.069 | 0.022 | 0.074 | 0.028 | 0.053 | 0.028 |
| | rs67696533 | 20 | 31173362 | A | G | 0.019 | 0.003 | 0.055 | 0.014 | 0.068 | 0.018 | 0.043 | 0.017 |
| | rs6103271 | 20 | 42015848 | A | G | 0.019 | 0.003 | 0.004 | 0.016 | 0.028 | 0.021 | -0.012 | 0.021 |
| | rs6011779 | 20 | 61984317 | C | T | 0.019 | 0.003 | 0.025 | 0.017 | 0.026 | 0.022 | 0.026 | 0.022 |
| | rs3810496 | 20 | 62406886 | C | T | 0.016 | 0.003 | 0.055 | 0.013 | 0.061 | 0.017 | 0.046 | 0.017 |
| | rs4818005 | 21 | 40588819 | G | A | 0.020 | 0.003 | 0.004 | 0.013 | 0.015 | 0.016 | -0.009 | 0.016 |
| | rs139896 | 22 | 38397797 | C | T | 0.015 | 0.003 | -0.022 | 0.013 | 0.006 | 0.017 | -0.042 | 0.016 |
| | rs4822102 | 22 | 42698430 | C | T | 0.017 | 0.003 | -0.024 | 0.013 | -0.022 | 0.017 | -0.024 | 0.016 |
| | rs9627290 | 22 | 46443040 | T | C | 0.015 | 0.003 | 0.035 | 0.013 | 0.019 | 0.018 | 0.047 | 0.017 |
| Cigarettes per day | rs11264100 | 1 | 35591626 | A | G | 0.022 | 0.004 | 0.003 | 0.020 | 0.008 | 0.026 | -0.001 | 0.025 |
| | rs7599488 | 2 | 60718347 | T | C | 0.014 | 0.002 | 0.010 | 0.013 | -0.005 | 0.016 | 0.013 | 0.016 |
| | rs78408772 | 2 | 62710608 | C | T | 0.022 | 0.004 | -0.011 | 0.021 | -0.008 | 0.027 | -0.020 | 0.026 |
| | rs10204824 | 2 | 148372720 | A | G | 0.018 | 0.003 | 0.031 | 0.013 | 0.027 | 0.017 | 0.042 | 0.017 |
| | rs2084533 | 3 | 16872929 | T | C | 0.016 | 0.003 | -0.002 | 0.013 | 0.015 | 0.017 | -0.030 | 0.017 |
| | rs7431710 | 3 | 48935583 | G | A | 0.018 | 0.003 | 0.008 | 0.013 | 0.027 | 0.017 | -0.010 | 0.016 |
| | rs2236951 | 3 | 50421081 | T | C | 0.017 | 0.003 | 0.043 | 0.016 | 0.057 | 0.020 | 0.037 | 0.020 |
| | rs699165 | 3 | 136224697 | G | A | 0.016 | 0.003 | -0.012 | 0.014 | -0.018 | 0.018 | 0.000 | 0.018 |
| | rs28813180 | 3 | 158083918 | G | A | 0.015 | 0.002 | -0.026 | 0.012 | -0.023 | 0.016 | -0.032 | 0.016 |
| | rs11940255 | 4 | 67086288 | G | A | 0.017 | 0.003 | -0.012 | 0.014 | 0.010 | 0.018 | -0.029 | 0.018 |
| | rs10454798 | 4 | 67980830 | T | G | 0.016 | 0.003 | 0.021 | 0.014 | 0.023 | 0.018 | 0.024 | 0.018 |
| | rs7766641 | 6 | 26184102 | G | A | 0.017 | 0.003 | -0.012 | 0.014 | 0.020 | 0.018 | -0.031 | 0.018 |
| | rs215600 | 7 | 32333642 | G | A | 0.024 | 0.003 | 0.014 | 0.013 | 0.008 | 0.017 | 0.006 | 0.016 |
| | rs62447179 | 7 | 50339609 | G | A | 0.015 | 0.003 | 0.015 | 0.014 | 0.067 | 0.018 | -0.029 | 0.017 |
| | rs2741351 | 8 | 27418040 | C | A | 0.018 | 0.003 | 0.024 | 0.017 | 0.015 | 0.021 | 0.026 | 0.021 |
| | rs73229090 | 8 | 27442127 | A | C | 0.026 | 0.004 | 0.008 | 0.020 | -0.010 | 0.026 | 0.001 | 0.025 |
| | rs13253502 | 8 | 42442018 | G | A | 0.014 | 0.002 | 0.048 | 0.013 | 0.066 | 0.017 | 0.031 | 0.016 |
| | rs4236926 | 8 | 42578059 | G | T | 0.034 | 0.003 | 0.003 | 0.015 | 0.012 | 0.019 | -0.005 | 0.019 |

| | | | | | | | | | | | | | |
|---|---|---|---|---|---|---|---|---|---|---|---|---|---|
| | rs790564 | 8 | 64604218 | A | C | 0.018 | 0.003 | -0.014 | 0.014 | -0.011 | 0.018 | -0.006 | 0.018 |
| | rs75596189 | 9 | 136468701 | T | C | 0.036 | 0.004 | -0.065 | 0.021 | -0.065 | 0.028 | -0.057 | 0.027 |
| | rs3025383 | 9 | 136502369 | T | C | 0.031 | 0.003 | -0.026 | 0.016 | -0.035 | 0.021 | -0.010 | 0.021 |
| | rs7951365 | 11 | 16377044 | C | T | 0.018 | 0.003 | 0.000 | 0.013 | 0.005 | 0.017 | -0.002 | 0.017 |
| | rs10742683 | 11 | 43667625 | G | A | 0.013 | 0.002 | 0.000 | 0.013 | 0.007 | 0.016 | -0.012 | 0.016 |
| | rs7125588 | 11 | 113436072 | A | G | 0.017 | 0.002 | 0.017 | 0.013 | 0.013 | 0.016 | 0.009 | 0.016 |
| | rs11846838 | 14 | 104184737 | A | G | 0.015 | 0.003 | 0.007 | 0.013 | 0.017 | 0.017 | -0.005 | 0.017 |
| | rs1115019 | 15 | 57141231 | T | C | 0.018 | 0.003 | -0.007 | 0.015 | 0.004 | 0.019 | -0.014 | 0.019 |
| | rs632811 | 15 | 59155050 | A | G | 0.018 | 0.003 | -0.021 | 0.013 | -0.008 | 0.017 | -0.031 | 0.017 |
| | rs10519203 | 15 | 78814046 | G | A | 0.094 | 0.003 | 0.001 | 0.013 | 0.004 | 0.017 | -0.009 | 0.017 |
| | rs182317 | 15 | 89943601 | G | T | 0.016 | 0.003 | -0.014 | 0.013 | -0.025 | 0.017 | -0.006 | 0.017 |
| | rs1592485 | 16 | 52093549 | C | A | 0.016 | 0.003 | 0.010 | 0.013 | -0.004 | 0.016 | 0.023 | 0.016 |
| | rs12924872 | 16 | 69552215 | C | T | 0.013 | 0.002 | 0.031 | 0.013 | 0.025 | 0.016 | 0.035 | 0.016 |
| | rs258321 | 16 | 89756473 | G | A | 0.016 | 0.002 | -0.001 | 0.013 | 0.001 | 0.016 | 0.001 | 0.016 |
| | rs4144686 | 18 | 53251725 | G | A | 0.019 | 0.003 | 0.003 | 0.017 | 0.016 | 0.022 | -0.010 | 0.021 |
| | rs4485470 | 18 | 62125063 | G | A | 0.015 | 0.002 | -0.014 | 0.013 | -0.016 | 0.017 | -0.004 | 0.016 |
| | rs7351050 | 19 | 4044579 | G | A | 0.020 | 0.003 | -0.053 | 0.017 | -0.069 | 0.022 | -0.044 | 0.022 |
| | rs145580088 | 19 | 41342842 | A | G | 0.086 | 0.008 | -0.082 | 0.048 | -0.075 | 0.063 | -0.069 | 0.061 |
| | rs56113850 | 19 | 41353107 | C | T | 0.052 | 0.002 | 0.001 | 0.013 | -0.006 | 0.017 | 0.002 | 0.017 |
| | rs8192726 | 19 | 41354496 | C | A | 0.039 | 0.005 | -0.005 | 0.029 | 0.015 | 0.037 | -0.011 | 0.036 |
| | rs117824460 | 19 | 41371480 | A | G | 0.095 | 0.008 | -0.002 | 0.043 | -0.037 | 0.055 | 0.018 | 0.054 |
| | rs6078373 | 20 | 11863500 | A | G | 0.016 | 0.002 | -0.021 | 0.013 | -0.019 | 0.016 | -0.022 | 0.016 |
| | rs293534 | 20 | 31065490 | G | A | 0.017 | 0.002 | 0.056 | 0.013 | 0.056 | 0.016 | 0.052 | 0.016 |
| | rs2273500 | 20 | 61986949 | C | T | 0.036 | 0.003 | 0.026 | 0.020 | 0.035 | 0.025 | 0.011 | 0.025 |
| | rs7281463 | 21 | 40520783 | C | A | 0.014 | 0.002 | -0.001 | 0.013 | 0.009 | 0.016 | -0.012 | 0.016 |
| Body-mass index | rs12044597 | 1 | 1708801 | G | A | 0.014 | 0.002 | -0.063 | 0.013 | -0.055 | 0.016 | -0.064 | 0.016 |
| | rs7535528 | 1 | 2444414 | G | A | 0.015 | 0.002 | -0.043 | 0.013 | 0.003 | 0.017 | -0.081 | 0.017 |
| | rs2235564 | 1 | 6713114 | T | C | 0.013 | 0.002 | -0.015 | 0.013 | -0.017 | 0.017 | -0.015 | 0.017 |
| | rs1891215 | 1 | 7727854 | C | T | 0.012 | 0.002 | -0.023 | 0.013 | -0.029 | 0.016 | -0.019 | 0.016 |
| | rs11121210 | 1 | 8708529 | C | T | 0.011 | 0.002 | -0.012 | 0.013 | 0.019 | 0.017 | -0.034 | 0.016 |
| | rs10779751 | 1 | 11284336 | A | G | 0.014 | 0.002 | -0.025 | 0.014 | -0.052 | 0.018 | -0.002 | 0.018 |
| | rs761423 | 1 | 17301672 | T | C | 0.011 | 0.002 | -0.004 | 0.013 | 0.015 | 0.016 | -0.020 | 0.016 |
| | rs3845520 | 1 | 23301943 | C | T | 0.019 | 0.002 | 0.014 | 0.016 | 0.018 | 0.021 | 0.013 | 0.021 |
| | rs3818788 | 1 | 32158374 | C | T | 0.013 | 0.002 | -0.012 | 0.013 | -0.008 | 0.017 | -0.019 | 0.017 |
| | rs12022461 | 1 | 33232525 | G | A | 0.016 | 0.002 | 0.019 | 0.016 | -0.005 | 0.021 | 0.040 | 0.021 |
| | rs4653017 | 1 | 33776728 | T | C | 0.012 | 0.002 | 0.041 | 0.013 | 0.024 | 0.017 | 0.056 | 0.017 |
| | rs9426003 | 1 | 34602870 | G | A | 0.012 | 0.002 | 0.022 | 0.014 | 0.009 | 0.018 | 0.031 | 0.018 |
| | rs11577094 | 1 | 38026600 | T | C | 0.018 | 0.003 | 0.031 | 0.023 | 0.059 | 0.030 | 0.004 | 0.029 |
| | rs2282231 | 1 | 39569571 | T | C | 0.017 | 0.002 | 0.010 | 0.015 | 0.040 | 0.020 | -0.022 | 0.020 |
| | rs11206436 | 1 | 40078447 | T | C | 0.017 | 0.003 | 0.021 | 0.020 | 0.021 | 0.026 | 0.018 | 0.026 |
| | rs946526 | 1 | 46487168 | C | T | 0.031 | 0.004 | -0.049 | 0.032 | -0.042 | 0.042 | -0.046 | 0.041 |
| | rs657452 | 1 | 49589847 | A | G | 0.019 | 0.002 | -0.050 | 0.013 | -0.059 | 0.016 | -0.039 | 0.016 |
| | rs7530169 | 1 | 50168591 | T | C | 0.019 | 0.002 | -0.049 | 0.013 | -0.043 | 0.017 | -0.054 | 0.017 |
| | rs4926853 | 1 | 50709222 | A | G | 0.012 | 0.002 | -0.036 | 0.014 | -0.038 | 0.018 | -0.034 | 0.017 |
| | rs3766430 | 1 | 54730651 | C | T | 0.011 | 0.002 | -0.003 | 0.013 | -0.003 | 0.016 | -0.001 | 0.016 |
| | rs17424278 | 1 | 57846394 | C | A | 0.017 | 0.003 | -0.032 | 0.020 | -0.030 | 0.026 | -0.028 | 0.026 |
| | rs2481665 | 1 | 62594677 | T | C | 0.016 | 0.002 | -0.006 | 0.013 | 0.011 | 0.016 | -0.017 | 0.016 |
| | rs10889550 | 1 | 65906067 | G | A | 0.024 | 0.004 | -0.037 | 0.031 | -0.024 | 0.040 | -0.052 | 0.040 |
| | rs7519259 | 1 | 66434743 | A | G | 0.013 | 0.002 | -0.008 | 0.012 | -0.016 | 0.016 | 0.001 | 0.016 |
| | rs1993709 | 1 | 72838529 | G | A | 0.033 | 0.002 | 0.018 | 0.016 | 0.011 | 0.021 | 0.009 | 0.020 |
| | rs7551507 | 1 | 74995225 | C | T | 0.018 | 0.002 | -0.007 | 0.013 | 0.001 | 0.016 | -0.006 | 0.016 |

| | | | | | | | | | | | | |
|---|---|---|---|---|---|---|---|---|---|---|---|---|
| rs12049202 | 1 | 77967523 | T | C | 0.024 | 0.002 | -0.037 | 0.016 | -0.062 | 0.020 | -0.003 | 0.020 |
| rs17391694 | 1 | 78623626 | T | C | 0.032 | 0.003 | -0.066 | 0.019 | -0.114 | 0.026 | -0.016 | 0.024 |
| rs12065553 | 1 | 80793118 | G | A | 0.012 | 0.002 | 0.012 | 0.014 | 0.026 | 0.018 | 0.011 | 0.017 |
| rs284227 | 1 | 82379446 | C | T | 0.015 | 0.002 | -0.001 | 0.015 | 0.008 | 0.019 | -0.011 | 0.019 |
| rs6690764 | 1 | 92976590 | G | A | 0.015 | 0.002 | 0.025 | 0.015 | 0.009 | 0.020 | 0.037 | 0.020 |
| rs1361739 | 1 | 96289734 | G | A | 0.018 | 0.002 | -0.018 | 0.013 | -0.021 | 0.017 | -0.020 | 0.017 |
| rs11165643 | 1 | 96924097 | T | C | 0.021 | 0.002 | -0.009 | 0.013 | -0.019 | 0.016 | -0.006 | 0.016 |
| rs995258 | 1 | 97431052 | A | C | 0.014 | 0.002 | -0.011 | 0.013 | -0.005 | 0.016 | -0.020 | 0.016 |
| rs4523552 | 1 | 98328093 | T | C | 0.014 | 0.002 | 0.000 | 0.015 | 0.010 | 0.020 | -0.013 | 0.019 |
| rs1730859 | 1 | 107617707 | G | A | 0.012 | 0.002 | -0.047 | 0.013 | -0.028 | 0.017 | -0.055 | 0.017 |
| rs17531363 | 1 | 107977075 | A | C | 0.013 | 0.002 | -0.022 | 0.014 | -0.037 | 0.018 | 0.004 | 0.017 |
| rs7550711 | 1 | 110082886 | T | C | 0.065 | 0.005 | -0.054 | 0.039 | -0.048 | 0.050 | -0.060 | 0.049 |
| rs12033257 | 1 | 112318484 | A | G | 0.015 | 0.002 | -0.002 | 0.014 | -0.006 | 0.017 | 0.003 | 0.018 |
| rs2007231 | 1 | 115266306 | C | T | 0.010 | 0.002 | 0.011 | 0.013 | -0.012 | 0.017 | 0.023 | 0.017 |
| rs7534091 | 1 | 118864616 | G | A | 0.011 | 0.002 | 0.015 | 0.014 | 0.017 | 0.018 | 0.019 | 0.018 |
| rs10923724 | 1 | 119546842 | C | T | 0.012 | 0.002 | 0.013 | 0.013 | 0.007 | 0.016 | 0.023 | 0.016 |
| rs6587552 | 1 | 151018861 | A | G | 0.017 | 0.002 | 0.022 | 0.015 | 0.027 | 0.019 | 0.012 | 0.019 |
| rs905938 | 1 | 154991389 | C | T | 0.015 | 0.002 | 0.037 | 0.014 | 0.036 | 0.019 | 0.037 | 0.018 |
| rs6695119 | 1 | 155739430 | T | C | 0.041 | 0.006 | 0.005 | 0.045 | 0.038 | 0.059 | -0.010 | 0.057 |
| rs4414033 | 1 | 156406853 | A | G | 0.013 | 0.002 | -0.010 | 0.013 | -0.014 | 0.017 | -0.010 | 0.017 |
| rs10733051 | 1 | 167280354 | A | G | 0.010 | 0.002 | 0.011 | 0.012 | 0.009 | 0.016 | 0.006 | 0.016 |
| rs16864515 | 1 | 171435542 | A | C | 0.019 | 0.003 | -0.024 | 0.021 | -0.050 | 0.027 | -0.001 | 0.027 |
| rs13375205 | 1 | 173947191 | A | G | 0.019 | 0.003 | 0.000 | 0.019 | 0.004 | 0.025 | -0.005 | 0.024 |
| rs12564992 | 1 | 174478100 | G | A | 0.020 | 0.003 | -0.002 | 0.019 | 0.009 | 0.025 | -0.009 | 0.025 |
| rs6691857 | 1 | 174885129 | C | T | 0.035 | 0.005 | 0.011 | 0.036 | -0.008 | 0.046 | 0.016 | 0.046 |
| rs11581304 | 1 | 177383443 | C | T | 0.018 | 0.003 | 0.014 | 0.021 | -0.017 | 0.027 | 0.045 | 0.027 |
| rs543874 | 1 | 177889480 | G | A | 0.048 | 0.002 | -0.027 | 0.016 | -0.004 | 0.020 | -0.045 | 0.020 |
| rs10920678 | 1 | 190239907 | A | G | 0.016 | 0.002 | -0.002 | 0.013 | 0.003 | 0.016 | 0.006 | 0.016 |
| rs645482 | 1 | 191386639 | A | G | 0.011 | 0.002 | -0.019 | 0.014 | -0.009 | 0.017 | -0.022 | 0.017 |
| rs12041258 | 1 | 195047936 | T | C | 0.015 | 0.002 | 0.007 | 0.015 | 0.014 | 0.019 | 0.007 | 0.019 |
| rs6676084 | 1 | 197094030 | C | T | 0.012 | 0.002 | 0.028 | 0.013 | 0.005 | 0.017 | 0.041 | 0.017 |
| rs2820311 | 1 | 201841476 | G | A | 0.024 | 0.002 | 0.023 | 0.013 | 0.020 | 0.017 | 0.027 | 0.017 |
| rs9077 | 1 | 202116238 | G | A | 0.014 | 0.002 | -0.006 | 0.014 | -0.011 | 0.017 | -0.005 | 0.017 |
| rs823074 | 1 | 205774839 | T | C | 0.011 | 0.002 | -0.035 | 0.013 | -0.028 | 0.016 | -0.049 | 0.016 |
| rs17014375 | 1 | 209543560 | G | T | 0.017 | 0.003 | -0.005 | 0.018 | -0.012 | 0.024 | 0.000 | 0.023 |
| rs6661316 | 1 | 210095527 | T | C | 0.011 | 0.002 | 0.000 | 0.013 | 0.012 | 0.016 | -0.013 | 0.016 |
| rs11118308 | 1 | 219633869 | A | G | 0.010 | 0.002 | 0.019 | 0.013 | 0.019 | 0.016 | 0.022 | 0.016 |
| rs10915840 | 1 | 225668524 | G | A | 0.012 | 0.002 | 0.015 | 0.014 | 0.034 | 0.018 | 0.009 | 0.018 |
| rs2491864 | 1 | 242986063 | A | G | 0.014 | 0.002 | -0.015 | 0.015 | 0.000 | 0.019 | -0.029 | 0.019 |
| rs946824 | 1 | 243684019 | T | C | 0.021 | 0.003 | -0.027 | 0.018 | -0.033 | 0.024 | -0.016 | 0.023 |
| rs13021737 | 2 | 632348 | G | A | 0.057 | 0.002 | -0.018 | 0.016 | -0.004 | 0.021 | -0.027 | 0.021 |
| rs10929925 | 2 | 6155557 | C | A | 0.014 | 0.002 | 0.027 | 0.013 | 0.022 | 0.016 | 0.026 | 0.016 |
| rs10182181 | 2 | 25150296 | G | A | 0.033 | 0.002 | 0.081 | 0.012 | 0.107 | 0.016 | 0.057 | 0.016 |
| rs12468863 | 2 | 26940294 | C | T | 0.015 | 0.002 | 0.006 | 0.012 | -0.013 | 0.016 | 0.022 | 0.016 |
| rs7567655 | 2 | 28273595 | G | A | 0.027 | 0.005 | 0.053 | 0.034 | 0.030 | 0.043 | 0.080 | 0.043 |
| rs4372836 | 2 | 28973883 | T | C | 0.014 | 0.002 | -0.018 | 0.013 | -0.017 | 0.017 | -0.010 | 0.017 |
| rs17327461 | 2 | 35512183 | T | C | 0.013 | 0.002 | -0.006 | 0.012 | -0.006 | 0.016 | -0.011 | 0.016 |
| rs17019087 | 2 | 36783599 | C | T | 0.011 | 0.002 | -0.001 | 0.013 | 0.006 | 0.017 | 0.003 | 0.016 |
| rs4670627 | 2 | 37046711 | T | C | 0.011 | 0.002 | 0.008 | 0.013 | 0.003 | 0.017 | 0.011 | 0.016 |
| rs6741690 | 2 | 40291593 | A | G | 0.012 | 0.002 | -0.002 | 0.014 | 0.006 | 0.018 | -0.007 | 0.018 |
| rs2063177 | 2 | 41712849 | A | G | 0.012 | 0.002 | 0.029 | 0.013 | 0.034 | 0.017 | 0.030 | 0.016 |

| | | | | | | | | | | | | |
|---|---|---|---|---|---|---|---|---|---|---|---|---|
| rs4953577 | 2 | 42935961 | T | C | 0.010 | 0.002 | 0.000 | 0.012 | 0.018 | 0.016 | -0.007 | 0.016 |
| rs13425435 | 2 | 44776597 | A | C | 0.012 | 0.002 | -0.027 | 0.013 | -0.016 | 0.017 | -0.028 | 0.017 |
| rs13012099 | 2 | 47010742 | G | A | 0.014 | 0.002 | -0.005 | 0.013 | 0.012 | 0.017 | -0.014 | 0.017 |
| rs7561278 | 2 | 48954905 | T | C | 0.016 | 0.002 | 0.001 | 0.015 | -0.007 | 0.020 | 0.005 | 0.019 |
| rs930295 | 2 | 50233352 | A | C | 0.021 | 0.002 | -0.012 | 0.017 | -0.003 | 0.022 | -0.030 | 0.021 |
| rs13406839 | 2 | 50735433 | A | G | 0.013 | 0.002 | 0.003 | 0.013 | -0.004 | 0.016 | 0.001 | 0.016 |
| rs3806572 | 2 | 55238677 | G | A | 0.015 | 0.002 | 0.009 | 0.014 | 0.043 | 0.018 | -0.016 | 0.018 |
| rs13432055 | 2 | 56603985 | C | T | 0.012 | 0.002 | 0.019 | 0.014 | 0.014 | 0.018 | 0.029 | 0.018 |
| rs1345942 | 2 | 57335044 | C | T | 0.010 | 0.002 | 0.012 | 0.013 | 0.017 | 0.017 | 0.008 | 0.016 |
| rs1106090 | 2 | 58068741 | G | A | 0.013 | 0.002 | -0.021 | 0.013 | -0.011 | 0.016 | -0.028 | 0.016 |
| rs4671328 | 2 | 58935282 | T | G | 0.022 | 0.002 | 0.002 | 0.013 | 0.019 | 0.016 | -0.007 | 0.016 |
| rs12053103 | 2 | 59540594 | A | G | 0.012 | 0.002 | -0.008 | 0.013 | -0.014 | 0.016 | -0.003 | 0.016 |
| rs12476772 | 2 | 60203917 | A | C | 0.012 | 0.002 | -0.033 | 0.013 | -0.039 | 0.017 | -0.040 | 0.017 |
| rs2195086 | 2 | 60814466 | G | T | 0.013 | 0.002 | -0.020 | 0.018 | -0.034 | 0.023 | -0.003 | 0.022 |
| rs13417156 | 2 | 62848319 | C | T | 0.014 | 0.002 | 0.005 | 0.013 | 0.007 | 0.017 | 0.002 | 0.016 |
| rs12477088 | 2 | 67841326 | T | C | 0.015 | 0.002 | 0.026 | 0.013 | 0.009 | 0.016 | 0.053 | 0.016 |
| rs7607351 | 2 | 69562127 | T | C | 0.012 | 0.002 | -0.008 | 0.013 | -0.012 | 0.016 | -0.013 | 0.016 |
| rs934515 | 2 | 79482643 | A | G | 0.019 | 0.003 | 0.004 | 0.019 | 0.016 | 0.025 | -0.009 | 0.025 |
| rs1371108 | 2 | 81816251 | A | C | 0.012 | 0.002 | 0.002 | 0.013 | 0.014 | 0.017 | -0.012 | 0.017 |
| rs7557796 | 2 | 86766153 | T | C | 0.016 | 0.002 | -0.001 | 0.013 | 0.009 | 0.017 | -0.009 | 0.017 |
| rs4556997 | 2 | 100814858 | A | C | 0.020 | 0.002 | -0.035 | 0.018 | -0.008 | 0.023 | -0.074 | 0.023 |
| rs6707445 | 2 | 104420858 | A | G | 0.013 | 0.002 | 0.006 | 0.013 | 0.000 | 0.016 | 0.014 | 0.016 |
| rs10197031 | 2 | 105454590 | C | T | 0.017 | 0.002 | -0.011 | 0.014 | -0.006 | 0.018 | -0.011 | 0.018 |
| rs731834 | 2 | 113957198 | A | C | 0.011 | 0.002 | -0.046 | 0.012 | -0.058 | 0.016 | -0.030 | 0.016 |
| rs4954638 | 2 | 137435455 | A | C | 0.012 | 0.002 | -0.011 | 0.014 | -0.016 | 0.018 | -0.001 | 0.018 |
| rs17551974 | 2 | 142293146 | C | A | 0.014 | 0.002 | 0.001 | 0.016 | 0.028 | 0.021 | -0.029 | 0.020 |
| rs2890652 | 2 | 142959931 | C | T | 0.017 | 0.002 | 0.024 | 0.017 | 0.010 | 0.022 | 0.018 | 0.022 |
| rs6710871 | 2 | 143960593 | A | G | 0.018 | 0.002 | 0.005 | 0.018 | -0.046 | 0.023 | 0.047 | 0.023 |
| rs7560871 | 2 | 145616899 | A | G | 0.022 | 0.003 | -0.108 | 0.024 | -0.117 | 0.032 | -0.100 | 0.031 |
| rs429343 | 2 | 147903382 | A | G | 0.015 | 0.002 | 0.021 | 0.013 | 0.016 | 0.016 | 0.032 | 0.016 |
| rs2652432 | 2 | 155734955 | G | T | 0.013 | 0.002 | -0.021 | 0.016 | -0.027 | 0.021 | -0.023 | 0.021 |
| rs16839578 | 2 | 156629693 | C | T | 0.011 | 0.002 | -0.013 | 0.013 | -0.023 | 0.017 | -0.006 | 0.017 |
| rs10198345 | 2 | 157109395 | C | T | 0.011 | 0.002 | 0.002 | 0.013 | 0.005 | 0.017 | 0.011 | 0.017 |
| rs3764835 | 2 | 159519368 | G | A | 0.014 | 0.002 | -0.023 | 0.017 | -0.015 | 0.022 | -0.015 | 0.022 |
| rs12692596 | 2 | 161265910 | T | C | 0.011 | 0.002 | -0.034 | 0.013 | -0.020 | 0.017 | -0.039 | 0.016 |
| rs10192119 | 2 | 164581241 | G | T | 0.017 | 0.002 | 0.001 | 0.017 | -0.012 | 0.022 | 0.013 | 0.021 |
| rs11677541 | 2 | 165429235 | G | A | 0.012 | 0.002 | -0.017 | 0.012 | -0.010 | 0.016 | -0.025 | 0.016 |
| rs3769948 | 2 | 166186923 | A | G | 0.013 | 0.002 | -0.022 | 0.014 | -0.020 | 0.019 | -0.025 | 0.018 |
| rs535533 | 2 | 166959505 | C | T | 0.010 | 0.002 | 0.008 | 0.013 | 0.007 | 0.016 | 0.004 | 0.016 |
| rs6738445 | 2 | 172599615 | C | T | 0.013 | 0.002 | -0.010 | 0.014 | -0.007 | 0.018 | -0.020 | 0.018 |
| rs7573263 | 2 | 174980833 | T | C | 0.014 | 0.002 | -0.010 | 0.013 | -0.007 | 0.016 | -0.013 | 0.016 |
| rs1528435 | 2 | 181550962 | T | C | 0.016 | 0.002 | 0.008 | 0.013 | 0.014 | 0.017 | -0.001 | 0.016 |
| rs2029085 | 2 | 187207393 | G | A | 0.016 | 0.003 | -0.031 | 0.020 | -0.034 | 0.026 | -0.023 | 0.026 |
| rs10497810 | 2 | 198895732 | C | T | 0.017 | 0.002 | 0.026 | 0.016 | -0.025 | 0.020 | 0.066 | 0.021 |
| rs4482463 | 2 | 205375909 | C | A | 0.033 | 0.003 | 0.010 | 0.023 | 0.004 | 0.030 | 0.006 | 0.029 |
| rs6435277 | 2 | 206087701 | G | A | 0.011 | 0.002 | 0.012 | 0.013 | 0.015 | 0.016 | 0.004 | 0.016 |
| rs12989476 | 2 | 207197155 | T | C | 0.013 | 0.002 | 0.012 | 0.013 | 0.027 | 0.017 | -0.006 | 0.017 |
| rs1263618 | 2 | 207956108 | C | T | 0.012 | 0.002 | 0.006 | 0.013 | 0.007 | 0.017 | 0.000 | 0.017 |
| rs17203016 | 2 | 208255518 | G | A | 0.015 | 0.002 | 0.008 | 0.016 | -0.018 | 0.021 | 0.014 | 0.020 |
| rs4673553 | 2 | 211608379 | G | T | 0.014 | 0.002 | -0.018 | 0.012 | -0.027 | 0.016 | -0.016 | 0.016 |
| rs7421089 | 2 | 211988412 | T | C | 0.014 | 0.002 | -0.022 | 0.014 | -0.019 | 0.018 | -0.031 | 0.017 |

| | | | | | | | | | | | | |
|---|---|---|---|---|---|---|---|---|---|---|---|---|
| rs16846136 | 2 | 212291371 | A | C | 0.013 | 0.002 | 0.034 | 0.014 | 0.015 | 0.018 | 0.048 | 0.018 |
| rs7599312 | 2 | 213413231 | G | A | 0.019 | 0.002 | -0.022 | 0.014 | -0.018 | 0.018 | -0.017 | 0.018 |
| rs7607369 | 2 | 219279097 | A | G | 0.012 | 0.002 | 0.011 | 0.013 | 0.030 | 0.016 | -0.008 | 0.016 |
| rs11889536 | 2 | 220163543 | A | G | 0.019 | 0.002 | 0.010 | 0.017 | -0.012 | 0.022 | 0.023 | 0.022 |
| rs4500930 | 2 | 228985505 | T | C | 0.016 | 0.002 | -0.016 | 0.013 | 0.001 | 0.017 | -0.029 | 0.017 |
| rs2162524 | 2 | 230817437 | C | T | 0.016 | 0.002 | -0.012 | 0.013 | -0.009 | 0.017 | -0.012 | 0.017 |
| rs4663629 | 2 | 236801872 | A | G | 0.013 | 0.002 | 0.001 | 0.015 | 0.004 | 0.019 | 0.003 | 0.019 |
| rs7652415 | 3 | 9505238 | T | C | 0.016 | 0.003 | 0.022 | 0.018 | 0.022 | 0.023 | 0.025 | 0.023 |
| rs17776719 | 3 | 11640481 | G | A | 0.017 | 0.003 | -0.029 | 0.017 | -0.083 | 0.023 | 0.010 | 0.022 |
| rs10510419 | 3 | 12426936 | G | T | 0.018 | 0.002 | 0.026 | 0.018 | 0.014 | 0.023 | 0.042 | 0.022 |
| rs2600226 | 3 | 12928762 | C | T | 0.012 | 0.002 | 0.001 | 0.013 | -0.005 | 0.017 | -0.004 | 0.017 |
| rs9845966 | 3 | 13433158 | T | G | 0.011 | 0.002 | 0.005 | 0.012 | -0.009 | 0.016 | 0.014 | 0.016 |
| rs11128760 | 3 | 15873407 | A | G | 0.011 | 0.002 | -0.010 | 0.013 | -0.015 | 0.016 | -0.001 | 0.016 |
| rs4858193 | 3 | 20441050 | T | C | 0.013 | 0.002 | 0.009 | 0.014 | 0.021 | 0.018 | 0.010 | 0.018 |
| rs6804842 | 3 | 25106437 | G | A | 0.016 | 0.002 | 0.003 | 0.013 | 0.008 | 0.016 | -0.004 | 0.016 |
| rs11921432 | 3 | 35117776 | C | T | 0.018 | 0.003 | 0.005 | 0.020 | -0.026 | 0.026 | 0.032 | 0.025 |
| rs11129662 | 3 | 35696096 | G | A | 0.012 | 0.002 | 0.019 | 0.013 | 0.027 | 0.017 | 0.010 | 0.017 |
| rs13072731 | 3 | 38533335 | C | A | 0.011 | 0.002 | -0.008 | 0.013 | -0.016 | 0.016 | -0.003 | 0.016 |
| rs9814633 | 3 | 41310470 | A | G | 0.012 | 0.002 | 0.008 | 0.013 | 0.011 | 0.017 | 0.004 | 0.017 |
| rs33485 | 3 | 42417982 | C | T | 0.016 | 0.002 | 0.005 | 0.014 | -0.002 | 0.019 | 0.007 | 0.018 |
| rs3852012 | 3 | 44045981 | G | A | 0.013 | 0.002 | 0.000 | 0.013 | 0.016 | 0.017 | -0.018 | 0.017 |
| rs4683096 | 3 | 45374496 | T | G | 0.012 | 0.002 | -0.018 | 0.012 | -0.008 | 0.016 | -0.031 | 0.016 |
| rs11716779 | 3 | 47606215 | G | A | 0.013 | 0.002 | -0.003 | 0.014 | -0.005 | 0.018 | -0.001 | 0.018 |
| rs11710798 | 3 | 48570686 | A | C | 0.017 | 0.003 | -0.099 | 0.021 | -0.113 | 0.026 | -0.084 | 0.026 |
| rs1800668 | 3 | 49395757 | G | A | 0.014 | 0.002 | -0.152 | 0.013 | -0.166 | 0.017 | -0.141 | 0.017 |
| rs11713193 | 3 | 49924424 | A | G | 0.024 | 0.002 | -0.079 | 0.012 | -0.080 | 0.016 | -0.086 | 0.016 |
| rs1034405 | 3 | 50597092 | A | G | 0.016 | 0.003 | 0.009 | 0.019 | -0.001 | 0.024 | 0.028 | 0.024 |
| rs6787805 | 3 | 51204788 | G | A | 0.017 | 0.002 | 0.008 | 0.019 | 0.017 | 0.024 | 0.006 | 0.024 |
| rs13090011 | 3 | 51728573 | A | G | 0.015 | 0.002 | 0.014 | 0.017 | 0.003 | 0.022 | 0.027 | 0.021 |
| rs353547 | 3 | 52268866 | T | C | 0.012 | 0.002 | -0.017 | 0.013 | -0.024 | 0.017 | -0.011 | 0.016 |
| rs2710323 | 3 | 52815905 | C | T | 0.014 | 0.002 | -0.014 | 0.012 | -0.005 | 0.016 | -0.016 | 0.016 |
| rs2680648 | 3 | 53777176 | T | C | 0.016 | 0.002 | 0.030 | 0.015 | 0.013 | 0.019 | 0.048 | 0.019 |
| rs12488237 | 3 | 56114861 | C | T | 0.024 | 0.004 | -0.042 | 0.028 | -0.038 | 0.036 | -0.030 | 0.035 |
| rs2365389 | 3 | 61236462 | C | T | 0.017 | 0.002 | -0.009 | 0.013 | -0.016 | 0.016 | -0.003 | 0.016 |
| rs1911746 | 3 | 62100840 | T | C | 0.012 | 0.002 | 0.009 | 0.015 | 0.002 | 0.019 | 0.021 | 0.019 |
| rs1452075 | 3 | 62481063 | T | C | 0.014 | 0.002 | -0.014 | 0.014 | -0.001 | 0.018 | -0.027 | 0.018 |
| rs17831815 | 3 | 66437086 | C | T | 0.011 | 0.002 | 0.005 | 0.013 | 0.016 | 0.017 | -0.004 | 0.017 |
| rs11915371 | 3 | 70539559 | C | A | 0.015 | 0.002 | 0.007 | 0.015 | 0.032 | 0.020 | -0.028 | 0.019 |
| rs1523768 | 3 | 77667044 | G | A | 0.011 | 0.002 | 0.014 | 0.013 | 0.016 | 0.017 | 0.010 | 0.017 |
| rs6419734 | 3 | 78458928 | T | C | 0.017 | 0.003 | -0.009 | 0.017 | 0.011 | 0.023 | -0.022 | 0.022 |
| rs328049 | 3 | 78985805 | A | G | 0.010 | 0.002 | 0.019 | 0.013 | 0.028 | 0.016 | 0.011 | 0.016 |
| rs6781254 | 3 | 80649139 | T | C | 0.011 | 0.002 | -0.006 | 0.014 | -0.017 | 0.018 | -0.007 | 0.017 |
| rs3849570 | 3 | 81792112 | A | C | 0.013 | 0.002 | 0.004 | 0.013 | -0.015 | 0.017 | 0.022 | 0.017 |
| rs716764 | 3 | 82646519 | T | C | 0.018 | 0.002 | 0.002 | 0.017 | -0.001 | 0.022 | -0.011 | 0.021 |
| rs9827823 | 3 | 84221774 | T | C | 0.019 | 0.002 | 0.008 | 0.017 | 0.007 | 0.022 | 0.015 | 0.022 |
| rs7628129 | 3 | 85095897 | C | T | 0.015 | 0.002 | -0.022 | 0.012 | -0.014 | 0.016 | -0.036 | 0.016 |
| rs2122042 | 3 | 85866335 | T | G | 0.024 | 0.002 | -0.062 | 0.015 | -0.050 | 0.019 | -0.078 | 0.019 |
| rs1006896 | 3 | 88104411 | A | C | 0.023 | 0.003 | 0.005 | 0.020 | -0.025 | 0.026 | 0.023 | 0.025 |
| rs13072412 | 3 | 88749998 | C | A | 0.011 | 0.002 | 0.012 | 0.012 | 0.017 | 0.016 | 0.002 | 0.016 |
| rs4857329 | 3 | 94036952 | A | G | 0.020 | 0.002 | 0.015 | 0.012 | 0.023 | 0.016 | 0.001 | 0.016 |
| rs1436343 | 3 | 104606130 | G | A | 0.014 | 0.002 | -0.001 | 0.013 | 0.001 | 0.016 | -0.002 | 0.016 |

| | | | | | | | | | | | | |
|---|---|---|---|---|---|---|---|---|---|---|---|---|
| rs13317489 | 3 | 107280241 | A | G | 0.013 | 0.002 | -0.036 | 0.016 | -0.064 | 0.021 | -0.012 | 0.021 |
| rs7640424 | 3 | 107820063 | C | T | 0.014 | 0.002 | 0.020 | 0.014 | 0.015 | 0.018 | 0.031 | 0.017 |
| rs13085471 | 3 | 108359880 | T | C | 0.011 | 0.002 | -0.027 | 0.013 | -0.021 | 0.016 | -0.029 | 0.016 |
| rs17619973 | 3 | 114417675 | A | G | 0.020 | 0.003 | 0.018 | 0.024 | 0.023 | 0.031 | 0.017 | 0.031 |
| rs2903971 | 3 | 116935744 | T | G | 0.015 | 0.002 | -0.015 | 0.016 | -0.023 | 0.021 | -0.009 | 0.020 |
| rs779206 | 3 | 118023515 | G | A | 0.013 | 0.002 | 0.006 | 0.014 | 0.028 | 0.018 | -0.013 | 0.018 |
| rs12629015 | 3 | 119618053 | A | G | 0.014 | 0.002 | 0.011 | 0.016 | -0.008 | 0.021 | 0.019 | 0.021 |
| rs10668 | 3 | 123211064 | G | T | 0.013 | 0.002 | -0.015 | 0.014 | -0.005 | 0.018 | -0.022 | 0.017 |
| rs2270778 | 3 | 124692689 | C | T | 0.010 | 0.002 | 0.034 | 0.013 | 0.036 | 0.017 | 0.032 | 0.016 |
| rs9848399 | 3 | 125205106 | A | G | 0.016 | 0.003 | 0.000 | 0.019 | 0.011 | 0.025 | -0.016 | 0.024 |
| rs1320903 | 3 | 131758077 | A | G | 0.022 | 0.002 | -0.006 | 0.013 | 0.013 | 0.017 | -0.015 | 0.017 |
| rs10935143 | 3 | 134665159 | G | A | 0.011 | 0.002 | -0.004 | 0.012 | -0.006 | 0.016 | -0.010 | 0.016 |
| rs13079205 | 3 | 135800693 | G | A | 0.015 | 0.002 | -0.021 | 0.014 | -0.016 | 0.018 | -0.027 | 0.018 |
| rs12630999 | 3 | 136300927 | A | G | 0.018 | 0.002 | -0.015 | 0.014 | -0.018 | 0.018 | -0.004 | 0.018 |
| rs1199334 | 3 | 138091140 | A | G | 0.014 | 0.002 | -0.019 | 0.016 | -0.008 | 0.020 | -0.024 | 0.020 |
| rs16851483 | 3 | 141275436 | T | G | 0.037 | 0.004 | 0.009 | 0.025 | -0.002 | 0.032 | 0.023 | 0.032 |
| rs171390 | 3 | 154038412 | C | T | 0.015 | 0.002 | -0.004 | 0.013 | 0.010 | 0.016 | -0.013 | 0.016 |
| rs6441080 | 3 | 156298935 | A | G | 0.015 | 0.002 | -0.009 | 0.017 | -0.014 | 0.022 | 0.003 | 0.022 |
| rs13081038 | 3 | 156950732 | G | A | 0.012 | 0.002 | 0.006 | 0.013 | 0.002 | 0.017 | 0.011 | 0.017 |
| rs827092 | 3 | 157985182 | T | C | 0.013 | 0.002 | 0.023 | 0.013 | 0.023 | 0.016 | 0.025 | 0.016 |
| rs1476322 | 3 | 161446055 | A | G | 0.010 | 0.002 | -0.004 | 0.012 | -0.015 | 0.016 | 0.009 | 0.016 |
| rs8192675 | 3 | 170724883 | C | T | 0.015 | 0.002 | -0.009 | 0.014 | -0.009 | 0.018 | -0.011 | 0.017 |
| rs488029 | 3 | 173119378 | A | G | 0.015 | 0.002 | 0.001 | 0.013 | 0.005 | 0.016 | -0.008 | 0.016 |
| rs9844274 | 3 | 173666966 | C | T | 0.010 | 0.002 | 0.009 | 0.014 | 0.008 | 0.018 | 0.010 | 0.018 |
| rs3860517 | 3 | 180804471 | A | G | 0.011 | 0.002 | -0.027 | 0.013 | -0.010 | 0.017 | -0.034 | 0.017 |
| rs6443750 | 3 | 181329682 | C | T | 0.015 | 0.002 | 0.031 | 0.018 | 0.032 | 0.024 | 0.030 | 0.023 |
| rs16833232 | 3 | 182262873 | C | T | 0.011 | 0.002 | -0.014 | 0.013 | -0.013 | 0.017 | -0.013 | 0.017 |
| rs263041 | 3 | 183522231 | A | G | 0.012 | 0.002 | -0.003 | 0.013 | -0.013 | 0.017 | 0.005 | 0.016 |
| rs2293605 | 3 | 184044433 | C | T | 0.017 | 0.003 | 0.028 | 0.020 | 0.036 | 0.026 | 0.015 | 0.025 |
| rs1516725 | 3 | 185824004 | C | T | 0.036 | 0.002 | -0.029 | 0.018 | -0.031 | 0.023 | -0.038 | 0.023 |
| rs4677812 | 3 | 194863794 | C | A | 0.014 | 0.002 | 0.007 | 0.014 | 0.015 | 0.018 | 0.003 | 0.017 |
| rs6764533 | 3 | 196088464 | A | G | 0.012 | 0.002 | -0.038 | 0.013 | -0.041 | 0.017 | -0.039 | 0.017 |
| rs2051559 | 4 | 3298800 | C | T | 0.018 | 0.003 | 0.006 | 0.018 | 0.024 | 0.023 | -0.002 | 0.023 |
| rs2320726 | 4 | 18383743 | G | A | 0.014 | 0.002 | -0.010 | 0.012 | -0.005 | 0.016 | -0.018 | 0.016 |
| rs6849518 | 4 | 20120274 | T | C | 0.017 | 0.003 | 0.006 | 0.019 | 0.004 | 0.025 | -0.007 | 0.024 |
| rs6850639 | 4 | 21010996 | T | C | 0.012 | 0.002 | 0.000 | 0.016 | 0.008 | 0.020 | -0.012 | 0.020 |
| rs6841761 | 4 | 25423538 | G | T | 0.013 | 0.002 | 0.019 | 0.012 | 0.038 | 0.016 | 0.006 | 0.016 |
| rs6448587 | 4 | 28561990 | A | C | 0.017 | 0.002 | 0.005 | 0.016 | 0.014 | 0.021 | -0.006 | 0.020 |
| rs10007906 | 4 | 31023610 | A | C | 0.013 | 0.002 | -0.003 | 0.013 | 0.001 | 0.017 | -0.014 | 0.017 |
| rs12502727 | 4 | 38344189 | G | A | 0.016 | 0.003 | 0.008 | 0.020 | 0.038 | 0.026 | -0.014 | 0.025 |
| rs3209570 | 4 | 38699657 | G | A | 0.014 | 0.002 | 0.049 | 0.013 | 0.063 | 0.017 | 0.032 | 0.016 |
| rs10009336 | 4 | 44480783 | C | T | 0.014 | 0.002 | -0.017 | 0.017 | -0.032 | 0.022 | -0.004 | 0.021 |
| rs10938397 | 4 | 45182527 | G | A | 0.032 | 0.002 | -0.006 | 0.013 | -0.005 | 0.016 | -0.003 | 0.016 |
| rs2768950 | 4 | 49064487 | A | G | 0.012 | 0.002 | -0.005 | 0.014 | -0.002 | 0.018 | 0.004 | 0.018 |
| rs784944 | 4 | 52927229 | A | G | 0.012 | 0.002 | -0.008 | 0.014 | 0.004 | 0.018 | -0.008 | 0.018 |
| rs2192158 | 4 | 55505360 | A | G | 0.013 | 0.002 | 0.010 | 0.012 | 0.033 | 0.016 | -0.014 | 0.016 |
| rs11945861 | 4 | 65700865 | G | A | 0.015 | 0.002 | 0.030 | 0.015 | 0.031 | 0.019 | 0.040 | 0.019 |
| rs17001561 | 4 | 77096118 | A | G | 0.015 | 0.002 | 0.021 | 0.017 | 0.017 | 0.022 | 0.034 | 0.022 |
| rs7674623 | 4 | 80794681 | T | C | 0.014 | 0.002 | 0.005 | 0.016 | 0.005 | 0.020 | 0.006 | 0.020 |
| rs4148155 | 4 | 89054667 | A | G | 0.019 | 0.003 | -0.003 | 0.020 | -0.023 | 0.026 | 0.014 | 0.025 |
| rs2169399 | 4 | 91244218 | C | T | 0.011 | 0.002 | 0.011 | 0.012 | 0.005 | 0.016 | 0.017 | 0.016 |

| | | | | | | | | | | | | |
|---|---|---|---|---|---|---|---|---|---|---|---|---|
| rs1485038 | 4 | 94409429 | T | C | 0.014 | 0.002 | -0.009 | 0.016 | -0.009 | 0.020 | -0.016 | 0.020 |
| rs7685048 | 4 | 95027784 | C | T | 0.010 | 0.002 | -0.040 | 0.012 | -0.042 | 0.016 | -0.026 | 0.016 |
| rs1863652 | 4 | 95991417 | G | A | 0.012 | 0.002 | 0.011 | 0.013 | 0.009 | 0.017 | 0.000 | 0.017 |
| rs2850969 | 4 | 102183594 | C | T | 0.017 | 0.002 | 0.019 | 0.017 | 0.046 | 0.022 | 0.011 | 0.022 |
| rs13149344 | 4 | 102686178 | G | A | 0.012 | 0.002 | 0.032 | 0.015 | 0.036 | 0.019 | 0.031 | 0.019 |
| rs13107325 | 4 | 103188709 | T | C | 0.047 | 0.003 | 0.113 | 0.023 | 0.201 | 0.028 | 0.033 | 0.030 |
| rs223391 | 4 | 103748224 | G | A | 0.013 | 0.002 | -0.006 | 0.016 | 0.043 | 0.021 | -0.049 | 0.020 |
| rs326889 | 4 | 112713436 | C | T | 0.013 | 0.002 | 0.006 | 0.013 | 0.027 | 0.016 | -0.006 | 0.016 |
| rs4834272 | 4 | 113313986 | C | T | 0.012 | 0.002 | 0.004 | 0.013 | -0.003 | 0.017 | 0.013 | 0.017 |
| rs7694732 | 4 | 115124089 | A | G | 0.010 | 0.002 | 0.015 | 0.012 | 0.039 | 0.016 | -0.004 | 0.016 |
| rs4864201 | 4 | 130731284 | T | C | 0.014 | 0.002 | -0.028 | 0.013 | -0.026 | 0.017 | -0.024 | 0.017 |
| rs1296328 | 4 | 137083193 | A | C | 0.018 | 0.002 | 0.011 | 0.013 | 0.004 | 0.017 | 0.015 | 0.016 |
| rs769657 | 4 | 140877659 | A | G | 0.014 | 0.002 | -0.012 | 0.013 | 0.013 | 0.017 | -0.026 | 0.017 |
| rs331949 | 4 | 143663206 | C | T | 0.011 | 0.002 | -0.009 | 0.013 | -0.001 | 0.017 | -0.017 | 0.016 |
| rs6852276 | 4 | 145261999 | G | A | 0.011 | 0.002 | 0.017 | 0.013 | 0.023 | 0.016 | 0.015 | 0.016 |
| rs1455137 | 4 | 145986668 | C | A | 0.011 | 0.002 | -0.001 | 0.013 | -0.012 | 0.017 | 0.002 | 0.016 |
| rs11733337 | 4 | 147372978 | T | G | 0.014 | 0.002 | 0.017 | 0.014 | 0.020 | 0.018 | 0.015 | 0.018 |
| rs6827083 | 4 | 153075491 | G | A | 0.010 | 0.002 | 0.006 | 0.013 | 0.021 | 0.016 | -0.013 | 0.016 |
| rs13110266 | 4 | 162129844 | G | A | 0.012 | 0.002 | 0.008 | 0.013 | 0.000 | 0.016 | 0.015 | 0.016 |
| rs17538472 | 4 | 163038241 | T | C | 0.013 | 0.002 | 0.039 | 0.016 | 0.033 | 0.020 | 0.043 | 0.020 |
| rs1522569 | 4 | 171632637 | T | G | 0.016 | 0.002 | 0.000 | 0.016 | -0.014 | 0.020 | 0.017 | 0.020 |
| rs1158684 | 4 | 173584086 | A | G | 0.010 | 0.002 | -0.012 | 0.012 | 0.004 | 0.016 | -0.016 | 0.016 |
| rs7683836 | 4 | 180167906 | G | A | 0.011 | 0.002 | 0.014 | 0.013 | -0.004 | 0.016 | 0.026 | 0.016 |
| rs16871902 | 5 | 3488462 | A | G | 0.013 | 0.002 | -0.034 | 0.013 | -0.025 | 0.016 | -0.035 | 0.016 |
| rs4518345 | 5 | 27185904 | G | A | 0.012 | 0.002 | 0.011 | 0.014 | -0.001 | 0.018 | 0.023 | 0.018 |
| rs7730004 | 5 | 43191033 | T | C | 0.015 | 0.002 | 0.009 | 0.013 | 0.011 | 0.017 | 0.013 | 0.017 |
| rs13168660 | 5 | 50412012 | A | G | 0.017 | 0.003 | 0.025 | 0.019 | 0.058 | 0.024 | 0.004 | 0.024 |
| rs12189178 | 5 | 50914726 | T | C | 0.036 | 0.005 | -0.076 | 0.035 | -0.082 | 0.045 | -0.077 | 0.044 |
| rs4865796 | 5 | 53272664 | G | A | 0.010 | 0.002 | 0.000 | 0.013 | 0.016 | 0.017 | -0.015 | 0.017 |
| rs6449532 | 5 | 60715446 | C | T | 0.013 | 0.002 | 0.032 | 0.013 | 0.021 | 0.017 | 0.041 | 0.017 |
| rs1503526 | 5 | 63020706 | C | T | 0.014 | 0.002 | 0.003 | 0.012 | 0.012 | 0.016 | -0.016 | 0.016 |
| rs2367112 | 5 | 64168193 | T | G | 0.012 | 0.002 | -0.002 | 0.012 | -0.011 | 0.016 | 0.010 | 0.016 |
| rs460799 | 5 | 66191971 | G | A | 0.012 | 0.002 | 0.013 | 0.014 | -0.001 | 0.018 | 0.024 | 0.018 |
| rs13153166 | 5 | 71494931 | A | C | 0.023 | 0.004 | 0.017 | 0.030 | -0.016 | 0.038 | 0.055 | 0.038 |
| rs2973564 | 5 | 73151033 | A | G | 0.011 | 0.002 | 0.023 | 0.014 | 0.018 | 0.018 | 0.025 | 0.017 |
| rs4621537 | 5 | 74362222 | A | G | 0.020 | 0.002 | 0.015 | 0.013 | 0.003 | 0.017 | 0.022 | 0.016 |
| rs2307111 | 5 | 75003678 | T | C | 0.027 | 0.002 | 0.009 | 0.013 | -0.011 | 0.016 | 0.030 | 0.016 |
| rs252749 | 5 | 77389973 | G | A | 0.012 | 0.002 | 0.007 | 0.014 | -0.004 | 0.019 | 0.023 | 0.018 |
| rs10942267 | 5 | 80841914 | A | G | 0.016 | 0.002 | 0.003 | 0.013 | -0.002 | 0.017 | 0.004 | 0.017 |
| rs10942476 | 5 | 86172206 | G | A | 0.010 | 0.002 | -0.026 | 0.012 | -0.009 | 0.016 | -0.040 | 0.016 |
| rs323781 | 5 | 86752616 | C | A | 0.038 | 0.005 | -0.030 | 0.037 | -0.042 | 0.047 | -0.019 | 0.047 |
| rs10474271 | 5 | 87454270 | T | C | 0.014 | 0.002 | 0.010 | 0.015 | 0.032 | 0.019 | -0.004 | 0.019 |
| rs16903285 | 5 | 87978252 | C | T | 0.033 | 0.003 | -0.031 | 0.018 | -0.017 | 0.024 | -0.037 | 0.023 |
| rs16867703 | 5 | 88799143 | G | T | 0.014 | 0.002 | 0.011 | 0.013 | 0.005 | 0.016 | 0.010 | 0.016 |
| rs2009416 | 5 | 92415111 | C | T | 0.012 | 0.002 | 0.004 | 0.013 | 0.013 | 0.017 | 0.002 | 0.017 |
| rs159032 | 5 | 94206202 | T | C | 0.013 | 0.002 | -0.003 | 0.014 | -0.005 | 0.019 | -0.003 | 0.018 |
| rs7713317 | 5 | 95716722 | G | A | 0.016 | 0.002 | 0.002 | 0.014 | 0.007 | 0.018 | -0.009 | 0.017 |
| rs4703019 | 5 | 102053558 | A | G | 0.010 | 0.002 | -0.029 | 0.013 | -0.008 | 0.016 | -0.042 | 0.016 |
| rs11739877 | 5 | 105876806 | T | C | 0.012 | 0.002 | 0.000 | 0.013 | 0.012 | 0.017 | -0.010 | 0.016 |
| rs13168288 | 5 | 106385815 | A | G | 0.013 | 0.002 | 0.006 | 0.015 | 0.008 | 0.019 | 0.006 | 0.019 |
| rs40067 | 5 | 107439012 | G | A | 0.027 | 0.002 | 0.022 | 0.016 | 0.027 | 0.021 | 0.007 | 0.021 |

| | | | | | | | | | | | | |
|---|---|---|---|---|---|---|---|---|---|---|---|---|
| rs9326846 | 5 | 111301546 | G | A | 0.011 | 0.002 | -0.014 | 0.013 | -0.012 | 0.017 | -0.007 | 0.017 |
| rs12109548 | 5 | 112452241 | T | C | 0.010 | 0.002 | 0.017 | 0.013 | 0.025 | 0.017 | 0.008 | 0.016 |
| rs1402025 | 5 | 113987898 | C | T | 0.012 | 0.002 | 0.016 | 0.015 | 0.010 | 0.019 | 0.024 | 0.019 |
| rs6594967 | 5 | 115710803 | T | C | 0.011 | 0.002 | -0.011 | 0.013 | 0.016 | 0.017 | -0.032 | 0.017 |
| rs351114 | 5 | 119368666 | T | C | 0.011 | 0.002 | 0.012 | 0.012 | 0.004 | 0.016 | 0.014 | 0.016 |
| rs1363695 | 5 | 130378027 | C | T | 0.013 | 0.002 | 0.055 | 0.015 | 0.093 | 0.020 | 0.025 | 0.019 |
| rs329122 | 5 | 133864599 | G | A | 0.013 | 0.002 | -0.034 | 0.013 | -0.036 | 0.016 | -0.026 | 0.016 |
| rs7716275 | 5 | 137631073 | G | T | 0.013 | 0.002 | 0.042 | 0.016 | 0.035 | 0.021 | 0.042 | 0.020 |
| rs13174863 | 5 | 139080745 | G | A | 0.019 | 0.002 | 0.007 | 0.018 | 0.018 | 0.023 | -0.015 | 0.023 |
| rs2074613 | 5 | 139714564 | T | C | 0.011 | 0.002 | 0.004 | 0.013 | -0.009 | 0.016 | 0.017 | 0.016 |
| rs2190788 | 5 | 144484261 | T | G | 0.014 | 0.002 | -0.001 | 0.013 | 0.008 | 0.017 | 0.001 | 0.017 |
| rs10066835 | 5 | 151254297 | T | C | 0.043 | 0.007 | 0.008 | 0.048 | -0.008 | 0.062 | 0.046 | 0.060 |
| rs294704 | 5 | 152519088 | G | T | 0.011 | 0.002 | -0.015 | 0.014 | -0.032 | 0.018 | 0.005 | 0.018 |
| rs7715256 | 5 | 153537893 | G | T | 0.017 | 0.002 | -0.010 | 0.013 | 0.022 | 0.016 | -0.035 | 0.016 |
| rs17056301 | 5 | 158271680 | C | T | 0.012 | 0.002 | -0.002 | 0.014 | 0.005 | 0.018 | -0.013 | 0.018 |
| rs248139 | 5 | 167352783 | A | G | 0.013 | 0.002 | -0.010 | 0.015 | -0.021 | 0.020 | -0.007 | 0.020 |
| rs7730898 | 5 | 170459675 | A | G | 0.017 | 0.002 | -0.018 | 0.014 | -0.007 | 0.018 | -0.023 | 0.018 |
| rs17695092 | 5 | 173337853 | T | G | 0.011 | 0.002 | 0.039 | 0.014 | 0.063 | 0.018 | 0.021 | 0.017 |
| rs6556301 | 5 | 176527577 | G | T | 0.011 | 0.002 | -0.001 | 0.013 | 0.001 | 0.017 | -0.006 | 0.017 |
| rs9463175 | 6 | 9510030 | C | T | 0.011 | 0.002 | -0.016 | 0.013 | -0.010 | 0.017 | -0.021 | 0.017 |
| rs2228213 | 6 | 12124855 | G | A | 0.014 | 0.002 | 0.011 | 0.013 | -0.001 | 0.017 | 0.026 | 0.016 |
| rs9367368 | 6 | 13189275 | T | C | 0.012 | 0.002 | 0.006 | 0.014 | 0.012 | 0.018 | 0.001 | 0.017 |
| rs17805532 | 6 | 18497348 | T | C | 0.023 | 0.004 | 0.011 | 0.027 | 0.032 | 0.034 | -0.010 | 0.034 |
| rs11753081 | 6 | 20705590 | T | G | 0.014 | 0.002 | -0.027 | 0.016 | -0.053 | 0.021 | -0.010 | 0.021 |
| rs7760082 | 6 | 21919387 | G | A | 0.012 | 0.002 | -0.015 | 0.013 | -0.023 | 0.017 | -0.008 | 0.017 |
| rs1511471 | 6 | 23868594 | A | G | 0.011 | 0.002 | -0.016 | 0.013 | -0.004 | 0.017 | -0.027 | 0.017 |
| rs3813680 | 6 | 24848086 | A | G | 0.014 | 0.002 | 0.005 | 0.017 | 0.025 | 0.023 | -0.007 | 0.022 |
| rs1150659 | 6 | 26100023 | G | A | 0.014 | 0.002 | -0.024 | 0.015 | -0.021 | 0.019 | -0.025 | 0.019 |
| rs4711122 | 6 | 26758479 | C | T | 0.013 | 0.002 | -0.022 | 0.015 | 0.005 | 0.019 | -0.039 | 0.018 |
| rs853681 | 6 | 28296650 | A | C | 0.014 | 0.002 | -0.011 | 0.017 | -0.031 | 0.022 | 0.005 | 0.022 |
| rs2523586 | 6 | 31327435 | T | G | 0.015 | 0.002 | 0.048 | 0.015 | 0.022 | 0.019 | 0.057 | 0.019 |
| rs498240 | 6 | 31892592 | G | A | 0.027 | 0.003 | 0.009 | 0.025 | -0.111 | 0.031 | 0.116 | 0.033 |
| rs6933607 | 6 | 33743997 | G | A | 0.016 | 0.002 | -0.007 | 0.015 | 0.007 | 0.019 | -0.025 | 0.019 |
| rs2744974 | 6 | 34579431 | T | C | 0.025 | 0.002 | -0.022 | 0.013 | -0.004 | 0.017 | -0.032 | 0.017 |
| rs732594 | 6 | 35206553 | C | A | 0.018 | 0.002 | 0.001 | 0.013 | -0.016 | 0.017 | 0.016 | 0.017 |
| rs17757975 | 6 | 38214150 | T | C | 0.014 | 0.002 | -0.005 | 0.018 | 0.002 | 0.023 | -0.013 | 0.022 |
| rs7748777 | 6 | 41133806 | A | G | 0.011 | 0.002 | -0.013 | 0.013 | 0.001 | 0.016 | -0.023 | 0.016 |
| rs6901756 | 6 | 41825590 | T | C | 0.015 | 0.003 | -0.024 | 0.019 | -0.010 | 0.024 | -0.044 | 0.024 |
| rs9349239 | 6 | 42676480 | A | G | 0.012 | 0.002 | -0.019 | 0.012 | -0.026 | 0.016 | -0.018 | 0.016 |
| rs998584 | 6 | 43757896 | C | A | 0.013 | 0.002 | 0.006 | 0.013 | 0.005 | 0.017 | -0.005 | 0.016 |
| rs6903387 | 6 | 46348834 | A | G | 0.013 | 0.002 | -0.022 | 0.013 | -0.016 | 0.016 | -0.019 | 0.016 |
| rs17665162 | 6 | 50275258 | C | T | 0.029 | 0.004 | 0.026 | 0.027 | 0.027 | 0.036 | 0.020 | 0.035 |
| rs987237 | 6 | 50803050 | G | A | 0.041 | 0.002 | 0.003 | 0.016 | 0.010 | 0.021 | 0.001 | 0.020 |
| rs2709671 | 6 | 51204404 | C | T | 0.012 | 0.002 | 0.003 | 0.012 | 0.012 | 0.016 | -0.005 | 0.016 |
| rs4711986 | 6 | 51789259 | A | G | 0.014 | 0.002 | -0.002 | 0.013 | -0.006 | 0.017 | -0.010 | 0.017 |
| rs9296723 | 6 | 53752062 | C | T | 0.011 | 0.002 | 0.003 | 0.013 | 0.012 | 0.017 | -0.003 | 0.016 |
| rs9475173 | 6 | 55013291 | A | G | 0.011 | 0.002 | -0.025 | 0.013 | -0.013 | 0.017 | -0.039 | 0.017 |
| rs1020548 | 6 | 56810539 | G | A | 0.013 | 0.002 | -0.023 | 0.016 | -0.034 | 0.021 | -0.002 | 0.021 |
| rs7742595 | 6 | 64161733 | T | C | 0.011 | 0.002 | 0.034 | 0.012 | 0.028 | 0.016 | 0.040 | 0.016 |
| rs2757763 | 6 | 70222480 | G | A | 0.012 | 0.002 | 0.023 | 0.014 | 0.027 | 0.018 | 0.025 | 0.018 |
| rs9688431 | 6 | 73922654 | T | C | 0.023 | 0.004 | 0.015 | 0.026 | 0.028 | 0.033 | 0.007 | 0.033 |

| | | | | | | | | | | | | |
|---|---|---|---|---|---|---|---|---|---|---|---|---|
| rs9294260 | 6 | 83433228 | A | G | 0.015 | 0.002 | -0.024 | 0.013 | -0.028 | 0.016 | -0.035 | 0.016 |
| rs1853639 | 6 | 87606842 | G | A | 0.011 | 0.002 | -0.001 | 0.013 | -0.015 | 0.017 | 0.007 | 0.017 |
| rs16882001 | 6 | 90322237 | G | A | 0.027 | 0.004 | -0.021 | 0.028 | -0.022 | 0.036 | -0.007 | 0.036 |
| rs7771567 | 6 | 97846571 | G | A | 0.015 | 0.002 | -0.010 | 0.013 | -0.036 | 0.017 | 0.008 | 0.017 |
| rs901630 | 6 | 98539519 | C | T | 0.015 | 0.002 | 0.005 | 0.013 | -0.004 | 0.016 | 0.002 | 0.016 |
| rs17789218 | 6 | 100600097 | C | T | 0.013 | 0.002 | 0.017 | 0.015 | 0.022 | 0.019 | 0.023 | 0.019 |
| rs13208757 | 6 | 101175014 | T | C | 0.011 | 0.002 | -0.014 | 0.012 | -0.023 | 0.016 | -0.014 | 0.016 |
| rs155510 | 6 | 104805862 | T | G | 0.017 | 0.002 | 0.009 | 0.016 | 0.005 | 0.020 | 0.013 | 0.020 |
| rs3800229 | 6 | 108996963 | T | G | 0.018 | 0.002 | 0.004 | 0.014 | -0.010 | 0.018 | 0.017 | 0.017 |
| rs9387640 | 6 | 119508871 | C | T | 0.012 | 0.002 | 0.001 | 0.013 | 0.006 | 0.017 | 0.000 | 0.016 |
| rs2357760 | 6 | 120213880 | A | G | 0.015 | 0.002 | -0.003 | 0.013 | 0.004 | 0.017 | -0.014 | 0.017 |
| rs1871329 | 6 | 124911534 | G | A | 0.013 | 0.002 | -0.005 | 0.015 | 0.012 | 0.019 | -0.017 | 0.019 |
| rs1159974 | 6 | 126090277 | C | T | 0.011 | 0.002 | 0.004 | 0.013 | 0.014 | 0.016 | -0.009 | 0.016 |
| rs6569648 | 6 | 130349119 | C | T | 0.013 | 0.002 | 0.010 | 0.015 | -0.006 | 0.019 | 0.029 | 0.019 |
| rs2246012 | 6 | 131898208 | C | T | 0.016 | 0.002 | 0.006 | 0.017 | 0.004 | 0.022 | 0.002 | 0.022 |
| rs13201877 | 6 | 137675541 | G | A | 0.015 | 0.002 | 0.001 | 0.018 | 0.011 | 0.024 | -0.015 | 0.023 |
| rs11754747 | 6 | 141494602 | T | C | 0.012 | 0.002 | -0.014 | 0.015 | -0.020 | 0.019 | -0.007 | 0.019 |
| rs765875 | 6 | 143185683 | C | T | 0.012 | 0.002 | -0.005 | 0.012 | 0.000 | 0.016 | -0.019 | 0.016 |
| rs2185027 | 6 | 153381622 | C | A | 0.014 | 0.002 | 0.006 | 0.014 | 0.004 | 0.018 | 0.009 | 0.017 |
| rs10499276 | 6 | 154309808 | T | C | 0.017 | 0.003 | 0.006 | 0.019 | 0.022 | 0.024 | -0.022 | 0.024 |
| rs9397928 | 6 | 156673243 | C | T | 0.012 | 0.002 | 0.008 | 0.014 | 0.004 | 0.018 | 0.003 | 0.018 |
| rs487060 | 6 | 160774459 | T | C | 0.011 | 0.002 | 0.004 | 0.012 | -0.007 | 0.016 | 0.012 | 0.016 |
| rs13191362 | 6 | 163033350 | A | G | 0.024 | 0.003 | -0.019 | 0.019 | -0.024 | 0.024 | -0.019 | 0.024 |
| rs9364687 | 6 | 163817911 | G | T | 0.011 | 0.002 | 0.009 | 0.013 | 0.027 | 0.016 | -0.014 | 0.016 |
| rs6461115 | 7 | 2103668 | A | G | 0.014 | 0.002 | 0.001 | 0.015 | 0.006 | 0.019 | -0.008 | 0.019 |
| rs4722398 | 7 | 3125220 | T | C | 0.016 | 0.003 | -0.006 | 0.018 | -0.043 | 0.024 | 0.024 | 0.023 |
| rs6463489 | 7 | 5542513 | T | C | 0.016 | 0.003 | -0.023 | 0.021 | -0.027 | 0.027 | -0.009 | 0.026 |
| rs7784465 | 7 | 6418275 | C | T | 0.016 | 0.003 | 0.006 | 0.018 | -0.009 | 0.024 | 0.019 | 0.023 |
| rs6968554 | 7 | 17287106 | G | A | 0.010 | 0.002 | 0.004 | 0.013 | 0.000 | 0.017 | 0.016 | 0.016 |
| rs10263780 | 7 | 19778086 | G | A | 0.016 | 0.003 | 0.020 | 0.018 | 0.013 | 0.023 | 0.029 | 0.023 |
| rs2158414 | 7 | 21491021 | T | C | 0.010 | 0.002 | -0.001 | 0.013 | -0.007 | 0.017 | 0.008 | 0.017 |
| rs4307239 | 7 | 24354300 | G | A | 0.012 | 0.002 | -0.004 | 0.013 | 0.006 | 0.016 | -0.008 | 0.016 |
| rs11971041 | 7 | 26698848 | G | A | 0.021 | 0.003 | 0.002 | 0.022 | 0.007 | 0.028 | -0.008 | 0.028 |
| rs4722672 | 7 | 27231762 | C | T | 0.015 | 0.002 | 0.083 | 0.016 | 0.054 | 0.021 | 0.108 | 0.021 |
| rs849135 | 7 | 28196413 | A | G | 0.011 | 0.002 | -0.036 | 0.012 | -0.069 | 0.016 | -0.003 | 0.016 |
| rs215634 | 7 | 32369148 | A | G | 0.015 | 0.002 | 0.005 | 0.013 | 0.002 | 0.016 | -0.005 | 0.016 |
| rs2237403 | 7 | 39448936 | C | T | 0.012 | 0.002 | -0.005 | 0.013 | 0.001 | 0.017 | -0.014 | 0.017 |
| rs2289379 | 7 | 44804225 | C | T | 0.014 | 0.002 | -0.041 | 0.013 | -0.042 | 0.017 | -0.032 | 0.016 |
| rs10269783 | 7 | 49616203 | A | G | 0.013 | 0.002 | 0.006 | 0.013 | 0.018 | 0.017 | -0.002 | 0.016 |
| rs3807566 | 7 | 50564204 | G | T | 0.013 | 0.002 | 0.012 | 0.012 | 0.003 | 0.016 | 0.021 | 0.016 |
| rs726372 | 7 | 69320268 | G | A | 0.011 | 0.002 | 0.003 | 0.013 | 0.007 | 0.017 | -0.005 | 0.017 |
| rs4718966 | 7 | 70040558 | T | C | 0.013 | 0.002 | -0.011 | 0.013 | -0.012 | 0.016 | -0.005 | 0.016 |
| rs4717623 | 7 | 71434369 | C | T | 0.014 | 0.002 | -0.005 | 0.014 | 0.007 | 0.018 | -0.009 | 0.018 |
| rs6964833 | 7 | 74101909 | C | T | 0.016 | 0.002 | -0.056 | 0.015 | -0.063 | 0.019 | -0.051 | 0.018 |
| rs17207196 | 7 | 75101065 | C | T | 0.022 | 0.002 | -0.040 | 0.013 | -0.035 | 0.017 | -0.052 | 0.017 |
| rs17685 | 7 | 75616105 | A | G | 0.011 | 0.002 | 0.010 | 0.014 | -0.008 | 0.018 | 0.024 | 0.018 |
| rs2245368 | 7 | 76608143 | C | T | 0.026 | 0.002 | 0.040 | 0.018 | 0.039 | 0.024 | 0.045 | 0.023 |
| rs740157 | 7 | 77055885 | A | G | 0.012 | 0.002 | -0.005 | 0.013 | -0.013 | 0.016 | 0.003 | 0.016 |
| rs1544459 | 7 | 77417584 | C | T | 0.010 | 0.002 | -0.008 | 0.012 | 0.004 | 0.016 | -0.021 | 0.016 |
| rs1852006 | 7 | 77829768 | G | A | 0.016 | 0.002 | -0.006 | 0.013 | 0.011 | 0.017 | -0.020 | 0.017 |
| rs6963840 | 7 | 78144371 | T | C | 0.015 | 0.002 | 0.001 | 0.017 | 0.041 | 0.022 | -0.036 | 0.022 |

| | | | | | | | | | | | | |
|---|---|---|---|---|---|---|---|---|---|---|---|---|
| rs10259665 | 7 | 79061837 | A | G | 0.011 | 0.002 | -0.027 | 0.013 | -0.010 | 0.017 | -0.041 | 0.017 |
| rs802460 | 7 | 86260816 | T | C | 0.011 | 0.002 | -0.015 | 0.013 | -0.022 | 0.017 | -0.013 | 0.017 |
| rs7780752 | 7 | 93241640 | C | T | 0.014 | 0.002 | -0.023 | 0.013 | -0.041 | 0.017 | -0.011 | 0.017 |
| rs13240600 | 7 | 99064466 | A | G | 0.020 | 0.002 | -0.015 | 0.017 | -0.009 | 0.022 | -0.024 | 0.022 |
| rs1048303 | 7 | 100804140 | C | T | 0.011 | 0.002 | -0.007 | 0.013 | 0.003 | 0.016 | -0.019 | 0.016 |
| rs11496125 | 7 | 103417557 | T | C | 0.017 | 0.002 | 0.022 | 0.013 | 0.005 | 0.016 | 0.029 | 0.016 |
| rs1524277 | 7 | 109173130 | C | T | 0.011 | 0.002 | 0.002 | 0.012 | 0.016 | 0.016 | -0.006 | 0.016 |
| rs7788008 | 7 | 112972483 | G | A | 0.016 | 0.002 | -0.009 | 0.013 | -0.043 | 0.016 | 0.024 | 0.016 |
| rs12705916 | 7 | 113492834 | C | T | 0.016 | 0.002 | 0.015 | 0.013 | 0.022 | 0.017 | 0.004 | 0.016 |
| rs2045293 | 7 | 114350102 | T | C | 0.014 | 0.002 | -0.030 | 0.013 | -0.037 | 0.016 | -0.023 | 0.016 |
| rs1899689 | 7 | 121964349 | T | C | 0.012 | 0.002 | -0.003 | 0.013 | 0.012 | 0.016 | -0.015 | 0.016 |
| rs2283093 | 7 | 126721231 | T | C | 0.013 | 0.002 | 0.010 | 0.015 | 0.017 | 0.020 | 0.000 | 0.020 |
| rs896183 | 7 | 127831580 | A | G | 0.010 | 0.002 | 0.018 | 0.013 | 0.026 | 0.017 | 0.004 | 0.017 |
| rs972283 | 7 | 130466854 | A | G | 0.010 | 0.002 | 0.027 | 0.013 | 0.025 | 0.016 | 0.035 | 0.016 |
| rs7802342 | 7 | 137435925 | G | T | 0.012 | 0.002 | -0.004 | 0.014 | -0.001 | 0.018 | -0.003 | 0.018 |
| rs7811342 | 7 | 138794618 | T | C | 0.020 | 0.003 | -0.027 | 0.020 | 0.016 | 0.027 | -0.052 | 0.026 |
| rs10243319 | 7 | 147674678 | T | C | 0.011 | 0.002 | -0.015 | 0.013 | -0.033 | 0.016 | -0.013 | 0.016 |
| rs2907948 | 7 | 150638484 | G | A | 0.014 | 0.002 | -0.005 | 0.015 | 0.009 | 0.019 | -0.017 | 0.018 |
| rs1658820 | 8 | 4288577 | T | G | 0.014 | 0.002 | 0.018 | 0.015 | 0.020 | 0.019 | 0.021 | 0.019 |
| rs7837587 | 8 | 8378992 | C | T | 0.016 | 0.002 | -0.001 | 0.012 | 0.009 | 0.016 | -0.009 | 0.016 |
| rs1045529 | 8 | 8890098 | T | C | 0.016 | 0.002 | 0.009 | 0.013 | 0.002 | 0.016 | 0.017 | 0.016 |
| rs615632 | 8 | 9796321 | C | T | 0.017 | 0.002 | 0.010 | 0.013 | 0.030 | 0.016 | -0.007 | 0.016 |
| rs6985109 | 8 | 10761585 | G | A | 0.018 | 0.002 | 0.016 | 0.012 | 0.038 | 0.016 | -0.010 | 0.016 |
| rs12549150 | 8 | 11422936 | T | C | 0.017 | 0.002 | 0.001 | 0.013 | 0.008 | 0.016 | -0.006 | 0.016 |
| rs13263601 | 8 | 14095900 | C | A | 0.015 | 0.002 | 0.013 | 0.013 | 0.019 | 0.017 | 0.007 | 0.017 |
| rs13278290 | 8 | 15560370 | T | C | 0.012 | 0.002 | -0.010 | 0.013 | -0.014 | 0.017 | -0.011 | 0.016 |
| rs2616192 | 8 | 20668624 | T | G | 0.013 | 0.002 | -0.005 | 0.013 | 0.008 | 0.017 | -0.014 | 0.017 |
| rs11781222 | 8 | 23389571 | T | C | 0.016 | 0.002 | 0.032 | 0.018 | -0.005 | 0.023 | 0.059 | 0.023 |
| rs11784576 | 8 | 25661397 | T | G | 0.011 | 0.002 | -0.023 | 0.014 | -0.014 | 0.017 | -0.036 | 0.017 |
| rs17446091 | 8 | 27167942 | C | T | 0.012 | 0.002 | 0.038 | 0.015 | 0.042 | 0.019 | 0.033 | 0.019 |
| rs1982441 | 8 | 28021769 | T | G | 0.018 | 0.003 | 0.041 | 0.018 | 0.061 | 0.024 | 0.022 | 0.023 |
| rs1421334 | 8 | 30865733 | A | C | 0.013 | 0.002 | 0.018 | 0.013 | 0.014 | 0.016 | 0.020 | 0.016 |
| rs7826312 | 8 | 32400115 | C | T | 0.010 | 0.002 | -0.019 | 0.013 | -0.025 | 0.016 | -0.027 | 0.016 |
| rs12545740 | 8 | 33455300 | A | C | 0.011 | 0.002 | 0.006 | 0.013 | 0.011 | 0.017 | 0.004 | 0.016 |
| rs7844647 | 8 | 34503776 | T | C | 0.012 | 0.002 | -0.001 | 0.014 | -0.012 | 0.018 | 0.010 | 0.018 |
| rs6471941 | 8 | 62117973 | A | G | 0.016 | 0.002 | -0.017 | 0.017 | -0.055 | 0.022 | 0.006 | 0.021 |
| rs705980 | 8 | 64727201 | C | T | 0.011 | 0.002 | 0.001 | 0.012 | 0.005 | 0.016 | -0.009 | 0.016 |
| rs16932761 | 8 | 67202787 | G | A | 0.014 | 0.002 | -0.002 | 0.014 | 0.013 | 0.018 | 0.001 | 0.018 |
| rs1431659 | 8 | 73439070 | A | G | 0.020 | 0.002 | -0.013 | 0.014 | -0.001 | 0.018 | -0.020 | 0.018 |
| rs2170382 | 8 | 74689288 | T | C | 0.017 | 0.003 | 0.008 | 0.020 | 0.022 | 0.025 | -0.014 | 0.025 |
| rs2926614 | 8 | 76301610 | C | T | 0.019 | 0.002 | -0.022 | 0.016 | 0.002 | 0.021 | -0.050 | 0.020 |
| rs17405819 | 8 | 76806584 | T | C | 0.022 | 0.002 | -0.003 | 0.014 | -0.002 | 0.017 | -0.013 | 0.017 |
| rs7010763 | 8 | 77342772 | G | A | 0.016 | 0.002 | 0.003 | 0.014 | 0.015 | 0.018 | -0.004 | 0.018 |
| rs16907751 | 8 | 81375457 | C | T | 0.021 | 0.003 | -0.009 | 0.021 | -0.009 | 0.027 | -0.024 | 0.026 |
| rs733594 | 8 | 85077686 | T | C | 0.014 | 0.002 | -0.018 | 0.014 | -0.040 | 0.018 | -0.003 | 0.018 |
| rs2467210 | 8 | 85679207 | G | A | 0.015 | 0.002 | 0.001 | 0.015 | -0.002 | 0.019 | 0.009 | 0.019 |
| rs7006629 | 8 | 87519542 | T | C | 0.011 | 0.002 | -0.021 | 0.012 | -0.023 | 0.016 | -0.024 | 0.016 |
| rs2174367 | 8 | 89408196 | G | T | 0.012 | 0.002 | -0.003 | 0.013 | -0.002 | 0.017 | 0.004 | 0.017 |
| rs1899898 | 8 | 93240419 | T | C | 0.011 | 0.002 | 0.022 | 0.013 | 0.028 | 0.017 | 0.025 | 0.017 |
| rs12680842 | 8 | 95582606 | A | G | 0.013 | 0.002 | 0.011 | 0.013 | -0.006 | 0.017 | 0.024 | 0.017 |
| rs3134358 | 8 | 101958433 | T | G | 0.013 | 0.002 | -0.008 | 0.013 | 0.000 | 0.016 | -0.008 | 0.016 |

| | | | | | | | | | | | | |
|---|---|---|---|---|---|---|---|---|---|---|---|---|
| rs13250058 | 8 | 112270826 | T | G | 0.011 | 0.002 | -0.015 | 0.013 | -0.013 | 0.017 | -0.026 | 0.017 |
| rs2694047 | 8 | 116750548 | G | A | 0.019 | 0.002 | -0.012 | 0.015 | -0.013 | 0.019 | -0.003 | 0.018 |
| rs17450772 | 8 | 118895712 | C | T | 0.019 | 0.003 | -0.009 | 0.020 | 0.008 | 0.025 | -0.026 | 0.025 |
| rs12682037 | 8 | 132880474 | C | T | 0.016 | 0.003 | 0.009 | 0.019 | 0.009 | 0.025 | 0.006 | 0.025 |
| rs16906845 | 8 | 138215228 | G | A | 0.023 | 0.004 | -0.027 | 0.026 | 0.002 | 0.034 | -0.057 | 0.033 |
| rs7357604 | 8 | 142629949 | G | A | 0.011 | 0.002 | -0.023 | 0.013 | -0.005 | 0.017 | -0.039 | 0.016 |
| rs4072917 | 8 | 143300279 | A | G | 0.012 | 0.002 | 0.032 | 0.013 | 0.030 | 0.016 | 0.031 | 0.016 |
| rs7042372 | 9 | 6959840 | A | G | 0.012 | 0.002 | -0.002 | 0.013 | -0.014 | 0.017 | 0.008 | 0.017 |
| rs1865341 | 9 | 8845911 | T | C | 0.013 | 0.002 | 0.002 | 0.015 | 0.011 | 0.019 | -0.005 | 0.018 |
| rs10118866 | 9 | 10119157 | T | G | 0.012 | 0.002 | 0.002 | 0.015 | -0.001 | 0.019 | 0.001 | 0.019 |
| rs1535660 | 9 | 10371073 | T | C | 0.015 | 0.003 | -0.010 | 0.018 | 0.003 | 0.023 | -0.029 | 0.023 |
| rs10959687 | 9 | 11280652 | A | G | 0.012 | 0.002 | 0.018 | 0.012 | 0.025 | 0.016 | 0.010 | 0.016 |
| rs17820822 | 9 | 11831420 | T | G | 0.014 | 0.002 | 0.004 | 0.013 | 0.009 | 0.017 | -0.002 | 0.016 |
| rs13298062 | 9 | 13941513 | A | G | 0.014 | 0.002 | -0.013 | 0.016 | -0.024 | 0.020 | -0.005 | 0.020 |
| rs7031064 | 9 | 14455076 | A | G | 0.011 | 0.002 | 0.021 | 0.012 | 0.029 | 0.016 | 0.008 | 0.016 |
| rs4740619 | 9 | 15634326 | T | C | 0.019 | 0.002 | 0.001 | 0.012 | -0.001 | 0.016 | 0.007 | 0.016 |
| rs10962549 | 9 | 16719445 | T | C | 0.020 | 0.002 | 0.012 | 0.016 | 0.011 | 0.021 | 0.011 | 0.021 |
| rs10811901 | 9 | 23356935 | A | G | 0.011 | 0.002 | 0.008 | 0.012 | 0.026 | 0.016 | -0.014 | 0.016 |
| rs7874154 | 9 | 27777012 | C | T | 0.013 | 0.002 | -0.023 | 0.012 | -0.023 | 0.016 | -0.024 | 0.016 |
| rs10968576 | 9 | 28414339 | G | A | 0.025 | 0.002 | 0.018 | 0.013 | 0.025 | 0.017 | 0.015 | 0.017 |
| rs16914051 | 9 | 28943416 | C | T | 0.015 | 0.002 | -0.013 | 0.017 | 0.000 | 0.022 | -0.031 | 0.022 |
| rs11792311 | 9 | 29672405 | G | A | 0.014 | 0.002 | 0.004 | 0.015 | -0.030 | 0.019 | 0.028 | 0.019 |
| rs17720922 | 9 | 31030917 | T | C | 0.013 | 0.002 | 0.000 | 0.016 | 0.001 | 0.020 | 0.002 | 0.020 |
| rs10971721 | 9 | 33827694 | C | T | 0.020 | 0.003 | -0.030 | 0.020 | -0.022 | 0.026 | -0.034 | 0.025 |
| rs13290794 | 9 | 37183628 | G | A | 0.014 | 0.002 | -0.003 | 0.013 | -0.008 | 0.017 | -0.001 | 0.016 |
| rs10746862 | 9 | 73780912 | G | T | 0.012 | 0.002 | 0.014 | 0.013 | 0.002 | 0.016 | 0.027 | 0.016 |
| rs1634350 | 9 | 81334684 | A | C | 0.012 | 0.002 | -0.002 | 0.013 | 0.012 | 0.017 | -0.015 | 0.016 |
| rs11138313 | 9 | 82240968 | A | G | 0.017 | 0.003 | -0.006 | 0.021 | -0.024 | 0.027 | -0.002 | 0.027 |
| rs2796456 | 9 | 84193487 | A | C | 0.012 | 0.002 | 0.024 | 0.014 | 0.026 | 0.018 | 0.018 | 0.018 |
| rs1187352 | 9 | 87293457 | C | T | 0.012 | 0.002 | 0.016 | 0.013 | 0.020 | 0.017 | 0.009 | 0.017 |
| rs12380880 | 9 | 88912765 | C | T | 0.013 | 0.002 | 0.008 | 0.015 | 0.006 | 0.019 | 0.017 | 0.019 |
| rs10797115 | 9 | 92191256 | T | C | 0.012 | 0.002 | -0.012 | 0.013 | -0.025 | 0.016 | 0.000 | 0.016 |
| rs7869771 | 9 | 94180627 | A | C | 0.014 | 0.002 | 0.026 | 0.014 | 0.012 | 0.018 | 0.025 | 0.018 |
| rs9650755 | 9 | 96484342 | G | A | 0.015 | 0.002 | -0.008 | 0.014 | -0.005 | 0.018 | -0.008 | 0.018 |
| rs420158 | 9 | 101477500 | C | T | 0.012 | 0.002 | 0.013 | 0.015 | 0.011 | 0.019 | 0.017 | 0.018 |
| rs4989244 | 9 | 102100348 | G | A | 0.011 | 0.002 | 0.005 | 0.013 | 0.023 | 0.016 | -0.015 | 0.016 |
| rs9299338 | 9 | 103108178 | G | A | 0.017 | 0.002 | 0.024 | 0.013 | 0.027 | 0.017 | 0.017 | 0.017 |
| rs10989568 | 9 | 104396304 | A | G | 0.011 | 0.002 | -0.010 | 0.012 | -0.009 | 0.016 | -0.005 | 0.016 |
| rs7024334 | 9 | 109072075 | T | G | 0.014 | 0.002 | -0.019 | 0.015 | -0.018 | 0.019 | -0.011 | 0.019 |
| rs6477694 | 9 | 111932342 | C | T | 0.012 | 0.002 | 0.022 | 0.013 | 0.033 | 0.017 | 0.014 | 0.017 |
| rs1928295 | 9 | 120378483 | T | C | 0.014 | 0.002 | -0.007 | 0.012 | 0.006 | 0.016 | -0.025 | 0.016 |
| rs1327808 | 9 | 120896390 | G | A | 0.013 | 0.002 | -0.039 | 0.016 | -0.038 | 0.021 | -0.028 | 0.021 |
| rs7865157 | 9 | 122631560 | T | C | 0.018 | 0.003 | -0.019 | 0.021 | -0.036 | 0.027 | 0.004 | 0.026 |
| rs10818810 | 9 | 126096522 | A | G | 0.013 | 0.002 | -0.014 | 0.013 | -0.013 | 0.017 | -0.008 | 0.016 |
| rs10818938 | 9 | 127049237 | A | G | 0.011 | 0.002 | -0.012 | 0.013 | -0.009 | 0.016 | -0.018 | 0.016 |
| rs13292976 | 9 | 129467340 | T | C | 0.013 | 0.002 | 0.003 | 0.013 | -0.008 | 0.016 | 0.005 | 0.016 |
| rs12005136 | 9 | 131059830 | C | A | 0.019 | 0.003 | -0.003 | 0.018 | -0.005 | 0.023 | 0.012 | 0.022 |
| rs4740383 | 9 | 133783566 | A | G | 0.013 | 0.002 | -0.004 | 0.013 | 0.004 | 0.017 | -0.012 | 0.016 |
| rs11792069 | 9 | 140646121 | A | G | 0.015 | 0.002 | -0.008 | 0.018 | 0.012 | 0.023 | -0.017 | 0.022 |
| rs11251352 | 10 | 2585792 | G | A | 0.011 | 0.002 | -0.009 | 0.013 | 0.001 | 0.016 | -0.020 | 0.016 |
| rs10795422 | 10 | 16759312 | G | A | 0.014 | 0.002 | 0.026 | 0.014 | 0.014 | 0.018 | 0.044 | 0.017 |

| | | | | | | | | | | | | |
|---|---|---|---|---|---|---|---|---|---|---|---|---|
| rs12768319 | 10 | 19812754 | C | T | 0.012 | 0.002 | 0.013 | 0.014 | 0.021 | 0.018 | 0.009 | 0.018 |
| rs7084454 | 10 | 21821274 | A | G | 0.019 | 0.002 | 0.021 | 0.013 | 0.011 | 0.017 | 0.016 | 0.017 |
| rs12762034 | 10 | 33969931 | C | T | 0.024 | 0.003 | 0.053 | 0.023 | 0.057 | 0.030 | 0.067 | 0.029 |
| rs2477017 | 10 | 34911879 | A | G | 0.010 | 0.002 | 0.013 | 0.013 | 0.039 | 0.017 | -0.003 | 0.016 |
| rs1937683 | 10 | 53679060 | T | C | 0.011 | 0.002 | 0.006 | 0.013 | 0.003 | 0.017 | 0.010 | 0.017 |
| rs6479905 | 10 | 65315231 | G | A | 0.013 | 0.002 | -0.021 | 0.012 | -0.021 | 0.016 | -0.012 | 0.016 |
| rs12098284 | 10 | 76047464 | T | C | 0.018 | 0.003 | 0.026 | 0.018 | 0.010 | 0.024 | 0.045 | 0.023 |
| rs4745794 | 10 | 77534000 | G | A | 0.011 | 0.002 | 0.007 | 0.013 | -0.017 | 0.016 | 0.026 | 0.016 |
| rs11001963 | 10 | 78760959 | T | C | 0.011 | 0.002 | 0.018 | 0.013 | 0.034 | 0.016 | 0.010 | 0.016 |
| rs7899106 | 10 | 87410904 | G | A | 0.033 | 0.004 | -0.029 | 0.029 | 0.011 | 0.037 | -0.075 | 0.037 |
| rs2114824 | 10 | 88119015 | G | A | 0.013 | 0.002 | -0.005 | 0.012 | -0.024 | 0.016 | 0.007 | 0.016 |
| rs1468069 | 10 | 98988759 | A | C | 0.010 | 0.002 | -0.009 | 0.013 | -0.008 | 0.017 | -0.012 | 0.017 |
| rs577525 | 10 | 99769388 | C | T | 0.017 | 0.002 | 0.023 | 0.013 | 0.053 | 0.016 | 0.006 | 0.016 |
| rs17094222 | 10 | 102395440 | C | T | 0.018 | 0.002 | 0.000 | 0.015 | -0.002 | 0.020 | 0.006 | 0.020 |
| rs9787495 | 10 | 103206115 | G | A | 0.010 | 0.002 | -0.012 | 0.013 | -0.002 | 0.016 | -0.020 | 0.016 |
| rs7083450 | 10 | 103984060 | T | C | 0.016 | 0.002 | 0.001 | 0.017 | 0.016 | 0.022 | -0.015 | 0.022 |
| rs12411886 | 10 | 104685299 | A | C | 0.027 | 0.003 | -0.013 | 0.022 | -0.015 | 0.029 | 0.001 | 0.029 |
| rs7903146 | 10 | 114758349 | C | T | 0.018 | 0.002 | 0.012 | 0.014 | 0.007 | 0.018 | 0.015 | 0.017 |
| rs10886017 | 10 | 118672531 | A | C | 0.015 | 0.002 | -0.038 | 0.015 | -0.041 | 0.019 | -0.039 | 0.018 |
| rs4523610 | 10 | 122069448 | C | T | 0.012 | 0.002 | -0.017 | 0.015 | -0.014 | 0.019 | -0.022 | 0.019 |
| rs845084 | 10 | 125220036 | A | G | 0.014 | 0.002 | 0.002 | 0.014 | 0.015 | 0.018 | 0.000 | 0.018 |
| rs17636031 | 10 | 126594078 | C | T | 0.016 | 0.002 | -0.016 | 0.015 | -0.028 | 0.019 | 0.004 | 0.019 |
| rs4880341 | 10 | 133992689 | C | T | 0.012 | 0.002 | -0.005 | 0.013 | -0.004 | 0.016 | -0.010 | 0.016 |
| rs4963120 | 11 | 825777 | T | C | 0.013 | 0.002 | 0.032 | 0.013 | 0.011 | 0.016 | 0.059 | 0.016 |
| rs10840606 | 11 | 2234690 | G | A | 0.016 | 0.002 | 0.047 | 0.016 | 0.057 | 0.021 | 0.035 | 0.021 |
| rs4929923 | 11 | 8639200 | C | T | 0.018 | 0.002 | 0.007 | 0.013 | 0.018 | 0.017 | -0.008 | 0.016 |
| rs1037587 | 11 | 11796727 | T | C | 0.011 | 0.002 | 0.028 | 0.013 | 0.036 | 0.016 | 0.018 | 0.016 |
| rs4757144 | 11 | 13331226 | A | G | 0.017 | 0.002 | 0.001 | 0.013 | -0.007 | 0.016 | 0.004 | 0.016 |
| rs1557765 | 11 | 17403639 | C | T | 0.012 | 0.002 | 0.028 | 0.013 | 0.043 | 0.016 | 0.010 | 0.016 |
| rs6265 | 11 | 27679916 | C | T | 0.041 | 0.002 | 0.008 | 0.016 | -0.026 | 0.020 | 0.030 | 0.020 |
| rs481554 | 11 | 28721187 | G | A | 0.011 | 0.002 | -0.027 | 0.013 | -0.022 | 0.017 | -0.037 | 0.017 |
| rs2467594 | 11 | 29224006 | G | A | 0.012 | 0.002 | 0.005 | 0.013 | 0.007 | 0.016 | -0.004 | 0.016 |
| rs2065418 | 11 | 30422068 | T | G | 0.017 | 0.002 | -0.005 | 0.013 | 0.002 | 0.017 | -0.011 | 0.016 |
| rs223051 | 11 | 32131303 | T | C | 0.011 | 0.002 | 0.018 | 0.013 | 0.038 | 0.017 | 0.003 | 0.017 |
| rs10768994 | 11 | 43936945 | T | C | 0.011 | 0.002 | -0.009 | 0.013 | -0.013 | 0.016 | 0.000 | 0.016 |
| rs10838465 | 11 | 45426141 | A | C | 0.014 | 0.002 | -0.013 | 0.013 | 0.013 | 0.017 | -0.044 | 0.017 |
| rs10160701 | 11 | 46551918 | T | C | 0.014 | 0.002 | 0.000 | 0.016 | 0.008 | 0.021 | 0.000 | 0.021 |
| rs7124681 | 11 | 47529947 | A | C | 0.026 | 0.002 | 0.016 | 0.013 | 0.026 | 0.016 | 0.012 | 0.016 |
| rs1483121 | 11 | 48333360 | G | A | 0.017 | 0.002 | -0.008 | 0.019 | -0.023 | 0.023 | 0.016 | 0.024 |
| rs7120873 | 11 | 49459474 | T | C | 0.017 | 0.003 | 0.000 | 0.020 | 0.011 | 0.026 | -0.003 | 0.025 |
| rs11228824 | 11 | 56634316 | G | A | 0.013 | 0.002 | 0.021 | 0.016 | 0.048 | 0.021 | -0.005 | 0.020 |
| rs7947143 | 11 | 64090422 | G | A | 0.018 | 0.002 | -0.024 | 0.017 | -0.035 | 0.022 | -0.009 | 0.022 |
| rs506338 | 11 | 64440920 | C | T | 0.013 | 0.002 | 0.004 | 0.014 | 0.000 | 0.018 | 0.006 | 0.017 |
| rs653264 | 11 | 68699775 | G | A | 0.010 | 0.002 | 0.002 | 0.012 | 0.006 | 0.016 | -0.001 | 0.016 |
| rs592483 | 11 | 69445173 | C | T | 0.015 | 0.002 | -0.007 | 0.013 | -0.003 | 0.017 | -0.012 | 0.016 |
| rs7123876 | 11 | 72444583 | C | T | 0.012 | 0.002 | 0.016 | 0.014 | 0.051 | 0.019 | -0.012 | 0.018 |
| rs1465900 | 11 | 76473138 | A | C | 0.013 | 0.002 | -0.011 | 0.015 | -0.009 | 0.019 | -0.016 | 0.019 |
| rs7117238 | 11 | 78040259 | G | A | 0.013 | 0.002 | -0.045 | 0.017 | -0.027 | 0.021 | -0.067 | 0.021 |
| rs349088 | 11 | 84814393 | C | A | 0.013 | 0.002 | 0.017 | 0.012 | 0.023 | 0.016 | 0.016 | 0.016 |
| rs3781815 | 11 | 85341933 | T | C | 0.013 | 0.002 | -0.001 | 0.016 | 0.016 | 0.020 | -0.014 | 0.020 |
| rs10830452 | 11 | 89966202 | G | A | 0.011 | 0.002 | -0.003 | 0.013 | -0.002 | 0.017 | -0.003 | 0.017 |

| | | | | | | | | | | | | |
|---|---|---|---|---|---|---|---|---|---|---|---|---|
| rs2605603 | 11 | 93221105 | G | A | 0.010 | 0.002 | -0.002 | 0.012 | 0.001 | 0.016 | -0.017 | 0.016 |
| rs2155645 | 11 | 112912947 | C | T | 0.012 | 0.002 | 0.016 | 0.014 | 0.027 | 0.018 | 0.005 | 0.018 |
| rs719802 | 11 | 113234679 | T | C | 0.010 | 0.002 | 0.001 | 0.013 | -0.009 | 0.017 | 0.000 | 0.016 |
| rs1048932 | 11 | 115044850 | C | A | 0.016 | 0.002 | 0.001 | 0.013 | 0.018 | 0.016 | -0.024 | 0.016 |
| rs12420725 | 11 | 117017530 | G | A | 0.023 | 0.004 | 0.041 | 0.027 | 0.032 | 0.034 | 0.051 | 0.034 |
| rs1786141 | 11 | 118938315 | T | C | 0.013 | 0.002 | 0.004 | 0.013 | 0.009 | 0.016 | 0.002 | 0.016 |
| rs7941030 | 11 | 122522375 | C | T | 0.011 | 0.002 | 0.010 | 0.013 | 0.018 | 0.016 | 0.010 | 0.016 |
| rs7925214 | 11 | 130794253 | T | C | 0.015 | 0.002 | -0.007 | 0.013 | 0.009 | 0.016 | -0.019 | 0.016 |
| rs2512885 | 11 | 131467794 | C | T | 0.013 | 0.002 | 0.022 | 0.012 | 0.020 | 0.016 | 0.020 | 0.016 |
| rs11222940 | 11 | 131985721 | C | A | 0.012 | 0.002 | -0.001 | 0.013 | -0.016 | 0.017 | 0.012 | 0.017 |
| rs4936175 | 11 | 132641959 | C | T | 0.012 | 0.002 | -0.020 | 0.013 | -0.017 | 0.016 | -0.019 | 0.016 |
| rs10894670 | 11 | 133221987 | C | A | 0.010 | 0.002 | 0.007 | 0.013 | 0.020 | 0.017 | -0.008 | 0.017 |
| rs329651 | 11 | 133767622 | T | G | 0.016 | 0.002 | 0.019 | 0.016 | 0.014 | 0.021 | 0.026 | 0.021 |
| rs12364470 | 11 | 134601012 | G | T | 0.018 | 0.002 | 0.023 | 0.017 | 0.025 | 0.022 | 0.028 | 0.022 |
| rs11611246 | 12 | 939480 | T | G | 0.024 | 0.002 | 0.033 | 0.015 | 0.037 | 0.020 | 0.034 | 0.020 |
| rs2429150 | 12 | 2152655 | C | A | 0.011 | 0.002 | -0.014 | 0.013 | -0.010 | 0.016 | -0.017 | 0.016 |
| rs10744146 | 12 | 17212881 | G | A | 0.012 | 0.002 | -0.011 | 0.012 | -0.013 | 0.016 | -0.017 | 0.016 |
| rs621042 | 12 | 18789007 | C | A | 0.011 | 0.002 | -0.006 | 0.013 | -0.004 | 0.016 | -0.016 | 0.016 |
| rs10841188 | 12 | 19379693 | G | A | 0.013 | 0.002 | 0.019 | 0.014 | 0.002 | 0.018 | 0.027 | 0.018 |
| rs1584121 | 12 | 23559163 | G | A | 0.013 | 0.002 | -0.024 | 0.016 | -0.029 | 0.021 | -0.022 | 0.020 |
| rs7970953 | 12 | 24075508 | A | G | 0.014 | 0.002 | -0.017 | 0.014 | -0.046 | 0.018 | 0.007 | 0.017 |
| rs11052536 | 12 | 33371849 | T | C | 0.017 | 0.002 | -0.016 | 0.018 | -0.042 | 0.024 | 0.006 | 0.023 |
| rs11170468 | 12 | 39430048 | A | C | 0.012 | 0.002 | -0.012 | 0.015 | 0.003 | 0.019 | -0.027 | 0.019 |
| rs2733287 | 12 | 41880909 | C | A | 0.016 | 0.002 | 0.008 | 0.012 | 0.029 | 0.016 | -0.025 | 0.016 |
| rs2269828 | 12 | 47471439 | G | A | 0.011 | 0.002 | 0.002 | 0.014 | 0.005 | 0.017 | -0.008 | 0.017 |
| rs7975791 | 12 | 49413486 | C | T | 0.026 | 0.004 | -0.059 | 0.037 | -0.094 | 0.046 | -0.021 | 0.049 |
| rs7138803 | 12 | 50247468 | A | G | 0.030 | 0.002 | 0.017 | 0.013 | 0.015 | 0.017 | 0.019 | 0.016 |
| rs4077093 | 12 | 51593616 | T | G | 0.013 | 0.002 | -0.002 | 0.015 | -0.020 | 0.020 | 0.018 | 0.019 |
| rs7134628 | 12 | 53785861 | A | G | 0.017 | 0.003 | 0.021 | 0.021 | 0.025 | 0.027 | 0.013 | 0.026 |
| rs4759075 | 12 | 54667285 | T | C | 0.011 | 0.002 | 0.022 | 0.013 | 0.036 | 0.017 | 0.012 | 0.016 |
| rs2271189 | 12 | 56494991 | G | A | 0.014 | 0.002 | 0.000 | 0.013 | -0.017 | 0.017 | 0.012 | 0.016 |
| rs774211 | 12 | 56920939 | T | C | 0.014 | 0.002 | -0.005 | 0.017 | -0.024 | 0.022 | 0.009 | 0.021 |
| rs11173522 | 12 | 60953472 | A | C | 0.013 | 0.002 | -0.012 | 0.015 | -0.009 | 0.020 | -0.016 | 0.019 |
| rs1819844 | 12 | 68205604 | A | G | 0.014 | 0.002 | 0.006 | 0.016 | 0.009 | 0.021 | 0.009 | 0.021 |
| rs10878946 | 12 | 69642315 | C | T | 0.014 | 0.002 | 0.025 | 0.014 | 0.044 | 0.018 | 0.015 | 0.018 |
| rs11115176 | 12 | 82465797 | T | C | 0.012 | 0.002 | 0.012 | 0.015 | 0.032 | 0.019 | -0.007 | 0.019 |
| rs12299814 | 12 | 90216146 | C | A | 0.016 | 0.002 | -0.011 | 0.014 | -0.024 | 0.019 | 0.000 | 0.018 |
| rs2579106 | 12 | 90628230 | C | T | 0.014 | 0.002 | 0.024 | 0.014 | 0.034 | 0.018 | 0.011 | 0.018 |
| rs11105842 | 12 | 91243910 | G | A | 0.011 | 0.002 | -0.014 | 0.013 | -0.025 | 0.017 | -0.003 | 0.016 |
| rs10745785 | 12 | 97586257 | C | T | 0.011 | 0.002 | -0.007 | 0.013 | -0.022 | 0.017 | 0.014 | 0.017 |
| rs1420341 | 12 | 97913785 | C | T | 0.014 | 0.002 | 0.000 | 0.017 | -0.012 | 0.022 | 0.010 | 0.021 |
| rs7488867 | 12 | 103699685 | C | T | 0.020 | 0.002 | -0.011 | 0.014 | -0.025 | 0.018 | 0.008 | 0.018 |
| rs12316047 | 12 | 108393845 | G | A | 0.017 | 0.002 | -0.018 | 0.015 | -0.003 | 0.019 | -0.041 | 0.019 |
| rs17608150 | 12 | 110046698 | T | C | 0.020 | 0.003 | -0.019 | 0.024 | -0.008 | 0.031 | -0.042 | 0.030 |
| rs4766500 | 12 | 110993791 | G | A | 0.013 | 0.002 | 0.006 | 0.013 | 0.022 | 0.017 | -0.009 | 0.016 |
| rs11065987 | 12 | 112072424 | A | G | 0.012 | 0.002 | -0.078 | 0.013 | -0.089 | 0.017 | -0.072 | 0.016 |
| rs11066188 | 12 | 112610714 | G | A | 0.012 | 0.002 | -0.087 | 0.013 | -0.098 | 0.017 | -0.083 | 0.017 |
| rs739742 | 12 | 113184739 | A | C | 0.012 | 0.002 | -0.069 | 0.013 | -0.085 | 0.017 | -0.054 | 0.017 |
| rs4766710 | 12 | 114437708 | A | G | 0.023 | 0.004 | 0.009 | 0.025 | 0.051 | 0.033 | -0.029 | 0.032 |
| rs17709991 | 12 | 116065057 | C | T | 0.011 | 0.002 | 0.017 | 0.014 | 0.003 | 0.018 | 0.028 | 0.018 |
| rs7973955 | 12 | 118409640 | G | A | 0.013 | 0.002 | -0.013 | 0.014 | -0.005 | 0.018 | -0.018 | 0.017 |

| | | | | | | | | | | | | |
|---|---|---|---|---|---|---|---|---|---|---|---|---|
| rs3887080 | 12 | 121661966 | A | G | 0.018 | 0.003 | -0.017 | 0.019 | 0.009 | 0.025 | -0.032 | 0.024 |
| rs1169091 | 12 | 122462486 | C | T | 0.011 | 0.002 | 0.028 | 0.014 | 0.011 | 0.018 | 0.045 | 0.018 |
| rs12369179 | 12 | 122963550 | C | T | 0.036 | 0.003 | 0.023 | 0.022 | 0.035 | 0.029 | 0.035 | 0.028 |
| rs7133378 | 12 | 124409502 | A | G | 0.013 | 0.002 | -0.038 | 0.013 | -0.030 | 0.017 | -0.040 | 0.017 |
| rs11614340 | 12 | 133426483 | C | T | 0.013 | 0.002 | 0.005 | 0.013 | 0.031 | 0.017 | -0.017 | 0.017 |
| rs9507983 | 13 | 28620036 | C | T | 0.016 | 0.002 | -0.003 | 0.013 | -0.025 | 0.016 | 0.007 | 0.016 |
| rs1045411 | 13 | 31033232 | C | T | 0.015 | 0.002 | 0.004 | 0.014 | 0.004 | 0.019 | 0.005 | 0.018 |
| rs7332115 | 13 | 33147548 | T | G | 0.016 | 0.002 | -0.011 | 0.013 | -0.027 | 0.017 | -0.005 | 0.016 |
| rs1336486 | 13 | 40784814 | G | T | 0.014 | 0.002 | 0.014 | 0.013 | 0.026 | 0.017 | 0.002 | 0.017 |
| rs12429545 | 13 | 54102206 | A | G | 0.032 | 0.003 | -0.002 | 0.019 | 0.019 | 0.024 | -0.023 | 0.024 |
| rs7995015 | 13 | 54828961 | T | G | 0.013 | 0.002 | -0.004 | 0.014 | 0.003 | 0.018 | -0.011 | 0.017 |
| rs9527455 | 13 | 56465597 | C | A | 0.012 | 0.002 | -0.003 | 0.015 | -0.013 | 0.019 | 0.006 | 0.019 |
| rs9569808 | 13 | 58631525 | A | G | 0.019 | 0.002 | 0.017 | 0.015 | 0.011 | 0.019 | 0.015 | 0.019 |
| rs9538141 | 13 | 59178258 | A | G | 0.016 | 0.002 | 0.007 | 0.013 | 0.005 | 0.016 | 0.005 | 0.016 |
| rs9317219 | 13 | 62686518 | G | A | 0.010 | 0.002 | 0.000 | 0.013 | -0.009 | 0.016 | 0.007 | 0.016 |
| rs1304070 | 13 | 65479449 | A | G | 0.013 | 0.002 | -0.021 | 0.015 | -0.020 | 0.019 | -0.017 | 0.018 |
| rs9540493 | 13 | 66205704 | A | G | 0.014 | 0.002 | -0.011 | 0.013 | 0.010 | 0.017 | -0.025 | 0.016 |
| rs9571687 | 13 | 67472713 | C | A | 0.013 | 0.002 | -0.009 | 0.013 | -0.028 | 0.017 | 0.009 | 0.017 |
| rs629443 | 13 | 76386075 | T | G | 0.012 | 0.002 | 0.009 | 0.015 | -0.011 | 0.019 | 0.014 | 0.018 |
| rs1668633 | 13 | 78371890 | T | C | 0.010 | 0.002 | -0.005 | 0.013 | 0.006 | 0.016 | -0.006 | 0.016 |
| rs9530843 | 13 | 79563749 | A | C | 0.013 | 0.002 | -0.014 | 0.013 | -0.009 | 0.016 | -0.024 | 0.016 |
| rs9547153 | 13 | 85903717 | G | A | 0.010 | 0.002 | -0.004 | 0.013 | 0.008 | 0.016 | -0.007 | 0.016 |
| rs7331467 | 13 | 86540789 | A | G | 0.013 | 0.002 | 0.006 | 0.013 | 0.009 | 0.017 | -0.003 | 0.016 |
| rs2198679 | 13 | 89131240 | A | G | 0.011 | 0.002 | -0.005 | 0.012 | -0.009 | 0.016 | -0.003 | 0.016 |
| rs9302083 | 13 | 96374386 | T | C | 0.011 | 0.002 | -0.016 | 0.012 | 0.002 | 0.016 | -0.030 | 0.016 |
| rs1927790 | 13 | 96922191 | C | T | 0.015 | 0.002 | -0.005 | 0.013 | 0.016 | 0.016 | -0.023 | 0.016 |
| rs9634445 | 13 | 98123032 | A | G | 0.011 | 0.002 | 0.007 | 0.014 | 0.011 | 0.018 | 0.003 | 0.017 |
| rs7334078 | 13 | 99120484 | T | C | 0.012 | 0.002 | 0.031 | 0.014 | 0.032 | 0.018 | 0.026 | 0.018 |
| rs9514131 | 13 | 104090848 | G | T | 0.015 | 0.003 | 0.009 | 0.019 | 0.011 | 0.025 | 0.007 | 0.025 |
| rs2479958 | 13 | 111984244 | A | G | 0.015 | 0.002 | 0.010 | 0.013 | 0.024 | 0.017 | -0.008 | 0.016 |
| rs10132280 | 14 | 25928179 | C | A | 0.022 | 0.002 | -0.021 | 0.014 | -0.020 | 0.018 | -0.027 | 0.017 |
| rs12885454 | 14 | 29736838 | C | A | 0.019 | 0.002 | -0.006 | 0.013 | 0.016 | 0.017 | -0.023 | 0.017 |
| rs10483389 | 14 | 30495719 | T | C | 0.034 | 0.004 | -0.040 | 0.032 | 0.006 | 0.041 | -0.067 | 0.041 |
| rs17522122 | 14 | 33302882 | T | G | 0.016 | 0.002 | -0.008 | 0.013 | -0.008 | 0.017 | -0.013 | 0.016 |
| rs7144747 | 14 | 35637511 | G | A | 0.016 | 0.003 | 0.043 | 0.019 | 0.021 | 0.024 | 0.067 | 0.024 |
| rs1956151 | 14 | 40101060 | G | A | 0.013 | 0.002 | 0.039 | 0.016 | 0.054 | 0.020 | 0.013 | 0.020 |
| rs1955540 | 14 | 40801751 | C | T | 0.016 | 0.002 | 0.007 | 0.016 | 0.004 | 0.021 | 0.011 | 0.020 |
| rs8007908 | 14 | 46838144 | G | A | 0.011 | 0.002 | -0.004 | 0.012 | -0.001 | 0.016 | 0.002 | 0.016 |
| rs3007105 | 14 | 47367616 | T | C | 0.014 | 0.002 | -0.009 | 0.013 | -0.005 | 0.016 | -0.001 | 0.016 |
| rs217671 | 14 | 62360464 | G | A | 0.014 | 0.002 | -0.003 | 0.014 | -0.017 | 0.018 | 0.000 | 0.018 |
| rs4430672 | 14 | 63094407 | T | C | 0.013 | 0.002 | -0.016 | 0.016 | -0.006 | 0.020 | -0.032 | 0.020 |
| rs3902951 | 14 | 69789755 | G | T | 0.013 | 0.002 | -0.011 | 0.015 | 0.000 | 0.019 | -0.025 | 0.019 |
| rs1007934 | 14 | 73463479 | G | A | 0.012 | 0.002 | -0.009 | 0.013 | -0.012 | 0.016 | -0.003 | 0.016 |
| rs17105272 | 14 | 77529783 | T | C | 0.011 | 0.002 | -0.020 | 0.013 | -0.029 | 0.018 | -0.006 | 0.017 |
| rs10146527 | 14 | 79499850 | T | C | 0.014 | 0.002 | -0.002 | 0.013 | 0.011 | 0.017 | -0.003 | 0.016 |
| rs7144011 | 14 | 79940383 | T | G | 0.028 | 0.002 | 0.037 | 0.015 | 0.015 | 0.019 | 0.049 | 0.019 |
| rs12888545 | 14 | 88308044 | G | A | 0.014 | 0.002 | 0.056 | 0.015 | 0.095 | 0.019 | 0.024 | 0.018 |
| rs1951455 | 14 | 91512339 | C | T | 0.015 | 0.002 | -0.009 | 0.014 | -0.001 | 0.018 | -0.021 | 0.018 |
| rs9989141 | 14 | 94006257 | T | C | 0.016 | 0.002 | 0.003 | 0.013 | 0.003 | 0.017 | -0.001 | 0.016 |
| rs17096510 | 14 | 98616999 | A | C | 0.015 | 0.003 | -0.031 | 0.018 | -0.014 | 0.023 | -0.034 | 0.023 |
| rs4082793 | 14 | 99700080 | C | T | 0.012 | 0.002 | 0.002 | 0.013 | -0.005 | 0.016 | 0.008 | 0.016 |

| | | | | | | | | | | | | |
|---|---|---|---|---|---|---|---|---|---|---|---|---|
| rs12147845 | 14 | 101144596 | T | C | 0.020 | 0.003 | 0.036 | 0.020 | 0.011 | 0.025 | 0.057 | 0.025 |
| rs7147503 | 14 | 101539384 | C | T | 0.012 | 0.002 | 0.034 | 0.013 | 0.029 | 0.017 | 0.033 | 0.017 |
| rs10431745 | 14 | 102302372 | A | G | 0.020 | 0.003 | -0.004 | 0.025 | 0.010 | 0.033 | -0.036 | 0.031 |
| rs8016771 | 14 | 102649451 | G | T | 0.019 | 0.003 | -0.020 | 0.022 | -0.004 | 0.029 | -0.029 | 0.029 |
| rs3803286 | 14 | 103246470 | A | G | 0.018 | 0.002 | 0.017 | 0.013 | 0.006 | 0.017 | 0.036 | 0.017 |
| rs2010281 | 14 | 103862322 | G | A | 0.016 | 0.002 | 0.053 | 0.013 | 0.081 | 0.017 | 0.034 | 0.017 |
| rs4906908 | 15 | 27040082 | G | T | 0.010 | 0.002 | -0.019 | 0.013 | -0.019 | 0.016 | -0.017 | 0.016 |
| rs7172627 | 15 | 31877690 | G | A | 0.012 | 0.002 | 0.025 | 0.012 | 0.029 | 0.016 | 0.024 | 0.016 |
| rs8036040 | 15 | 36402716 | A | C | 0.011 | 0.002 | 0.006 | 0.012 | 0.014 | 0.016 | 0.005 | 0.016 |
| rs7178205 | 15 | 41971213 | A | G | 0.014 | 0.002 | -0.012 | 0.016 | 0.007 | 0.021 | -0.042 | 0.021 |
| rs12439798 | 15 | 46584787 | T | G | 0.013 | 0.002 | -0.004 | 0.012 | 0.007 | 0.016 | -0.011 | 0.016 |
| rs12899905 | 15 | 47142090 | C | T | 0.011 | 0.002 | -0.021 | 0.014 | -0.029 | 0.018 | -0.012 | 0.018 |
| rs1912631 | 15 | 47914691 | G | A | 0.012 | 0.002 | -0.032 | 0.013 | -0.026 | 0.016 | -0.040 | 0.016 |
| rs3736485 | 15 | 51748610 | A | G | 0.013 | 0.002 | 0.011 | 0.013 | 0.014 | 0.016 | 0.009 | 0.016 |
| rs2414128 | 15 | 52369728 | T | C | 0.012 | 0.002 | 0.023 | 0.013 | 0.020 | 0.017 | 0.024 | 0.016 |
| rs16965225 | 15 | 53143170 | T | G | 0.022 | 0.003 | 0.007 | 0.025 | -0.011 | 0.032 | 0.018 | 0.032 |
| rs10163018 | 15 | 53525415 | C | T | 0.011 | 0.002 | 0.006 | 0.013 | 0.011 | 0.017 | 0.001 | 0.016 |
| rs17236194 | 15 | 59002755 | C | T | 0.015 | 0.002 | 0.018 | 0.018 | 0.015 | 0.024 | 0.017 | 0.023 |
| rs340025 | 15 | 60908307 | C | T | 0.012 | 0.002 | 0.014 | 0.013 | 0.032 | 0.016 | 0.006 | 0.016 |
| rs7162238 | 15 | 61447827 | A | G | 0.011 | 0.002 | 0.021 | 0.013 | 0.016 | 0.016 | 0.031 | 0.016 |
| rs12595158 | 15 | 62316035 | C | T | 0.039 | 0.005 | 0.090 | 0.045 | 0.063 | 0.059 | 0.086 | 0.057 |
| rs11635675 | 15 | 63793238 | T | G | 0.012 | 0.002 | -0.028 | 0.013 | -0.029 | 0.017 | -0.023 | 0.017 |
| rs17200912 | 15 | 66730307 | C | T | 0.015 | 0.002 | -0.008 | 0.015 | -0.001 | 0.019 | -0.004 | 0.019 |
| rs13329567 | 15 | 68104367 | C | T | 0.029 | 0.002 | 0.001 | 0.015 | 0.006 | 0.019 | -0.001 | 0.019 |
| rs7164727 | 15 | 73093991 | T | C | 0.018 | 0.002 | -0.017 | 0.013 | -0.004 | 0.017 | -0.027 | 0.017 |
| rs11630240 | 15 | 73645403 | G | A | 0.024 | 0.003 | 0.003 | 0.024 | 0.018 | 0.031 | -0.016 | 0.030 |
| rs5742914 | 15 | 74286929 | T | C | 0.018 | 0.003 | -0.016 | 0.020 | -0.010 | 0.025 | -0.021 | 0.025 |
| rs936227 | 15 | 75131959 | G | A | 0.012 | 0.002 | 0.008 | 0.013 | 0.011 | 0.016 | 0.012 | 0.016 |
| rs1829130 | 15 | 76107730 | C | T | 0.011 | 0.002 | 0.007 | 0.013 | 0.007 | 0.017 | 0.007 | 0.017 |
| rs11855853 | 15 | 78012618 | C | T | 0.015 | 0.002 | -0.013 | 0.014 | 0.002 | 0.018 | -0.021 | 0.018 |
| rs12595749 | 15 | 79432359 | A | G | 0.014 | 0.002 | -0.009 | 0.013 | -0.025 | 0.016 | 0.011 | 0.016 |
| rs12593036 | 15 | 81058652 | A | G | 0.015 | 0.002 | 0.014 | 0.014 | 0.022 | 0.018 | 0.010 | 0.017 |
| rs12906208 | 15 | 92571691 | G | A | 0.013 | 0.002 | 0.015 | 0.015 | 0.048 | 0.020 | -0.011 | 0.019 |
| rs7181498 | 15 | 95271404 | T | C | 0.016 | 0.002 | 0.011 | 0.013 | 0.001 | 0.017 | 0.023 | 0.016 |
| rs1021985 | 15 | 98285658 | G | A | 0.011 | 0.002 | -0.026 | 0.013 | -0.030 | 0.017 | -0.025 | 0.016 |
| rs2715423 | 15 | 99511873 | G | A | 0.012 | 0.002 | -0.019 | 0.014 | -0.035 | 0.018 | 0.002 | 0.018 |
| rs11866815 | 16 | 387867 | C | T | 0.016 | 0.002 | -0.003 | 0.014 | -0.006 | 0.019 | -0.011 | 0.018 |
| rs2516739 | 16 | 2097158 | G | A | 0.016 | 0.002 | 0.015 | 0.016 | 0.048 | 0.020 | -0.009 | 0.020 |
| rs12448257 | 16 | 3599655 | A | G | 0.018 | 0.002 | -0.026 | 0.015 | 0.004 | 0.020 | -0.046 | 0.020 |
| rs879620 | 16 | 4015729 | T | C | 0.023 | 0.002 | -0.032 | 0.013 | -0.038 | 0.017 | -0.029 | 0.017 |
| rs1876359 | 16 | 4930100 | T | C | 0.013 | 0.002 | 0.024 | 0.013 | 0.032 | 0.017 | 0.008 | 0.016 |
| rs12149756 | 16 | 6728603 | G | A | 0.013 | 0.002 | -0.022 | 0.014 | -0.030 | 0.019 | -0.023 | 0.018 |
| rs249292 | 16 | 9412804 | T | C | 0.012 | 0.002 | 0.014 | 0.013 | -0.001 | 0.017 | 0.022 | 0.017 |
| rs1990573 | 16 | 9713688 | G | A | 0.013 | 0.002 | -0.017 | 0.013 | 0.002 | 0.017 | -0.035 | 0.017 |
| rs4985155 | 16 | 15129459 | A | G | 0.012 | 0.002 | -0.054 | 0.013 | -0.044 | 0.017 | -0.070 | 0.017 |
| rs12446632 | 16 | 19935389 | G | A | 0.035 | 0.002 | 0.021 | 0.018 | 0.037 | 0.023 | 0.023 | 0.022 |
| rs9931967 | 16 | 20375351 | T | G | 0.016 | 0.002 | -0.013 | 0.013 | 0.002 | 0.016 | -0.025 | 0.016 |
| rs2023671 | 16 | 23849839 | G | T | 0.012 | 0.002 | -0.062 | 0.014 | -0.046 | 0.019 | -0.079 | 0.018 |
| rs7195386 | 16 | 24578458 | T | C | 0.013 | 0.002 | -0.027 | 0.012 | -0.038 | 0.016 | -0.024 | 0.016 |
| rs2726039 | 16 | 28327463 | G | A | 0.019 | 0.002 | 0.063 | 0.013 | 0.071 | 0.017 | 0.049 | 0.017 |
| rs7498665 | 16 | 28883241 | G | A | 0.027 | 0.002 | 0.097 | 0.013 | 0.115 | 0.017 | 0.073 | 0.016 |

| | | | | | | | | | | | | | |
|---|---|---|---|---|---|---|---|---|---|---|---|---|---|
| | rs3814883 | 16 | 29994922 | T | C | 0.023 | 0.002 | -0.011 | 0.013 | -0.016 | 0.017 | -0.006 | 0.016 |
| | rs1557672 | 16 | 30510571 | C | T | 0.011 | 0.002 | 0.032 | 0.014 | 0.016 | 0.018 | 0.048 | 0.018 |
| | rs4889606 | 16 | 31011183 | A | G | 0.020 | 0.002 | 0.002 | 0.013 | 0.002 | 0.017 | -0.004 | 0.016 |
| | rs2080454 | 16 | 49062590 | C | A | 0.013 | 0.002 | -0.010 | 0.013 | -0.004 | 0.017 | -0.017 | 0.016 |
| | rs10852521 | 16 | 53804965 | C | T | 0.064 | 0.002 | 0.017 | 0.012 | 0.031 | 0.016 | 0.003 | 0.016 |
| | rs12448738 | 16 | 56489343 | C | A | 0.017 | 0.003 | -0.008 | 0.018 | 0.003 | 0.023 | -0.025 | 0.023 |
| | rs11075489 | 16 | 62803841 | C | T | 0.011 | 0.002 | 0.011 | 0.012 | 0.016 | 0.016 | 0.012 | 0.016 |
| | rs7200919 | 16 | 67316600 | A | G | 0.010 | 0.002 | 0.028 | 0.013 | 0.025 | 0.017 | 0.029 | 0.016 |
| | rs2307022 | 16 | 68381978 | A | G | 0.014 | 0.002 | -0.010 | 0.013 | 0.017 | 0.017 | -0.023 | 0.017 |
| | rs244418 | 16 | 69622762 | G | A | 0.021 | 0.002 | 0.019 | 0.013 | 0.021 | 0.016 | 0.018 | 0.016 |
| | rs7919 | 16 | 70514828 | C | A | 0.016 | 0.002 | 0.036 | 0.013 | 0.053 | 0.016 | 0.022 | 0.016 |
| | rs11643673 | 16 | 71353719 | A | G | 0.011 | 0.002 | 0.015 | 0.013 | -0.006 | 0.017 | 0.032 | 0.016 |
| | rs811054 | 16 | 72251132 | T | C | 0.014 | 0.002 | -0.015 | 0.013 | -0.030 | 0.016 | 0.000 | 0.016 |
| | rs756717 | 16 | 72996162 | G | A | 0.015 | 0.002 | -0.020 | 0.013 | -0.017 | 0.017 | -0.027 | 0.017 |
| | rs1125804 | 16 | 74148932 | G | A | 0.011 | 0.002 | 0.011 | 0.013 | 0.007 | 0.016 | 0.006 | 0.016 |
| | rs6564360 | 16 | 76779612 | G | A | 0.014 | 0.002 | 0.007 | 0.016 | 0.007 | 0.020 | -0.005 | 0.020 |
| | rs2012502 | 16 | 81728081 | A | C | 0.011 | 0.002 | 0.022 | 0.013 | 0.027 | 0.017 | 0.010 | 0.017 |
| | rs11150463 | 16 | 82452519 | C | A | 0.012 | 0.002 | -0.001 | 0.013 | 0.029 | 0.017 | -0.025 | 0.016 |
| | rs10492861 | 16 | 82866767 | T | G | 0.012 | 0.002 | 0.060 | 0.014 | 0.106 | 0.018 | 0.008 | 0.018 |
| | rs4516268 | 17 | 1846831 | C | A | 0.022 | 0.002 | -0.018 | 0.017 | 0.012 | 0.021 | -0.045 | 0.021 |
| | rs9904177 | 17 | 4755424 | G | A | 0.012 | 0.002 | -0.024 | 0.014 | 0.002 | 0.018 | -0.052 | 0.018 |
| | rs1000940 | 17 | 5283252 | G | A | 0.015 | 0.002 | -0.008 | 0.014 | 0.015 | 0.017 | -0.032 | 0.017 |
| | rs17681708 | 17 | 9792872 | C | T | 0.011 | 0.002 | 0.011 | 0.013 | -0.005 | 0.017 | 0.024 | 0.017 |
| | rs1075901 | 17 | 15943910 | C | T | 0.012 | 0.002 | 0.009 | 0.012 | 0.002 | 0.016 | 0.010 | 0.016 |
| | rs4986044 | 17 | 21261560 | C | T | 0.016 | 0.002 | 0.011 | 0.013 | 0.021 | 0.017 | 0.005 | 0.016 |
| | rs11080090 | 17 | 27502029 | G | A | 0.013 | 0.002 | 0.005 | 0.018 | -0.012 | 0.023 | 0.018 | 0.023 |
| | rs1038088 | 17 | 28074563 | G | T | 0.012 | 0.002 | 0.014 | 0.012 | -0.010 | 0.016 | 0.032 | 0.016 |
| | rs3930349 | 17 | 31475545 | C | A | 0.014 | 0.002 | -0.017 | 0.015 | -0.026 | 0.019 | -0.017 | 0.019 |
| | rs12150665 | 17 | 34914787 | T | C | 0.016 | 0.002 | 0.033 | 0.013 | 0.015 | 0.016 | 0.049 | 0.016 |
| | rs8070454 | 17 | 38160754 | C | T | 0.010 | 0.002 | 0.073 | 0.013 | 0.072 | 0.017 | 0.079 | 0.016 |
| | rs16966801 | 17 | 39573713 | G | A | 0.016 | 0.002 | 0.007 | 0.016 | 0.009 | 0.020 | 0.019 | 0.020 |
| | rs17599948 | 17 | 41353410 | A | G | 0.013 | 0.002 | -0.007 | 0.017 | 0.003 | 0.022 | -0.015 | 0.021 |
| | rs9901595 | 17 | 42305699 | A | G | 0.012 | 0.002 | -0.043 | 0.013 | -0.071 | 0.017 | -0.019 | 0.017 |
| | rs208015 | 17 | 46252346 | T | C | 0.036 | 0.003 | 0.004 | 0.024 | -0.035 | 0.032 | 0.033 | 0.031 |
| | rs11079849 | 17 | 47090785 | C | T | 0.019 | 0.002 | 0.000 | 0.013 | 0.008 | 0.017 | -0.004 | 0.017 |
| | rs8071182 | 17 | 55336155 | A | G | 0.013 | 0.002 | -0.008 | 0.017 | 0.033 | 0.021 | -0.027 | 0.022 |
| | rs11649864 | 17 | 56093061 | A | G | 0.018 | 0.003 | -0.055 | 0.022 | -0.070 | 0.030 | -0.040 | 0.028 |
| | rs8075273 | 17 | 61728881 | C | A | 0.013 | 0.002 | -0.017 | 0.014 | -0.001 | 0.018 | -0.038 | 0.017 |
| | rs12602912 | 17 | 65870073 | T | C | 0.018 | 0.002 | -0.028 | 0.016 | -0.005 | 0.020 | -0.049 | 0.020 |
| | rs2619976 | 17 | 71754545 | T | C | 0.010 | 0.002 | 0.019 | 0.013 | 0.020 | 0.017 | 0.024 | 0.016 |
| | rs7209235 | 17 | 73759552 | G | A | 0.011 | 0.002 | -0.049 | 0.014 | -0.062 | 0.018 | -0.042 | 0.018 |
| | rs8081039 | 17 | 75995829 | T | C | 0.023 | 0.004 | 0.039 | 0.026 | 0.014 | 0.034 | 0.049 | 0.034 |
| | rs1696757 | 17 | 77800743 | A | G | 0.010 | 0.002 | -0.011 | 0.013 | -0.010 | 0.016 | -0.011 | 0.016 |
| | rs12939549 | 17 | 78611724 | A | G | 0.018 | 0.002 | 0.003 | 0.012 | 0.005 | 0.016 | 0.000 | 0.016 |
| | rs7210438 | 17 | 79060180 | T | C | 0.014 | 0.002 | -0.014 | 0.015 | -0.021 | 0.019 | -0.007 | 0.019 |
| | rs11658335 | 17 | 80084821 | C | T | 0.010 | 0.002 | -0.009 | 0.013 | -0.010 | 0.016 | -0.005 | 0.016 |
| | rs1608445 | 18 | 947954 | G | A | 0.010 | 0.002 | -0.002 | 0.013 | 0.008 | 0.016 | -0.011 | 0.016 |
| | rs8097544 | 18 | 1839564 | G | A | 0.020 | 0.003 | -0.028 | 0.018 | -0.056 | 0.023 | -0.002 | 0.022 |
| | rs12964689 | 18 | 21116998 | A | G | 0.020 | 0.002 | 0.019 | 0.012 | 0.030 | 0.016 | 0.007 | 0.016 |
| | rs16940823 | 18 | 22137319 | C | A | 0.015 | 0.002 | -0.014 | 0.016 | -0.006 | 0.021 | -0.021 | 0.021 |
| | rs1941697 | 18 | 31251276 | A | G | 0.012 | 0.002 | 0.009 | 0.012 | 0.022 | 0.016 | -0.008 | 0.016 |

| | | | | | | | | | | | | |
|---|---|---|---|---|---|---|---|---|---|---|---|---|
| rs1365466 | 18 | 36182440 | C | T | 0.014 | 0.002 | 0.013 | 0.014 | 0.014 | 0.018 | 0.012 | 0.018 |
| rs559231 | 18 | 39644247 | T | G | 0.014 | 0.002 | 0.004 | 0.013 | -0.002 | 0.017 | 0.006 | 0.016 |
| rs7239883 | 18 | 40147671 | G | A | 0.013 | 0.002 | 0.016 | 0.013 | 0.014 | 0.016 | 0.008 | 0.016 |
| rs1158805 | 18 | 40736590 | C | A | 0.014 | 0.002 | -0.010 | 0.013 | -0.029 | 0.016 | -0.005 | 0.016 |
| rs954018 | 18 | 42598463 | G | A | 0.013 | 0.002 | -0.009 | 0.014 | -0.025 | 0.017 | 0.007 | 0.017 |
| rs10438964 | 18 | 42950629 | C | T | 0.013 | 0.002 | -0.006 | 0.014 | -0.033 | 0.018 | 0.019 | 0.018 |
| rs7239114 | 18 | 45921214 | A | G | 0.012 | 0.002 | -0.014 | 0.013 | -0.035 | 0.017 | -0.007 | 0.017 |
| rs9964756 | 18 | 50441817 | G | T | 0.016 | 0.003 | 0.018 | 0.020 | 0.004 | 0.026 | 0.030 | 0.026 |
| rs12607207 | 18 | 51560823 | C | T | 0.012 | 0.002 | -0.028 | 0.014 | -0.017 | 0.018 | -0.041 | 0.018 |
| rs8092503 | 18 | 52479487 | G | A | 0.017 | 0.002 | -0.027 | 0.015 | -0.015 | 0.019 | -0.028 | 0.019 |
| rs7243357 | 18 | 56883319 | T | G | 0.019 | 0.002 | -0.044 | 0.016 | -0.079 | 0.021 | -0.020 | 0.021 |
| rs663129 | 18 | 57838401 | A | G | 0.055 | 0.002 | 0.005 | 0.015 | 0.031 | 0.019 | -0.012 | 0.019 |
| rs8096899 | 18 | 58094046 | G | T | 0.040 | 0.004 | -0.041 | 0.031 | -0.013 | 0.040 | -0.068 | 0.040 |
| rs1426284 | 18 | 58738938 | A | G | 0.037 | 0.005 | -0.039 | 0.035 | -0.020 | 0.046 | -0.067 | 0.045 |
| rs7235205 | 18 | 63373762 | G | A | 0.014 | 0.002 | 0.014 | 0.014 | 0.016 | 0.018 | 0.006 | 0.018 |
| rs12604935 | 18 | 69233570 | C | A | 0.011 | 0.002 | 0.027 | 0.013 | 0.030 | 0.017 | 0.033 | 0.016 |
| rs11150911 | 18 | 73498528 | A | C | 0.013 | 0.002 | 0.012 | 0.014 | -0.007 | 0.018 | 0.026 | 0.017 |
| rs673429 | 18 | 76743413 | T | C | 0.024 | 0.004 | 0.015 | 0.027 | -0.009 | 0.035 | 0.042 | 0.034 |
| rs3957285 | 19 | 1891992 | A | G | 0.014 | 0.002 | -0.010 | 0.013 | -0.016 | 0.017 | -0.007 | 0.016 |
| rs350832 | 19 | 4069426 | A | G | 0.018 | 0.002 | -0.047 | 0.015 | -0.076 | 0.020 | -0.026 | 0.020 |
| rs243387 | 19 | 4445156 | G | A | 0.010 | 0.002 | -0.004 | 0.012 | -0.013 | 0.016 | 0.004 | 0.016 |
| rs12609744 | 19 | 12994140 | C | T | 0.013 | 0.002 | -0.029 | 0.014 | -0.022 | 0.018 | -0.038 | 0.018 |
| rs17724992 | 19 | 18454825 | A | G | 0.018 | 0.002 | 0.017 | 0.014 | 0.026 | 0.018 | 0.022 | 0.018 |
| rs709679 | 19 | 18726856 | T | C | 0.010 | 0.002 | 0.001 | 0.013 | 0.005 | 0.016 | 0.003 | 0.016 |
| rs998732 | 19 | 19378671 | A | G | 0.017 | 0.002 | -0.043 | 0.017 | -0.037 | 0.021 | -0.064 | 0.021 |
| rs17513613 | 19 | 30286822 | C | T | 0.019 | 0.002 | -0.006 | 0.013 | 0.000 | 0.017 | -0.020 | 0.017 |
| rs11084553 | 19 | 31019780 | A | G | 0.021 | 0.002 | -0.018 | 0.018 | 0.006 | 0.023 | -0.037 | 0.022 |
| rs10408013 | 19 | 33963766 | T | C | 0.011 | 0.002 | 0.011 | 0.014 | 0.031 | 0.018 | -0.007 | 0.017 |
| rs29941 | 19 | 34309532 | G | A | 0.015 | 0.002 | 0.003 | 0.013 | 0.004 | 0.017 | 0.004 | 0.017 |
| rs3826705 | 19 | 42637232 | C | T | 0.016 | 0.003 | 0.028 | 0.019 | 0.019 | 0.025 | 0.043 | 0.024 |
| rs2075650 | 19 | 45395619 | A | G | 0.024 | 0.002 | -0.020 | 0.019 | -0.017 | 0.024 | -0.008 | 0.024 |
| rs11672660 | 19 | 46180184 | C | T | 0.034 | 0.002 | 0.019 | 0.016 | 0.021 | 0.021 | 0.027 | 0.020 |
| rs3810291 | 19 | 47569003 | A | G | 0.027 | 0.002 | -0.025 | 0.013 | 0.006 | 0.017 | -0.060 | 0.017 |
| rs11670142 | 19 | 49650471 | G | T | 0.011 | 0.002 | 0.014 | 0.013 | 0.021 | 0.016 | 0.015 | 0.016 |
| rs4802778 | 19 | 51812034 | G | A | 0.012 | 0.002 | -0.014 | 0.013 | -0.029 | 0.017 | -0.005 | 0.016 |
| rs1884389 | 20 | 1410582 | C | T | 0.010 | 0.002 | 0.012 | 0.013 | 0.012 | 0.016 | 0.005 | 0.016 |
| rs4813619 | 20 | 2815715 | G | T | 0.011 | 0.002 | -0.014 | 0.012 | -0.012 | 0.016 | -0.017 | 0.016 |
| rs1884897 | 20 | 6612832 | G | A | 0.019 | 0.002 | 0.017 | 0.013 | 0.019 | 0.016 | 0.011 | 0.016 |
| rs2719771 | 20 | 8313607 | C | A | 0.012 | 0.002 | -0.009 | 0.014 | -0.008 | 0.018 | -0.002 | 0.018 |
| rs2876248 | 20 | 12700157 | T | C | 0.011 | 0.002 | 0.006 | 0.013 | 0.011 | 0.017 | -0.005 | 0.017 |
| rs17272434 | 20 | 15085365 | G | A | 0.011 | 0.002 | 0.019 | 0.013 | 0.005 | 0.017 | 0.027 | 0.017 |
| rs8123881 | 20 | 15819495 | G | A | 0.020 | 0.002 | -0.015 | 0.019 | -0.014 | 0.024 | -0.021 | 0.024 |
| rs4814512 | 20 | 16564210 | A | C | 0.013 | 0.002 | -0.017 | 0.015 | -0.011 | 0.019 | -0.029 | 0.019 |
| rs852056 | 20 | 17102860 | T | C | 0.013 | 0.002 | -0.024 | 0.014 | -0.003 | 0.019 | -0.043 | 0.018 |
| rs1409818 | 20 | 21381121 | T | C | 0.020 | 0.003 | 0.008 | 0.020 | -0.015 | 0.027 | 0.033 | 0.026 |
| rs6050446 | 20 | 25195509 | G | A | 0.034 | 0.005 | 0.110 | 0.036 | 0.182 | 0.048 | 0.026 | 0.045 |
| rs7260957 | 20 | 25722462 | C | T | 0.013 | 0.002 | -0.015 | 0.016 | 0.017 | 0.021 | -0.035 | 0.021 |
| rs3813922 | 20 | 30777336 | T | G | 0.014 | 0.002 | 0.011 | 0.017 | 0.030 | 0.022 | -0.013 | 0.022 |
| rs4012234 | 20 | 32553047 | G | T | 0.014 | 0.002 | 0.002 | 0.013 | -0.011 | 0.016 | 0.012 | 0.016 |
| rs4911442 | 20 | 33355046 | A | G | 0.015 | 0.002 | 0.000 | 0.019 | 0.001 | 0.025 | -0.012 | 0.024 |
| rs1474976 | 20 | 34732372 | A | C | 0.017 | 0.003 | 0.012 | 0.022 | 0.038 | 0.028 | -0.011 | 0.028 |

| | | | | | | | | | | | | | |
|---|---|---|---|---|---|---|---|---|---|---|---|---|---|
| | rs4812405 | 20 | 35276585 | C | A | 0.020 | 0.003 | -0.007 | 0.025 | -0.012 | 0.032 | -0.020 | 0.031 |
| | rs2143253 | 20 | 41987392 | G | A | 0.019 | 0.003 | 0.025 | 0.019 | 0.037 | 0.024 | 0.024 | 0.024 |
| | rs2868202 | 20 | 43428832 | C | T | 0.011 | 0.002 | 0.024 | 0.014 | 0.017 | 0.018 | 0.026 | 0.018 |
| | rs2425840 | 20 | 44904838 | C | A | 0.012 | 0.002 | 0.021 | 0.013 | 0.026 | 0.016 | 0.012 | 0.016 |
| | rs6019482 | 20 | 47495560 | C | T | 0.018 | 0.002 | 0.000 | 0.017 | 0.040 | 0.022 | -0.027 | 0.022 |
| | rs2143624 | 20 | 49781718 | A | G | 0.010 | 0.002 | 0.025 | 0.013 | 0.018 | 0.017 | 0.032 | 0.016 |
| | rs17806379 | 20 | 51107290 | C | T | 0.026 | 0.002 | 0.014 | 0.016 | 0.025 | 0.021 | 0.001 | 0.021 |
| | rs6023633 | 20 | 53453850 | G | A | 0.014 | 0.002 | -0.011 | 0.015 | -0.014 | 0.019 | -0.013 | 0.019 |
| | rs6014523 | 20 | 54394975 | T | C | 0.015 | 0.002 | -0.014 | 0.016 | -0.037 | 0.020 | 0.007 | 0.020 |
| | rs2427453 | 20 | 61577515 | T | C | 0.011 | 0.002 | -0.023 | 0.013 | -0.021 | 0.016 | -0.029 | 0.016 |
| | rs310618 | 20 | 62127121 | C | T | 0.011 | 0.002 | 0.030 | 0.014 | 0.049 | 0.018 | 0.013 | 0.018 |
| | rs6090041 | 20 | 62712676 | A | G | 0.012 | 0.002 | 0.019 | 0.014 | 0.001 | 0.018 | 0.033 | 0.018 |
| | rs2832283 | 21 | 30690558 | A | G | 0.012 | 0.002 | 0.019 | 0.015 | 0.029 | 0.020 | 0.007 | 0.019 |
| | rs762147 | 21 | 39238610 | G | A | 0.012 | 0.002 | -0.007 | 0.014 | 0.016 | 0.018 | -0.032 | 0.018 |
| | rs2836754 | 21 | 40291740 | C | T | 0.014 | 0.002 | -0.057 | 0.013 | -0.060 | 0.017 | -0.055 | 0.016 |
| | rs2836961 | 21 | 40627020 | C | A | 0.010 | 0.002 | 0.001 | 0.013 | 0.004 | 0.017 | -0.001 | 0.016 |
| | rs1964927 | 21 | 42653237 | G | A | 0.012 | 0.002 | 0.019 | 0.013 | 0.050 | 0.017 | -0.002 | 0.017 |
| | rs427943 | 21 | 46570896 | C | A | 0.017 | 0.002 | 0.022 | 0.013 | 0.019 | 0.016 | 0.018 | 0.016 |
| | rs11538 | 22 | 18220831 | G | A | 0.014 | 0.002 | -0.012 | 0.017 | 0.006 | 0.022 | -0.025 | 0.021 |
| | rs175165 | 22 | 20116015 | T | G | 0.010 | 0.002 | -0.013 | 0.013 | -0.009 | 0.017 | -0.025 | 0.016 |
| | rs12628891 | 22 | 38317137 | C | T | 0.011 | 0.002 | 0.003 | 0.014 | -0.014 | 0.018 | 0.018 | 0.017 |
| | rs11089885 | 22 | 38809485 | C | T | 0.010 | 0.002 | -0.004 | 0.013 | -0.007 | 0.016 | 0.001 | 0.016 |
| | rs738140 | 22 | 41884954 | A | G | 0.014 | 0.002 | 0.015 | 0.014 | 0.032 | 0.017 | -0.001 | 0.017 |
| | rs5751239 | 22 | 42592239 | T | C | 0.011 | 0.002 | 0.020 | 0.012 | 0.023 | 0.016 | 0.015 | 0.016 |
| Waist-to-hip ratio | rs9615905 | 22 | 48875699 | T | C | 0.011 | 0.002 | 0.045 | 0.013 | 0.026 | 0.016 | 0.052 | 0.016 |
| | rs7525092 | 1 | 1810090 | C | T | 0.012 | 0.002 | -0.043 | 0.014 | -0.035 | 0.018 | -0.043 | 0.018 |
| | rs2742690 | 1 | 2987268 | A | C | 0.017 | 0.003 | 0.022 | 0.017 | 0.017 | 0.021 | 0.006 | 0.021 |
| | rs6688233 | 1 | 9335745 | T | C | 0.019 | 0.002 | -0.022 | 0.015 | -0.031 | 0.019 | -0.024 | 0.019 |
| | rs12024554 | 1 | 19925759 | C | T | 0.015 | 0.002 | 0.000 | 0.015 | -0.002 | 0.019 | 0.003 | 0.019 |
| | rs2298632 | 1 | 23710475 | C | T | 0.014 | 0.002 | 0.006 | 0.013 | 0.001 | 0.017 | 0.017 | 0.016 |
| | rs213637 | 1 | 26188625 | T | G | 0.013 | 0.002 | -0.010 | 0.013 | -0.002 | 0.016 | -0.010 | 0.016 |
| | rs4660808 | 1 | 40018509 | T | C | 0.015 | 0.002 | 0.027 | 0.015 | 0.050 | 0.019 | -0.001 | 0.019 |
| | rs41286710 | 1 | 46383020 | C | T | 0.026 | 0.005 | -0.027 | 0.031 | -0.011 | 0.040 | -0.039 | 0.039 |
| | rs7531656 | 1 | 49828663 | A | G | 0.016 | 0.002 | -0.050 | 0.013 | -0.044 | 0.017 | -0.054 | 0.017 |
| | rs4593876 | 1 | 50389102 | T | C | 0.016 | 0.002 | -0.049 | 0.013 | -0.043 | 0.017 | -0.054 | 0.017 |
| | rs2481665 | 1 | 62594677 | T | C | 0.011 | 0.002 | -0.006 | 0.013 | 0.011 | 0.016 | -0.017 | 0.016 |
| | rs11208660 | 1 | 65983626 | T | C | 0.023 | 0.003 | 0.004 | 0.022 | -0.009 | 0.029 | 0.021 | 0.028 |
| | rs2815749 | 1 | 72814783 | G | A | 0.017 | 0.002 | 0.019 | 0.016 | 0.014 | 0.021 | 0.008 | 0.020 |
| | rs313732 | 1 | 86258603 | G | A | 0.012 | 0.002 | 0.005 | 0.013 | 0.018 | 0.016 | -0.001 | 0.016 |
| | rs6699397 | 1 | 91212216 | G | A | 0.012 | 0.002 | 0.017 | 0.013 | 0.012 | 0.017 | 0.016 | 0.016 |
| | rs9659380 | 1 | 98423149 | G | A | 0.018 | 0.002 | -0.014 | 0.017 | -0.008 | 0.022 | -0.013 | 0.022 |
| | rs2889351 | 1 | 103543532 | T | C | 0.015 | 0.002 | -0.005 | 0.013 | 0.002 | 0.016 | -0.010 | 0.016 |
| | rs2335077 | 1 | 107573565 | G | A | 0.012 | 0.002 | -0.047 | 0.013 | -0.028 | 0.017 | -0.056 | 0.017 |
| | rs17024393 | 1 | 110154688 | C | T | 0.030 | 0.005 | -0.057 | 0.039 | -0.058 | 0.050 | -0.055 | 0.049 |
| | rs6658723 | 1 | 112274162 | T | C | 0.015 | 0.002 | -0.006 | 0.013 | -0.010 | 0.017 | -0.003 | 0.016 |
| | rs3789615 | 1 | 114941326 | C | T | 0.013 | 0.002 | 0.006 | 0.013 | 0.001 | 0.016 | 0.015 | 0.016 |
| | rs10923724 | 1 | 119546842 | T | C | 0.024 | 0.002 | -0.013 | 0.013 | -0.007 | 0.016 | -0.023 | 0.016 |
| | rs905938 | 1 | 154991389 | T | C | 0.013 | 0.002 | -0.037 | 0.014 | -0.036 | 0.019 | -0.037 | 0.018 |
| | rs61813324 | 1 | 156049877 | T | C | 0.019 | 0.003 | -0.012 | 0.020 | -0.014 | 0.026 | -0.013 | 0.026 |
| | rs1535572 | 1 | 163649901 | C | T | 0.012 | 0.002 | 0.005 | 0.013 | 0.015 | 0.016 | -0.010 | 0.016 |
| | rs10919388 | 1 | 170372503 | C | A | 0.027 | 0.002 | -0.021 | 0.014 | -0.020 | 0.018 | -0.016 | 0.018 |

| | | | | | | | | | | | | |
|---|---|---|---|---|---|---|---|---|---|---|---|---|
| rs2301453 | 1 | 172358167 | G | A | 0.022 | 0.002 | 0.003 | 0.013 | 0.002 | 0.016 | 0.010 | 0.016 |
| rs28680958 | 1 | 173848808 | G | A | 0.014 | 0.002 | 0.006 | 0.015 | -0.006 | 0.019 | 0.011 | 0.019 |
| rs543874 | 1 | 177889480 | G | A | 0.020 | 0.002 | -0.027 | 0.016 | -0.004 | 0.020 | -0.045 | 0.020 |
| rs3828112 | 1 | 200042031 | T | C | 0.012 | 0.002 | 0.009 | 0.014 | 0.034 | 0.017 | -0.014 | 0.017 |
| rs1572993 | 1 | 205045087 | A | G | 0.013 | 0.002 | 0.012 | 0.013 | 0.020 | 0.016 | -0.001 | 0.016 |
| rs17015701 | 1 | 210337691 | A | G | 0.012 | 0.002 | 0.015 | 0.015 | -0.017 | 0.020 | 0.043 | 0.020 |
| rs3767848 | 1 | 214173840 | G | A | 0.012 | 0.002 | -0.030 | 0.014 | -0.041 | 0.019 | -0.022 | 0.018 |
| rs1563355 | 1 | 219653101 | C | T | 0.028 | 0.002 | -0.022 | 0.013 | -0.006 | 0.017 | -0.036 | 0.017 |
| rs6604731 | 1 | 224051439 | C | T | 0.012 | 0.002 | 0.021 | 0.014 | 0.032 | 0.018 | 0.009 | 0.017 |
| rs12042959 | 1 | 243533273 | A | G | 0.016 | 0.003 | -0.019 | 0.018 | 0.003 | 0.023 | -0.020 | 0.023 |
| rs6743060 | 2 | 629510 | A | C | 0.027 | 0.002 | -0.019 | 0.016 | -0.010 | 0.021 | -0.024 | 0.021 |
| rs10495563 | 2 | 9662210 | A | G | 0.012 | 0.002 | 0.032 | 0.013 | 0.014 | 0.017 | 0.038 | 0.017 |
| rs711869 | 2 | 13073967 | G | A | 0.017 | 0.002 | 0.001 | 0.013 | 0.016 | 0.016 | -0.011 | 0.016 |
| rs28445639 | 2 | 25182488 | T | C | 0.022 | 0.002 | 0.099 | 0.015 | 0.127 | 0.020 | 0.075 | 0.019 |
| rs2384463 | 2 | 26942256 | A | G | 0.011 | 0.002 | 0.004 | 0.012 | -0.016 | 0.016 | 0.019 | 0.016 |
| rs10153926 | 2 | 43189120 | A | G | 0.014 | 0.002 | -0.013 | 0.016 | 0.006 | 0.021 | -0.037 | 0.020 |
| rs7591387 | 2 | 43756032 | T | C | 0.018 | 0.003 | 0.103 | 0.020 | 0.145 | 0.025 | 0.057 | 0.025 |
| rs17326656 | 2 | 48962291 | T | G | 0.015 | 0.002 | -0.003 | 0.015 | 0.005 | 0.019 | -0.005 | 0.019 |
| rs929641 | 2 | 58792377 | A | G | 0.013 | 0.002 | 0.021 | 0.013 | 0.029 | 0.016 | 0.015 | 0.016 |
| rs6545714 | 2 | 59307725 | G | A | 0.014 | 0.002 | 0.006 | 0.013 | 0.016 | 0.016 | 0.005 | 0.016 |
| rs13028903 | 2 | 59951465 | T | C | 0.011 | 0.002 | -0.017 | 0.013 | -0.026 | 0.016 | -0.006 | 0.016 |
| rs2195086 | 2 | 60814466 | G | T | 0.014 | 0.002 | -0.020 | 0.018 | -0.034 | 0.023 | -0.003 | 0.022 |
| rs13391573 | 2 | 66237388 | T | C | 0.021 | 0.002 | -0.023 | 0.016 | -0.040 | 0.021 | -0.002 | 0.021 |
| rs11897119 | 2 | 66772000 | C | T | 0.013 | 0.002 | 0.017 | 0.013 | 0.015 | 0.016 | 0.011 | 0.016 |
| rs7598832 | 2 | 67853013 | C | T | 0.019 | 0.002 | 0.029 | 0.013 | 0.041 | 0.017 | 0.035 | 0.017 |
| rs6751316 | 2 | 100365061 | T | C | 0.018 | 0.003 | 0.012 | 0.018 | -0.002 | 0.024 | 0.023 | 0.023 |
| rs6542924 | 2 | 100893113 | C | A | 0.015 | 0.002 | -0.005 | 0.013 | -0.029 | 0.017 | 0.013 | 0.017 |
| rs4851057 | 2 | 105939237 | T | C | 0.016 | 0.003 | 0.015 | 0.019 | 0.013 | 0.024 | 0.023 | 0.024 |
| rs1345203 | 2 | 112253851 | T | C | 0.019 | 0.002 | 0.026 | 0.018 | 0.008 | 0.023 | 0.025 | 0.023 |
| rs4849294 | 2 | 114619997 | T | C | 0.012 | 0.002 | 0.015 | 0.013 | 0.007 | 0.016 | 0.017 | 0.016 |
| rs332105 | 2 | 119444229 | G | A | 0.014 | 0.002 | 0.005 | 0.013 | 0.003 | 0.016 | 0.001 | 0.016 |
| rs55920843 | 2 | 158412701 | T | G | 0.062 | 0.009 | -0.035 | 0.062 | -0.103 | 0.079 | 0.020 | 0.079 |
| rs1020731 | 2 | 161144055 | A | G | 0.013 | 0.002 | -0.032 | 0.014 | -0.035 | 0.017 | -0.022 | 0.017 |
| rs12621633 | 2 | 162859436 | A | G | 0.054 | 0.009 | 0.072 | 0.059 | 0.065 | 0.078 | 0.129 | 0.075 |
| rs10207936 | 2 | 164893801 | C | T | 0.013 | 0.002 | -0.015 | 0.015 | -0.006 | 0.020 | -0.017 | 0.019 |
| rs10195252 | 2 | 165513091 | T | C | 0.023 | 0.002 | -0.007 | 0.013 | -0.019 | 0.016 | -0.002 | 0.016 |
| rs12469667 | 2 | 166162705 | G | A | 0.013 | 0.002 | -0.025 | 0.015 | -0.021 | 0.019 | -0.027 | 0.018 |
| rs6433219 | 2 | 171421125 | A | G | 0.014 | 0.002 | -0.013 | 0.015 | -0.003 | 0.019 | -0.028 | 0.019 |
| rs2216931 | 2 | 181599070 | A | C | 0.012 | 0.002 | 0.013 | 0.013 | 0.020 | 0.017 | 0.006 | 0.017 |
| rs1569135 | 2 | 188115398 | A | G | 0.022 | 0.002 | 0.005 | 0.012 | 0.015 | 0.016 | -0.001 | 0.016 |
| rs1124639 | 2 | 200775744 | C | T | 0.011 | 0.002 | -0.022 | 0.012 | -0.005 | 0.016 | -0.035 | 0.016 |
| rs4673616 | 2 | 212281381 | G | T | 0.012 | 0.002 | 0.031 | 0.014 | 0.014 | 0.018 | 0.043 | 0.017 |
| rs7599312 | 2 | 213413231 | G | A | 0.011 | 0.002 | -0.022 | 0.014 | -0.018 | 0.018 | -0.017 | 0.018 |
| rs1017698 | 2 | 219170525 | G | A | 0.011 | 0.002 | 0.066 | 0.013 | 0.059 | 0.016 | 0.064 | 0.016 |
| rs7561798 | 2 | 228973660 | G | A | 0.010 | 0.002 | -0.009 | 0.012 | 0.007 | 0.016 | -0.024 | 0.016 |
| rs3796072 | 2 | 230667539 | C | T | 0.012 | 0.002 | -0.008 | 0.013 | -0.009 | 0.017 | -0.004 | 0.017 |
| rs3891424 | 2 | 239365456 | G | A | 0.028 | 0.004 | -0.015 | 0.029 | -0.046 | 0.038 | 0.014 | 0.037 |
| rs4686340 | 3 | 9345218 | A | C | 0.012 | 0.002 | 0.006 | 0.015 | -0.002 | 0.019 | 0.004 | 0.019 |
| rs2595004 | 3 | 11406721 | C | T | 0.016 | 0.002 | -0.009 | 0.016 | 0.019 | 0.021 | -0.035 | 0.021 |
| rs9872031 | 3 | 12496461 | A | G | 0.020 | 0.002 | -0.006 | 0.013 | -0.001 | 0.016 | -0.012 | 0.016 |
| rs2455848 | 3 | 15771372 | T | C | 0.013 | 0.002 | -0.004 | 0.013 | -0.019 | 0.017 | 0.011 | 0.017 |

| | | | | | | | | | | | | | |
|---|---|---|---|---|---|---|---|---|---|---|---|---|---|
| rs6442644 | 3 | 16852853 | G | T | 0.011 | 0.002 | 0.007 | 0.013 | 0.022 | 0.017 | -0.022 | 0.017 |
| rs4635727 | 3 | 18705035 | A | G | 0.012 | 0.002 | 0.037 | 0.014 | 0.046 | 0.018 | 0.043 | 0.018 |
| rs7616762 | 3 | 33869679 | G | A | 0.012 | 0.002 | 0.027 | 0.014 | 0.008 | 0.018 | 0.047 | 0.018 |
| rs11129657 | 3 | 35636709 | C | T | 0.018 | 0.002 | -0.005 | 0.015 | 0.012 | 0.020 | -0.023 | 0.020 |
| rs155524 | 3 | 37562141 | A | G | 0.013 | 0.002 | -0.005 | 0.013 | 0.014 | 0.016 | -0.020 | 0.016 |
| rs13092573 | 3 | 46988561 | T | C | 0.014 | 0.002 | 0.037 | 0.014 | 0.032 | 0.018 | 0.039 | 0.017 |
| rs2267846 | 3 | 48556339 | G | A | 0.014 | 0.002 | 0.050 | 0.014 | 0.021 | 0.018 | 0.083 | 0.018 |
| rs13316065 | 3 | 49884913 | T | C | 0.017 | 0.002 | -0.088 | 0.014 | -0.126 | 0.018 | -0.057 | 0.017 |
| rs12637870 | 3 | 50506073 | C | A | 0.017 | 0.003 | 0.036 | 0.017 | 0.053 | 0.022 | 0.005 | 0.021 |
| rs13080929 | 3 | 52885112 | A | C | 0.018 | 0.002 | -0.022 | 0.017 | -0.020 | 0.022 | -0.031 | 0.022 |
| rs1452075 | 3 | 62481063 | T | C | 0.012 | 0.002 | -0.014 | 0.014 | -0.001 | 0.018 | -0.027 | 0.018 |
| rs4132228 | 3 | 64708114 | C | T | 0.027 | 0.002 | -0.005 | 0.014 | 0.003 | 0.018 | -0.004 | 0.017 |
| rs13080520 | 3 | 81850742 | C | T | 0.011 | 0.002 | -0.002 | 0.013 | -0.014 | 0.017 | 0.013 | 0.016 |
| rs6548834 | 3 | 82704753 | A | G | 0.012 | 0.002 | -0.009 | 0.013 | -0.001 | 0.017 | -0.026 | 0.016 |
| rs12495178 | 3 | 85886077 | T | C | 0.012 | 0.002 | -0.035 | 0.013 | -0.045 | 0.017 | -0.031 | 0.016 |
| rs9942009 | 3 | 89121921 | T | C | 0.013 | 0.002 | -0.002 | 0.013 | -0.017 | 0.016 | -0.003 | 0.016 |
| rs1609906 | 3 | 94033599 | G | A | 0.013 | 0.002 | 0.016 | 0.012 | 0.023 | 0.016 | 0.002 | 0.016 |
| rs793456 | 3 | 99525631 | G | A | 0.011 | 0.002 | 0.026 | 0.013 | 0.020 | 0.016 | 0.036 | 0.016 |
| rs9861425 | 3 | 123072883 | C | A | 0.010 | 0.002 | 0.011 | 0.012 | 0.017 | 0.016 | 0.014 | 0.016 |
| rs111489708 | 3 | 127715518 | C | T | 0.027 | 0.005 | -0.007 | 0.030 | -0.012 | 0.039 | -0.002 | 0.038 |
| rs55683935 | 3 | 128349376 | T | G | 0.038 | 0.005 | 0.007 | 0.034 | -0.032 | 0.044 | 0.035 | 0.044 |
| rs2625963 | 3 | 129292948 | C | T | 0.027 | 0.002 | 0.018 | 0.017 | 0.029 | 0.022 | -0.009 | 0.022 |
| rs13063979 | 3 | 131564741 | G | T | 0.014 | 0.002 | -0.020 | 0.014 | -0.005 | 0.018 | -0.030 | 0.018 |
| rs645040 | 3 | 135926622 | T | G | 0.016 | 0.002 | -0.016 | 0.015 | -0.010 | 0.019 | -0.009 | 0.019 |
| rs13075615 | 3 | 136505832 | C | T | 0.018 | 0.002 | -0.019 | 0.018 | -0.016 | 0.022 | -0.014 | 0.023 |
| rs1672936 | 3 | 138340590 | A | G | 0.012 | 0.002 | 0.001 | 0.013 | 0.008 | 0.016 | 0.000 | 0.016 |
| rs11710396 | 3 | 156278785 | G | A | 0.013 | 0.002 | 0.001 | 0.014 | 0.008 | 0.018 | -0.005 | 0.017 |
| rs10049088 | 3 | 156797648 | C | T | 0.027 | 0.002 | -0.002 | 0.013 | -0.001 | 0.016 | -0.008 | 0.016 |
| rs998749 | 3 | 168972802 | A | G | 0.013 | 0.002 | -0.006 | 0.013 | -0.029 | 0.016 | 0.013 | 0.016 |
| rs4894803 | 3 | 171800256 | A | G | 0.015 | 0.002 | -0.018 | 0.014 | -0.011 | 0.017 | -0.024 | 0.018 |
| rs7647305 | 3 | 185834290 | C | T | 0.014 | 0.002 | -0.022 | 0.015 | -0.022 | 0.020 | -0.035 | 0.019 |
| rs2590440 | 3 | 187674660 | G | A | 0.014 | 0.002 | -0.017 | 0.015 | -0.014 | 0.020 | -0.022 | 0.020 |
| rs11724804 | 4 | 965779 | G | A | 0.017 | 0.002 | -0.010 | 0.013 | -0.024 | 0.017 | -0.006 | 0.016 |
| rs3121419 | 4 | 3232257 | C | T | 0.013 | 0.002 | 0.046 | 0.013 | 0.052 | 0.017 | 0.040 | 0.017 |
| rs10004900 | 4 | 20115547 | G | T | 0.017 | 0.003 | 0.007 | 0.019 | 0.010 | 0.024 | -0.004 | 0.024 |
| rs10019888 | 4 | 26062990 | G | A | 0.021 | 0.002 | 0.066 | 0.017 | 0.106 | 0.021 | 0.037 | 0.021 |
| rs13130484 | 4 | 45175691 | T | C | 0.015 | 0.002 | -0.006 | 0.013 | -0.006 | 0.016 | -0.002 | 0.016 |
| rs12643960 | 4 | 78655671 | G | A | 0.017 | 0.003 | -0.009 | 0.018 | 0.005 | 0.023 | -0.013 | 0.023 |
| rs2167750 | 4 | 89730074 | T | C | 0.020 | 0.002 | 0.001 | 0.012 | 0.000 | 0.016 | -0.004 | 0.016 |
| rs1789882 | 4 | 100235053 | A | G | 0.016 | 0.002 | 0.010 | 0.016 | 0.006 | 0.021 | 0.014 | 0.021 |
| rs3804381 | 4 | 102143616 | A | G | 0.012 | 0.002 | 0.038 | 0.014 | 0.058 | 0.018 | 0.026 | 0.018 |
| rs809955 | 4 | 140874760 | G | A | 0.016 | 0.002 | -0.011 | 0.013 | 0.007 | 0.017 | -0.020 | 0.016 |
| rs789351 | 4 | 145868370 | T | C | 0.012 | 0.002 | 0.023 | 0.013 | 0.030 | 0.016 | 0.027 | 0.016 |
| rs7727202 | 5 | 4007914 | C | A | 0.013 | 0.002 | -0.008 | 0.013 | -0.010 | 0.016 | -0.011 | 0.016 |
| rs2448 | 5 | 53302354 | T | C | 0.016 | 0.002 | -0.005 | 0.014 | 0.004 | 0.018 | -0.018 | 0.018 |
| rs459193 | 5 | 55806751 | A | G | 0.024 | 0.002 | 0.006 | 0.014 | -0.004 | 0.019 | 0.024 | 0.018 |
| rs628763 | 5 | 66312570 | G | T | 0.011 | 0.002 | 0.018 | 0.013 | 0.008 | 0.016 | 0.020 | 0.016 |
| rs4704187 | 5 | 74480288 | T | C | 0.012 | 0.002 | 0.009 | 0.013 | -0.011 | 0.017 | 0.027 | 0.016 |
| rs2112347 | 5 | 75015242 | T | G | 0.015 | 0.002 | 0.008 | 0.013 | -0.013 | 0.017 | 0.029 | 0.016 |
| rs17568628 | 5 | 76046939 | C | T | 0.026 | 0.004 | -0.029 | 0.031 | -0.035 | 0.039 | 0.002 | 0.039 |
| rs13167306 | 5 | 76574841 | C | T | 0.012 | 0.002 | -0.001 | 0.013 | 0.004 | 0.017 | -0.001 | 0.017 |

| | | | | | | | | | | | | |
|---|---|---|---|---|---|---|---|---|---|---|---|---|
| rs6870983 | 5 | 87697533 | C | T | 0.016 | 0.002 | 0.007 | 0.015 | 0.025 | 0.019 | -0.003 | 0.019 |
| rs2161097 | 5 | 103945178 | T | C | 0.013 | 0.002 | 0.014 | 0.012 | 0.002 | 0.016 | 0.018 | 0.016 |
| rs4395620 | 5 | 106328326 | T | C | 0.011 | 0.002 | 0.009 | 0.013 | 0.017 | 0.016 | 0.002 | 0.016 |
| rs11956399 | 5 | 112888676 | T | G | 0.016 | 0.002 | -0.024 | 0.016 | -0.027 | 0.021 | -0.014 | 0.020 |
| rs3813309 | 5 | 118690998 | A | G | 0.014 | 0.002 | -0.016 | 0.014 | -0.021 | 0.018 | -0.014 | 0.017 |
| rs11747001 | 5 | 132412299 | A | G | 0.015 | 0.002 | -0.031 | 0.014 | -0.044 | 0.019 | -0.022 | 0.018 |
| rs11167753 | 5 | 140989410 | C | T | 0.011 | 0.002 | 0.038 | 0.014 | 0.071 | 0.018 | 0.012 | 0.018 |
| rs10477191 | 5 | 142077715 | G | A | 0.028 | 0.004 | -0.023 | 0.032 | -0.023 | 0.041 | -0.005 | 0.040 |
| rs2964006 | 5 | 153213880 | T | G | 0.013 | 0.002 | -0.009 | 0.012 | 0.011 | 0.016 | -0.021 | 0.016 |
| rs4454042 | 5 | 155824774 | T | C | 0.013 | 0.002 | 0.000 | 0.014 | 0.001 | 0.018 | -0.003 | 0.018 |
| rs1122080 | 5 | 158015903 | G | A | 0.013 | 0.002 | 0.013 | 0.016 | 0.032 | 0.021 | 0.002 | 0.020 |
| rs72846967 | 5 | 171849098 | G | A | 0.012 | 0.002 | 0.000 | 0.014 | -0.007 | 0.018 | -0.001 | 0.017 |
| rs17738166 | 5 | 172997978 | A | G | 0.011 | 0.002 | 0.003 | 0.013 | -0.001 | 0.016 | 0.013 | 0.016 |
| rs6861681 | 5 | 173362458 | A | G | 0.018 | 0.002 | -0.040 | 0.014 | -0.062 | 0.018 | -0.022 | 0.017 |
| rs6556301 | 5 | 176527577 | T | G | 0.013 | 0.002 | 0.001 | 0.013 | -0.001 | 0.017 | 0.006 | 0.017 |
| rs1294410 | 6 | 6738752 | C | T | 0.025 | 0.002 | 0.012 | 0.013 | -0.002 | 0.017 | 0.019 | 0.016 |
| rs1334576 | 6 | 7211818 | G | A | 0.016 | 0.002 | 0.010 | 0.013 | 0.016 | 0.016 | 0.004 | 0.016 |
| rs9296938 | 6 | 14573063 | G | A | 0.015 | 0.002 | -0.030 | 0.014 | -0.018 | 0.018 | -0.037 | 0.018 |
| rs112266013 | 6 | 15230743 | G | A | 0.017 | 0.003 | -0.042 | 0.018 | -0.030 | 0.024 | -0.052 | 0.023 |
| rs7744833 | 6 | 20581828 | A | G | 0.013 | 0.002 | -0.044 | 0.013 | -0.060 | 0.017 | -0.027 | 0.017 |
| rs3094621 | 6 | 30328753 | T | C | 0.019 | 0.003 | 0.070 | 0.021 | 0.162 | 0.028 | 0.000 | 0.027 |
| rs1793892 | 6 | 31221351 | C | T | 0.018 | 0.003 | 0.044 | 0.020 | 0.107 | 0.025 | 0.000 | 0.026 |
| rs454748 | 6 | 32213210 | A | G | 0.015 | 0.002 | 0.027 | 0.014 | 0.053 | 0.018 | 0.004 | 0.018 |
| rs3117324 | 6 | 33324527 | T | G | 0.012 | 0.002 | 0.010 | 0.013 | 0.006 | 0.017 | 0.016 | 0.016 |
| rs114760566 | 6 | 34192036 | A | C | 0.067 | 0.005 | -0.022 | 0.032 | -0.014 | 0.041 | -0.027 | 0.040 |
| rs147499178 | 6 | 34817079 | T | C | 0.048 | 0.006 | -0.057 | 0.047 | -0.039 | 0.059 | -0.074 | 0.059 |
| rs146874451 | 6 | 35383027 | G | A | 0.040 | 0.006 | -0.072 | 0.042 | -0.095 | 0.053 | -0.074 | 0.053 |
| rs4487571 | 6 | 41702619 | A | C | 0.021 | 0.004 | 0.036 | 0.026 | 0.042 | 0.034 | 0.025 | 0.032 |
| rs3828755 | 6 | 43115220 | G | A | 0.013 | 0.002 | 0.013 | 0.013 | 0.010 | 0.016 | 0.023 | 0.016 |
| rs998584 | 6 | 43757896 | A | C | 0.035 | 0.002 | -0.006 | 0.013 | -0.005 | 0.017 | 0.005 | 0.016 |
| rs17665162 | 6 | 50275258 | C | T | 0.023 | 0.004 | 0.026 | 0.027 | 0.027 | 0.036 | 0.020 | 0.035 |
| rs987237 | 6 | 50803050 | G | A | 0.020 | 0.002 | 0.003 | 0.016 | 0.010 | 0.021 | 0.001 | 0.020 |
| rs9370243 | 6 | 53789830 | T | G | 0.020 | 0.003 | -0.014 | 0.022 | 0.002 | 0.029 | -0.034 | 0.029 |
| rs2503749 | 6 | 81349756 | T | G | 0.014 | 0.002 | -0.019 | 0.012 | -0.024 | 0.016 | -0.016 | 0.016 |
| rs72895340 | 6 | 82297307 | A | G | 0.028 | 0.005 | -0.003 | 0.029 | -0.015 | 0.038 | 0.014 | 0.037 |
| rs9294260 | 6 | 83433228 | A | G | 0.010 | 0.002 | -0.024 | 0.013 | -0.028 | 0.016 | -0.035 | 0.016 |
| rs9341990 | 6 | 85391568 | A | G | 0.012 | 0.002 | 0.016 | 0.013 | 0.012 | 0.016 | 0.020 | 0.016 |
| rs10499013 | 6 | 97946396 | G | A | 0.013 | 0.002 | -0.007 | 0.014 | -0.033 | 0.018 | 0.010 | 0.018 |
| rs901630 | 6 | 98539519 | C | T | 0.012 | 0.002 | 0.005 | 0.013 | -0.004 | 0.016 | 0.002 | 0.016 |
| rs2503099 | 6 | 100610101 | G | A | 0.021 | 0.002 | -0.019 | 0.017 | -0.017 | 0.021 | -0.015 | 0.021 |
| rs9400239 | 6 | 108977663 | C | T | 0.013 | 0.002 | 0.001 | 0.014 | -0.007 | 0.018 | 0.012 | 0.017 |
| rs2357760 | 6 | 120213880 | A | G | 0.011 | 0.002 | -0.003 | 0.013 | 0.004 | 0.017 | -0.014 | 0.017 |
| rs9375417 | 6 | 126248374 | C | T | 0.012 | 0.002 | -0.013 | 0.012 | 0.004 | 0.016 | -0.027 | 0.016 |
| rs143474978 | 6 | 126814933 | C | T | 0.074 | 0.006 | -0.017 | 0.036 | 0.011 | 0.047 | -0.061 | 0.047 |
| rs72959041 | 6 | 127454893 | A | G | 0.126 | 0.004 | -0.026 | 0.031 | 0.018 | 0.039 | -0.090 | 0.039 |
| rs605066 | 6 | 139829666 | C | T | 0.019 | 0.002 | -0.027 | 0.013 | -0.027 | 0.016 | -0.022 | 0.016 |
| rs672341 | 6 | 153455994 | G | A | 0.012 | 0.002 | 0.010 | 0.013 | 0.011 | 0.016 | 0.007 | 0.016 |
| rs668871 | 6 | 160769811 | C | T | 0.013 | 0.002 | -0.011 | 0.012 | -0.002 | 0.016 | -0.018 | 0.016 |
| rs6955431 | 7 | 20444060 | T | C | 0.011 | 0.002 | -0.031 | 0.013 | -0.035 | 0.017 | -0.029 | 0.017 |
| rs2391168 | 7 | 25862790 | A | C | 0.026 | 0.002 | -0.026 | 0.016 | -0.015 | 0.020 | -0.034 | 0.020 |
| rs1534696 | 7 | 26397239 | C | A | 0.023 | 0.002 | -0.004 | 0.013 | 0.000 | 0.017 | -0.003 | 0.016 |

| | | | | | | | | | | | | |
|---|---|---|---|---|---|---|---|---|---|---|---|---|
| rs7801581 | 7 | 27223771 | T | C | 0.017 | 0.002 | -0.043 | 0.015 | -0.017 | 0.019 | -0.067 | 0.019 |
| rs1708302 | 7 | 28198677 | C | T | 0.011 | 0.002 | 0.036 | 0.012 | 0.068 | 0.016 | 0.002 | 0.016 |
| rs2715135 | 7 | 50750128 | T | G | 0.011 | 0.002 | 0.024 | 0.013 | 0.028 | 0.017 | 0.018 | 0.016 |
| rs1718618 | 7 | 68632251 | G | A | 0.024 | 0.004 | 0.018 | 0.028 | 0.002 | 0.036 | 0.045 | 0.036 |
| rs55747707 | 7 | 73037366 | G | A | 0.015 | 0.002 | 0.039 | 0.016 | 0.056 | 0.021 | 0.025 | 0.020 |
| rs12669521 | 7 | 77047102 | A | G | 0.013 | 0.002 | 0.005 | 0.013 | -0.018 | 0.017 | 0.031 | 0.017 |
| rs11764879 | 7 | 77333267 | G | A | 0.012 | 0.002 | -0.017 | 0.014 | -0.021 | 0.018 | -0.011 | 0.018 |
| rs1011024 | 7 | 99197401 | A | G | 0.015 | 0.002 | -0.016 | 0.017 | -0.005 | 0.022 | -0.026 | 0.022 |
| rs1142 | 7 | 104756326 | T | C | 0.015 | 0.002 | 0.008 | 0.013 | 0.018 | 0.017 | 0.012 | 0.017 |
| rs4727695 | 7 | 107614003 | A | G | 0.022 | 0.003 | 0.001 | 0.021 | -0.016 | 0.027 | 0.022 | 0.027 |
| rs4476935 | 7 | 112987650 | C | T | 0.011 | 0.002 | -0.010 | 0.013 | -0.043 | 0.016 | 0.021 | 0.016 |
| rs39312 | 7 | 116954785 | C | A | 0.015 | 0.002 | 0.013 | 0.013 | 0.034 | 0.017 | -0.008 | 0.017 |
| rs6466774 | 7 | 120886915 | T | C | 0.013 | 0.002 | 0.005 | 0.013 | -0.001 | 0.016 | 0.018 | 0.016 |
| rs13229637 | 7 | 136717447 | T | C | 0.016 | 0.003 | 0.011 | 0.017 | 0.033 | 0.022 | -0.007 | 0.022 |
| rs9969455 | 8 | 12617155 | A | G | 0.011 | 0.002 | -0.013 | 0.013 | 0.006 | 0.017 | -0.028 | 0.017 |
| rs15285 | 8 | 19824667 | C | T | 0.012 | 0.002 | 0.022 | 0.014 | 0.028 | 0.018 | 0.012 | 0.018 |
| rs6983481 | 8 | 23609009 | G | T | 0.019 | 0.002 | 0.012 | 0.015 | 0.009 | 0.019 | 0.006 | 0.018 |
| rs11992444 | 8 | 25464690 | T | G | 0.019 | 0.002 | 0.010 | 0.015 | -0.003 | 0.020 | 0.004 | 0.020 |
| rs2725371 | 8 | 30854033 | A | G | 0.017 | 0.002 | 0.026 | 0.013 | 0.027 | 0.017 | 0.019 | 0.017 |
| rs881301 | 8 | 38332318 | C | T | 0.013 | 0.002 | 0.001 | 0.013 | -0.009 | 0.016 | 0.006 | 0.016 |
| rs62506196 | 8 | 60264465 | C | A | 0.016 | 0.003 | 0.038 | 0.017 | 0.052 | 0.022 | 0.027 | 0.022 |
| rs13255070 | 8 | 68203608 | G | A | 0.014 | 0.002 | -0.015 | 0.014 | 0.002 | 0.018 | -0.028 | 0.018 |
| rs4738141 | 8 | 72469742 | G | A | 0.020 | 0.002 | 0.010 | 0.014 | 0.019 | 0.018 | 0.002 | 0.018 |
| rs1431659 | 8 | 73439070 | A | G | 0.014 | 0.002 | -0.013 | 0.014 | -0.001 | 0.018 | -0.020 | 0.018 |
| rs13256367 | 8 | 128334900 | A | C | 0.014 | 0.002 | -0.008 | 0.013 | 0.013 | 0.017 | -0.019 | 0.017 |
| rs10505628 | 8 | 135708575 | C | T | 0.010 | 0.002 | 0.015 | 0.013 | 0.022 | 0.016 | 0.009 | 0.016 |
| rs11782074 | 8 | 142617096 | T | G | 0.011 | 0.002 | -0.022 | 0.013 | -0.003 | 0.017 | -0.040 | 0.016 |
| rs6474945 | 9 | 15670492 | G | T | 0.010 | 0.002 | -0.001 | 0.012 | 0.000 | 0.016 | 0.002 | 0.016 |
| rs10968576 | 9 | 28414339 | G | A | 0.014 | 0.002 | 0.018 | 0.013 | 0.025 | 0.017 | 0.015 | 0.017 |
| rs1680490 | 9 | 29642688 | G | A | 0.012 | 0.002 | 0.001 | 0.013 | -0.009 | 0.017 | 0.009 | 0.017 |
| rs10991926 | 9 | 94180339 | T | C | 0.012 | 0.002 | 0.021 | 0.013 | 0.026 | 0.017 | 0.011 | 0.017 |
| rs10821003 | 9 | 95452843 | A | C | 0.017 | 0.002 | 0.064 | 0.016 | 0.078 | 0.021 | 0.064 | 0.021 |
| rs7848336 | 9 | 96454513 | C | T | 0.013 | 0.002 | -0.006 | 0.014 | -0.011 | 0.018 | 0.004 | 0.018 |
| rs2398893 | 9 | 96758342 | A | G | 0.016 | 0.002 | -0.034 | 0.014 | -0.029 | 0.018 | -0.049 | 0.018 |
| rs62565259 | 9 | 102162570 | C | T | 0.016 | 0.003 | -0.032 | 0.017 | -0.028 | 0.022 | -0.043 | 0.022 |
| rs10991433 | 9 | 107726918 | C | T | 0.025 | 0.003 | 0.021 | 0.021 | -0.010 | 0.027 | 0.051 | 0.026 |
| rs10978310 | 9 | 108622461 | A | G | 0.023 | 0.004 | -0.015 | 0.028 | 0.003 | 0.036 | -0.025 | 0.036 |
| rs10116353 | 9 | 111950088 | G | T | 0.014 | 0.002 | 0.020 | 0.014 | 0.003 | 0.017 | 0.038 | 0.017 |
| rs10980797 | 9 | 113912553 | G | A | 0.015 | 0.002 | 0.011 | 0.012 | 0.025 | 0.016 | 0.002 | 0.016 |
| rs1752169 | 9 | 126586563 | A | C | 0.012 | 0.002 | -0.006 | 0.014 | 0.001 | 0.018 | -0.012 | 0.018 |
| rs4382592 | 9 | 134870755 | T | G | 0.012 | 0.002 | 0.019 | 0.014 | 0.025 | 0.017 | 0.019 | 0.017 |
| rs28647893 | 9 | 136994893 | C | T | 0.012 | 0.002 | 0.001 | 0.014 | 0.030 | 0.018 | -0.023 | 0.017 |
| rs10795055 | 10 | 3581221 | A | G | 0.011 | 0.002 | 0.019 | 0.013 | 0.030 | 0.016 | 0.018 | 0.016 |
| rs12774134 | 10 | 4963327 | C | T | 0.016 | 0.003 | -0.012 | 0.019 | -0.029 | 0.025 | 0.001 | 0.025 |
| rs7907173 | 10 | 5648787 | G | A | 0.011 | 0.002 | 0.004 | 0.012 | -0.011 | 0.016 | 0.016 | 0.016 |
| rs1243188 | 10 | 21908803 | C | T | 0.015 | 0.002 | 0.029 | 0.013 | 0.019 | 0.017 | 0.031 | 0.017 |
| rs1494204 | 10 | 27904321 | C | T | 0.011 | 0.002 | -0.007 | 0.013 | 0.012 | 0.016 | -0.021 | 0.016 |
| rs2907794 | 10 | 32391109 | G | A | 0.012 | 0.002 | 0.057 | 0.015 | 0.054 | 0.019 | 0.054 | 0.019 |
| rs10827252 | 10 | 33672884 | G | A | 0.011 | 0.002 | -0.017 | 0.013 | -0.007 | 0.016 | -0.021 | 0.016 |
| rs1757471 | 10 | 34168090 | T | C | 0.013 | 0.002 | -0.005 | 0.012 | -0.002 | 0.016 | -0.005 | 0.016 |
| rs708437 | 10 | 36227656 | A | G | 0.014 | 0.002 | 0.011 | 0.016 | -0.022 | 0.021 | 0.042 | 0.021 |

| | | | | | | | | | | | | |
|---|---|---|---|---|---|---|---|---|---|---|---|---|
| rs7070670 | 10 | 61842645 | C | T | 0.013 | 0.002 | -0.023 | 0.014 | -0.046 | 0.017 | -0.019 | 0.017 |
| rs7070749 | 10 | 63882682 | A | G | 0.013 | 0.002 | 0.008 | 0.013 | 0.005 | 0.016 | 0.005 | 0.016 |
| rs780159 | 10 | 80907147 | G | A | 0.013 | 0.002 | -0.016 | 0.013 | -0.013 | 0.016 | -0.025 | 0.016 |
| rs3814614 | 10 | 88128281 | A | G | 0.011 | 0.002 | -0.008 | 0.013 | -0.022 | 0.016 | -0.002 | 0.016 |
| rs10788569 | 10 | 89604732 | C | T | 0.013 | 0.002 | 0.013 | 0.014 | 0.021 | 0.018 | 0.009 | 0.018 |
| rs117471638 | 10 | 93158084 | A | G | 0.042 | 0.007 | -0.156 | 0.049 | -0.142 | 0.064 | -0.108 | 0.062 |
| rs1437 | 10 | 93790523 | A | G | 0.013 | 0.002 | 0.034 | 0.013 | 0.020 | 0.017 | 0.041 | 0.016 |
| rs10882271 | 10 | 95346067 | A | G | 0.014 | 0.002 | 0.021 | 0.014 | 0.021 | 0.018 | 0.020 | 0.018 |
| rs10883518 | 10 | 102416555 | C | A | 0.011 | 0.002 | -0.008 | 0.013 | 0.006 | 0.017 | -0.011 | 0.017 |
| rs12777288 | 10 | 115860058 | C | T | 0.014 | 0.002 | 0.017 | 0.015 | -0.004 | 0.019 | 0.034 | 0.019 |
| rs2254069 | 10 | 122875589 | A | G | 0.021 | 0.003 | -0.023 | 0.020 | -0.014 | 0.025 | -0.036 | 0.025 |
| rs61876729 | 11 | 778857 | A | G | 0.021 | 0.003 | -0.017 | 0.023 | -0.021 | 0.030 | -0.035 | 0.030 |
| rs4929927 | 11 | 8658485 | G | A | 0.015 | 0.002 | 0.001 | 0.013 | 0.014 | 0.017 | -0.013 | 0.016 |
| rs2957658 | 11 | 10393468 | G | A | 0.013 | 0.002 | -0.010 | 0.013 | -0.031 | 0.016 | 0.006 | 0.016 |
| rs7932891 | 11 | 10921512 | A | G | 0.011 | 0.002 | -0.009 | 0.014 | 0.011 | 0.018 | -0.022 | 0.018 |
| rs1481880 | 11 | 13269548 | A | G | 0.013 | 0.002 | 0.008 | 0.014 | -0.002 | 0.018 | 0.007 | 0.018 |
| rs4141261 | 11 | 17708379 | G | T | 0.011 | 0.002 | -0.008 | 0.013 | -0.021 | 0.017 | -0.006 | 0.017 |
| rs11030107 | 11 | 27694835 | G | A | 0.018 | 0.002 | -0.002 | 0.014 | -0.004 | 0.019 | -0.010 | 0.018 |
| rs605765 | 11 | 30217503 | C | T | 0.011 | 0.002 | -0.015 | 0.013 | -0.011 | 0.016 | -0.024 | 0.016 |
| rs4755720 | 11 | 43628749 | C | T | 0.013 | 0.002 | 0.006 | 0.013 | 0.014 | 0.016 | -0.007 | 0.016 |
| rs11039290 | 11 | 47572279 | G | A | 0.017 | 0.002 | -0.005 | 0.013 | 0.004 | 0.017 | -0.007 | 0.017 |
| rs2428548 | 11 | 62311462 | G | A | 0.015 | 0.002 | -0.008 | 0.013 | -0.023 | 0.017 | -0.013 | 0.016 |
| rs35169799 | 11 | 64031241 | T | C | 0.037 | 0.004 | 0.070 | 0.027 | 0.092 | 0.034 | 0.053 | 0.034 |
| rs10896012 | 11 | 65278461 | C | T | 0.018 | 0.002 | -0.022 | 0.016 | -0.032 | 0.020 | -0.010 | 0.020 |
| rs2276106 | 11 | 66105817 | T | C | 0.015 | 0.002 | -0.007 | 0.014 | -0.013 | 0.018 | -0.006 | 0.018 |
| rs7395513 | 11 | 69262756 | G | A | 0.017 | 0.002 | -0.020 | 0.013 | -0.021 | 0.016 | -0.015 | 0.016 |
| rs536665 | 11 | 85322400 | G | A | 0.017 | 0.002 | 0.010 | 0.016 | 0.005 | 0.020 | 0.023 | 0.020 |
| rs647248 | 11 | 89917699 | A | G | 0.011 | 0.002 | -0.009 | 0.013 | 0.000 | 0.016 | -0.024 | 0.016 |
| rs2513987 | 11 | 102977271 | G | A | 0.014 | 0.002 | 0.013 | 0.014 | 0.000 | 0.018 | 0.018 | 0.018 |
| rs2276390 | 11 | 111895254 | G | T | 0.021 | 0.002 | -0.007 | 0.013 | -0.012 | 0.017 | -0.001 | 0.017 |
| rs11214589 | 11 | 113245048 | G | A | 0.011 | 0.002 | 0.004 | 0.013 | -0.002 | 0.016 | 0.000 | 0.016 |
| rs11216183 | 11 | 116781545 | A | C | 0.023 | 0.003 | 0.025 | 0.021 | -0.002 | 0.027 | 0.033 | 0.027 |
| rs3825061 | 11 | 118944675 | T | C | 0.014 | 0.002 | 0.001 | 0.013 | 0.006 | 0.016 | 0.001 | 0.016 |
| rs579682 | 11 | 122014110 | C | T | 0.013 | 0.002 | 0.018 | 0.014 | -0.011 | 0.018 | 0.044 | 0.017 |
| rs2512885 | 11 | 131467794 | C | T | 0.010 | 0.002 | 0.022 | 0.012 | 0.020 | 0.016 | 0.020 | 0.016 |
| rs4936175 | 11 | 132641959 | C | T | 0.010 | 0.002 | -0.020 | 0.013 | -0.017 | 0.016 | -0.019 | 0.016 |
| rs12828016 | 12 | 998365 | G | T | 0.011 | 0.002 | 0.028 | 0.013 | -0.005 | 0.016 | 0.052 | 0.016 |
| rs7222 | 12 | 2055266 | T | C | 0.011 | 0.002 | -0.020 | 0.013 | -0.035 | 0.016 | -0.004 | 0.016 |
| rs544668 | 12 | 3388004 | T | C | 0.011 | 0.002 | 0.014 | 0.013 | 0.007 | 0.016 | 0.012 | 0.016 |
| rs34322 | 12 | 12879570 | T | C | 0.010 | 0.002 | 0.017 | 0.013 | 0.024 | 0.016 | 0.007 | 0.016 |
| rs11055887 | 12 | 14417179 | G | A | 0.013 | 0.002 | 0.041 | 0.016 | 0.036 | 0.021 | 0.041 | 0.020 |
| rs11048456 | 12 | 26463082 | C | T | 0.027 | 0.002 | 0.026 | 0.014 | 0.004 | 0.018 | 0.036 | 0.018 |
| rs11051005 | 12 | 30783475 | A | G | 0.013 | 0.002 | -0.051 | 0.014 | -0.022 | 0.018 | -0.070 | 0.018 |
| rs10506110 | 12 | 33708552 | A | C | 0.014 | 0.002 | -0.012 | 0.013 | -0.007 | 0.017 | -0.018 | 0.017 |
| rs12372466 | 12 | 34636450 | A | G | 0.011 | 0.002 | -0.005 | 0.013 | -0.008 | 0.017 | -0.005 | 0.016 |
| rs11183232 | 12 | 37941053 | A | G | 0.011 | 0.002 | -0.001 | 0.013 | -0.004 | 0.017 | -0.002 | 0.016 |
| rs1026462 | 12 | 41821630 | G | A | 0.013 | 0.002 | 0.004 | 0.012 | 0.028 | 0.016 | -0.030 | 0.016 |
| rs7138803 | 12 | 50247468 | A | G | 0.013 | 0.002 | 0.017 | 0.013 | 0.015 | 0.017 | 0.019 | 0.016 |
| rs1443512 | 12 | 54342684 | A | C | 0.028 | 0.002 | -0.005 | 0.015 | -0.018 | 0.019 | 0.011 | 0.019 |
| rs2277339 | 12 | 57146069 | G | T | 0.017 | 0.003 | 0.035 | 0.021 | 0.035 | 0.026 | 0.026 | 0.026 |
| rs11176015 | 12 | 66441684 | T | C | 0.015 | 0.002 | -0.003 | 0.014 | 0.010 | 0.018 | -0.004 | 0.018 |

| | | | | | | | | | | | | |
|---|---|---|---|---|---|---|---|---|---|---|---|---|
| rs704061 | 12 | 89771903 | C | T | 0.012 | 0.002 | -0.008 | 0.012 | 0.021 | 0.016 | -0.035 | 0.016 |
| rs11107169 | 12 | 94126855 | A | G | 0.012 | 0.002 | 0.000 | 0.013 | -0.028 | 0.016 | 0.025 | 0.016 |
| rs7311622 | 12 | 98772975 | C | T | 0.012 | 0.002 | -0.019 | 0.013 | -0.024 | 0.016 | -0.014 | 0.016 |
| rs3764002 | 12 | 108618630 | C | T | 0.021 | 0.002 | 0.021 | 0.014 | 0.041 | 0.018 | 0.003 | 0.018 |
| rs74628422 | 12 | 116034706 | A | G | 0.014 | 0.002 | 0.004 | 0.015 | -0.029 | 0.020 | 0.024 | 0.019 |
| rs7961979 | 12 | 121671261 | A | C | 0.016 | 0.003 | -0.012 | 0.019 | 0.015 | 0.025 | -0.030 | 0.024 |
| rs11608693 | 12 | 122208968 | G | A | 0.017 | 0.003 | 0.019 | 0.018 | 0.016 | 0.023 | 0.018 | 0.024 |
| rs2454702 | 12 | 123209159 | C | T | 0.020 | 0.002 | 0.015 | 0.015 | 0.009 | 0.020 | 0.015 | 0.020 |
| rs1568427 | 12 | 123738678 | G | A | 0.019 | 0.002 | -0.014 | 0.014 | -0.028 | 0.018 | -0.004 | 0.018 |
| rs863750 | 12 | 124505444 | T | C | 0.026 | 0.002 | -0.011 | 0.013 | -0.006 | 0.016 | -0.022 | 0.016 |
| rs12828318 | 12 | 133766122 | A | G | 0.013 | 0.002 | 0.028 | 0.018 | 0.032 | 0.023 | 0.021 | 0.023 |
| rs1360485 | 13 | 31031884 | T | C | 0.015 | 0.002 | 0.010 | 0.014 | 0.010 | 0.018 | 0.010 | 0.017 |
| rs2475837 | 13 | 50535863 | T | C | 0.015 | 0.003 | 0.006 | 0.020 | 0.027 | 0.025 | -0.014 | 0.025 |
| rs797486 | 13 | 51221618 | A | C | 0.032 | 0.003 | -0.001 | 0.019 | 0.006 | 0.024 | -0.004 | 0.024 |
| rs1379828 | 13 | 54396602 | C | T | 0.013 | 0.002 | -0.026 | 0.015 | -0.040 | 0.020 | -0.015 | 0.020 |
| rs564930 | 13 | 54756074 | T | C | 0.020 | 0.003 | 0.010 | 0.019 | 0.027 | 0.024 | 0.002 | 0.024 |
| rs9569777 | 13 | 58484786 | G | T | 0.013 | 0.002 | -0.002 | 0.017 | 0.002 | 0.022 | -0.017 | 0.021 |
| rs4055791 | 13 | 59266053 | C | T | 0.012 | 0.002 | -0.011 | 0.013 | -0.028 | 0.016 | -0.001 | 0.016 |
| rs1441264 | 13 | 79580919 | A | G | 0.011 | 0.002 | -0.024 | 0.013 | -0.032 | 0.017 | -0.017 | 0.016 |
| rs12430764 | 13 | 93896935 | A | G | 0.011 | 0.002 | 0.018 | 0.012 | 0.032 | 0.016 | 0.012 | 0.016 |
| rs9556979 | 13 | 99241507 | G | T | 0.011 | 0.002 | 0.036 | 0.014 | 0.037 | 0.018 | 0.036 | 0.017 |
| rs9515201 | 13 | 111040798 | C | A | 0.012 | 0.002 | 0.025 | 0.014 | 0.029 | 0.018 | 0.022 | 0.017 |
| rs1163627 | 13 | 112225701 | A | C | 0.010 | 0.002 | 0.003 | 0.013 | 0.001 | 0.016 | -0.002 | 0.016 |
| rs10132280 | 14 | 25928179 | C | A | 0.012 | 0.002 | -0.021 | 0.014 | -0.020 | 0.018 | -0.027 | 0.017 |
| rs61986159 | 14 | 53956168 | T | C | 0.014 | 0.002 | 0.004 | 0.015 | -0.018 | 0.019 | 0.017 | 0.019 |
| rs1190982 | 14 | 58815839 | T | C | 0.016 | 0.002 | 0.000 | 0.014 | -0.011 | 0.018 | 0.008 | 0.017 |
| rs2412107 | 14 | 65426216 | T | G | 0.013 | 0.002 | -0.002 | 0.015 | 0.009 | 0.019 | -0.005 | 0.019 |
| rs2526886 | 14 | 71359064 | T | G | 0.013 | 0.002 | -0.013 | 0.013 | -0.017 | 0.017 | -0.011 | 0.017 |
| rs17109256 | 14 | 79939993 | A | G | 0.017 | 0.002 | 0.036 | 0.015 | 0.015 | 0.019 | 0.047 | 0.019 |
| rs11620623 | 14 | 91555547 | C | T | 0.011 | 0.002 | -0.010 | 0.014 | -0.001 | 0.018 | -0.015 | 0.018 |
| rs7143963 | 14 | 103304425 | T | C | 0.014 | 0.002 | 0.013 | 0.016 | 0.010 | 0.021 | 0.030 | 0.021 |
| rs12441543 | 15 | 31689543 | G | A | 0.012 | 0.002 | 0.009 | 0.014 | -0.002 | 0.018 | 0.023 | 0.018 |
| rs8036817 | 15 | 40970724 | A | C | 0.012 | 0.002 | 0.000 | 0.013 | 0.004 | 0.016 | 0.002 | 0.016 |
| rs12440605 | 15 | 42102285 | A | G | 0.012 | 0.002 | -0.023 | 0.012 | -0.016 | 0.016 | -0.033 | 0.016 |
| rs3736485 | 15 | 51748610 | A | G | 0.012 | 0.002 | 0.011 | 0.013 | 0.014 | 0.016 | 0.009 | 0.016 |
| rs2456530 | 15 | 53091553 | T | C | 0.016 | 0.003 | 0.012 | 0.019 | 0.010 | 0.024 | 0.016 | 0.024 |
| rs12595496 | 15 | 56528806 | G | A | 0.018 | 0.003 | -0.045 | 0.019 | -0.058 | 0.024 | -0.026 | 0.024 |
| rs1657930 | 15 | 57120989 | G | A | 0.013 | 0.002 | -0.007 | 0.015 | 0.005 | 0.020 | -0.012 | 0.019 |
| rs12440695 | 15 | 62435156 | C | T | 0.010 | 0.002 | -0.002 | 0.013 | -0.006 | 0.017 | 0.008 | 0.016 |
| rs11071759 | 15 | 63922474 | T | C | 0.010 | 0.002 | -0.034 | 0.013 | -0.048 | 0.016 | -0.028 | 0.016 |
| rs1440372 | 15 | 67033151 | C | T | 0.014 | 0.002 | -0.015 | 0.014 | -0.013 | 0.018 | -0.017 | 0.018 |
| rs8043060 | 15 | 67661784 | G | A | 0.017 | 0.002 | 0.046 | 0.015 | 0.053 | 0.019 | 0.044 | 0.019 |
| rs8039418 | 15 | 73441432 | C | T | 0.011 | 0.002 | -0.011 | 0.013 | -0.022 | 0.016 | -0.008 | 0.016 |
| rs7183908 | 15 | 74329193 | C | T | 0.016 | 0.002 | 0.011 | 0.012 | 0.000 | 0.016 | 0.012 | 0.016 |
| rs936226 | 15 | 75069282 | T | C | 0.011 | 0.002 | 0.013 | 0.014 | 0.010 | 0.018 | 0.018 | 0.018 |
| rs12593088 | 15 | 81058640 | G | A | 0.013 | 0.002 | 0.012 | 0.014 | 0.022 | 0.018 | 0.006 | 0.017 |
| rs12101386 | 15 | 92571283 | G | T | 0.014 | 0.002 | 0.013 | 0.015 | 0.046 | 0.020 | -0.013 | 0.019 |
| rs8024294 | 15 | 94023132 | A | G | 0.018 | 0.003 | -0.007 | 0.021 | -0.013 | 0.027 | -0.009 | 0.026 |
| rs2862867 | 15 | 100268584 | T | C | 0.011 | 0.002 | 0.019 | 0.013 | 0.011 | 0.017 | 0.028 | 0.016 |
| rs3747579 | 16 | 4445327 | C | T | 0.015 | 0.002 | 0.010 | 0.014 | 0.006 | 0.018 | 0.015 | 0.018 |
| rs7200819 | 16 | 4945985 | G | A | 0.015 | 0.003 | 0.025 | 0.018 | 0.020 | 0.024 | 0.022 | 0.023 |

| | | | | | | | | | | | | |
|---|---|---|---|---|---|---|---|---|---|---|---|---|
| rs4782289 | 16 | 19859332 | G | A | 0.015 | 0.003 | 0.029 | 0.018 | 0.049 | 0.024 | 0.026 | 0.023 |
| rs7186893 | 16 | 24806420 | G | T | 0.014 | 0.002 | 0.038 | 0.014 | 0.046 | 0.018 | 0.034 | 0.018 |
| rs2650494 | 16 | 28318440 | A | G | 0.011 | 0.002 | 0.052 | 0.013 | 0.052 | 0.017 | 0.043 | 0.017 |
| rs2008514 | 16 | 28825605 | A | G | 0.017 | 0.002 | 0.094 | 0.013 | 0.113 | 0.016 | 0.073 | 0.016 |
| rs4788204 | 16 | 29995218 | A | G | 0.017 | 0.002 | -0.014 | 0.012 | -0.022 | 0.016 | -0.006 | 0.016 |
| rs2047937 | 16 | 49864791 | C | T | 0.011 | 0.002 | 0.000 | 0.013 | -0.016 | 0.016 | 0.007 | 0.016 |
| rs1421085 | 16 | 53800954 | C | T | 0.040 | 0.002 | 0.026 | 0.013 | 0.039 | 0.016 | 0.014 | 0.016 |
| rs889398 | 16 | 69556715 | C | T | 0.017 | 0.002 | 0.024 | 0.013 | 0.025 | 0.016 | 0.026 | 0.016 |
| rs2925979 | 16 | 81534790 | T | C | 0.022 | 0.002 | -0.036 | 0.014 | -0.013 | 0.018 | -0.049 | 0.017 |
| rs7196397 | 16 | 82874522 | T | C | 0.013 | 0.002 | 0.046 | 0.013 | 0.092 | 0.017 | -0.001 | 0.017 |
| rs7198287 | 16 | 85258191 | C | T | 0.014 | 0.002 | 0.021 | 0.015 | 0.001 | 0.020 | 0.042 | 0.020 |
| rs8070737 | 17 | 3981066 | T | G | 0.016 | 0.002 | 0.002 | 0.016 | 0.023 | 0.021 | -0.014 | 0.021 |
| rs858519 | 17 | 7531965 | T | C | 0.012 | 0.002 | 0.011 | 0.013 | 0.035 | 0.017 | -0.008 | 0.016 |
| rs12947517 | 17 | 17539278 | C | T | 0.014 | 0.002 | 0.029 | 0.013 | 0.013 | 0.017 | 0.046 | 0.016 |
| rs672356 | 17 | 18266567 | G | A | 0.013 | 0.002 | 0.006 | 0.014 | 0.002 | 0.018 | 0.016 | 0.018 |
| rs7213608 | 17 | 21279289 | C | T | 0.017 | 0.002 | -0.017 | 0.014 | -0.015 | 0.018 | -0.019 | 0.017 |
| rs2306589 | 17 | 34848874 | T | C | 0.016 | 0.002 | 0.023 | 0.012 | 0.010 | 0.016 | 0.038 | 0.016 |
| rs72820838 | 17 | 40113631 | T | C | 0.025 | 0.004 | -0.053 | 0.031 | -0.027 | 0.041 | -0.062 | 0.039 |
| rs591939 | 17 | 40698075 | G | A | 0.018 | 0.002 | -0.068 | 0.015 | -0.076 | 0.019 | -0.064 | 0.019 |
| rs62064595 | 17 | 43460374 | G | A | 0.021 | 0.003 | -0.038 | 0.019 | -0.038 | 0.024 | -0.041 | 0.024 |
| rs62063286 | 17 | 44041987 | T | C | 0.024 | 0.002 | 0.011 | 0.018 | 0.016 | 0.023 | 0.002 | 0.024 |
| rs62074125 | 17 | 44852612 | A | C | 0.020 | 0.002 | 0.011 | 0.016 | 0.029 | 0.021 | -0.008 | 0.021 |
| rs11079810 | 17 | 46227846 | C | T | 0.017 | 0.003 | -0.064 | 0.020 | -0.047 | 0.026 | -0.081 | 0.026 |
| rs12450225 | 17 | 46592413 | G | A | 0.011 | 0.002 | 0.008 | 0.012 | 0.018 | 0.016 | -0.004 | 0.016 |
| rs9905140 | 17 | 59496238 | T | C | 0.011 | 0.002 | 0.021 | 0.013 | 0.015 | 0.017 | 0.034 | 0.017 |
| rs8075273 | 17 | 61728881 | C | A | 0.014 | 0.002 | -0.017 | 0.014 | -0.001 | 0.018 | -0.038 | 0.017 |
| rs12602912 | 17 | 65870073 | T | C | 0.018 | 0.002 | -0.028 | 0.016 | -0.005 | 0.020 | -0.049 | 0.020 |
| rs9897538 | 17 | 68432122 | A | C | 0.016 | 0.002 | -0.022 | 0.012 | -0.033 | 0.016 | -0.006 | 0.016 |
| rs9988 | 17 | 73230856 | T | C | 0.015 | 0.002 | 0.006 | 0.016 | 0.014 | 0.021 | 0.009 | 0.021 |
| rs10512605 | 17 | 74212382 | T | C | 0.023 | 0.003 | -0.020 | 0.023 | -0.040 | 0.029 | 0.030 | 0.029 |
| rs4239275 | 17 | 79923718 | T | C | 0.012 | 0.002 | 0.002 | 0.014 | 0.016 | 0.018 | -0.009 | 0.017 |
| rs3810068 | 18 | 2846499 | T | C | 0.016 | 0.002 | 0.000 | 0.014 | 0.009 | 0.018 | -0.008 | 0.019 |
| rs1787013 | 18 | 13072979 | C | T | 0.011 | 0.002 | 0.017 | 0.012 | 0.033 | 0.016 | 0.012 | 0.016 |
| rs62095889 | 18 | 21069068 | G | A | 0.017 | 0.002 | 0.002 | 0.013 | 0.019 | 0.017 | -0.015 | 0.017 |
| rs10164099 | 18 | 34690744 | C | T | 0.015 | 0.003 | -0.016 | 0.018 | -0.007 | 0.023 | -0.015 | 0.023 |
| rs8096564 | 18 | 39910592 | T | G | 0.011 | 0.002 | 0.011 | 0.014 | 0.005 | 0.017 | 0.010 | 0.017 |
| rs1158805 | 18 | 40736590 | C | A | 0.013 | 0.002 | -0.010 | 0.013 | -0.029 | 0.016 | -0.005 | 0.016 |
| rs9951872 | 18 | 46678832 | A | G | 0.021 | 0.002 | -0.026 | 0.017 | -0.052 | 0.022 | -0.013 | 0.022 |
| rs7235891 | 18 | 53454774 | C | T | 0.012 | 0.002 | -0.023 | 0.012 | -0.030 | 0.016 | -0.022 | 0.016 |
| rs6567160 | 18 | 57829135 | C | T | 0.026 | 0.002 | 0.006 | 0.015 | 0.030 | 0.019 | -0.010 | 0.019 |
| rs112099489 | 18 | 58417964 | C | T | 0.044 | 0.008 | -0.111 | 0.054 | -0.103 | 0.069 | -0.152 | 0.069 |
| rs28434748 | 18 | 63306008 | G | A | 0.015 | 0.002 | 0.021 | 0.015 | 0.046 | 0.020 | 0.008 | 0.020 |
| rs12459350 | 19 | 2176586 | A | G | 0.013 | 0.002 | 0.012 | 0.012 | 0.033 | 0.016 | 0.000 | 0.016 |
| rs1035942 | 19 | 7199803 | A | G | 0.012 | 0.002 | -0.027 | 0.014 | -0.013 | 0.018 | -0.035 | 0.018 |
| rs12608504 | 19 | 18389135 | A | G | 0.025 | 0.002 | 0.021 | 0.013 | 0.011 | 0.017 | 0.038 | 0.016 |
| rs4808845 | 19 | 18812024 | A | G | 0.012 | 0.002 | 0.002 | 0.013 | 0.008 | 0.017 | 0.003 | 0.016 |
| rs998732 | 19 | 19378671 | A | G | 0.017 | 0.002 | -0.043 | 0.017 | -0.037 | 0.021 | -0.064 | 0.021 |
| rs7257330 | 19 | 30301823 | A | G | 0.013 | 0.002 | -0.021 | 0.013 | -0.012 | 0.017 | -0.038 | 0.016 |
| rs3786897 | 19 | 33893008 | G | A | 0.024 | 0.002 | 0.004 | 0.013 | 0.013 | 0.016 | -0.010 | 0.016 |
| rs12461964 | 19 | 41341229 | G | A | 0.013 | 0.002 | 0.003 | 0.013 | -0.005 | 0.017 | 0.008 | 0.017 |
| rs429358 | 19 | 45411941 | T | C | 0.035 | 0.003 | -0.010 | 0.019 | -0.005 | 0.024 | 0.007 | 0.024 |

| | | | | | | | | | | | | | |
|---|---|---|---|---|---|---|---|---|---|---|---|---|---|
| | rs11672660 | 19 | 46180184 | C | T | 0.021 | 0.002 | 0.019 | 0.016 | 0.021 | 0.021 | 0.027 | 0.020 |
| | rs3810291 | 19 | 47569003 | A | G | 0.012 | 0.002 | -0.025 | 0.013 | 0.006 | 0.017 | -0.060 | 0.017 |
| | rs11084399 | 19 | 56000492 | C | T | 0.011 | 0.002 | -0.011 | 0.013 | 0.002 | 0.017 | -0.023 | 0.017 |
| | rs805770 | 20 | 5668714 | T | C | 0.018 | 0.002 | -0.009 | 0.013 | 0.021 | 0.017 | -0.032 | 0.017 |
| | rs979012 | 20 | 6623374 | T | C | 0.012 | 0.002 | -0.024 | 0.013 | -0.018 | 0.017 | -0.022 | 0.017 |
| | rs7267979 | 20 | 25298087 | A | G | 0.010 | 0.002 | -0.003 | 0.013 | 0.030 | 0.016 | -0.026 | 0.016 |
| | rs143384 | 20 | 34025756 | A | G | 0.016 | 0.002 | -0.015 | 0.013 | -0.004 | 0.017 | -0.028 | 0.016 |
| | rs4812700 | 20 | 41989051 | C | T | 0.018 | 0.003 | 0.023 | 0.019 | 0.034 | 0.024 | 0.022 | 0.024 |
| | rs2236519 | 20 | 45529571 | A | G | 0.021 | 0.002 | 0.022 | 0.013 | 0.019 | 0.017 | 0.028 | 0.016 |
| | rs3092781 | 20 | 45789953 | C | T | 0.015 | 0.002 | -0.001 | 0.012 | 0.004 | 0.016 | -0.010 | 0.016 |
| | rs6021889 | 20 | 50982870 | A | G | 0.020 | 0.002 | 0.026 | 0.014 | 0.043 | 0.018 | 0.011 | 0.018 |
| | rs910382 | 20 | 51699189 | G | A | 0.017 | 0.002 | -0.003 | 0.012 | 0.008 | 0.016 | -0.018 | 0.016 |
| | rs1328757 | 20 | 56135199 | T | C | 0.011 | 0.002 | -0.011 | 0.012 | 0.005 | 0.016 | -0.021 | 0.016 |
| | rs28451064 | 21 | 35593827 | A | G | 0.018 | 0.003 | -0.007 | 0.019 | 0.019 | 0.025 | -0.011 | 0.024 |
| | rs2836179 | 21 | 39544159 | G | A | 0.013 | 0.002 | 0.028 | 0.013 | 0.044 | 0.016 | 0.013 | 0.016 |
| | rs2838006 | 21 | 42653567 | C | T | 0.011 | 0.002 | 0.019 | 0.013 | 0.049 | 0.017 | -0.001 | 0.017 |
| | rs510197 | 22 | 27584316 | G | A | 0.015 | 0.002 | 0.002 | 0.016 | 0.000 | 0.021 | 0.010 | 0.020 |
| | rs2294239 | 22 | 29449477 | A | G | 0.020 | 0.002 | 0.028 | 0.013 | 0.047 | 0.016 | 0.012 | 0.016 |
| | rs713770 | 22 | 35667975 | A | G | 0.012 | 0.002 | -0.058 | 0.013 | -0.033 | 0.017 | -0.076 | 0.017 |
| | rs8141715 | 22 | 47214749 | G | T | 0.014 | 0.002 | 0.002 | 0.014 | 0.005 | 0.018 | -0.008 | 0.018 |
| Body fat percentage | rs12124126 | 1 | 6660349 | A | G | 0.012 | 0.002 | -0.017 | 0.013 | -0.013 | 0.017 | -0.018 | 0.017 |
| | rs4908677 | 1 | 7738180 | T | C | 0.011 | 0.002 | -0.011 | 0.013 | -0.010 | 0.016 | -0.015 | 0.016 |
| | rs159961 | 1 | 8484228 | T | C | 0.012 | 0.002 | 0.027 | 0.013 | 0.037 | 0.017 | 0.022 | 0.017 |
| | rs1318408 | 1 | 11925781 | G | A | 0.016 | 0.003 | -0.035 | 0.020 | -0.026 | 0.025 | -0.040 | 0.025 |
| | rs3766823 | 1 | 32197257 | A | G | 0.017 | 0.002 | -0.022 | 0.017 | -0.016 | 0.022 | -0.029 | 0.021 |
| | rs2050256 | 1 | 32204683 | G | A | 0.016 | 0.002 | -0.025 | 0.017 | -0.021 | 0.022 | -0.031 | 0.021 |
| | rs6686901 | 1 | 42436054 | C | T | 0.012 | 0.002 | -0.002 | 0.012 | 0.000 | 0.016 | 0.001 | 0.016 |
| | rs138556772 | 1 | 46174888 | G | A | 0.027 | 0.004 | -0.049 | 0.034 | -0.020 | 0.044 | -0.059 | 0.043 |
| | rs1167309 | 1 | 49997674 | C | T | 0.014 | 0.002 | -0.049 | 0.013 | -0.043 | 0.017 | -0.053 | 0.017 |
| | rs12566626 | 1 | 50499636 | T | C | 0.013 | 0.002 | -0.048 | 0.013 | -0.041 | 0.017 | -0.050 | 0.017 |
| | rs1013293 | 1 | 62570321 | G | A | 0.014 | 0.002 | -0.009 | 0.013 | 0.001 | 0.016 | -0.014 | 0.016 |
| | rs12140153 | 1 | 62579891 | G | T | 0.025 | 0.003 | 0.042 | 0.024 | 0.071 | 0.031 | 0.026 | 0.030 |
| | rs2186120 | 1 | 66453163 | A | G | 0.010 | 0.002 | -0.010 | 0.012 | -0.018 | 0.016 | 0.001 | 0.016 |
| | rs2815764 | 1 | 72754123 | G | A | 0.020 | 0.002 | 0.015 | 0.016 | 0.010 | 0.021 | 0.003 | 0.020 |
| | rs12039708 | 1 | 77947009 | T | C | 0.016 | 0.002 | -0.029 | 0.016 | -0.059 | 0.021 | 0.010 | 0.021 |
| | rs34517439 | 1 | 78450517 | A | C | 0.021 | 0.003 | -0.068 | 0.021 | -0.125 | 0.027 | -0.013 | 0.026 |
| | rs6688826 | 1 | 80812329 | C | T | 0.011 | 0.002 | 0.018 | 0.014 | 0.035 | 0.018 | 0.014 | 0.017 |
| | rs2181375 | 1 | 96940119 | G | A | 0.015 | 0.002 | -0.008 | 0.013 | -0.013 | 0.016 | -0.008 | 0.016 |
| | rs12072739 | 1 | 98315893 | G | A | 0.014 | 0.002 | 0.004 | 0.015 | 0.013 | 0.019 | -0.009 | 0.019 |
| | rs2077569 | 1 | 103350876 | G | A | 0.011 | 0.002 | 0.004 | 0.013 | -0.005 | 0.016 | 0.014 | 0.016 |
| | rs1730858 | 1 | 107619244 | T | C | 0.011 | 0.002 | -0.046 | 0.013 | -0.025 | 0.017 | -0.057 | 0.017 |
| | rs17024393 | 1 | 110154688 | C | T | 0.047 | 0.006 | -0.057 | 0.039 | -0.058 | 0.050 | -0.055 | 0.049 |
| | rs2306937 | 1 | 113246506 | C | T | 0.015 | 0.002 | -0.052 | 0.015 | -0.067 | 0.020 | -0.030 | 0.020 |
| | rs11205303 | 1 | 149906413 | C | T | 0.017 | 0.002 | 0.004 | 0.013 | 0.013 | 0.017 | -0.004 | 0.017 |
| | rs143453062 | 1 | 150340182 | A | G | 0.031 | 0.004 | -0.027 | 0.030 | -0.019 | 0.038 | -0.028 | 0.038 |
| | rs114529840 | 1 | 150457517 | T | G | 0.031 | 0.004 | -0.033 | 0.031 | -0.031 | 0.039 | -0.024 | 0.039 |
| | rs146468719 | 1 | 151000790 | A | C | 0.013 | 0.002 | 0.059 | 0.017 | 0.062 | 0.021 | 0.050 | 0.021 |
| | rs35154152 | 1 | 155172725 | T | C | 0.020 | 0.003 | -0.027 | 0.020 | -0.027 | 0.026 | -0.037 | 0.026 |
| | rs61813324 | 1 | 156049877 | T | C | 0.017 | 0.003 | -0.012 | 0.020 | -0.014 | 0.026 | -0.013 | 0.026 |
| | rs148137538 | 1 | 173399677 | A | G | 0.034 | 0.006 | -0.024 | 0.045 | -0.065 | 0.057 | 0.021 | 0.058 |
| | rs77560793 | 1 | 175001179 | G | A | 0.031 | 0.005 | 0.000 | 0.037 | -0.031 | 0.047 | 0.020 | 0.047 |

| | | | | | | | | | | | | |
|---|---|---|---|---|---|---|---|---|---|---|---|---|
| rs543874 | 1 | 177889480 | G | A | 0.028 | 0.002 | -0.027 | 0.016 | -0.004 | 0.020 | -0.045 | 0.020 |
| rs9425633 | 1 | 184657251 | C | T | 0.012 | 0.002 | -0.014 | 0.012 | -0.016 | 0.016 | -0.018 | 0.016 |
| rs672313 | 1 | 195142845 | G | A | 0.014 | 0.002 | 0.004 | 0.015 | 0.005 | 0.020 | 0.008 | 0.019 |
| rs2678204 | 1 | 201800511 | G | T | 0.014 | 0.002 | 0.020 | 0.013 | 0.019 | 0.017 | 0.022 | 0.017 |
| rs11119208 | 1 | 209211968 | A | G | 0.011 | 0.002 | 0.004 | 0.013 | 0.007 | 0.016 | 0.004 | 0.016 |
| rs78508049 | 1 | 210344884 | C | T | 0.014 | 0.002 | 0.011 | 0.016 | -0.018 | 0.020 | 0.038 | 0.020 |
| rs2494196 | 1 | 219762581 | A | C | 0.022 | 0.002 | 0.021 | 0.014 | 0.006 | 0.018 | 0.046 | 0.018 |
| rs12133169 | 1 | 219792380 | A | G | 0.018 | 0.002 | 0.019 | 0.015 | -0.002 | 0.020 | 0.043 | 0.019 |
| rs12042959 | 1 | 243533273 | A | G | 0.016 | 0.002 | -0.019 | 0.018 | 0.003 | 0.023 | -0.020 | 0.023 |
| rs62106258 | 2 | 417167 | T | C | 0.049 | 0.004 | 0.011 | 0.032 | 0.042 | 0.042 | -0.021 | 0.040 |
| rs13393304 | 2 | 637830 | G | A | 0.028 | 0.002 | -0.023 | 0.016 | -0.010 | 0.021 | -0.031 | 0.021 |
| rs11096549 | 2 | 16607101 | T | C | 0.012 | 0.002 | -0.007 | 0.014 | -0.008 | 0.018 | -0.017 | 0.018 |
| rs141240885 | 2 | 24449850 | T | G | 0.024 | 0.004 | 0.001 | 0.027 | 0.026 | 0.036 | -0.028 | 0.034 |
| rs6752378 | 2 | 25150116 | A | C | 0.022 | 0.002 | 0.080 | 0.012 | 0.106 | 0.016 | 0.057 | 0.016 |
| rs76286777 | 2 | 25195577 | C | T | 0.023 | 0.002 | 0.100 | 0.015 | 0.125 | 0.020 | 0.077 | 0.019 |
| rs1731260 | 2 | 26953354 | T | G | 0.012 | 0.002 | 0.002 | 0.012 | -0.020 | 0.016 | 0.020 | 0.016 |
| rs11678385 | 2 | 30492847 | G | A | 0.010 | 0.002 | 0.027 | 0.013 | 0.045 | 0.016 | 0.012 | 0.016 |
| rs10172196 | 2 | 36780549 | A | G | 0.013 | 0.002 | -0.006 | 0.014 | 0.005 | 0.017 | -0.002 | 0.017 |
| rs10169594 | 2 | 41637688 | C | T | 0.013 | 0.002 | 0.032 | 0.013 | 0.037 | 0.017 | 0.032 | 0.017 |
| rs113019802 | 2 | 46884824 | G | A | 0.014 | 0.002 | -0.009 | 0.015 | 0.011 | 0.019 | -0.023 | 0.019 |
| rs2436772 | 2 | 47283557 | G | A | 0.015 | 0.002 | 0.014 | 0.015 | 0.033 | 0.020 | 0.003 | 0.020 |
| rs13406839 | 2 | 50735433 | A | G | 0.011 | 0.002 | 0.003 | 0.013 | -0.004 | 0.016 | 0.001 | 0.016 |
| rs13387836 | 2 | 55278559 | T | C | 0.012 | 0.002 | 0.000 | 0.014 | 0.034 | 0.018 | -0.025 | 0.017 |
| rs1559556 | 2 | 57332617 | G | A | 0.011 | 0.002 | 0.013 | 0.013 | 0.016 | 0.017 | 0.009 | 0.016 |
| rs7608397 | 2 | 58769042 | G | T | 0.011 | 0.002 | 0.015 | 0.013 | 0.026 | 0.016 | 0.005 | 0.016 |
| rs11125768 | 2 | 59306564 | T | C | 0.015 | 0.002 | 0.002 | 0.013 | 0.010 | 0.016 | 0.002 | 0.016 |
| rs6739755 | 2 | 59330227 | A | G | 0.015 | 0.002 | 0.006 | 0.013 | 0.014 | 0.016 | 0.007 | 0.016 |
| rs12477088 | 2 | 67841326 | T | C | 0.012 | 0.002 | 0.026 | 0.013 | 0.009 | 0.016 | 0.053 | 0.016 |
| rs3552 | 2 | 69698158 | A | G | 0.013 | 0.002 | -0.004 | 0.013 | -0.011 | 0.016 | -0.009 | 0.016 |
| rs12619178 | 2 | 100838157 | C | T | 0.014 | 0.002 | 0.002 | 0.013 | -0.018 | 0.016 | 0.011 | 0.016 |
| rs6730157 | 2 | 135907088 | A | G | 0.013 | 0.002 | 0.022 | 0.015 | 0.016 | 0.019 | 0.028 | 0.019 |
| rs1446585 | 2 | 136407479 | A | G | 0.015 | 0.002 | 0.011 | 0.015 | 0.017 | 0.019 | 0.007 | 0.019 |
| rs10181181 | 2 | 161087411 | C | T | 0.012 | 0.002 | -0.025 | 0.014 | -0.024 | 0.018 | -0.020 | 0.017 |
| rs6717858 | 2 | 165539661 | C | T | 0.018 | 0.002 | 0.007 | 0.013 | 0.008 | 0.016 | 0.009 | 0.016 |
| rs12477385 | 2 | 166144850 | G | T | 0.012 | 0.002 | -0.023 | 0.015 | -0.022 | 0.020 | -0.021 | 0.019 |
| rs4668314 | 2 | 171631258 | G | T | 0.010 | 0.002 | 0.019 | 0.013 | 0.020 | 0.016 | 0.010 | 0.016 |
| rs2129475 | 2 | 172916772 | G | A | 0.011 | 0.002 | 0.005 | 0.013 | 0.021 | 0.017 | -0.018 | 0.016 |
| rs79869125 | 2 | 176422238 | G | T | 0.019 | 0.003 | 0.020 | 0.020 | 0.012 | 0.026 | 0.021 | 0.026 |
| rs12622267 | 2 | 181568007 | A | G | 0.013 | 0.002 | 0.007 | 0.013 | 0.014 | 0.017 | -0.003 | 0.017 |
| rs7570258 | 2 | 193791720 | C | T | 0.010 | 0.002 | -0.002 | 0.012 | -0.009 | 0.016 | -0.006 | 0.016 |
| rs2043016 | 2 | 198146381 | T | C | 0.011 | 0.002 | 0.027 | 0.013 | 0.048 | 0.017 | 0.003 | 0.017 |
| rs4482463 | 2 | 205375909 | C | A | 0.022 | 0.003 | 0.010 | 0.023 | 0.004 | 0.030 | 0.006 | 0.029 |
| rs2712169 | 2 | 217671349 | G | A | 0.011 | 0.002 | -0.010 | 0.013 | -0.009 | 0.016 | -0.007 | 0.016 |
| rs2943650 | 2 | 227105921 | C | T | 0.015 | 0.002 | 0.017 | 0.013 | 0.011 | 0.017 | 0.024 | 0.016 |
| rs4321353 | 2 | 229003882 | T | G | 0.014 | 0.002 | -0.017 | 0.013 | -0.003 | 0.017 | -0.026 | 0.017 |
| rs10498240 | 2 | 230734531 | A | C | 0.016 | 0.002 | -0.009 | 0.013 | -0.007 | 0.017 | -0.010 | 0.017 |
| rs62246314 | 3 | 9504099 | A | G | 0.017 | 0.003 | 0.012 | 0.020 | 0.017 | 0.026 | 0.010 | 0.026 |
| rs4684847 | 3 | 12386337 | T | C | 0.030 | 0.003 | 0.007 | 0.019 | -0.008 | 0.025 | 0.015 | 0.024 |
| rs7649970 | 3 | 12392272 | T | C | 0.031 | 0.003 | 0.006 | 0.019 | -0.008 | 0.025 | 0.014 | 0.024 |
| rs4684848 | 3 | 12395645 | A | G | 0.030 | 0.003 | 0.003 | 0.019 | -0.009 | 0.025 | 0.010 | 0.024 |
| rs4619804 | 3 | 18674644 | C | A | 0.013 | 0.002 | 0.039 | 0.014 | 0.047 | 0.018 | 0.045 | 0.018 |

| | | | | | | | | | | | | |
|---|---|---|---|---|---|---|---|---|---|---|---|---|
| rs13062093 | 3 | 35667057 | G | T | 0.012 | 0.002 | 0.010 | 0.013 | 0.017 | 0.016 | 0.000 | 0.016 |
| rs1348252 | 3 | 42418752 | C | T | 0.012 | 0.002 | 0.009 | 0.014 | -0.001 | 0.019 | 0.010 | 0.018 |
| rs7637852 | 3 | 44041777 | A | G | 0.013 | 0.002 | 0.000 | 0.014 | 0.019 | 0.017 | -0.021 | 0.017 |
| rs62259939 | 3 | 49386047 | A | G | 0.013 | 0.002 | -0.101 | 0.013 | -0.140 | 0.016 | -0.060 | 0.016 |
| rs73079014 | 3 | 49863483 | C | T | 0.015 | 0.003 | -0.130 | 0.019 | -0.130 | 0.025 | -0.135 | 0.024 |
| rs3774581 | 3 | 53802748 | A | G | 0.011 | 0.002 | 0.021 | 0.014 | 0.002 | 0.017 | 0.038 | 0.017 |
| rs17639546 | 3 | 61251635 | G | A | 0.014 | 0.002 | 0.016 | 0.018 | -0.008 | 0.023 | 0.038 | 0.023 |
| rs9968060 | 3 | 62471282 | T | C | 0.012 | 0.002 | -0.030 | 0.013 | -0.025 | 0.017 | -0.035 | 0.017 |
| rs66815886 | 3 | 64703394 | T | G | 0.012 | 0.002 | 0.004 | 0.014 | -0.006 | 0.018 | 0.005 | 0.018 |
| rs7630228 | 3 | 71681487 | T | C | 0.011 | 0.002 | 0.005 | 0.013 | 0.009 | 0.017 | 0.002 | 0.016 |
| rs9856109 | 3 | 82563862 | T | C | 0.011 | 0.002 | 0.009 | 0.013 | 0.004 | 0.017 | 0.007 | 0.017 |
| rs114712833 | 3 | 84139974 | C | T | 0.021 | 0.004 | -0.001 | 0.024 | -0.013 | 0.031 | -0.004 | 0.031 |
| rs3911063 | 3 | 85906928 | T | C | 0.013 | 0.002 | -0.032 | 0.013 | -0.042 | 0.017 | -0.030 | 0.017 |
| rs35714284 | 3 | 93529866 | G | A | 0.012 | 0.002 | 0.004 | 0.013 | -0.004 | 0.017 | 0.004 | 0.017 |
| rs1609906 | 3 | 94033599 | G | A | 0.016 | 0.002 | 0.016 | 0.012 | 0.023 | 0.016 | 0.002 | 0.016 |
| rs10934646 | 3 | 123084541 | A | G | 0.013 | 0.002 | 0.014 | 0.013 | 0.020 | 0.017 | 0.017 | 0.016 |
| rs9820766 | 3 | 123264017 | C | T | 0.012 | 0.002 | -0.026 | 0.013 | -0.024 | 0.017 | -0.031 | 0.017 |
| rs9816797 | 3 | 131614595 | G | A | 0.014 | 0.002 | -0.020 | 0.014 | -0.004 | 0.018 | -0.030 | 0.018 |
| rs7635592 | 3 | 131718029 | T | C | 0.016 | 0.002 | 0.008 | 0.015 | 0.013 | 0.020 | 0.019 | 0.020 |
| rs2042864 | 3 | 141178979 | C | T | 0.012 | 0.002 | 0.036 | 0.013 | 0.066 | 0.016 | 0.011 | 0.016 |
| rs1568489 | 3 | 153673681 | G | A | 0.012 | 0.002 | 0.008 | 0.013 | 0.033 | 0.016 | -0.008 | 0.016 |
| rs8192675 | 3 | 170724883 | C | T | 0.011 | 0.002 | -0.009 | 0.014 | -0.009 | 0.018 | -0.011 | 0.017 |
| rs12635614 | 3 | 173113041 | A | G | 0.010 | 0.002 | -0.001 | 0.013 | 0.011 | 0.016 | -0.014 | 0.016 |
| rs2606228 | 3 | 183537759 | A | C | 0.012 | 0.002 | -0.002 | 0.013 | -0.004 | 0.017 | 0.002 | 0.017 |
| rs9867130 | 3 | 185729753 | G | A | 0.016 | 0.003 | -0.005 | 0.018 | -0.008 | 0.024 | 0.002 | 0.023 |
| rs2192527 | 4 | 18329824 | G | A | 0.014 | 0.002 | -0.009 | 0.012 | -0.001 | 0.016 | -0.020 | 0.016 |
| rs28602597 | 4 | 20112947 | A | G | 0.019 | 0.003 | 0.011 | 0.019 | 0.012 | 0.024 | 0.002 | 0.024 |
| rs34811474 | 4 | 25408838 | G | A | 0.014 | 0.002 | 0.032 | 0.016 | 0.047 | 0.021 | 0.030 | 0.021 |
| rs73213501 | 4 | 28514830 | A | C | 0.015 | 0.002 | 0.010 | 0.016 | 0.018 | 0.021 | 0.002 | 0.021 |
| rs10938398 | 4 | 45186139 | A | G | 0.019 | 0.002 | -0.008 | 0.013 | -0.006 | 0.016 | -0.005 | 0.016 |
| rs2102278 | 4 | 52818664 | G | A | 0.011 | 0.002 | 0.007 | 0.014 | 0.018 | 0.018 | 0.009 | 0.017 |
| rs6840236 | 4 | 56289785 | C | T | 0.012 | 0.002 | 0.003 | 0.012 | -0.003 | 0.016 | 0.004 | 0.016 |
| rs2318543 | 4 | 67803263 | A | G | 0.012 | 0.002 | 0.024 | 0.015 | 0.019 | 0.019 | 0.025 | 0.019 |
| rs58125425 | 4 | 73550383 | T | C | 0.023 | 0.004 | -0.017 | 0.026 | -0.041 | 0.034 | 0.004 | 0.033 |
| rs12503232 | 4 | 80911468 | A | C | 0.013 | 0.002 | 0.024 | 0.014 | 0.029 | 0.018 | 0.029 | 0.018 |
| rs7692359 | 4 | 83209346 | T | C | 0.013 | 0.002 | 0.011 | 0.015 | 0.022 | 0.019 | 0.002 | 0.019 |
| rs3796658 | 4 | 89708241 | G | A | 0.012 | 0.002 | 0.000 | 0.012 | -0.001 | 0.016 | 0.007 | 0.016 |
| rs1229984 | 4 | 100239319 | C | T | 0.035 | 0.006 | 0.011 | 0.033 | 0.004 | 0.042 | 0.040 | 0.043 |
| rs13126505 | 4 | 102865304 | A | G | 0.030 | 0.003 | 0.112 | 0.024 | 0.170 | 0.031 | 0.079 | 0.031 |
| rs13107325 | 4 | 103188709 | T | C | 0.037 | 0.003 | 0.113 | 0.023 | 0.201 | 0.028 | 0.033 | 0.030 |
| rs11099020 | 4 | 130724902 | C | T | 0.011 | 0.002 | -0.030 | 0.013 | -0.027 | 0.017 | -0.028 | 0.017 |
| rs1296328 | 4 | 137083193 | A | C | 0.010 | 0.002 | 0.011 | 0.013 | 0.004 | 0.017 | 0.015 | 0.016 |
| rs7720791 | 5 | 50361133 | G | A | 0.011 | 0.002 | -0.011 | 0.014 | 0.008 | 0.017 | -0.027 | 0.017 |
| rs6861649 | 5 | 50864788 | C | T | 0.011 | 0.002 | -0.007 | 0.013 | -0.010 | 0.016 | -0.011 | 0.016 |
| rs157845 | 5 | 55796639 | T | C | 0.012 | 0.002 | 0.002 | 0.014 | -0.010 | 0.019 | 0.020 | 0.018 |
| rs7736910 | 5 | 63040773 | G | A | 0.012 | 0.002 | 0.005 | 0.013 | 0.014 | 0.016 | -0.014 | 0.016 |
| rs6893495 | 5 | 64409378 | T | C | 0.013 | 0.002 | 0.018 | 0.015 | 0.037 | 0.020 | 0.003 | 0.020 |
| rs249612 | 5 | 66200783 | T | C | 0.012 | 0.002 | 0.015 | 0.014 | -0.001 | 0.018 | 0.027 | 0.018 |
| rs4976033 | 5 | 67714246 | A | G | 0.012 | 0.002 | -0.017 | 0.013 | -0.010 | 0.017 | -0.012 | 0.017 |
| rs4704187 | 5 | 74480288 | T | C | 0.014 | 0.002 | 0.009 | 0.013 | -0.011 | 0.017 | 0.027 | 0.016 |
| rs13356670 | 5 | 74673707 | A | G | 0.018 | 0.002 | 0.011 | 0.015 | 0.007 | 0.019 | 0.012 | 0.019 |

| | | | | | | | | | | | | |
|---|---|---|---|---|---|---|---|---|---|---|---|---|
| rs2307111 | 5 | 75003678 | T | C | 0.017 | 0.002 | 0.009 | 0.013 | -0.011 | 0.016 | 0.030 | 0.016 |
| rs59893724 | 5 | 80830788 | A | G | 0.013 | 0.002 | 0.007 | 0.015 | 0.013 | 0.019 | -0.002 | 0.019 |
| rs34580448 | 5 | 82810884 | T | C | 0.032 | 0.004 | 0.018 | 0.031 | 0.027 | 0.040 | -0.012 | 0.040 |
| rs11951885 | 5 | 86727566 | C | T | 0.036 | 0.006 | -0.030 | 0.040 | -0.043 | 0.051 | -0.023 | 0.051 |
| rs6870983 | 5 | 87697533 | C | T | 0.014 | 0.002 | 0.007 | 0.015 | 0.025 | 0.019 | -0.003 | 0.019 |
| rs34483452 | 5 | 87986314 | A | C | 0.024 | 0.003 | -0.028 | 0.018 | -0.006 | 0.023 | -0.042 | 0.023 |
| rs59399491 | 5 | 92560816 | G | A | 0.013 | 0.002 | 0.008 | 0.016 | -0.010 | 0.020 | 0.027 | 0.020 |
| rs11135450 | 5 | 95554016 | G | A | 0.011 | 0.002 | 0.024 | 0.013 | 0.004 | 0.017 | 0.034 | 0.017 |
| rs254024 | 5 | 103944020 | T | G | 0.010 | 0.002 | 0.013 | 0.012 | 0.002 | 0.016 | 0.017 | 0.016 |
| rs40067 | 5 | 107439012 | G | A | 0.017 | 0.002 | 0.022 | 0.016 | 0.027 | 0.021 | 0.007 | 0.021 |
| rs4502882 | 5 | 153093998 | C | T | 0.012 | 0.002 | 0.000 | 0.013 | 0.029 | 0.017 | -0.028 | 0.017 |
| rs2964481 | 5 | 157892530 | T | C | 0.015 | 0.002 | 0.022 | 0.014 | 0.034 | 0.018 | 0.005 | 0.018 |
| rs245775 | 5 | 170532105 | G | A | 0.013 | 0.002 | -0.015 | 0.014 | -0.005 | 0.018 | -0.020 | 0.018 |
| rs10947793 | 6 | 12142817 | A | G | 0.012 | 0.002 | 0.001 | 0.013 | -0.015 | 0.017 | 0.021 | 0.017 |
| rs1042317 | 6 | 20492995 | T | C | 0.011 | 0.002 | -0.022 | 0.014 | -0.044 | 0.018 | -0.006 | 0.018 |
| rs75499503 | 6 | 26145217 | C | T | 0.025 | 0.002 | -0.043 | 0.015 | -0.023 | 0.020 | -0.053 | 0.019 |
| rs7766641 | 6 | 26184102 | G | A | 0.024 | 0.002 | -0.012 | 0.014 | 0.020 | 0.018 | -0.031 | 0.018 |
| rs72843644 | 6 | 27211601 | A | C | 0.021 | 0.003 | -0.006 | 0.022 | 0.038 | 0.029 | -0.039 | 0.028 |
| rs6902687 | 6 | 28413491 | C | T | 0.010 | 0.002 | -0.023 | 0.013 | 0.006 | 0.017 | -0.051 | 0.017 |
| rs62395827 | 6 | 31786730 | C | T | 0.023 | 0.003 | 0.009 | 0.022 | -0.061 | 0.028 | 0.064 | 0.028 |
| rs1061801 | 6 | 33282338 | A | G | 0.014 | 0.002 | -0.011 | 0.016 | -0.021 | 0.021 | -0.002 | 0.020 |
| rs59137082 | 6 | 33732365 | C | T | 0.012 | 0.002 | -0.013 | 0.014 | -0.006 | 0.019 | -0.025 | 0.018 |
| rs2744956 | 6 | 34618937 | C | T | 0.025 | 0.003 | -0.039 | 0.018 | -0.032 | 0.023 | -0.052 | 0.023 |
| rs9469887 | 6 | 34758940 | T | C | 0.021 | 0.002 | -0.017 | 0.013 | 0.002 | 0.017 | -0.028 | 0.017 |
| rs2064317 | 6 | 35477032 | A | G | 0.015 | 0.002 | 0.043 | 0.013 | 0.044 | 0.017 | 0.034 | 0.017 |
| rs9471333 | 6 | 40362023 | C | T | 0.016 | 0.002 | 0.026 | 0.013 | 0.018 | 0.016 | 0.040 | 0.016 |
| rs73737608 | 6 | 50376763 | G | A | 0.023 | 0.004 | 0.051 | 0.028 | 0.057 | 0.037 | 0.039 | 0.036 |
| rs4715208 | 6 | 50829471 | G | A | 0.017 | 0.002 | -0.015 | 0.014 | -0.021 | 0.018 | -0.020 | 0.018 |
| rs1928185 | 6 | 50935513 | C | T | 0.023 | 0.002 | 0.005 | 0.017 | 0.000 | 0.021 | 0.008 | 0.021 |
| rs1414506 | 6 | 51487416 | T | C | 0.011 | 0.002 | -0.003 | 0.013 | 0.003 | 0.017 | -0.010 | 0.017 |
| rs13191298 | 6 | 70010225 | G | A | 0.016 | 0.003 | 0.028 | 0.019 | 0.039 | 0.024 | 0.016 | 0.024 |
| rs62422090 | 6 | 97944199 | A | G | 0.012 | 0.002 | -0.006 | 0.014 | -0.030 | 0.018 | 0.011 | 0.018 |
| rs9320823 | 6 | 98429337 | C | T | 0.017 | 0.002 | 0.001 | 0.013 | -0.009 | 0.016 | 0.000 | 0.016 |
| rs9375188 | 6 | 98555272 | C | T | 0.017 | 0.002 | 0.008 | 0.012 | 0.012 | 0.016 | -0.005 | 0.016 |
| rs314279 | 6 | 105402083 | C | A | 0.016 | 0.003 | 0.019 | 0.020 | 0.009 | 0.026 | 0.020 | 0.026 |
| rs6927268 | 6 | 108865663 | T | G | 0.014 | 0.002 | -0.010 | 0.015 | -0.020 | 0.020 | -0.004 | 0.020 |
| rs9400479 | 6 | 111826959 | G | T | 0.014 | 0.002 | -0.009 | 0.016 | 0.007 | 0.021 | -0.027 | 0.020 |
| rs111743285 | 6 | 127048230 | T | C | 0.016 | 0.002 | 0.059 | 0.015 | 0.067 | 0.020 | 0.052 | 0.019 |
| rs72959041 | 6 | 127454893 | G | A | 0.025 | 0.004 | 0.026 | 0.031 | -0.018 | 0.039 | 0.090 | 0.039 |
| rs9321191 | 6 | 130165691 | T | C | 0.013 | 0.002 | 0.008 | 0.015 | -0.001 | 0.020 | 0.020 | 0.020 |
| rs72995085 | 6 | 143193971 | T | C | 0.014 | 0.002 | -0.010 | 0.016 | -0.022 | 0.021 | 0.001 | 0.021 |
| rs4709745 | 6 | 164105984 | C | T | 0.011 | 0.002 | 0.015 | 0.013 | 0.013 | 0.017 | 0.010 | 0.017 |
| rs2529050 | 7 | 24595823 | C | T | 0.011 | 0.002 | 0.006 | 0.014 | 0.021 | 0.018 | -0.004 | 0.017 |
| rs10259620 | 7 | 27202289 | A | G | 0.016 | 0.002 | 0.062 | 0.015 | 0.046 | 0.020 | 0.083 | 0.019 |
| rs10264581 | 7 | 27255417 | G | A | 0.025 | 0.004 | 0.041 | 0.026 | 0.054 | 0.034 | 0.032 | 0.033 |
| rs215669 | 7 | 32378979 | G | A | 0.011 | 0.002 | 0.005 | 0.013 | 0.001 | 0.016 | -0.004 | 0.016 |
| rs4549685 | 7 | 39326478 | C | T | 0.013 | 0.002 | -0.004 | 0.013 | -0.002 | 0.017 | 0.005 | 0.017 |
| rs2289379 | 7 | 44804225 | C | T | 0.012 | 0.002 | -0.041 | 0.013 | -0.042 | 0.017 | -0.032 | 0.016 |
| rs6948959 | 7 | 50697051 | G | A | 0.012 | 0.002 | -0.003 | 0.015 | -0.013 | 0.019 | 0.008 | 0.019 |
| rs4718964 | 7 | 70038969 | T | G | 0.012 | 0.002 | -0.010 | 0.013 | -0.014 | 0.016 | -0.003 | 0.016 |
| rs6955671 | 7 | 74489486 | C | T | 0.011 | 0.002 | -0.063 | 0.016 | -0.075 | 0.020 | -0.055 | 0.020 |

| | | | | | | | | | | | | |
|---|---|---|---|---|---|---|---|---|---|---|---|---|
| rs62477684 | 7 | 75101427 | C | T | 0.013 | 0.002 | -0.041 | 0.013 | -0.035 | 0.017 | -0.053 | 0.017 |
| rs3901286 | 7 | 99107727 | C | A | 0.016 | 0.002 | -0.019 | 0.017 | -0.014 | 0.022 | -0.025 | 0.022 |
| rs3847072 | 7 | 105091519 | G | A | 0.012 | 0.002 | -0.017 | 0.016 | -0.013 | 0.021 | -0.027 | 0.021 |
| rs6946860 | 7 | 112953475 | A | G | 0.013 | 0.002 | -0.009 | 0.013 | -0.042 | 0.017 | 0.020 | 0.017 |
| rs80172389 | 7 | 114432036 | G | T | 0.018 | 0.003 | 0.010 | 0.022 | 0.039 | 0.028 | -0.002 | 0.028 |
| rs972283 | 7 | 130466854 | A | G | 0.013 | 0.002 | 0.027 | 0.013 | 0.025 | 0.016 | 0.035 | 0.016 |
| rs6977416 | 7 | 150542711 | G | A | 0.012 | 0.002 | -0.013 | 0.013 | -0.022 | 0.017 | 0.004 | 0.017 |
| rs11782341 | 8 | 4813459 | G | A | 0.014 | 0.002 | -0.010 | 0.016 | -0.007 | 0.020 | -0.017 | 0.020 |
| rs13268133 | 8 | 9731470 | T | C | 0.012 | 0.002 | 0.002 | 0.013 | 0.010 | 0.017 | -0.006 | 0.017 |
| rs11786089 | 8 | 21975521 | G | A | 0.012 | 0.002 | -0.005 | 0.013 | -0.013 | 0.016 | -0.002 | 0.016 |
| rs11781222 | 8 | 23389571 | T | C | 0.017 | 0.003 | 0.032 | 0.018 | -0.005 | 0.023 | 0.059 | 0.023 |
| rs59104534 | 8 | 25666169 | T | C | 0.011 | 0.002 | -0.026 | 0.014 | -0.016 | 0.018 | -0.039 | 0.018 |
| rs2725370 | 8 | 30852826 | T | C | 0.013 | 0.002 | 0.025 | 0.013 | 0.026 | 0.017 | 0.018 | 0.017 |
| rs1808629 | 8 | 73435964 | G | A | 0.016 | 0.002 | -0.018 | 0.013 | -0.005 | 0.017 | -0.026 | 0.017 |
| rs2977345 | 8 | 76725867 | C | T | 0.015 | 0.002 | -0.003 | 0.014 | -0.003 | 0.017 | -0.014 | 0.017 |
| rs10100245 | 8 | 77226919 | A | G | 0.015 | 0.002 | 0.003 | 0.013 | 0.007 | 0.016 | -0.007 | 0.016 |
| rs879256 | 8 | 78893942 | A | G | 0.011 | 0.002 | 0.002 | 0.013 | 0.000 | 0.017 | -0.005 | 0.016 |
| rs2721963 | 8 | 116661174 | A | C | 0.014 | 0.002 | -0.008 | 0.013 | -0.011 | 0.017 | -0.001 | 0.017 |
| rs4876611 | 8 | 116671848 | G | A | 0.016 | 0.002 | -0.018 | 0.014 | -0.024 | 0.018 | -0.006 | 0.018 |
| rs4466418 | 8 | 126323787 | A | G | 0.011 | 0.002 | 0.016 | 0.013 | 0.011 | 0.016 | 0.020 | 0.016 |
| rs2954033 | 8 | 126493746 | G | A | 0.011 | 0.002 | 0.026 | 0.014 | 0.062 | 0.017 | 0.003 | 0.017 |
| rs10959841 | 9 | 11469190 | T | C | 0.011 | 0.002 | 0.009 | 0.013 | 0.019 | 0.016 | -0.002 | 0.016 |
| rs7046483 | 9 | 14777395 | G | A | 0.010 | 0.002 | 0.022 | 0.013 | 0.017 | 0.016 | 0.032 | 0.016 |
| rs10962016 | 9 | 15389970 | G | A | 0.024 | 0.004 | -0.025 | 0.027 | -0.027 | 0.035 | -0.019 | 0.035 |
| rs13292699 | 9 | 15910044 | A | C | 0.021 | 0.002 | 0.008 | 0.013 | 0.009 | 0.016 | 0.009 | 0.016 |
| rs1415475 | 9 | 16591502 | T | C | 0.024 | 0.004 | 0.021 | 0.025 | -0.015 | 0.032 | 0.035 | 0.032 |
| rs10756798 | 9 | 16739763 | C | T | 0.014 | 0.002 | 0.033 | 0.013 | 0.033 | 0.017 | 0.040 | 0.017 |
| rs17770336 | 9 | 28414625 | T | C | 0.015 | 0.002 | 0.017 | 0.013 | 0.024 | 0.017 | 0.015 | 0.017 |
| rs7848702 | 9 | 31210051 | T | C | 0.012 | 0.002 | 0.001 | 0.014 | 0.005 | 0.018 | 0.009 | 0.018 |
| rs10973160 | 9 | 36994969 | C | T | 0.011 | 0.002 | -0.011 | 0.014 | -0.018 | 0.018 | 0.001 | 0.018 |
| rs12339822 | 9 | 92187178 | G | A | 0.014 | 0.002 | -0.010 | 0.013 | -0.020 | 0.016 | 0.000 | 0.016 |
| rs10820739 | 9 | 99263821 | A | G | 0.013 | 0.002 | 0.001 | 0.016 | 0.000 | 0.021 | -0.006 | 0.020 |
| rs11012732 | 10 | 21830104 | G | A | 0.018 | 0.002 | 0.025 | 0.013 | 0.015 | 0.017 | 0.022 | 0.017 |
| rs7078183 | 10 | 70367452 | C | A | 0.010 | 0.002 | -0.010 | 0.013 | 0.008 | 0.016 | -0.021 | 0.016 |
| rs10999456 | 10 | 72413827 | T | C | 0.016 | 0.002 | 0.008 | 0.014 | 0.008 | 0.018 | 0.004 | 0.018 |
| rs10999460 | 10 | 72428283 | T | C | 0.016 | 0.002 | 0.005 | 0.014 | 0.006 | 0.018 | 0.003 | 0.018 |
| rs2002023 | 10 | 76848524 | T | C | 0.012 | 0.002 | 0.007 | 0.013 | 0.013 | 0.016 | -0.003 | 0.016 |
| rs11594905 | 10 | 77659733 | A | G | 0.015 | 0.003 | 0.039 | 0.021 | -0.010 | 0.027 | 0.060 | 0.026 |
| rs10887582 | 10 | 88110792 | C | T | 0.010 | 0.002 | -0.003 | 0.013 | -0.017 | 0.016 | 0.006 | 0.016 |
| rs11187838 | 10 | 96038686 | G | A | 0.014 | 0.002 | -0.020 | 0.013 | -0.009 | 0.016 | -0.021 | 0.016 |
| rs4110517 | 10 | 96650328 | G | A | 0.012 | 0.002 | -0.017 | 0.015 | -0.029 | 0.019 | -0.009 | 0.019 |
| rs577525 | 10 | 99769388 | C | T | 0.014 | 0.002 | 0.023 | 0.013 | 0.053 | 0.016 | 0.006 | 0.016 |
| rs41310284 | 10 | 102447647 | C | A | 0.018 | 0.003 | -0.007 | 0.022 | 0.019 | 0.028 | -0.030 | 0.027 |
| rs6585201 | 10 | 114768783 | G | A | 0.011 | 0.002 | -0.003 | 0.013 | -0.010 | 0.016 | -0.001 | 0.016 |
| rs1225404 | 10 | 114914665 | C | T | 0.011 | 0.002 | 0.003 | 0.014 | 0.010 | 0.018 | 0.001 | 0.018 |
| rs10510025 | 10 | 118650996 | T | C | 0.013 | 0.002 | -0.040 | 0.014 | -0.047 | 0.018 | -0.039 | 0.018 |
| rs4752182 | 10 | 120397131 | A | G | 0.010 | 0.002 | 0.011 | 0.013 | 0.006 | 0.016 | 0.013 | 0.016 |
| rs12218858 | 10 | 126474200 | T | C | 0.010 | 0.002 | 0.049 | 0.013 | 0.056 | 0.016 | 0.038 | 0.016 |
| rs2172131 | 10 | 133978962 | T | C | 0.011 | 0.002 | -0.006 | 0.013 | -0.008 | 0.016 | -0.009 | 0.016 |
| rs11042030 | 11 | 8690718 | T | C | 0.013 | 0.002 | 0.005 | 0.014 | 0.028 | 0.018 | -0.019 | 0.018 |
| rs11042725 | 11 | 10325325 | A | C | 0.011 | 0.002 | -0.001 | 0.013 | 0.001 | 0.016 | 0.007 | 0.016 |

| | | | | | | | | | | | | |
|---|---|---|---|---|---|---|---|---|---|---|---|---|
| rs10767659 | 11 | 27686196 | G | T | 0.020 | 0.002 | 0.002 | 0.013 | -0.011 | 0.017 | 0.004 | 0.017 |
| rs11030108 | 11 | 27695464 | A | G | 0.021 | 0.002 | -0.004 | 0.014 | -0.021 | 0.018 | 0.002 | 0.017 |
| rs71474196 | 11 | 46977160 | C | T | 0.016 | 0.003 | -0.038 | 0.020 | -0.055 | 0.026 | -0.017 | 0.026 |
| rs7124681 | 11 | 47529947 | A | C | 0.022 | 0.002 | 0.016 | 0.013 | 0.026 | 0.016 | 0.012 | 0.016 |
| rs12146571 | 11 | 48314077 | C | T | 0.014 | 0.002 | 0.021 | 0.018 | 0.005 | 0.022 | 0.044 | 0.023 |
| rs477895 | 11 | 64048912 | T | C | 0.015 | 0.002 | -0.019 | 0.017 | -0.025 | 0.022 | -0.007 | 0.021 |
| rs801742 | 11 | 65914766 | C | A | 0.015 | 0.002 | 0.005 | 0.013 | 0.027 | 0.017 | -0.014 | 0.016 |
| rs1213257 | 11 | 85220773 | C | T | 0.014 | 0.002 | 0.000 | 0.017 | 0.029 | 0.022 | -0.024 | 0.021 |
| rs61903695 | 11 | 89922417 | G | A | 0.012 | 0.002 | 0.010 | 0.014 | 0.020 | 0.019 | 0.006 | 0.018 |
| rs3802851 | 11 | 112912550 | T | C | 0.012 | 0.002 | 0.017 | 0.014 | 0.027 | 0.018 | 0.006 | 0.018 |
| rs719802 | 11 | 113234679 | T | C | 0.011 | 0.002 | 0.001 | 0.013 | -0.009 | 0.017 | 0.000 | 0.016 |
| rs4545564 | 11 | 118937518 | C | T | 0.011 | 0.002 | 0.003 | 0.013 | 0.009 | 0.016 | 0.001 | 0.016 |
| rs10791109 | 11 | 130850377 | G | T | 0.011 | 0.002 | -0.005 | 0.012 | 0.015 | 0.016 | -0.018 | 0.016 |
| rs55726687 | 12 | 991306 | A | G | 0.014 | 0.002 | 0.032 | 0.015 | 0.033 | 0.020 | 0.036 | 0.020 |
| rs765123 | 12 | 2155997 | G | A | 0.012 | 0.002 | -0.018 | 0.016 | -0.018 | 0.020 | -0.014 | 0.020 |
| rs7296615 | 12 | 3350679 | A | G | 0.021 | 0.003 | 0.006 | 0.022 | 0.012 | 0.028 | 0.003 | 0.028 |
| rs12367809 | 12 | 50256063 | T | C | 0.019 | 0.002 | 0.016 | 0.013 | 0.014 | 0.017 | 0.018 | 0.016 |
| rs7132908 | 12 | 50263148 | A | G | 0.019 | 0.002 | 0.013 | 0.013 | 0.012 | 0.017 | 0.011 | 0.016 |
| rs4759318 | 12 | 54420098 | T | C | 0.011 | 0.002 | 0.008 | 0.013 | 0.036 | 0.017 | -0.012 | 0.017 |
| rs704061 | 12 | 89771903 | C | T | 0.014 | 0.002 | -0.008 | 0.012 | 0.021 | 0.016 | -0.035 | 0.016 |
| rs12813149 | 12 | 90142637 | G | A | 0.015 | 0.002 | -0.012 | 0.014 | -0.026 | 0.019 | -0.002 | 0.018 |
| rs7975788 | 12 | 90273927 | G | T | 0.012 | 0.002 | -0.034 | 0.014 | -0.039 | 0.018 | -0.027 | 0.018 |
| rs10777259 | 12 | 91248014 | C | T | 0.010 | 0.002 | -0.011 | 0.012 | -0.022 | 0.016 | -0.001 | 0.016 |
| rs59066241 | 12 | 97925364 | G | T | 0.016 | 0.003 | 0.014 | 0.019 | 0.023 | 0.025 | 0.011 | 0.024 |
| rs11113445 | 12 | 108088682 | G | A | 0.012 | 0.002 | 0.041 | 0.013 | 0.021 | 0.016 | 0.057 | 0.016 |
| rs11609659 | 12 | 108296260 | T | C | 0.014 | 0.002 | -0.016 | 0.015 | 0.012 | 0.019 | -0.045 | 0.019 |
| rs3764002 | 12 | 108618630 | C | T | 0.016 | 0.002 | 0.021 | 0.014 | 0.041 | 0.018 | 0.003 | 0.018 |
| rs10492229 | 12 | 110602173 | T | C | 0.012 | 0.002 | 0.002 | 0.015 | 0.003 | 0.019 | 0.001 | 0.019 |
| rs61945850 | 12 | 120846213 | G | A | 0.032 | 0.005 | -0.013 | 0.035 | -0.005 | 0.046 | -0.018 | 0.044 |
| rs76929617 | 12 | 120867798 | A | G | 0.032 | 0.005 | -0.054 | 0.042 | -0.051 | 0.053 | -0.053 | 0.054 |
| rs75412871 | 12 | 121709430 | C | T | 0.025 | 0.004 | -0.042 | 0.028 | -0.018 | 0.037 | -0.057 | 0.036 |
| rs116861199 | 12 | 122464941 | G | A | 0.025 | 0.003 | 0.055 | 0.028 | 0.034 | 0.035 | 0.102 | 0.036 |
| rs35249105 | 12 | 122507810 | G | A | 0.011 | 0.002 | -0.011 | 0.013 | -0.001 | 0.016 | -0.022 | 0.016 |
| rs147730268 | 12 | 123024476 | G | T | 0.032 | 0.003 | 0.023 | 0.024 | 0.039 | 0.031 | 0.041 | 0.030 |
| rs10773394 | 12 | 123124778 | A | C | 0.013 | 0.002 | 0.015 | 0.013 | 0.012 | 0.017 | 0.024 | 0.017 |
| rs147530811 | 12 | 123817477 | C | A | 0.030 | 0.005 | 0.073 | 0.042 | 0.069 | 0.051 | 0.111 | 0.054 |
| rs7133378 | 12 | 124409502 | A | G | 0.019 | 0.002 | -0.038 | 0.013 | -0.030 | 0.017 | -0.040 | 0.017 |
| rs825452 | 12 | 124509177 | G | A | 0.015 | 0.002 | 0.011 | 0.013 | 0.006 | 0.017 | 0.024 | 0.016 |
| rs11619393 | 13 | 20262266 | C | T | 0.015 | 0.002 | -0.010 | 0.018 | -0.015 | 0.023 | -0.019 | 0.023 |
| rs1928496 | 13 | 31012904 | T | C | 0.012 | 0.002 | 0.000 | 0.015 | 0.004 | 0.019 | -0.002 | 0.018 |
| rs9568867 | 13 | 54107352 | A | G | 0.017 | 0.003 | -0.003 | 0.019 | 0.018 | 0.024 | -0.023 | 0.024 |
| rs7982447 | 13 | 54453811 | C | T | 0.015 | 0.002 | -0.023 | 0.015 | -0.035 | 0.020 | -0.015 | 0.019 |
| rs7319102 | 13 | 58252801 | G | A | 0.013 | 0.002 | -0.001 | 0.015 | 0.004 | 0.019 | 0.004 | 0.019 |
| rs11839227 | 13 | 59280705 | T | C | 0.014 | 0.002 | 0.003 | 0.016 | 0.007 | 0.021 | -0.013 | 0.020 |
| rs1218307 | 13 | 79417832 | G | A | 0.011 | 0.002 | 0.009 | 0.013 | 0.014 | 0.016 | 0.008 | 0.016 |
| rs1441264 | 13 | 79580919 | A | G | 0.012 | 0.002 | -0.024 | 0.013 | -0.032 | 0.017 | -0.017 | 0.016 |
| rs6491427 | 13 | 99113166 | A | G | 0.015 | 0.002 | 0.030 | 0.014 | 0.035 | 0.018 | 0.021 | 0.018 |
| rs35413307 | 13 | 112191778 | G | T | 0.012 | 0.002 | 0.001 | 0.013 | -0.021 | 0.017 | 0.019 | 0.017 |
| rs9522279 | 13 | 112221296 | T | C | 0.013 | 0.002 | 0.015 | 0.013 | 0.004 | 0.016 | 0.017 | 0.016 |
| rs4981693 | 14 | 29680331 | A | G | 0.014 | 0.002 | -0.014 | 0.015 | -0.012 | 0.019 | -0.018 | 0.019 |
| rs1959430 | 14 | 30184162 | C | T | 0.010 | 0.002 | 0.018 | 0.013 | 0.019 | 0.016 | 0.020 | 0.016 |

| | | | | | | | | | | | | |
|---|---|---|---|---|---|---|---|---|---|---|---|---|
| rs61979560 | 14 | 30727033 | A | C | 0.012 | 0.002 | -0.002 | 0.015 | 0.009 | 0.019 | -0.022 | 0.019 |
| rs2239647 | 14 | 33292743 | A | C | 0.013 | 0.002 | -0.012 | 0.013 | -0.011 | 0.017 | -0.017 | 0.016 |
| rs72681698 | 14 | 51207741 | T | C | 0.050 | 0.008 | 0.015 | 0.071 | 0.116 | 0.092 | -0.020 | 0.089 |
| rs61975142 | 14 | 59402590 | G | A | 0.015 | 0.002 | 0.035 | 0.017 | 0.025 | 0.021 | 0.041 | 0.021 |
| rs12890931 | 14 | 69753369 | G | T | 0.012 | 0.002 | 0.000 | 0.013 | 0.015 | 0.017 | -0.016 | 0.016 |
| rs2370982 | 14 | 79890677 | T | C | 0.019 | 0.002 | 0.032 | 0.015 | 0.017 | 0.020 | 0.039 | 0.019 |
| rs1075472 | 14 | 93108131 | A | G | 0.015 | 0.002 | 0.024 | 0.016 | 0.047 | 0.021 | 0.008 | 0.020 |
| rs6575340 | 14 | 94023972 | A | G | 0.015 | 0.002 | 0.004 | 0.013 | 0.004 | 0.017 | -0.001 | 0.016 |
| rs3803286 | 14 | 103246470 | A | G | 0.013 | 0.002 | 0.017 | 0.013 | 0.006 | 0.017 | 0.036 | 0.017 |
| rs8042404 | 15 | 31680016 | A | G | 0.012 | 0.002 | -0.002 | 0.014 | 0.011 | 0.018 | -0.014 | 0.018 |
| rs28538451 | 15 | 41455769 | A | C | 0.013 | 0.002 | -0.047 | 0.014 | 0.009 | 0.018 | -0.091 | 0.018 |
| rs3751585 | 15 | 51741056 | A | G | 0.013 | 0.002 | 0.013 | 0.016 | 0.030 | 0.020 | -0.009 | 0.020 |
| rs117632017 | 15 | 52260107 | A | G | 0.031 | 0.005 | 0.025 | 0.038 | 0.035 | 0.050 | 0.046 | 0.048 |
| rs2165991 | 15 | 53101700 | G | A | 0.012 | 0.002 | 0.006 | 0.014 | 0.015 | 0.018 | -0.003 | 0.018 |
| rs17291497 | 15 | 67362994 | C | T | 0.010 | 0.002 | 0.012 | 0.013 | 0.047 | 0.016 | -0.018 | 0.016 |
| rs7166081 | 15 | 67492301 | A | G | 0.015 | 0.002 | 0.047 | 0.015 | 0.053 | 0.019 | 0.046 | 0.019 |
| rs16951304 | 15 | 68089618 | T | C | 0.020 | 0.002 | -0.001 | 0.015 | 0.003 | 0.020 | -0.001 | 0.019 |
| rs7164727 | 15 | 73093991 | T | C | 0.015 | 0.002 | -0.017 | 0.013 | -0.004 | 0.017 | -0.027 | 0.017 |
| rs2660824 | 15 | 73618309 | C | T | 0.011 | 0.002 | -0.002 | 0.013 | -0.001 | 0.016 | -0.010 | 0.016 |
| rs34769775 | 15 | 80989172 | C | T | 0.011 | 0.002 | 0.012 | 0.014 | 0.034 | 0.018 | -0.002 | 0.018 |
| rs6602997 | 15 | 84521398 | T | C | 0.022 | 0.002 | -0.015 | 0.014 | -0.022 | 0.018 | -0.005 | 0.017 |
| rs2135877 | 15 | 84663107 | G | A | 0.021 | 0.002 | 0.003 | 0.013 | -0.007 | 0.017 | 0.010 | 0.017 |
| rs62021171 | 15 | 85128550 | G | A | 0.016 | 0.002 | -0.034 | 0.016 | -0.047 | 0.020 | -0.018 | 0.020 |
| rs1879529 | 15 | 89414295 | G | T | 0.015 | 0.002 | -0.005 | 0.014 | -0.010 | 0.019 | -0.001 | 0.018 |
| rs57221746 | 15 | 99230035 | G | A | 0.012 | 0.002 | 0.015 | 0.015 | 0.024 | 0.019 | 0.009 | 0.019 |
| rs72755233 | 15 | 100692953 | G | A | 0.017 | 0.003 | 0.024 | 0.022 | 0.011 | 0.028 | 0.026 | 0.028 |
| rs412243 | 16 | 339672 | T | C | 0.012 | 0.002 | -0.007 | 0.013 | 0.006 | 0.017 | -0.024 | 0.016 |
| rs181179291 | 16 | 2787062 | C | A | 0.054 | 0.008 | 0.067 | 0.068 | 0.126 | 0.091 | 0.072 | 0.084 |
| rs879620 | 16 | 4015729 | T | C | 0.016 | 0.002 | -0.032 | 0.013 | -0.038 | 0.017 | -0.029 | 0.017 |
| rs4785955 | 16 | 4297651 | T | G | 0.013 | 0.002 | 0.000 | 0.015 | 0.011 | 0.020 | -0.015 | 0.020 |
| rs7193783 | 16 | 4949608 | A | C | 0.011 | 0.002 | 0.025 | 0.013 | 0.034 | 0.017 | 0.009 | 0.017 |
| rs4474693 | 16 | 20246313 | A | G | 0.017 | 0.003 | 0.047 | 0.018 | 0.062 | 0.024 | 0.028 | 0.023 |
| rs200534 | 16 | 24752641 | A | G | 0.015 | 0.002 | 0.040 | 0.014 | 0.048 | 0.018 | 0.038 | 0.018 |
| rs9939450 | 16 | 28301487 | T | C | 0.014 | 0.002 | 0.032 | 0.013 | 0.029 | 0.017 | 0.026 | 0.016 |
| rs56186137 | 16 | 28825953 | G | A | 0.023 | 0.002 | 0.094 | 0.013 | 0.113 | 0.016 | 0.073 | 0.016 |
| rs72798148 | 16 | 29926552 | T | C | 0.018 | 0.002 | -0.004 | 0.017 | -0.025 | 0.022 | 0.009 | 0.022 |
| rs9746755 | 16 | 30519628 | G | A | 0.010 | 0.002 | 0.006 | 0.014 | 0.003 | 0.017 | 0.010 | 0.017 |
| rs34898535 | 16 | 31025641 | C | T | 0.016 | 0.002 | 0.002 | 0.013 | -0.001 | 0.017 | 0.001 | 0.016 |
| rs11642015 | 16 | 53802494 | T | C | 0.040 | 0.002 | 0.026 | 0.013 | 0.038 | 0.016 | 0.013 | 0.016 |
| rs7202116 | 16 | 53821615 | G | A | 0.039 | 0.002 | 0.024 | 0.013 | 0.040 | 0.016 | 0.008 | 0.016 |
| rs9940346 | 16 | 64716851 | C | T | 0.011 | 0.002 | 0.002 | 0.013 | -0.021 | 0.017 | 0.019 | 0.016 |
| rs11866219 | 16 | 69549749 | A | C | 0.017 | 0.002 | 0.032 | 0.013 | 0.026 | 0.016 | 0.036 | 0.016 |
| rs889398 | 16 | 69556715 | C | T | 0.016 | 0.002 | 0.024 | 0.013 | 0.025 | 0.016 | 0.026 | 0.016 |
| rs7191938 | 16 | 71407530 | G | A | 0.012 | 0.002 | 0.018 | 0.014 | -0.006 | 0.017 | 0.033 | 0.017 |
| rs2012817 | 16 | 72038659 | C | T | 0.011 | 0.002 | -0.028 | 0.013 | -0.057 | 0.017 | 0.002 | 0.017 |
| rs4411525 | 16 | 74595502 | A | C | 0.010 | 0.002 | -0.031 | 0.013 | -0.031 | 0.016 | -0.030 | 0.016 |
| rs55637757 | 16 | 89535888 | C | T | 0.018 | 0.003 | 0.009 | 0.019 | 0.040 | 0.025 | -0.017 | 0.024 |
| rs77733403 | 16 | 90080723 | T | C | 0.015 | 0.002 | -0.005 | 0.018 | -0.014 | 0.023 | 0.002 | 0.022 |
| rs8082551 | 17 | 1310661 | C | T | 0.017 | 0.003 | 0.080 | 0.020 | 0.064 | 0.026 | 0.103 | 0.025 |
| rs4790841 | 17 | 1835482 | C | T | 0.022 | 0.002 | -0.015 | 0.018 | -0.001 | 0.023 | -0.029 | 0.023 |
| rs34356467 | 17 | 3978531 | T | G | 0.014 | 0.002 | 0.001 | 0.016 | 0.023 | 0.021 | -0.015 | 0.021 |

| | | | | | | | | | | | | | |
|---|---|---|---|---|---|---|---|---|---|---|---|---|---|
| | rs9902386 | 17 | 21280185 | T | G | 0.012 | 0.002 | -0.020 | 0.014 | -0.019 | 0.018 | -0.019 | 0.017 |
| | rs71371126 | 17 | 28002642 | A | G | 0.012 | 0.002 | 0.005 | 0.013 | -0.014 | 0.017 | 0.023 | 0.017 |
| | rs11872020 | 17 | 28553489 | A | G | 0.013 | 0.002 | 0.014 | 0.013 | -0.012 | 0.017 | 0.033 | 0.017 |
| | rs2855818 | 17 | 42290015 | A | G | 0.017 | 0.002 | -0.029 | 0.015 | -0.062 | 0.019 | -0.007 | 0.019 |
| | rs8074938 | 17 | 43153006 | A | G | 0.011 | 0.002 | 0.025 | 0.013 | 0.031 | 0.017 | 0.028 | 0.017 |
| | rs17698176 | 17 | 44819595 | G | T | 0.012 | 0.002 | 0.006 | 0.016 | 0.019 | 0.021 | -0.017 | 0.021 |
| | rs208015 | 17 | 46252346 | T | C | 0.025 | 0.003 | 0.004 | 0.024 | -0.035 | 0.032 | 0.033 | 0.031 |
| | rs11079852 | 17 | 47095041 | G | A | 0.013 | 0.002 | 0.002 | 0.013 | 0.013 | 0.017 | -0.005 | 0.017 |
| | rs12602556 | 17 | 65826861 | G | A | 0.022 | 0.002 | -0.030 | 0.016 | -0.004 | 0.020 | -0.053 | 0.020 |
| | rs11150745 | 17 | 78757626 | A | G | 0.013 | 0.002 | 0.008 | 0.013 | 0.011 | 0.017 | -0.002 | 0.017 |
| | rs11873650 | 18 | 13163903 | G | A | 0.011 | 0.002 | -0.008 | 0.014 | -0.016 | 0.018 | -0.005 | 0.017 |
| | rs111581974 | 18 | 22205000 | C | T | 0.016 | 0.002 | -0.016 | 0.016 | 0.001 | 0.021 | -0.032 | 0.021 |
| | rs2052607 | 18 | 40788387 | G | A | 0.015 | 0.002 | -0.002 | 0.013 | -0.030 | 0.017 | 0.012 | 0.017 |
| | rs11876574 | 18 | 42595844 | T | C | 0.012 | 0.002 | -0.009 | 0.014 | -0.024 | 0.018 | 0.006 | 0.017 |
| | rs12967878 | 18 | 57826570 | C | T | 0.024 | 0.002 | 0.010 | 0.015 | 0.033 | 0.019 | -0.006 | 0.019 |
| | rs538656 | 18 | 57850422 | T | G | 0.023 | 0.002 | 0.004 | 0.015 | 0.032 | 0.019 | -0.015 | 0.019 |
| | rs111718521 | 18 | 58085845 | G | A | 0.072 | 0.007 | -0.070 | 0.050 | -0.056 | 0.066 | -0.086 | 0.063 |
| | rs193239997 | 18 | 58639547 | T | C | 0.046 | 0.007 | -0.060 | 0.048 | -0.017 | 0.064 | -0.135 | 0.061 |
| | rs350832 | 19 | 4069426 | A | G | 0.015 | 0.002 | -0.047 | 0.015 | -0.076 | 0.020 | -0.026 | 0.020 |
| | rs273507 | 19 | 18221964 | C | A | 0.018 | 0.002 | 0.015 | 0.014 | 0.005 | 0.018 | 0.029 | 0.018 |
| | rs10404726 | 19 | 18834514 | C | T | 0.013 | 0.002 | 0.007 | 0.013 | -0.004 | 0.016 | 0.018 | 0.016 |
| | rs62104483 | 19 | 30300017 | A | G | 0.013 | 0.002 | -0.009 | 0.013 | -0.001 | 0.017 | -0.024 | 0.017 |
| | rs12610925 | 19 | 33880349 | G | A | 0.017 | 0.002 | -0.001 | 0.013 | 0.021 | 0.017 | -0.019 | 0.017 |
| | rs33836 | 19 | 34008600 | C | T | 0.017 | 0.002 | 0.020 | 0.013 | 0.047 | 0.016 | -0.002 | 0.016 |
| | rs6857 | 19 | 45392254 | C | T | 0.016 | 0.002 | -0.016 | 0.018 | -0.015 | 0.023 | 0.003 | 0.023 |
| | rs429358 | 19 | 45411941 | T | C | 0.017 | 0.002 | -0.010 | 0.019 | -0.005 | 0.024 | 0.007 | 0.024 |
| | rs11672660 | 19 | 46180184 | C | T | 0.023 | 0.002 | 0.019 | 0.016 | 0.021 | 0.021 | 0.027 | 0.020 |
| | rs3810291 | 19 | 47569003 | A | G | 0.012 | 0.002 | -0.025 | 0.013 | 0.006 | 0.017 | -0.060 | 0.017 |
| | rs7020 | 20 | 25278600 | A | G | 0.013 | 0.002 | -0.003 | 0.013 | 0.028 | 0.016 | -0.026 | 0.016 |
| | rs819168 | 20 | 32903845 | G | T | 0.021 | 0.004 | -0.030 | 0.025 | 0.003 | 0.032 | -0.064 | 0.031 |
| | rs17265513 | 20 | 39832628 | C | T | 0.013 | 0.002 | 0.061 | 0.016 | 0.054 | 0.021 | 0.080 | 0.020 |
| | rs6103254 | 20 | 41990761 | T | C | 0.016 | 0.003 | 0.025 | 0.019 | 0.034 | 0.024 | 0.026 | 0.024 |
| | rs112852122 | 20 | 47498117 | G | A | 0.018 | 0.002 | -0.002 | 0.018 | 0.039 | 0.023 | -0.033 | 0.023 |
| | rs73142879 | 20 | 51195932 | C | T | 0.015 | 0.002 | 0.013 | 0.016 | 0.020 | 0.021 | 0.003 | 0.020 |
| | rs1056441 | 20 | 62370349 | C | T | 0.013 | 0.002 | 0.117 | 0.014 | 0.126 | 0.018 | 0.100 | 0.017 |
| | rs76040172 | 21 | 46488959 | G | A | 0.028 | 0.004 | -0.010 | 0.029 | -0.033 | 0.038 | -0.004 | 0.036 |
| | rs394608 | 21 | 46581798 | C | T | 0.013 | 0.002 | 0.022 | 0.012 | 0.019 | 0.016 | 0.016 | 0.016 |
| | rs4820325 | 22 | 38599978 | G | A | 0.017 | 0.002 | 0.011 | 0.013 | 0.009 | 0.016 | 0.017 | 0.016 |
| | rs202661 | 22 | 41812439 | A | G | 0.013 | 0.002 | 0.050 | 0.015 | 0.068 | 0.019 | 0.039 | 0.019 |
| | rs10854853 | 22 | 48874412 | T | G | 0.011 | 0.002 | 0.046 | 0.013 | 0.028 | 0.016 | 0.053 | 0.016 |
| Overall physical activity | rs55657917 | 17 | 43844560 | G | T | 0.037 | 0.005 | -0.045 | 0.016 | -0.057 | 0.020 | -0.041 | 0.020 |
| | rs2052607 | 18 | 40788387 | A | G | 0.028 | 0.005 | 0.002 | 0.013 | 0.030 | 0.017 | -0.012 | 0.017 |
| Vitamin D | rs6698680 | 1 | 2329661 | A | G | 0.012 | 0.002 | -0.0108 | 0.0131 | -0.0199 | 0.0169 | -0.0116 | 0.0167 |
| | rs11203338 | 1 | 17559907 | T | C | 0.021 | 0.002 | 0.0194 | 0.0131 | 0.0112 | 0.0169 | 0.0291 | 0.0166 |
| | rs7519574 | 1 | 34726552 | A | G | 0.017 | 0.003 | -0.0069 | 0.0166 | -0.0152 | 0.0215 | 0.0023 | 0.0211 |
| | rs7528419 | 1 | 109817192 | G | A | 0.019 | 0.002 | -0.0111 | 0.015 | -0.0168 | 0.0194 | -0.012 | 0.0191 |
| | rs3768013 | 1 | 150815411 | G | A | 0.015 | 0.002 | 0.0645 | 0.0129 | 0.0557 | 0.0166 | 0.0659 | 0.0164 |
| | rs61816761 | 1 | 152285861 | A | G | 0.125 | 0.007 | 0.0259 | 0.0579 | 0.0557 | 0.0719 | 0.0246 | 0.0743 |
| | rs10796941 | 1 | 155284261 | T | C | 0.018 | 0.002 | 0.076 | 0.0142 | 0.0986 | 0.0183 | 0.0565 | 0.0182 |
| | rs867772 | 1 | 220972343 | A | G | 0.014 | 0.002 | -0.0333 | 0.0136 | -0.0336 | 0.0174 | -0.0331 | 0.0173 |
| | rs12997242 | 2 | 21381177 | G | A | 0.013 | 0.002 | -0.0059 | 0.0125 | -0.0226 | 0.0161 | 0.008 | 0.0159 |

| | | | | | | | | | | | | | |
|---|---|---|---|---|---|---|---|---|---|---|---|---|---|
| | rs11127048 | 2 | 27752463 | A | G | 0.018 | 0.002 | -0.0618 | 0.0129 | -0.0976 | 0.0167 | -0.0362 | 0.0165 |
| | rs6724965 | 2 | 101440151 | A | G | 0.017 | 0.003 | -0.0069 | 0.0163 | -0.0263 | 0.0211 | 0.0057 | 0.0207 |
| | rs7569755 | 2 | 118648261 | A | G | 0.014 | 0.002 | -0.011 | 0.0136 | 0.0062 | 0.0175 | -0.0252 | 0.0174 |
| | rs1047891 | 2 | 211540507 | C | A | 0.014 | 0.002 | 0.0349 | 0.0137 | 0.0629 | 0.0178 | 0.0135 | 0.0175 |
| | rs2011425 | 2 | 234627608 | T | G | 0.046 | 0.004 | -0.0227 | 0.0228 | -0.0522 | 0.0289 | -0.0142 | 0.0291 |
| | rs1551042 | 3 | 85630551 | A | C | 0.018 | 0.002 | -0.0352 | 0.013 | -0.0315 | 0.0167 | -0.0399 | 0.0165 |
| | rs9859616 | 3 | 125149488 | A | G | 0.014 | 0.002 | -0.0207 | 0.0147 | -0.0086 | 0.019 | -0.034 | 0.0187 |
| | rs6773343 | 3 | 141825598 | T | C | 0.013 | 0.002 | -0.0091 | 0.0138 | 0.0091 | 0.0179 | -0.0278 | 0.0176 |
| | rs73080940 | 4 | 3482296 | T | C | 0.018 | 0.003 | 0.0207 | 0.0207 | 0.017 | 0.0268 | 0.0264 | 0.0262 |
| | rs11723621 | 4 | 72615362 | A | G | 0.187 | 0.002 | -0.0164 | 0.0136 | -0.0062 | 0.0176 | -0.0261 | 0.0174 |
| | rs58073039 | 4 | 88287363 | A | G | 0.014 | 0.002 | 0.0257 | 0.0136 | 0.0326 | 0.0175 | 0.0169 | 0.0173 |
| | rs28364331 | 4 | 100201295 | G | A | 0.061 | 0.007 | -0.038 | 0.0476 | -0.0429 | 0.0618 | -0.016 | 0.06 |
| | rs2112325 | 5 | 118651979 | C | T | 0.013 | 0.002 | -0.0038 | 0.013 | 0.0104 | 0.0168 | -0.0222 | 0.0166 |
| | rs28459049 | 7 | 21567331 | C | T | 0.015 | 0.002 | 0.0087 | 0.0154 | -0.0173 | 0.0197 | 0.0301 | 0.0196 |
| | rs1011468 | 7 | 104613791 | G | A | 0.014 | 0.002 | -0.0069 | 0.0125 | -0.0099 | 0.0161 | -0.0134 | 0.0159 |
| | rs1858889 | 7 | 107117447 | C | A | 0.013 | 0.002 | -0.0058 | 0.0124 | -0.0128 | 0.016 | -0.0043 | 0.0157 |
| | rs804280 | 8 | 11612698 | A | C | 0.013 | 0.002 | 0.0071 | 0.0124 | 0.0278 | 0.0161 | -0.0105 | 0.0159 |
| | rs34726834 | 8 | 25889606 | T | C | 0.014 | 0.002 | -0.0302 | 0.0142 | -0.0154 | 0.0183 | -0.0415 | 0.0181 |
| | rs7828742 | 8 | 116960729 | A | G | 0.022 | 0.002 | 0.0236 | 0.0127 | 0.012 | 0.0164 | 0.0322 | 0.0161 |
| | rs10818771 | 9 | 125724054 | A | G | 0.017 | 0.003 | -0.0026 | 0.018 | 0.0117 | 0.023 | -0.0253 | 0.0229 |
| | rs532436 | 9 | 136149830 | G | A | 0.015 | 0.003 | -0.0197 | 0.0158 | -0.0263 | 0.0204 | -0.0101 | 0.0201 |
| | rs10887718 | 10 | 82042624 | C | T | 0.012 | 0.002 | -0.0286 | 0.0124 | -0.0484 | 0.016 | -0.0119 | 0.0158 |
| | rs117913124 | 11 | 14900931 | G | A | 0.354 | 0.006 | -0.026 | 0.0423 | 0.0277 | 0.055 | -0.0661 | 0.0532 |
| | rs523583 | 11 | 66070146 | C | A | 0.012 | 0.002 | 0.0101 | 0.0126 | -0.0036 | 0.0161 | 0.0267 | 0.0161 |
| | rs12803256 | 11 | 71132868 | G | A | 0.1 | 0.002 | 0.0061 | 0.0144 | -0.0126 | 0.0185 | 0.0267 | 0.0182 |
| | rs1149605 | 11 | 76485216 | C | T | 0.019 | 0.003 | 0.0064 | 0.0168 | 0.0391 | 0.0216 | -0.0134 | 0.0215 |
| | rs2847500 | 11 | 120114421 | G | A | 0.021 | 0.003 | 0.0391 | 0.0201 | 0.0078 | 0.0253 | 0.0586 | 0.0259 |
| | rs12317268 | 12 | 21352541 | A | G | 0.019 | 0.003 | 0.0306 | 0.017 | 0.0084 | 0.0218 | 0.035 | 0.0217 |
| | rs9668081 | 12 | 38602911 | T | C | 0.012 | 0.002 | 0.0106 | 0.0123 | 0.0136 | 0.0159 | 0.0131 | 0.0157 |
| | rs10859995 | 12 | 96375682 | T | C | 0.039 | 0.002 | -0.0089 | 0.0125 | -0.0195 | 0.0162 | -0.0051 | 0.016 |
| | rs2144530 | 14 | 39552484 | C | T | 0.032 | 0.003 | -0.0221 | 0.0164 | -0.028 | 0.0211 | -0.0207 | 0.0208 |
| | rs1800588 | 15 | 58723675 | C | T | 0.03 | 0.002 | 0.0251 | 0.0154 | 0.0362 | 0.0198 | 0.0122 | 0.0195 |
| | rs17765311 | 15 | 63789952 | A | C | 0.015 | 0.002 | -0.0278 | 0.013 | -0.0299 | 0.0169 | -0.0218 | 0.0166 |
| | rs62007299 | 15 | 77711719 | G | A | 0.014 | 0.002 | 0.0352 | 0.0135 | 0.0447 | 0.0174 | 0.0371 | 0.0173 |
| | rs7205121 | 16 | 11908776 | C | T | 0.013 | 0.002 | -0.0068 | 0.0143 | 0.0172 | 0.0184 | -0.0319 | 0.0182 |
| | rs77924615 | 16 | 20392332 | G | A | 0.016 | 0.002 | 0.0192 | 0.0162 | -0.0056 | 0.0209 | 0.0497 | 0.0207 |
| | rs1800775 | 16 | 56995236 | C | A | 0.017 | 0.002 | 0.0085 | 0.0125 | 0.0051 | 0.0162 | 0.0083 | 0.016 |
| | rs2909218 | 17 | 66464546 | T | C | 0.017 | 0.002 | -0.0025 | 0.0153 | -0.0267 | 0.0198 | 0.021 | 0.0196 |
| | rs8091117 | 18 | 28919794 | C | A | 0.024 | 0.004 | -0.0226 | 0.0245 | -0.0555 | 0.0314 | 0.0217 | 0.0313 |
| | rs2037511 | 18 | 61366207 | A | G | 0.016 | 0.003 | 0.0206 | 0.0164 | 0.013 | 0.0212 | 0.02 | 0.021 |
| | rs57631352 | 19 | 4338173 | A | G | 0.013 | 0.002 | -0.0229 | 0.0137 | -0.0183 | 0.0177 | -0.0272 | 0.0174 |
| | rs73015021 | 19 | 11192915 | G | A | 0.023 | 0.003 | -0.0181 | 0.0196 | -0.0212 | 0.0251 | 0.0011 | 0.0252 |
| | rs10500209 | 19 | 11979164 | T | C | 0.013 | 0.002 | 0.04 | 0.0139 | 0.0467 | 0.0179 | 0.0357 | 0.0176 |
| | rs58542926 | 19 | 19379549 | T | C | 0.032 | 0.004 | 0.0025 | 0.0239 | 0.027 | 0.0308 | -0.0332 | 0.0305 |
| | rs3814995 | 19 | 36342212 | C | T | 0.015 | 0.002 | 0.0126 | 0.0162 | 0.0213 | 0.0206 | 0.0051 | 0.0208 |
| | rs157595 | 19 | 45425460 | A | G | 0.016 | 0.002 | 0.0311 | 0.0139 | 0.0276 | 0.0177 | 0.0315 | 0.0178 |
| | rs10426 | 19 | 51517798 | A | G | 0.025 | 0.002 | 0.0077 | 0.0154 | 0.0085 | 0.0198 | 7.00E-04 | 0.0197 |
| | rs8103262 | 19 | 53065814 | C | T | 0.013 | 0.002 | -0.0022 | 0.0137 | -2.00E-04 | 0.0179 | 0.0024 | 0.0173 |
| | rs17274750 | 21 | 16353809 | A | C | 0.026 | 0.003 | -0.0706 | 0.0211 | -0.0599 | 0.027 | -0.0573 | 0.0273 |
| | rs960596 | 22 | 41393520 | T | C | 0.012 | 0.002 | -0.0312 | 0.0134 | -0.0408 | 0.0173 | -0.0265 | 0.0171 |
| Vitamin B9 (folate) | rs1801133 | 1 | 11856378 | G | A | 0.115 | 0.008 | -0.036 | 0.013 | -0.046 | 0.017 | -0.022 | 0.017 |

| | | | | | | | | | | | | | |
|---|---|---|---|---|---|---|---|---|---|---|---|---|---|
| | rs652197 | 11 | 71849741 | C | T | 0.069 | 0.010 | 0.042 | 0.019 | 0.059 | 0.025 | 0.021 | 0.025 |
| Vitamin B12 | rs116075662 | 4 | 146574155 | G | A | 0.099 | 0.015 | 0.053 | 0.028 | 0.048 | 0.036 | 0.039 | 0.036 |
| | rs1141321 | 6 | 49412433 | C | T | 0.070 | 0.007 | -0.008 | 0.013 | -0.026 | 0.017 | 0.012 | 0.016 |
| | rs1801222 | 10 | 17156151 | G | A | 0.119 | 0.007 | -0.012 | 0.013 | -0.014 | 0.017 | -0.006 | 0.017 |
| | rs34324219 | 11 | 59623378 | C | A | 0.236 | 0.010 | 0.007 | 0.021 | 0.039 | 0.028 | -0.022 | 0.027 |
| | rs12272669 | 11 | 71392610 | A | G | 0.510 | 0.086 | -0.001 | 0.034 | -0.046 | 0.045 | 0.064 | 0.043 |
| | rs41281112 | 13 | 100518634 | C | T | 0.181 | 0.015 | -0.076 | 0.048 | -0.047 | 0.061 | -0.114 | 0.061 |
| | rs3742801 | 14 | 74759006 | T | C | 0.053 | 0.007 | -0.002 | 0.013 | -0.017 | 0.017 | 0.017 | 0.016 |
| | rs2336573 | 19 | 8367709 | T | C | 0.313 | 0.019 | -0.002 | 0.033 | -0.032 | 0.043 | 0.021 | 0.043 |
| | rs602662 | 19 | 49206985 | A | G | 0.171 | 0.007 | 0.052 | 0.013 | 0.102 | 0.016 | 0.013 | 0.016 |
| | rs1131603 | 22 | 31018975 | C | T | 0.224 | 0.015 | 0.019 | 0.031 | -0.012 | 0.041 | 0.032 | 0.039 |
| Omega-3 fatty acids | rs174546 | 11 | 61569830 | C | T | 0.154 | 0.012 | -0.051 | 0.013 | -0.083 | 0.017 | -0.027 | 0.017 |
| | rs11604424 | 11 | 116651115 | C | T | 0.090 | 0.014 | 0.003 | 0.015 | 0.001 | 0.020 | -0.005 | 0.019 |
| | rs1077835 | 15 | 58723426 | G | A | 0.089 | 0.014 | -0.025 | 0.015 | -0.034 | 0.020 | -0.014 | 0.020 |
| | rs8100204 | 19 | 19393714 | G | A | 0.122 | 0.020 | -0.022 | 0.019 | -0.036 | 0.025 | -0.003 | 0.025 |
| Omega-6 fatty acids | rs191448950 | 1 | 55584844 | G | A | 0.294 | 0.036 | 0.029 | 0.083 | 0.006 | 0.106 | 0.018 | 0.108 |
| | rs4344355 | 1 | 63153953 | C | T | 0.093 | 0.014 | -0.026 | 0.013 | -0.065 | 0.017 | 0.009 | 0.017 |
| | rs144064722 | 4 | 73406173 | G | A | 0.237 | 0.040 | -0.036 | 0.042 | -0.034 | 0.054 | -0.043 | 0.054 |
| | rs79225634 | 5 | 74619639 | T | C | 0.085 | 0.013 | -0.023 | 0.013 | -0.030 | 0.017 | -0.018 | 0.017 |
| | rs3741298 | 11 | 116657561 | C | T | 0.143 | 0.015 | -0.001 | 0.016 | -0.007 | 0.020 | -0.004 | 0.020 |
| | rs1800588 | 15 | 58723675 | T | C | 0.143 | 0.014 | -0.025 | 0.015 | -0.036 | 0.020 | -0.012 | 0.020 |
| | rs112898275 | 19 | 11188850 | T | C | 0.161 | 0.021 | 0.014 | 0.020 | 0.019 | 0.025 | -0.007 | 0.025 |
| | rs7412 | 19 | 45412079 | C | T | 0.272 | 0.028 | 0.037 | 0.024 | 0.011 | 0.030 | 0.054 | 0.030 |
| Monounsaturated fatty acids (mainly omega-9 a | rs115849089 | 8 | 19912370 | G | A | 0.121 | 0.020 | 0.016 | 0.020 | 0.022 | 0.025 | 0.015 | 0.025 |
| | rs3741298 | 11 | 116657561 | C | T | 0.121 | 0.014 | -0.001 | 0.016 | -0.007 | 0.020 | -0.004 | 0.020 |
| | rs1800588 | 15 | 58723675 | T | C | 0.130 | 0.014 | -0.025 | 0.015 | -0.036 | 0.020 | -0.012 | 0.020 |
| | rs56255430 | 19 | 19477877 | A | C | 0.147 | 0.025 | -0.013 | 0.023 | -0.031 | 0.029 | 0.011 | 0.029 |
| Total fatty acids | rs191448950 | 1 | 55584844 | G | A | 0.229 | 0.036 | 0.029 | 0.083 | 0.006 | 0.106 | 0.018 | 0.108 |
| | rs1168041 | 1 | 62960250 | C | T | 0.085 | 0.014 | -0.029 | 0.013 | -0.067 | 0.017 | 0.007 | 0.017 |
| | rs115849089 | 8 | 19912370 | G | A | 0.118 | 0.020 | 0.016 | 0.020 | 0.022 | 0.025 | 0.015 | 0.025 |
| | rs3741298 | 11 | 116657561 | C | T | 0.136 | 0.014 | -0.001 | 0.016 | -0.007 | 0.020 | -0.004 | 0.020 |
| | rs1800588 | 15 | 58723675 | T | C | 0.153 | 0.014 | -0.025 | 0.015 | -0.036 | 0.020 | -0.012 | 0.020 |
| | rs56289821 | 19 | 11188247 | G | A | 0.122 | 0.021 | 0.016 | 0.020 | 0.021 | 0.026 | -0.006 | 0.025 |
| | rs8100204 | 19 | 19393714 | G | A | 0.123 | 0.020 | -0.022 | 0.019 | -0.036 | 0.025 | -0.003 | 0.025 |
| | rs429358 | 19 | 45411941 | C | T | 0.106 | 0.016 | 0.010 | 0.019 | 0.005 | 0.024 | -0.007 | 0.024 |

**Table S2- Results of sensitivity Mendelian randomization methods for observed environmental risk factors on inflammatory bowel diseases (IBD), including Crohn's disease (CD), and ulcerative colitis (UC). OR: Odds ratio. LIC: Lower confidence interval. UCI: Upper confidence interval. P: P value. Phet: P value for SNP heterogeneity**

| | | | Likelihood-based method | | | | | Weigthed median | | | | Modal-based method | | | | MR-Egger Simex approach | | | | | MR-Egger intercept | | | | MR-PRESSO | | |
|---|---|---|---|---|---|---|---|---|---|---|---|---|---|---|---|---|---|---|---|---|---|---|---|---|---|---|---|
| **Phenotype** | **n SNP** | **Disease** | **OR** | **LCI** | **UCI** | **P** | **Phet** | **OR** | **LCI** | **UCI** | **P** | **OR** | **LCI** | **UCI** | **P** | **OR** | **LCI** | **UCI** | **P** | **I2GX** | **Est** | **LCI** | **UCI** | **P** | **P Global** | **P Distortion** | **Outlier SNPs** |
| **Lifestyle** | | | | | | | | | | | | | | | | | | | | | | | | | | | |
| Smoking status (ever vs never) | 331 | IBD | 1.05 | 0.97 | 1.14 | 0.24 | 6.3E-34 | 1.04 | 0.91 | 1.18 | 0.62 | 0.95 | 0.68 | 1.32 | 0.76 | 0.76 | 0.39 | 1.46 | 0.40 | 0.40 | 0.005 | -0.002 | 0.011 | 0.15 | <1e-04 | 0.61 | rs2526390,rs3172494,rs3764351,rs3810496,rs41264285,rs61886926,rs67696533,rs77215829 |
| | | CD | 1.13 | 1.02 | 1.25 | 0.02 | 1.8E-24 | 1.07 | 0.91 | 1.27 | 0.41 | 1.00 | 0.62 | 1.61 | 1.00 | 0.73 | 0.33 | 1.62 | 0.43 | 0.40 | 0.007 | -0.001 | 0.015 | 0.10 | <1e-04 | 0.97 | rs3172494,rs359431,rs3764351,rs41264285,rs61886926,rs8103660 |
| | | UC | 0.99 | 0.90 | 1.10 | 0.88 | 3.9E-15 | 1.09 | 0.93 | 1.28 | 0.30 | 1.22 | 0.75 | 1.98 | 0.43 | 0.75 | 0.36 | 1.56 | 0.44 | 0.40 | 0.004 | -0.004 | 0.012 | 0.34 | <1e-04 | 1.00 | rs17229285,rs2526390 |
| Cigarettes per day | 44 | IBD | 1.04 | 0.88 | 1.22 | 0.67 | 3.1E-07 | 1.01 | 0.78 | 1.31 | 0.94 | 1.03 | 0.82 | 1.29 | 0.80 | 0.83 | 0.52 | 1.33 | 0.43 | 0.96 | 0.007 | -0.001 | 0.014 | 0.07 | 1.0E-04 | 0.19 | rs13253502,rs293534 |
| | | CD | 1.17 | 0.94 | 1.44 | 0.16 | 1.8E-06 | 1.04 | 0.76 | 1.42 | 0.82 | 1.03 | 0.77 | 1.38 | 0.85 | 0.73 | 0.41 | 1.28 | 0.26 | 0.96 | 0.015 | 0.005 | 0.024 | 4.2E-03 | <1e-04 | 0.67 | rs13253502 |
| | | UC | 0.90 | 0.73 | 1.10 | 0.30 | 0.01 | 0.91 | 0.66 | 1.24 | 0.55 | 0.93 | 0.70 | 1.23 | 0.61 | 0.85 | 0.52 | 1.39 | 0.51 | 0.96 | 0.001 | -0.008 | 0.011 | 0.76 | 8.7E-03 | - | - |
| Body-mass index | 816 | IBD | 0.97 | 0.91 | 1.04 | 0.38 | 1.5E-96 | 1.07 | 0.95 | 1.20 | 0.26 | 1.19 | 0.91 | 1.54 | 0.20 | 1.17 | 0.84 | 1.62 | 0.36 | 0.85 | -0.003 | -0.005 | 0.000 | 0.07 | <1e-04 | 0.97 | rs10182181,rs10492861,rs11065987,rs11066188,rs11710798,rs11713193,rs12044597,rs13107325,rs1800668,rs2010281,rs2023671,rs2726039,rs2836754,rs4722672,rs4985155,rs739742,rs7498665,rs7560871,rs8070454 |
| | | CD | 1.11 | 1.02 | 1.20 | 0.02 | 2.4E-71 | 1.26 | 1.09 | 1.45 | 1.6E-03 | 1.49 | 1.09 | 2.03 | 0.01 | 1.38 | 0.93 | 2.07 | 0.11 | 0.85 | -0.003 | -0.007 | 0.001 | 0.10 | <1e-04 | 0.82 | rs10182181,rs10492861,rs11065987,rs11066188,rs11710798,rs11713193,rs12888545,rs13107325,rs1363695,rs17391694,rs1800668,rs2010281,rs2726039,rs350832,rs657452,rs739742,rs7498665,rs7560871,rs8070454,rs849135,rs9901595 |
| | | UC | 0.85 | 0.78 | 0.92 | 5.2E-05 | 1.5E-46 | 0.88 | 0.76 | 1.02 | 0.10 | 0.92 | 0.65 | 1.29 | 0.61 | 1.00 | 0.69 | 1.44 | 0.98 | 0.84 | -0.003 | -0.006 | 0.001 | 0.14 | <1e-04 | 0.47 | rs11065987,rs11066188,rs11713193,rs12044597,rs1800668,rs4722672,rs4985155,rs7498665,rs7535528,rs8070454 |
| Waist-to-hip ratio | 403 | IBD | 1.05 | 0.96 | 1.14 | 0.33 | 3.8E-59 | 0.92 | 0.79 | 1.08 | 0.31 | 0.86 | 0.63 | 1.18 | 0.34 | 0.83 | 0.52 | 1.33 | 0.45 | 0.83 | 0.003 | -0.001 | 0.007 | 0.09 | <1e-04 | 0.19 | rs10019888,rs1017698,rs13316065,rs2008514,rs28445639,rs591939,rs7531656,rs7591387 |
| | | CD | 1.22 | 1.09 | 1.37 | 6.0E-04 | 2.0E-47 | 1.10 | 0.90 | 1.35 | 0.35 | 1.02 | 0.70 | 1.48 | 0.92 | 1.03 | 0.58 | 1.82 | 0.92 | 0.83 | 0.003 | -0.002 | 0.008 | 0.24 | <1e-04 | 0.10 | rs10019888,rs13316065,rs1708302,rs1793892,rs2008514,rs28445639,rs3094621,rs591939,rs7196397,rs7591387 |
| | | UC | 0.90 | 0.81 | 1.01 | 0.07 | 1.3E-26 | 0.87 | 0.72 | 1.04 | 0.13 | 0.78 | 0.50 | 1.23 | 0.29 | 0.72 | 0.43 | 1.20 | 0.20 | 0.83 | 0.003 | -0.002 | 0.008 | 0.26 | <1e-04 | 0.84 | rs1017698,rs11051005,rs2008514,rs2267846,rs713770 |
| Body fat percentage | 379 | IBD | 1.26 | 1.14 | 1.39 | 3.8E-06 | 6.5E-71 | 1.26 | 1.06 | 1.50 | 0.01 | 1.49 | 1.01 | 2.20 | 0.04 | 1.84 | 0.99 | 3.41 | 0.05 | 0.74 | -0.004 | -0.009 | 0.000 | 0.07 | <1e-04 | 0.16 | rs1056441,rs1167309,rs13107325,rs56186137,rs62259939,rs6752378,rs6955671,rs73079014,rs76286777 |
| | | CD | 1.50 | 1.32 | 1.71 | 3.0E-10 | 9.3E-58 | 1.49 | 1.21 | 1.83 | 1.9E-04 | 2.01 | 1.14 | 3.55 | 0.02 | 2.83 | 1.31 | 6.14 | 0.01 | 0.73 | -0.007 | -0.014 | -0.001 | 0.02 | <1e-04 | 0.08 | rs1056441,rs13107325,rs13126505,rs2012817,rs34517439,rs56186137,rs62259939,rs6752378,rs73079014,rs76286777 |
| | | UC | 1.11 | 0.98 | 1.25 | 0.11 | 4.2E-32 | 1.17 | 0.94 | 1.45 | 0.15 | 1.22 | 0.75 | 1.99 | 0.41 | 1.36 | 0.69 | 2.68 | 0.37 | 0.73 | -0.003 | -0.009 | 0.003 | 0.36 | <1e-04 | 0.82 | rs10259620,rs1056441,rs28538451,rs56186137,rs62259939,rs73079014 |
| Overall physical activity | 2 | IBD | 0.51 | 0.27 | 0.98 | 0.04 | 0.05 | 0.53 | 0.27 | 1.05 | 0.07 | 0.54 | 0.29 | 1.02 | 0.31 | | | | | | | | | | | | |
| | | CD | 0.67 | 0.28 | 1.56 | 0.35 | 1.5E-03 | 0.70 | 0.29 | 1.72 | 0.44 | 0.57 | 0.25 | 1.32 | 0.42 | | | | | | | | | | | | |
| | | UC | 0.45 | 0.20 | 1.00 | 0.05 | 0.40 | 0.45 | 0.20 | 1.05 | 0.07 | 0.43 | 0.19 | 0.97 | 0.29 | | | | | | | | | | | | |
| **Nutrients** | | | | | | | | | | | | | | | | | | | | | | | | | | | |
| Vitamin D | 59 | IBD | 0.96 | 0.87 | 1.06 | 0.38 | 4.1E-16 | 0.92 | 0.81 | 1.05 | 0.22 | 0.93 | 0.83 | 1.04 | 0.21 | 0.93 | 0.74 | 1.17 | 0.51 | 0.99 | 0.002 | -0.003 | 0.007 | 0.41 | <1e-04 | 0.77 | rs10796941,rs11127048,rs3768013 |
| | | CD | 0.96 | 0.85 | 1.09 | 0.56 | 4.0E-14 | 0.97 | 0.82 | 1.15 | 0.72 | 0.98 | 0.85 | 1.14 | 0.83 | 0.97 | 0.73 | 1.29 | 0.82 | 0.99 | -3.E-04 | -0.006 | 0.006 | 0.92 | <1e-04 | 0.93 | rs10796941,rs11127048 |
| | | UC | 0.95 | 0.83 | 1.07 | 0.39 | 6.2E-07 | 0.87 | 0.74 | 1.02 | 0.09 | 0.91 | 0.79 | 1.05 | 0.19 | 0.91 | 0.71 | 1.15 | 0.40 | 0.99 | 0.003 | -0.003 | 0.009 | 0.34 | <1e-04 | 0.87 | rs3768013 |
| Vitamin B9 (folate) | 2 | IBD | 0.83 | 0.67 | 1.03 | 0.09 | 2.5E-03 | 0.84 | 0.67 | 1.04 | 0.10 | 0.75 | 0.60 | 0.94 | 0.24 | | | | | | | | | | | | |
| | | CD | 0.80 | 0.61 | 1.04 | 0.10 | 1.1E-03 | 0.80 | 0.61 | 1.06 | 0.12 | 0.70 | 0.52 | 0.92 | 0.24 | | | | | | | | | | | | |
| | | UC | 0.89 | 0.68 | 1.16 | 0.37 | 0.20 | 0.89 | 0.67 | 1.17 | 0.40 | 0.84 | 0.63 | 1.11 | 0.43 | | | | | | | | | | | | |
| Vitamin B12 | 10 | IBD | 1.07 | 1.00 | 1.15 | 0.06 | 0.01 | 1.01 | 0.92 | 1.12 | 0.83 | 1.00 | 0.89 | 1.12 | 0.97 | 1.06 | 0.83 | 1.35 | 0.59 | 0.98 | 0.001 | -0.023 | 0.024 | 0.94 | 0.01 | - | - |
| | | CD | 1.10 | 1.00 | 1.21 | 0.04 | 8.5E-07 | 0.93 | 0.81 | 1.07 | 0.32 | 0.92 | 0.79 | 1.07 | 0.33 | 1.12 | 0.71 | 1.76 | 0.59 | 0.98 | -0.003 | -0.034 | 0.027 | 0.80 | <1e-04 | 0.02 | rs602662 |
| | | UC | 1.06 | 0.97 | 1.15 | 0.21 | 0.41 | 1.08 | 0.97 | 1.21 | 0.15 | 1.09 | 0.96 | 1.24 | 0.21 | 1.04 | 0.85 | 1.26 | 0.69 | 0.98 | 0.005 | -0.025 | 0.035 | 0.72 | 0.45 | - | - |
| Omega-3 fatty acids | 4 | IBD | 0.78 | 0.68 | 0.88 | 1.3E-04 | 0.29 | 0.74 | 0.64 | 0.86 | 6.7E-05 | 0.73 | 0.62 | 0.86 | 0.03 | 0.53 | 0.14 | 1.95 | 0.17 | 0.89 | 0.044 | -0.091 | 0.180 | 0.30 | 0.22 | - | - |
| | | CD | 0.67 | 0.56 | 0.79 | 2.3E-06 | 0.18 | 0.64 | 0.53 | 0.79 | 3.1E-05 | 0.61 | 0.49 | 0.75 | 0.02 | 0.36 | 0.04 | 2.84 | 0.17 | 0.89 | 0.075 | -0.100 | 0.250 | 0.21 | 0.10 | - | - |
| | | UC | 0.88 | 0.75 | 1.03 | 0.11 | 0.90 | 0.85 | 0.71 | 1.01 | 0.07 | 0.84 | 0.69 | 1.04 | 0.20 | 0.77 | 0.41 | 1.45 | 0.22 | 0.89 | 0.016 | -0.157 | 0.188 | 0.74 | 0.86 | - | - |
| Omega-6 fatty acids | 8 | IBD | 0.96 | 0.88 | 1.05 | 0.36 | 0.08 | 1.00 | 0.87 | 1.15 | 0.97 | 1.09 | 0.89 | 1.32 | 0.43 | 1.35 | 1.05 | 1.73 | 0.03 | 0.92 | -0.049 | -0.091 | -0.008 | 0.03 | 0.05 | - | - |
| | | CD | 0.87 | 0.78 | 0.98 | 0.02 | 0.02 | 0.95 | 0.81 | 1.12 | 0.58 | 1.01 | 0.84 | 1.21 | 0.95 | 1.43 | 0.93 | 2.18 | 0.09 | 0.91 | -0.071 | -0.124 | -0.018 | 0.02 | 0.01 | - | - |
| | | UC | 1.01 | 0.90 | 1.13 | 0.88 | 0.57 | 0.97 | 0.84 | 1.13 | 0.71 | 0.95 | 0.75 | 1.20 | 0.68 | 1.22 | 0.88 | 1.71 | 0.19 | 0.92 | -0.027 | -0.080 | 0.026 | 0.26 | 0.46 | - | - |
| Monounsaturated fatty acids (mainly omeg | 4 | IBD | 0.94 | 0.82 | 1.08 | 0.38 | 0.42 | 0.94 | 0.80 | 1.10 | 0.44 | 0.92 | 0.73 | 1.17 | 0.55 | 0.15 | 0.00 | 155.98 | 0.36 | 0.12 | 0.107 | -0.428 | 0.642 | 0.48 | 0.40 | - | - |
| | | CD | 0.89 | 0.74 | 1.06 | 0.18 | 0.31 | 0.85 | 0.69 | 1.04 | 0.11 | 0.81 | 0.60 | 1.08 | 0.25 | 0.04 | 0.00 | 812.05 | 0.29 | 0.12 | 0.185 | -0.505 | 0.874 | 0.37 | 0.32 | - | - |
| | | UC | 1.00 | 0.84 | 1.18 | 0.97 | 0.82 | 0.98 | 0.80 | 1.20 | 0.85 | 0.95 | 0.74 | 1.22 | 0.71 | 1.35 | 0.00 | 527.99 | 0.85 | 0.19 | -0.020 | -0.702 | 0.663 | 0.91 | 0.82 | - | - |
| Total fatty acids | 8 | IBD | 0.94 | 0.85 | 1.04 | 0.21 | 0.27 | 0.94 | 0.82 | 1.07 | 0.34 | 0.85 | 0.70 | 1.04 | 0.16 | 1.19 | 0.46 | 3.11 | 0.67 | 0.91 | -0.024 | -0.102 | 0.053 | 0.48 | 0.27 | - | - |
| | | CD | 0.85 | 0.75 | 0.98 | 0.02 | 0.02 | 0.89 | 0.74 | 1.06 | 0.18 | 0.89 | 0.71 | 1.13 | 0.37 | 1.88 | 0.40 | 8.77 | 0.36 | 0.91 | -0.077 | -0.176 | 0.023 | 0.11 | 0.02 | - | - |
| | | UC | 0.98 | 0.86 | 1.12 | 0.79 | 0.99 | 0.97 | 0.83 | 1.14 | 0.72 | 0.95 | 0.78 | 1.16 | 0.63 | 0.77 | 0.54 | 1.09 | 0.11 | 0.91 | 0.023 | -0.077 | 0.122 | 0.60 | 0.99 | - | - |
